# Combined Methylome, Transcriptome and Proteome Analyses Document Rapid Acclimatization of a Bacterium to Environmental Changes

**DOI:** 10.3389/fmicb.2020.544785

**Published:** 2020-09-15

**Authors:** Abhishek Srivastava, Jayaseelan Murugaiyan, Juan A. L. Garcia, Daniele De Corte, Matthias Hoetzinger, Murat Eravci, Christoph Weise, Yadhu Kumar, Uwe Roesler, Martin W. Hahn, Hans-Peter Grossart

**Affiliations:** ^1^Leibniz-Institute of Freshwater Ecology and Inland Fisheries, Stechlin, Germany; ^2^Department of Functional and Evolutionary Ecology, University of Vienna, Vienna, Austria; ^3^Centre for Infectious Medicine, Institute for Animal Health and Environmental Hygiene, Freie Universität Berlin, Berlin, Germany; ^4^Department of Biotechnology, SRM University-AP, Guntur, India; ^5^Research and Development Center for Marine Biosciences, Japan Agency for Marine-Earth Science and Technology, Yokosuka, Japan; ^6^Department of Biology and Environmental Science, Linnaeus University, Kalmar, Sweden; ^7^Institute of Chemistry and Biochemistry, Freie Universität Berlin, Berlin, Germany; ^8^Eurofins Genomics Europe Sequencing GmbH, Konstanz, Germany; ^9^Research Department for Limnology, University of Innsbruck, Mondsee, Austria; ^10^Institute for Biochemistry and Biology, Potsdam University, Potsdam, Germany

**Keywords:** DNA modification, gene expression, freshwater heterotrophic bacteria, UV radiation, purifying selection

## Abstract

*Polynucleobacter asymbioticus* strain QLW-P1DMWA-1^T^ represents a group of highly successful heterotrophic ultramicrobacteria that is frequently very abundant (up to 70% of total bacterioplankton) in freshwater habitats across all seven continents. This strain was originally isolated from a shallow Alpine pond characterized by rapid changes in water temperature and elevated UV radiation due to its location at an altitude of 1300 m. To elucidate the strain’s adjustment to fluctuating environmental conditions, we recorded changes occurring in its transcriptomic and proteomic profiles under contrasting experimental conditions by simulating thermal conditions in winter and summer as well as high UV irradiation. To analyze the potential connection between gene expression and regulation via methyl group modification of the genome, we also analyzed its methylome. The methylation pattern differed between the three treatments, pointing to its potential role in differential gene expression. An adaptive process due to evolutionary pressure in the genus was deduced by calculating the ratios of non-synonymous to synonymous substitution rates for 20 *Polynucleobacter* spp. genomes obtained from geographically diverse isolates. The results indicate purifying selection.

## Introduction

The model bacterium *Polynucleobacter asymbioticus* strain QLW-P1DMWA-1^T^ (Meincke et al., 2012) belongs to the class Gammaproteobacteria (Parks et al., 2018). The genus *Polynucleobacter* mainly represents a group of highly successful heterotrophic planktonic bacteria (Jezberová et al., 2010) inhabiting freshwater ecosystems (lakes, ponds, and streams) across all climatic zones and across all continents (Hahn et al., 2015). The genus also includes some obligate endosymbionts of ciliates, which represent evolutionarily derived stages (Boscaro et al., 2013). The strain investigated here persistently inhabits a small and shallow acidic Alpine pond in Austria (Hahn et al., 2005, 2012, 2016; Hoetzinger et al., 2017) that is covered by ice for 6 months of the year and, during the warmer season, undergoes pronounced diurnal fluctuations in thermal and irradiation conditions (Hahn et al., 2012).

Previous investigations revealed that the global ubiquity of *Polynucleobacter* in freshwater ecosystems results from ecological diversification (Jezbera et al., 2011; Hahn et al., 2012). Moreover, the small cell size of the ultramicrobacteria related to the genus *Polynucleobacter* is reportedly an advantage: flagellate predation was weak if not completely absent (Boenigk et al., 2004). Ultramicrobacteria (cell volume < 0.1 μm^3^) in general are numerically dominant organisms in many freshwater ecosystems (Proctor et al., 2018). They display extreme (e.g., LD12 *Alphaproteobacteria* and acI *Actinobacteria*) or moderate genome streamlining (e.g., genus *Polynucleobacter*) (Hahn et al., 2005; Newton et al., 2007; Salcher et al., 2011; Neuenschwander et al., 2017). Finally, this strain exhibits a non-motile planktonic lifestyle with limited metabolic plasticity: its members lack motility and quorum sensing genes with a very low number of signal transduction-related genes (Hahn et al., 2012). Our expectation therefore was that only minimal metabolic adjustment occurs in this strain. We strove to cover the majority of the molecular cause and effects in this strain’s adjustment to varying stress scenarios. A standard transcriptome and proteome study was designed to determine the underlying regulatory mechanisms and metabolic changes. At the same time, the presence of several methyltransferases in the genome^1^ prompted us to test whether any one of them could enable DNA modification that would possibly be involved in gene expression followed by metabolic adjustment.

DNA methylation is the most common epigenetic modification in nearly all life-forms, whereby the information is passed on from the parent cells to their daughter cells in addition to the genetic makeup (Jeltsch, 2002). Such modifications help bacteria defend their genome against viral infection using restriction-modification (R-M) systems, control DNA damage by mismatch-repair, coordinate gene regulation, control transposition events, and mediate host-pathogen associations (Wion and Casadesus, 2006; Sánchez-Romero et al., 2015). Many bacterial methylomes have been resolved at a single base resolution, but the efforts have largely been directed toward discovering methylated motifs and describing restriction-modification systems (Fang et al., 2012; Murray et al., 2012; Krebes et al., 2013; Lluch-Senar et al., 2013; Lee et al., 2015; Blow et al., 2016; Hiraoka et al., 2019). We, however, focus on testing the co-occurrence of methylation on the bacterial genome and gene expression. This approach enables us to assess whether the DNA methylation patterns play a role for the cell’s flexibility in manifesting important traits under different environmental circumstances.

Our study therefore included pinpointing molecular candidates responsible for the strain’s acclimatization in its natural habitat. We chose distinct experimental parameters for this strain, i.e., incubation scenarios at 4°C, at 26°C, and at 26°C with UV irradiation (hereafter denoted as 26°C^∗^). These experimental conditions reflect the abiotic factors common in the strain’s Alpine habitat (Hahn et al., 2012).

## Materials and Methods

### Investigated Strains and Genomes

The strain QLW-P1DMWA-1^T^ was used for stressor-related experiments as detailed below. Twenty other genomes of freshwater strains belonging to *Polynucleobacter* spp. were also used for this study. See Supplementary Table S1 for more details. Their genomes were used to assess the natural selection process operating in all these strains.

### Background Information on the Criteria for Selecting the Experimental Conditions and Details of the Bacterial Growth Conditions

**FIGURE 1:**
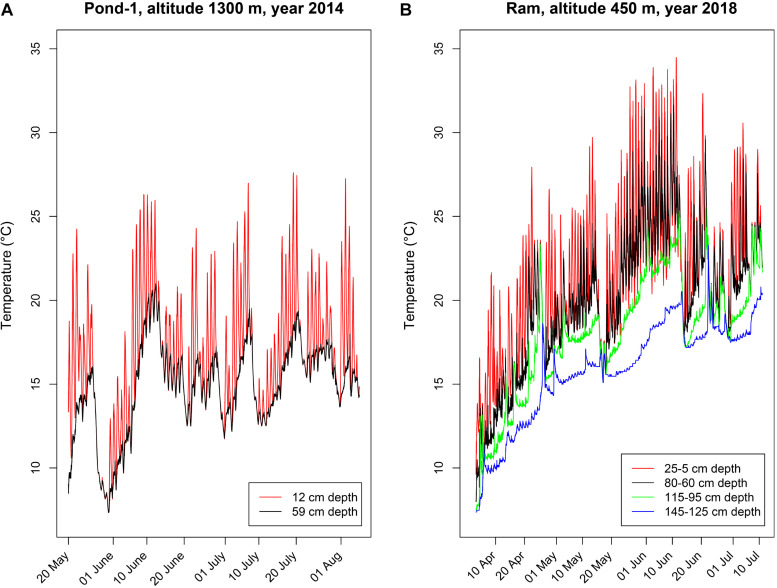
Depth-specific time series of water temperatures in two shallow freshwater systems inhabited by *Polynucleobacter asymbioticus*. Loggers deployed in different water depths measured temperatures in 30-min intervals. **(A)** Water temperatures measured in Pond-1, the home habitat of strain QLW-P1DMWA-1^T^. This shallow pond (maximum depth ∼ 1.2 m) is located at an altitude of 1300 m. **(B)** Water temperatures measured in four different water depths in a shallow (maximum water depth 1.5 m) bog pond located in Schönramer Moor. Designated here as Ram, this pond is located at an altitude of 450 m and 35 km away from Pond-1. The water level dropped by 20 cm during the investigation period in summer, which changed the water depths in which the loggers were moored. Note the diurnal changes in water temperature in the upper water layers, which frequently show amplitudes of more than 5°C of warming and nocturnal cooling with mixing.

The species *Polynucleobacter asymbioticus* typically inhabits shallow bog ponds (Hahn et al., 2012), which are characterized by rather unstable environmental conditions (Figures 1A,B), with strong diurnal changes in the upper water layers (<50 cm) and more stable conditions in deeper layers (>50 cm). This habitat type can be characterized as polymictic, slightly acidic (typically pH 5–6), shallow (max. depths usually 1–1.5 m) freshwater systems. We compared the temperature parameters of a high altitude (1300 m, Pond-1) and a low altitude (450 m, Schönramer Moor) Alpine habitat of *Polynucleobacter* bacteria that are 35 km apart. During calm and sunny days, the upper water layers quickly warm up (frequently by up to 5–8°C) and thermally stratify. Although, upper layers of Pond-1 (strain’s home habitat) can reach up to 26°C, the temperature is frequently higher in the lower altitude ponds during the summer where this species is also found (Figures 1A,B). Nocturnal cooling causes mixing of the upper water layers, but such mixing usually does not extend down to the lower layers. Thunderstorms with strong winds or cool weather periods cause complete mixing of these water bodies. Such complete mixing events take place several times during spring to late fall. The unstable environmental conditions result in sudden changes in growth conditions for the bacteria in the water columns of such ponds. Beyond the changes in water temperature, sudden strong changes in UV intensities also occur during the diurnal mixing cycles (Hahn et al., 2012).

The bacterial samples were cultured in 500 ml R2A liquid media in sterile UV-permeable polyethylene bags (∼35% permeability; Whirl-Pak^®^, Nasco). Experiments were performed in triplicates. Initial bacterial inoculum was transferred from pre-culture to the 500 ml main culture in the nine bags, which were kept on constant rotation of 200 rpm at 26°C. All bacterial samples were initially allowed to grow in an incubator maintained at 26°C. During this time the optical density (OD) at a wavelength of 575 nm approached 0.10. Afterward, the bags were distributed according to their planned treatment, i.e., (a) 26°C incubation continued for three bags, (b) three other bags were transferred to the 4°C incubator at 200 rpm, (c) radiation (UV) experiment was conducted with the remaining three bags, which were also maintained at 26°C (26°C^∗^). Humic substances stain the water of the home habitat Pond-1, thus the UV intensity is already reduced in deeper water layers (probably at 10–50 cm). Due to wind-driven mixing, the cells may cycle through different water depths with different UV intensities. Accordingly, only during a calm day with strong thermal stratification of the water column would cells in the top centimeters (i.e., a small fraction of the population) experience the natural UV intensity over a period of a whole day. We therefore opted for a shorter UV exposure duration. For the UV irradiation experiment, all three bags containing bacterial samples were transferred from the 26°C incubator to a position that was 40 cm from five parallel 100W ARIMED^®^ B UV lamps (Cosmedico^®^, Germany) for 30 min of exposure two times a day. At this distance, the spectral irradiance readings of UV lamps by the spectro-radiometer were 3 and 97 Wm^–2^ for the UVB and UVA range, respectively. Samples were harvested when the optical density (OD) values of the media (at wavelength 575 nm) approached 0.2 (late logarithmic/early stationary phase). Harvesting at this OD best resembles the state in which they are found in their natural setting, where they live a non-motile planktonic lifestyle (Hahn et al., 2012). 500 μl of UV-treated bacterial sample was spread on an R2A agar-media-containing plate. This produced viable bacterial colonies in 5 days at room temperature, confirming that radiation was not provided at lethal dose. Every experimental bag yielded 3 × 150 ml of bacterial samples that were harvested for the methylome, transcriptome and proteome studies.

### Sequencing and Methylome Analysis

Sample preparation and sequencing were performed at GATC Biotech (Constance, Germany) using the PacBio RSII system (Pacific Biosciences, San Diego). PacBio libraries with 8–12 kb insert size were prepared and sequenced on 1–2 SMRT (Single Molecule, Real-Time) cells with 120-min movie length and MagBead loading. For each sample, about 100x average coverage per genome was achieved (minimum >25× per strand). Depth coverage varied (∼54–∼70) in the three samples (Supplementary Figure S1). The base modification was analyzed with SMRT Portal 2.1 (Pacific Biosciences, San Diego).

SMRT sequencing reads from 4°C, 26°C, and 26°C plus UV-treated samples (26°C^∗^) were mapped against the *Polynucleobacter asymbioticus* reference genome down-loaded from NCBI (Accession Nr. NC_009379) using the BLASR mapper^2^ and the Pacific Biosciences SMRTAnalysis pipeline^3^ using the standard mapping protocol. Interpulse durations (IPDs) were measured as described by Flusberg et al. (2010) for all pulses aligned to each position in the reference sequence. IPD is used to determine if a DNA nucleobase is modified. This is achieved by monitoring a real time progression of DNA polymerase while it incorporates fluorescently labeled nucleotides. This is followed by detecting a fluorescence pulse and obtaining the kinetics of polymerase translocating toward the next base on a DNA template. The pulse extinguishes when the fluorophore attached to the terminal phosphate of a nucleotide is clipped away by the progressing polymerase. Modified bases affect the kinetics of DNA polymerase, and IPD increases when the base is methylated because the time needed for next base incorporation into the active site of the enzyme differs from the unmethylated site (Flusberg et al., 2010). Delayed incorporation of nucleotide when a methylated base is encountered eventually helps calculate the IPD ratio. IPDs were normalized by calculating the ratio of the IPD in the 4°C, 26°C, and 26°C^∗^ samples to the IPD of a control (the ‘IPD ratio’).

### Transcriptomic Study

An Agilent 60-mer microarray containing the *P. asymbioticus* str. QLW-P1DMWA-1^T^ genome was designed by the company Source BioScience GmbH. The 8 × 15K microarrays have 2123 genes of *P. asymbioticus* str. QLW-P1DMWA-1^T^ represented by seven 60-mer oligonucleotide probes on average. Additionally, 536 Agilent control probes were spotted onto the array for intra-array reproducibility measurements and against 10 different mRNA spike-in control transcripts. For the design, the Agilent web-based design-tool e-array was used, and the arrays were printed with Agilent SurePrint^®^ technology. The RNA was extracted using the NucleoSpin^®^ RNA kit (Macherey-Nagel GmbH & Co. KG; Düren, Germany) according to the manufacturer’s recommendations. The extracted RNA was quantified using a Nanodrop-ND-1000 (PEQLAB Biotechnologie GmbH, Erlangen, Germany), and RNA integrity was evaluated with the Agilent 2100 Bioanalyzer and RNA 6000 Nano Kit (Agilent Technologies, Böblingen, Germany). Cyanine-3 (Cy3-) labeled cRNA was prepared from 0.2 μg total RNA using the Quick Amp Labeling Kit, One-Color (Agilent Technologies, Böblingen, Germany) according to the manufacturer’s instructions. The full spectrum Multistart Primer (BioCat GmbH, Heidelberg, Germany) was used for the RNA isolated from the Gram-negative bacteria instead of the oligo-dT primer. Samples were purified with the RNeasy Mini Kit (QIAGEN, Hilden, Germany), and the cRNA yield and the Cy3 incorporation were determined with the Nanodrop-ND-1000 (PEQLAB Biotechnologie GmbH, Erlangen, Germany). 625 ng of cy-labeled cRNA was fragmented at 60°C for exactly 30 min in a volume of 25 μl containing 1 × Agilent fragmentation buffer and 2 × Agilent blocking agent. After incubation, 25 μl 2 × Agilent hybridization buffer was added to the sample and 40 μl were hybridized on the Agilent *P. asymbioticus* 8 × 15K microarray for 17 h at 65°C and at 10 rpm in an Agilent hybridization oven. After hybridization the microarray was dissembled at room temperature in Gene Expression Wash Buffer 1 (Agilent Technologies, Böblingen, Germany) and then washed for 1 min at room temperature with Gene Expression Wash Buffer 1, and for 1 min at 37°C with Gene Expression Wash Buffer 2. Acetonitrile was used to dry the array. The microarray was scanned with the Agilent DNA Microarray scanner (G2565CA) using the following settings: scan area 61 × 21.6 mm, scan resolution 3 μm, Dye Channel Green, and PMT 100%. The scanned array was extracted with the Feature Extraction software (version 10.5.1.1), and group comparisons were performed with the analysis pipeline of the company Source Bioscience GmbH to detect those genes that are significantly differentially expressed. Raw data were analyzed, normalized, and finally evaluated with *t*-tests (unequal variance), principal component analysis (PCA), and hierarchical clustering. The *P*-value cut-off was set to 0.05, and values less than that were considered significant in our study.

### Proteomic Study

We used an Orbitrap mass analyzer to identify the proteins present in our three experimental samples, i.e., 26°C, 26°C^∗^, and 4°C-incubated bacterial culture. The cells were harvested and the whole cell proteins were extracted as described elsewhere (Murugaiyan et al., 2016). The cell pellet was reconstituted with 500 μl of 20 mM HEPES (pH 7.4) and subjected to sonication on ice for 1 min (cycle, 1.0; amplitude, 100%; UP100H; Hielscher Ultrasound Technology, Teltow, Germany). The suspension was centrifuged at 11,290 × *g* for 5 min at 4°C and the clear supernatant was collected. The protein content was estimated using a modified Bradford’s method (Bio-Rad, Munich, Germany). The protein estimation consistency was verified using a volume of protein extraction containing 5 μg of protein mixing with 10 μL sample loading buffer, heated for 5 min at 60°C and subjected to sodium dodecyl sulfate polyacrylamide gel electrophoresis (SDS-PAGE) (stacking gel 4%, and separating gel 12%). The protein bands were visualized using Coomassie Brilliant Blue staining (Candiano et al., 2004).

### *In-Solution* Trypsin Digestion and Mass Spectrometry (MS) Analysis

*In-solution* trypsin digestion was carried out with incubation steps at room temperature under gentle shaking. 10 μg of bacterial proteins were precipitated with acetone and reconstituted in 20 μl of denaturation buffer (6 M urea/2 M thiourea in 10 mM HEPES, pH 8.0). 0.2 μl of 10 mM dithiothreitol were added in 50 mM of ammonium bicarbonate (NH_4_HCO_3_) and incubated for 30 min. Thereafter, 0.4 μl of 55 mM iodoacetamide were added in NH_4_HCO_3_ and then incubated for 20 min. 0.4 μl of Lys-C protease (Sigma-Aldrich, Germany) solution (0.5 μg/μl in NH_4_HCO_3_) were added and incubated overnight. The urea concentration was diluted by adding 75 μl of NH_4_HCO_3_, whereby 0.4 μl of 0.5 μg/μL trypsin protease were added in 50 mM NH_4_HCO_3_. Following overnight incubation, trypsin digestion was stopped by adding 100 μl of 5% acetonitrile in 3% trifluroacetic acid. Overnight trypsin treatment was performed to obtain a better sequence coverage of all digested proteins.

After digestion, peptide samples were desalted by solid phase extraction (SPE) using C18 Empore^TM^ disks Stagetips (Supelco, Germany). Desalted peptide mixtures were separated by reversed phase chromatography using the Dionex UltiMate 3000 Nano LC on in-house manufactured 25 cm fritless silica microcolumns with an inner diameter of 100 μm. Columns were packed with ReproSil-Pur C18-AQ 3 μm resin. Peptides were separated on a 5–60% acetonitrile gradient (90 min) with 0.1% formic acid at a flow rate of 350 nl/min. Eluting peptides were ionized online by electrospray ionization and transferred into a Thermo Scientific^TM^ LTQ-Orbitrap Velos^TM^ mass spectrometer (Thermo Fisher Scientific, Germany).

The LTQ-Orbitrap was operated in the positive mode to record full scan MS spectra (from m/z 300–1700) in the Orbitrap mass analyzer at a resolution of *R* = 60,000. This was followed by isolation and fragmentation of the 20 most intense ions in the LTQ part by collision-induced dissociation. The MaxQuant (version 1.3.0.5) software suit was used to process the raw MS files. The search engine ANDROMEDA (Cox et al., 2011) was utilized to search the peak list files against forward and backward protein sequences of *P. asymbioticus* down-loaded from the Uniprot database and 248 frequently observed laboratory contaminants. Initial maximum precursor and fragment mass deviations were set to 7 ppm and 0.5 Da, respectively. Methionine oxidation/acetylation of peptide N-termini and cysteine carbamidomethylation were set as variable and fixed modification, respectively, for the search. Furthermore, enzyme specificity was set to trypsin and a maximum of two missed cleavages was allowed for searching. The target-decoy-based false discovery rate (FDR) for peptide and protein identification was set to 1% for peptides and proteins, and the minimum peptide length was set to six amino acids. Precursor mass tolerance was set to 20 ppm. The mass tolerance for fragment ions was set to 0.5 Da. Protein identification was based on the detection of more than one unique peptide specific for a protein. MS-Quantification of proteins was performed using the label-free quantification algorithm of the MaxQuant software package. The freely available software Perseus (version 1.4.1.3, Max-Planck-Institute of Biochemistry, Martinsried, Germany) was used to compare the peak intensity across the whole set of measurements to obtain the quantitative measurements for all of the peptides in the sample. The normalized protein intensities from the MaxQuant analysis were imported and transformed into logarithmic scale with base two. The missing values were replaced with the value of the lowest intensity. The proteins were quantified and statistical significance determined using two-sample Student *t*-test and FDR using the method of Benjamini–Hochberg (Benjamini and Hochberg, 1995). For further visualization, heat-map and principal component analysis (PCA) were computed.

### Melting Temperature Calculation

The melting temperature (*T*_m_) calculation was adopted from SantaLucia’s study on unified nearest-neighbor thermodynamic stability parameters (ΔG°_37_ or Gibb’s free energy at 37°C) of Watson–Crick base pairs in 1 M sodium chloride solution (SantaLucia, 1998) with 100-kb window size. Data were plotted to prepare circular maps using Circos software package v0.69^4^.

### Non-synonymous Substitution Rate (*K*a) and Synonymous Substitution Rate (*K*s) Calculation

*K*a/*K*s values were calculated with the KaKs calculator 2.0 (Wang et al., 2010) using both an approximate method (γ-MYN) and a maximum-likelihood method based on a substitution model selected from a set of candidate models (MS). Gene pairs with less than three substitutions and *K*a*K*s values with *p*-values higher than 0.05 were removed from our study.

The GEO accession number corresponding to microarray data is GSE98129.

The mass spectrometry proteomics data were deposited at the ProteomeXchange Consortium (Perez-Riverol et al., 2019) via the PRIDE partner repository with the dataset identifier PXD020103.

## Results and Discussion

### Transcriptome and Proteome Analyses

#### 4°C-Incubated Sample

**FIGURE 2:**
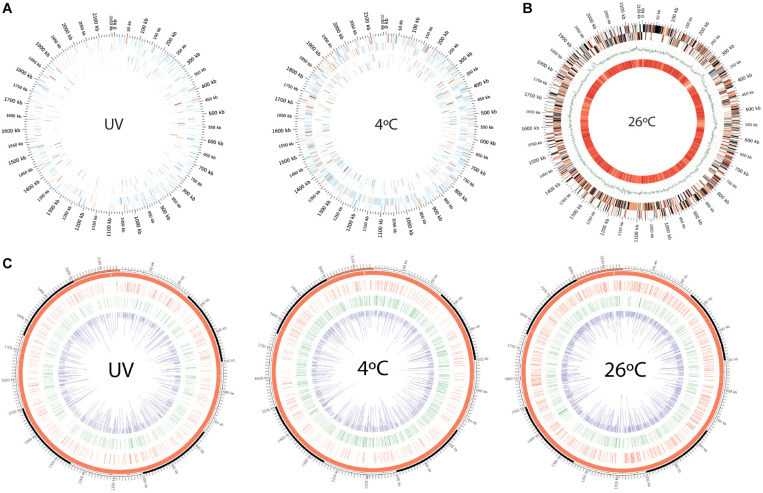
**(A)** Circos plot depiction of differentially expressed genes and proteins in cold treatment and UV-irradiated bacterial samples compared against 26°C treatment, as revealed by microarray and LC-MS. Outermost tick marks: bacterial genome of 2.15-Mb. Concentric tracks from outer to inner: sense and antisense strand, respectively. **(B)** Circos plot representation of differentially expressed genes in bacteria incubated at 26°C (in relative comparison to 4°C-incubated cells) and averaged melting energy distribution in bacterial genome. The outer two concentric tracks represent sense and antisense strands. Color-coding: red (up-regulated genes) with transient color (expressed transcript gradients) toward blue (down-regulated genes), black genes are undetected in microarray analysis. Green GC skew curve is then followed with the innermost track, representing average negative DNA melting energy with 100-kb scanning window size (bright orange to pale yellow represent high to low values). **(C)** Circos plots showing the kinetic variation measured as IPD ratio recorded across the *Polynucleobacter asymbioticus* strain QLW-P1DMWA-1^T^ genome. Red and green tracks represent the genome locations where 4mC and 6mA modification motifs were recorded, respectively. Inner (purple) track: IPD ratios of the corresponding modifications recorded across the genome.

When incubated at 4°C (winter conditions), only 10% of the total 2088 protein-coding genes were up-regulated (Figure 2A, Supplementary Figure S2, and Supplementary Tables S2a,b). Bacterial responses to cold temperature have been investigated and summarized in detail elsewhere (Barria et al., 2013), and our findings agree with earlier reports on maintaining membrane fluidity, efficient transcription or translation, enriched chaperones, oxidative stress compensation, and cell shape maintenance. Several key features are summarized below that might be helpful for strain QLW-P1DMWA-1^T^ to survive in alpine winter.

#### General Low Temperature Anti-stress Proteins

Cold treatment induced a nine-fold higher expression of cold-shock protein (CSP) (*p* = 9.4 × 10^–6^) (Table 1 and Supplementary Table S3). Low temperatures can impose secondary folding of the mRNA, and CSP can therefore efficiently counteract such folding, which impedes ribosomal access to the mRNA (Giuliodori et al., 2004). Moreover, we observed that the fatty acid desaturase-encoding gene was moderately over expressed (+1.0 fold; *p* = 0.001) in the cold-treated samples. Such a feature may help bacteria to acclimatize in near-freezing lakes during the winter season.

**TABLE 1:** Quantitative proteome analysis. **(A)** Tabulation of 50 strongest up-regulated proteins obtained from 4°C-incubated cells. **(B)** Tabulation of 50 strongest down-regulated proteins obtained from 4°C-incubated cells. **(C)** Tabulation of 50 strongest up-regulated proteins obtained from UV irradiated (26°C-incubated) cells (26°C*). **(D)** Tabulation of 50 strongest down-regulated proteins obtained from UV irradiated (26°C-incubated) cells (26°C*).

(A)			
4°C			
Locus tag	Up-regulated proteins	Log2 fold changes	*p*-Value
Pnuc_0586	Cold-shock DNA-binding protein family	9.06	0.000009
Pnuc_0379	Peptidylprolyl isomerase (EC 5.2.1.8)	7.74	0.000000
Pnuc_0443	Carboxylic ester hydrolase (EC 3.1.1.-)	7.56	0.000008
Pnec_0187	Peroxiredoxin (EC 1.11.1.15)	7.51	0.000269
Pnuc_1435	Secretory lipase	6.05	0.000076
Pnuc_2054	CHRD domain containing protein	5.79	0.011172
Pnuc_1229	Ribosome maturation factor RimP	5.46	0.003365
Pnuc_0323	Phytanoyl-CoA dioxygenase	4.42	0.012292
Pnuc_1343	Transglutaminase domain protein	4.23	0.000000
Pnuc_1808	Transcriptional regulator, LuxR	4.04	0.000521
Pnuc_1898	DUF971 domain-containing protein	3.54	0.000096
Pnuc_1418	7-Carboxy-7-deazaguanine synthase	3.52	0.006376
Pnuc_1758	Transglut_core2 domain-containing protein	3.50	0.000508
Pnuc_1342	ATP-dependent RNA helicase RhlE	3.46	0.000013
Pnuc_1230	Pseudouridine synthase (EC 5.4.99.-)	3.36	0.000493
Pnuc_0666	Alanine dehydrogenase (EC 1.4.1.1)	3.08	0.000417
Pnuc_0679	Uncharacterized protein	2.90	0.000078
Pnuc_0132	Phasin family protein	2.64	0.010966
Pnuc_0659	DEAD/DEAH box helicase domain protein	2.58	0.000052
Pnuc_1751	Uncharacterized protein	2.57	0.000013
Pnuc_1732	Peptidyl-prolyl *cis–trans* isomerase	2.53	0.000054
Pnuc_0820	ABC transporter related protein	2.52	0.000072
Pnuc_0643	Penicillin amidase	2.48	0.000163
Pnuc_1260	YVTN family beta-propeller repeat protein	2.45	0.000195
Pnuc_1650	Peptidase S16, lon domain protein	2.43	0.000975
Pnuc_1414	SOUL heme-binding protein	2.40	0.001025
Pnuc_0757	Transcriptional regulator, GntR family	2.23	0.000988
Pnuc_1961	Lipoyl synthase (EC 2.8.1.8)	2.20	0.000787
Pnuc_1490	Co-chaperone protein HscB homolog	2.19	0.001331
Pnuc_1637	NAD-dependent epimerase/dehydratase	2.17	0.009716
Pnuc_1224	GTP-binding protein TypA	2.11	0.000017
Pnuc_1980	Methionine synthase (B12-dependent)	2.11	0.000110
Pnuc_1453	Metal dependent phosphohydrolase	1.99	0.002009
Pnuc_1869	Chaperone SurA	1.98	0.000033
Pnuc_0657	Uroporphyrinogen-III synthase (EC 4.2.1.75)	1.96	0.011631
Pnuc_1708	3-Mercaptopyruvate sulfurtransferase	1.94	0.000033
Pnuc_0196	GTPase Obg	1.87	0.000303
Pnuc_0173	Peroxiredoxin (EC 1.11.1.15)	1.87	0.000706
Pnuc_0162	UDP-*N*-acetylmuramyl-tripeptide synthetase	1.79	0.000235
Pnuc_1809	Histidine kinase	1.75	0.000040
Pnuc_0303	GDP-L-fucose synthase (EC 1.1.1.271)	1.73	0.004093
Pnuc_1699	DNA primase (EC 2.7.7.-)	1.69	0.010299
Pnuc_0755	Uncharacterized protein	1.63	0.004281
Pnuc_0169	D-Alanine–D-alanine ligase (EC 6.3.2.4)	1.59	0.013978
Pnuc_0543	Protein tyrosine/serine phosphatase	1.58	0.001057
Pnuc_0644	Pantothenate synthetase (PS) (EC 6.3.2.1)	1.57	0.007072
Pnuc_1209	Uncharacterized protein	1.57	0.007234
Pnuc_1231	Segregation and condensation protein B	1.50	0.012714
Pnuc_1184	Amidase	1.47	0.007345
Pnuc_0935	ATP-dependent Clp protease ClpX	1.46	0.002080

**(B)**			

**4°C**			

**Locus tag**	**Down-regulated proteins**	**Log2 fold changes**	***p*-Value**

Pnuc_1043	NADH-quinone oxidoreductase subunit I	−5.38	0.020876
Pnuc_1177	tRNA-U16,U17-dihydrouridine synthase	−5.12	0.000011
Pnuc_1261	ATP-dependent helicase HrpA	−4.98	0.000004
Pnuc_1865	D,D-Heptose 1,7-bisphosphate phosphatase	−4.58	0.000275
Pnuc_0332	Uncharacterized protein	−4.57	0.000008
Pnuc_0167	MurG transferase	−4.35	0.008714
Pnuc_1380	Uncharacterized protein	−4.30	0.000007
Pnuc_1862	Apolipoprotein *N*-acyltransferase	−4.07	0.000166
Pnuc_0454	Cytochrome c oxidase, cbb3-type, subunit II	−4.00	0.047962
Pnuc_0143	Uncharacterized protein	−3.91	0.045963
Pnuc_0456	Cbb3-type cytochrome c oxidase subunit	−3.87	0.033499
Pnuc_0370	4-Hydroxyphenylacetate 3-hydroxylase	−3.28	0.037016
Pnuc_1185	ABC transporter, substrate binding protein	−2.98	0.037609
Pnuc_1407	Heavy metal translocating P-type ATPase	−2.21	0.001641
Pnuc_1026	Acriflavin resistance protein	−1.65	0.000232
Pnuc_1325	Uncharacterized protein UPF0065	−1.65	0.008220
Pnuc_2003	*S*-Adenosylmethionine synthase	−1.53	0.032302
Pnuc_0357	Amino acid ABC transporter ATP-binding protein	−1.49	0.001101
Pnuc_1012	Dihydropteroate synthase	−1.46	0.021628
Pnuc_1417	Uncharacterized protein	−1.45	0.015339
Pnuc_1799	NAD(P) transhydrogenase subunit beta	−1.41	0.000007
Pnuc_0818	Acyl-CoA dehydrogenase domain protein	−1.33	0.001235
Pnuc_0676	TonB-dependent receptor	−1.32	0.022800
Pnuc_1792	Protein-export membrane protein SecF	−1.32	0.000016
Pnuc_0193	Farnesyltranstransferase (EC 2.5.1.29)	−1.31	0.001025
Pnuc_0553	Pyruvate dehydrogenase (cytochrome)	−1.30	0.002854
Pnuc_0447	Chaperone protein ClpB	−1.28	0.005296
Pnuc_2048	Uncharacterized protein	−1.26	0.024349
Pnuc_0594	2-Hydroxy-3-oxopropionate reductase	−1.25	0.001032
Pnuc_1559	Methylmalonate-semialdehyde dehydrogenase	−1.23	0.003859
Pnuc_0539	Citryl-CoA lyase (EC 4.1.3.34)	−1.23	0.011722
Pnuc_1430	Uncharacterized protein	−1.21	0.034432
Pnuc_1351	Fumarylacetoacetate (FAA) hydrolase	−1.19	0.014934
Pnuc_1769	Chaperone protein DnaK (HSP70)	−1.18	0.011158
Pnuc_1275	Uncharacterized protein	−1.17	0.025918
Pnuc_1797	Alanine dehydrogenase/PNT domain protein	−1.16	0.000427
Pnuc_1385	Superoxide dismutase (EC 1.15.1.1)	−1.15	0.019111
Pnuc_0728	Sulfide dehydrogenase (flavocytochrome)	−1.12	0.029945
Pnuc_0417	Uncharacterized protein	−1.10	0.000601
Pnuc_1192	Urease accessory protein UreE	−1.10	0.025236
Pnuc_1101	Uncharacterized protein	−1.09	0.002920
Pnuc_1677	Heavy metal translocating P-type ATPase	−1.07	0.014295
Pnuc_0852	Serine acetyltransferase (EC 2.3.1.30)	−1.06	0.000099
Pnuc_1562	Propionyl-CoA synthetase (EC 6.2.1.17)	−1.06	0.008504
Pnuc_1485	Uncharacterized protein	−1.06	0.003455
Pnuc_1126	Uncharacterized protein	−1.04	0.003297
Pnuc_1153	Formyltetrahydrofolate deformylase	−1.04	0.010592
Pnuc_1502	Acetyl-CoA acetyltransferase	−1.03	0.004161
Pnuc_0358	Amino acid ABC transporter ATP-binding protein	−1.03	0.006260
Pnuc_0085	Cytochrome c553-like protein	−1.03	0.000713

**(C)**			

**4°C**			

**Locus tag**	**Down-regulated proteins**	**Log2 fold changes**	***p*-Value**

Pnuc_0379	Peptidylprolyl isomerase (EC 5.2.1.8)	8.02	0.000006
Pnuc_0443	Carboxylic ester hydrolase (EC 3.1.1.-)	7.98	0.000000
Pnuc_1376	Rubrerythrin	6.52	0.008427
Pnuc_1435	Secretory lipase	6.24	0.000001
Pnuc_2069	Uncharacterized protein	6.09	0.000000
Pnuc_2054	CHRD domain containing protein	6.01	0.005885
Pnuc_2053	Catalase-related peroxidase (EC 1.11.1.-)	5.25	0.000002
Pnuc_1810	D-Alanyl-D-alanine carboxypeptidase	4.69	0.000178
Pnuc_1815	Fructose-1,6-bisphosphate aldolase	4.48	0.030060
Pnuc_2031	Lipid A biosynthesis acyltransferase	4.46	0.001167
Pnuc_1676	Conserved secreted protein	4.14	0.006069
Pnuc_0888	Uncharacterized protein	4.13	0.000000
Pnuc_1222	RNA binding S1 domain protein	3.84	0.000018
Pnuc_1229	Ribosome maturation factor RimP	3.73	0.002870
Pnuc_0679	Uncharacterized protein	3.46	0.000000
Pnuc_0487	Pirin domain protein	3.43	0.031825
Pnuc_0586	Cold-shock DNA-binding protein family	3.41	0.000000
Pnuc_0541	Glutathione *S*-transferase	3.40	0.005543
Pnuc_1343	Transglutaminase domain protein	3.21	0.000021
Pnuc_1731	4-Hydroxy-3-methylbut-2-enyl diphosphate reductase	3.21	0.116034
Pnuc_0643	Penicillin amidase	3.21	0.000010
Pnuc_1808	Two component transcriptional regulator, LuxR	3.19	0.000008
Pnuc_1751	Uncharacterized protein	3.18	0.000001
Pnuc_2078	Methionyl-tRNA formyltransferase	3.15	0.029296
Pnuc_1414	SOUL heme-binding protein	2.89	0.000116
Pnuc_1758	Transglut_core2 domain-containing protein	2.85	0.000839
Pnuc_1418	7-Carboxy-7-deazaguanine synthase	2.74	0.011066
Pnuc_1732	Peptidyl-prolyl *cis–trans* isomerase	2.66	0.000207
Pnuc_0657	Uroporphyrinogen-III synthase (EC 4.2.1.75)	2.42	0.000339
Pnuc_1898	DUF971 domain-containing protein	2.36	0.005023
Pnuc_0391	HAD-superfamily hydrolase, subfamily IA	2.31	0.000160
Pnuc_1869	Chaperone SurA	2.29	0.000047
Pnuc_1650	Peptidase S16, lon domain protein	2.22	0.000017
Pnuc_1342	ATP-dependent RNA helicase RhlE	2.19	0.000275
Pnuc_1230	Pseudouridine synthase (EC 5.4.99.-)	2.17	0.003768
Pnuc_0839	DNA binding domain, excisionase family	2.14	0.000000
Pnuc_0244	Putative pre-16S rRNA nuclease (EC 3.1.-.-)	2.07	0.000224
Pnuc_1453	Metal dependent phosphohydrolase	1.99	0.001607
Pnuc_2008	Exodeoxyribonuclease III Xth (EC 4.2.99.18)	1.95	0.000799
Pnuc_1980	Methionine synthase (B12-dependent)	1.93	0.000120
Pnuc_0644	Pantothenate synthetase (PS) (EC 6.3.2.1)	1.92	0.003478
Pnuc_1324	Haloacid dehalogenase, type II	1.89	0.022058
Pnuc_0217	Shikimate dehydrogenase [NADP(+)]	1.83	0.000209
Pnuc_1131	Phage transcriptional regulator, AlpA	1.79	0.000486
Pnuc_0606	DNA polymerase III subunit alpha (EC 2.7.7.7)	1.76	0.013830
Pnuc_1133	*N*-6 DNA methylase	1.76	0.000066
Pnuc_1241	Uncharacterized protein	1.74	0.013508
Pnuc_0303	GDP-L-fucose synthase (EC 1.1.1.271)	1.74	0.004615
Pnuc_0757	Transcriptional regulator, GntR family	1.73	0.000935
Pnuc_0820	ABC transporter related protein	1.72	0.000181

**(D)**			

**4°C**			

**Locus tag**	**Down-regulated proteins**	**Log2 fold changes**	***p*-Value**

Pnuc_1261	ATP-dependent helicase HrpA	−4.98	0.000004
Pnuc_0543	Protein tyrosine/serine phosphatase	−4.27	0.000013
Pnuc_1718	SirA family protein	−3.91	0.000000
Pnuc_1043	NADH-quinone oxidoreductase subunit I	−2.46	0.000256
Pnuc_1407	Heavy metal translocating P-type ATPase	−2.21	0.001641
Pnuc_0454	Cytochrome c oxidase, cbb3-type, subunit II	−2.10	0.000007
Pnuc_0954	Phosphopantetheine-binding protein	−1.79	0.021560
Pnuc_1459	Succinyl-diaminopimelate desuccinylase	−1.72	0.000975
Pnuc_0930	Cytidylyltransferase	−1.63	0.003455
Pnuc_1060	Phosphatidylserine decarboxylase proenzyme	−1.57	0.006396
Pnuc_0553	Pyruvate dehydrogenase (Cytochrome)	−1.55	0.002755
Pnuc_1126	Uncharacterized protein	−1.53	0.009665
Pnuc_0910	Methylmalonyl-CoA mutase (EC 5.4.99.2)	−1.51	0.000243
Pnuc_0864	HipA domain protein	−1.49	0.003129
Pnuc_1354	Uncharacterized protein	−1.44	0.000743
Pnuc_1430	Uncharacterized protein	−1.31	0.000725
Pnuc_1984	Alcohol dehydrogenase, zinc-binding	−1.30	0.000613
Pnuc_1770	Protein GrpE (HSP-70 cofactor)	−1.26	0.000531
Pnuc_1630	Intracellular protease, PfpI family	−1.21	0.000173
Pnuc_1768	Chaperone protein DnaJ	−1.19	0.000369
Pnuc_1205	Uncharacterized protein	−1.19	0.023330
Pnuc_0913	Acetyl-CoA carboxylase, biotin carboxylase	−1.14	0.001123
Pnuc_1769	Chaperone protein DnaK (HSP70)	−1.14	0.000022
Pnuc_1545	Class II aldolase/adducin family protein	−1.13	0.009892
Pnuc_0840	2-Oxoglutarate dehydrogenase, E1 subunit	−1.11	0.000146
Pnuc_0126	Cytochrome b	−1.05	0.000333
Pnuc_1805	60 kDa chaperonin (GroEL protein)	−1.05	0.000000
Pnuc_0417	Uncharacterized protein	−1.05	0.000158
Pnuc_0152	Amidohydrolase (EC 3.5.1.32)	−1.03	0.002076
Pnuc_1196	Urease subunit gamma (EC 3.5.1.5)	−1.02	0.006508
Pnuc_0772	Tryptophan synthase beta chain (EC 4.2.1.20)	−0.99	0.012556
Pnuc_1138	Acetyl-coenzyme A synthetase	−0.99	0.012013
Pnuc_0912	Carboxyl transferase	−0.97	0.000049
Pnuc_1286	RNA-binding protein Hfq	−0.97	0.000455
Pnuc_0842	Dihydrolipoyl dehydrogenase (EC 1.8.1.4)	−0.97	0.000569
Pnuc_1400	4Fe–4S ferredoxin	−0.96	0.009343
Pnuc_1433	Phosphoenolpyruvate synthase (PEP synthase)	−0.95	0.003936
Pnuc_1063	Acetolactate synthase (EC 2.2.1.6)	−0.93	0.000031
Pnuc_0841	2-Oxoglutarate dehydrogenase, E2 subunit	−0.93	0.000228
Pnuc_0194	50S ribosomal protein L21	−0.92	0.025782
Pnuc_0403	Periplasmic serine endoprotease DegP-like	−0.92	0.000556
Pnuc_0690	DNA translocase FtsK	−0.92	0.048204
Pnuc_1111	Uncharacterized protein	−0.91	0.002288
Pnuc_0447	Chaperone protein ClpB	−0.90	0.000003
Pnuc_0970	Thioesterase superfamily protein	−0.90	0.007117
Pnuc_0594	2-Hydroxy-3-oxopropionate reductase	−0.90	0.000235
Pnuc_0655	Argininosuccinate lyase	−0.90	0.009229
Pnuc_0735	Dihydrolipoyllysine-residue acetyltransferase	−0.89	0.000333
Pnuc_1863	Glycine–tRNA ligase alpha subunit	−0.88	0.002340
Pnuc_1616	Malate:quinone-oxidoreductase	−0.88	0.008483

The upregulation of several chaperones, co-chaperones, and anti-oxidative stress factors indicates the necessity of keeping the cell and its molecular machineries operational at stressful low temperature. For example, chaperone HscA and co-chaperone HscB, which participate in iron–sulfur-cluster biosynthesis, were enriched (+0.93; *p* = 0.006 and +2.19; *p* = 0.001, respectively) (Vickery and Cupp-Vickery, 2007). We also observed that antioxidant-related genes are also up-regulated, e.g., superoxide dismutase Pnuc_1626 (+3.9 fold; *p* = 0.04), catalase Pnuc_2053 (+0.7 fold; *p* = 0.03), and alkyl hydroperoxide reductase Pnuc_1534 (+0.7 fold; *p* = 0.002). These antioxidant genes, amongst many located in this strain’s genome, might be cold induced (Smirnova et al., 2001). Similarly, lipoic acid is a cofactor for enzymes that are multicomponent dehydrogenases. Due to its solubility in lipid- or aqueous solutions, it functions as a wide-range antioxidant that can quench free radicals (Spalding and Prigge, 2010). Cold treatments led to a +2.2 fold (*p* = 0.001) higher detection of lipoyl synthase compared to warmer incubation of bacterial cells. The antioxidant peroxiredoxin (Pnuc_0173) was also enriched (+1.87 fold; *p* = 7 × 10^–4^) in cold-treated samples.

Another class of protein folding chaperones consists of peptidyl-prolyl isomerase (PPIase), which catalyses the isomerization reaction of the *cis–trans* configuration in peptide bonds (Shaw, 2002). Proteins generally contain *trans* configuration peptide linkages except proline amino acid, which exists in a *cis* configuration (Joseph et al., 2012). Therefore, prolyl isomerization is a rate-limiting step during protein folding, which is additionally affected by extreme temperatures leading to potential impaired folding during cold (Feller, 2018). Such mis-folding is avoided by PPIases, which help microbes to adapt to low temperature (Budiman et al., 2011). Our dataset clearly indicates that cold treatment induced PPIases in the cell: Pnuc_0379 - PpiC-type (+7.74 fold; *p* = 1 × 10^–8^), Pnuc_1732 - FKBP-type (+2.53 fold; *p* = 5.4 × 10^–5^), Pnuc_1869 - PpiC-type SurA (+1.98 fold; *p* = 3.3 × 10^–5^). Note, however, that a consecutive run of proline residues in an elongating polypeptide may cause increased stalling of ribosomes during the translation process. Such stalling is prevented by the elongation factor P (EF-P), which facilitates peptide-bond formation (Doerfel et al., 2013). Interestingly, we detected an elevated EF-P signal (+1.43 fold; *p* = 4 × 10^–4^) in our proteomic analysis, suggesting its important role in rapid protein biosynthesis.

### Cell Shape Maintenance

Bacterial cell wall integrity is crucial in cell shape maintenance and viability (Dörr et al., 2019). Importantly, an additional role of the cell wall in psychrophilic bacteria is protection against cold, as revealed by transcriptomic analyses in *Exiguobacterium sibiricum* (Rodrigues et al., 2008). Increased expression of peptidoglycan biosynthetic transcripts during cold treatment was explained by cell wall thickening, which possibly contributes to cell protection in the event of ice formation and related cell disruption (Rodrigues et al., 2008). We recorded moderately increased protein expression related to the cell wall biosynthetic pathway, which supports the cell protection notion [UDP-MurNAc-tripeptide synthetase (+1.79 fold; *p* = 2.3 × 10^–4^), UDP-*N*-acetylmuramoyl-tripeptide–D-alanyl-D-alanine ligase (+1.04 fold; *p* = 0.02), UDP-*N*-acetylmuramoyl-L-alanyl-D-glutamate synthetase (+0.56 fold; p = 0.01), D-alanylalanine synthetase (+1.59 fold; *p* = 0.01)]. Similarly, the structural protein MreB was enriched, which would help maintain bacterial cell shape (+1.3 fold; *p* = 1.6 × 10^–5^) at 4°C. This was also reported for cold-shock treated *Vibrio parahaemolyticus* (Chiu et al., 2008).

### GTP Homeostasis-Related Cellular Processes

The GTPase family proteins act as a sensor for the cellular GDP/GTP pools by cycling between the inactive GDP- and active GTP-bound state (Verstraeten et al., 2011). GTPase proteins also participate in diverse cellular processes that affect cell physiology (Verstraeten et al., 2011). For example, the GTPase Obg in *Escherichia coli* is involved in chromosomal partitioning (Kobayashi et al., 2001). Similarly, the GTPase Era is required in septum initiation and acts as a critical checkpoint regulator in the *E. coli* cell cycle (Britton et al., 1998). The GTPase FtsZ multimerization at the future cell division site forms a ring where other cytokinesis molecular machinery also assembles (Arjes et al., 2015). The GTPase TypA (tyrosine phosphorylation protein A) acts as a global regulator in bacteria and has been shown to be important in the survival and growth of *Sinorhizobium meliloti* and *E. coli* at low temperatures (Pfennig and Flower, 2001; Kiss et al., 2004). Interestingly, we observed increased protein signals of the above-mentioned GTPases in the cold-treated bacterial samples: Obg (+1.87 fold; *p* = 3 × 10^–4^), Era (+1.23 fold; *p* = 2.9 × 10^–3^), FtsZ (+1.04 fold; *p* = 3.2 × 10^–3^), TypA (+2.1 fold; *p* = 1.7 × 10^–5^).

In contrast, one GTPase, namely LepA (or, elongation factor 4) transcripts, were under-expressed (Pnuc_0404 fold; −1.3 fold, *p* = 0.02). The protein signal of LepA was also under-detected in the cold-treated sample, although non-significantly (−0.53 fold; *p* = 0.2). Interesting in this respect is that LepA translocates the ribosome complex toward one codon backward and helps to increase translation fidelity at low concentration only (≤0.3 LepA molecules per 70S ribosome subunits). Higher cellular concentrations of LepA, however, cause a non-productive translation process (Qin et al., 2006). Collectively, GTPases may act as a molecular switch that monitors intracellular GDP/GTP, and our study highlights their potential roles during bacterial cold stress.

It is also interesting to note that the *relA* (Pnuc_0828) transcript is down-regulated (−0.65 fold; *p* = 0.002) in cold treatment but remains nominally enriched at the protein level (+0.53 fold; *p* = 0.01). Such an expression mismatch might be due to post-transcriptional regulation of *relA*. Potential regulation of *relA* under-expression is discussed in the section “Methylation Features.” *relA* encodes for the stress responsive alarmone guanosine pentaphosphate [(p)ppGpp]. This is a nucleotide second messenger and its accumulation is associated with stress survival. Such a signaling molecule can bind directly to RNA polymerase and influence numerous metabolic reactions, thereby possibly helping bacteria to survive cold periods. Regulation of the GTP pool in the cell is one outcome of the (p)ppGpp availability (Gaca et al., 2015).

### Translational Machinery Assembly and Functioning at Low Temperature

Low temperature causes instability in bacterial 70S ribosomal subunits (Bayles et al., 2000). The 70S ribosome is composed of 30S and 50S subunits. Our expression data show that assemblies of ribosomal subunits are prioritized in the cold-treated samples. The GTPase Era (+1.23 fold; *p* = 2.9 × 10^–3^), the maturation factor RimM (+1.33 fold; *p* = 0.01), and the maturation factor RimP (+5.5 fold; *p* = 0.003) involved in 30S subunit assembly were up-regulated. Similarly, the GTPase Der is a 50S ribosomal subunit-associated factor that helps in maturing and stabilizing the large subunit (Hwang and Inouye, 2006). Cold-induced samples contained a moderately over-represented Der protein signal (+0.9 fold; *p* = 8.4 × 10^–5^). The ATP-dependent RNA helicase RhlE was also enriched (+3.5 fold; *p* = 1.3 × 10^–5^) and may contribute toward 50S subunit assembly by catalyzing unwinding of RNA under cold induction (Owttrim, 2013). Thus, RhlE overexpression indicates a promotion of the cold-specific ribosomal assembly pathway (Owttrim, 2013). Higher expression of the *sua5* gene (+1.3 fold; *p* = 0.03) at cold temperature indicates threonylcarbamoyl group modification on adenine of tRNA to recognize ANN codons. Such modification helps accurate translation by stabilizing the tRNA codon interaction with the ribosome (El Yacoubi et al., 2009).

### Storage Reserves and Metabolic Adaptation

The lower detection of transcript and proteome signals of the respiratory chain and Krebs cycle process at 4°C indicates a slowing down of key metabolic processes (Supplementary Table S4). This is apparently crucial for cost-effectiveness of the operating cellular machinery during cold periods. Upregulation of the amino acid ABC transporter subunit gene (+3.69 fold; *p* = 0.03) underlines the importance of amino acid utilization for protein biosynthesis or in replenishing TCA cycle intermediates. Moreover, moderate upregulation of cyanophycin synthetase (+1.30 fold; *p* = 0.04) also suggests that storage of polypeptides in the form of cyanophycin is an important step to ensure sufficient nutrients for the winter period. Similarly, carbon and energy storage biopolymers such as polyhydroxyalkanoate (PHA) and the associated stabilizer phasin protein are moderately stimulated at colder temperature, i.e., poly(R)-hydroxyalkanoic acid synthase (+1.34 fold; *p* = 0.00002), phasin (+2.64 fold; *p* = 0.01). PHA biosynthesis is known to increase fitness in the Antarctic bacterium *Pseudomonas* sp. 14-3 during cold shock (Ayub et al., 2009). Polymeric PHA is actually an esterified (hydroxy-) fatty acid that represents a lipophilic carbon storage compound. Accumulating PHA might be advantageous during nutrient-scarce long winter scenarios until sufficient nutrients again become available. Interestingly, we detected several lipases and esterases in the cold-treated bacterial samples. The signal peptide-bearing lipase (Pnuc_1435) and carboxylesterase (Pnuc_0443) were overexpressed: +6.0 fold; *p* = 7.6 × 10^–5^ and +7.56 fold; *p* = 8 × 10^–6^, respectively. Intracellular predicted esterases (Pnuc_0531, Pnuc_0959) were also overexpressed: +1.21 fold; *p* = 0.002 and +0.78 fold; *p* = 0.04, respectively. Lipases and esterases can directly modulate the turnover of lipids and long/short chain triacylglycerols (Röttiga and Steinbüchel, 2013) and possibly participate in membrane recycling.

### UV-Irradiated and 26°C-Incubated Sample

Stress-inducing ultraviolet doses prompt bacterial cells to assume a defensive stance, and survival becomes the priority. Cells harvested from irradiated bacteria grown at 26°C^∗^ showed an upregulation of only 4% of the total genes (Figure 2A, Supplementary Figure S2, and Supplementary Tables S2c,d). UV-irradiated bacterial cells displayed over-expression of repair- and defense-related genes, a feature often reported since the 1950s (Beukers et al., 2008). We therefore outlined important features and expression profiles that may help strain QLW-P1DMWA-1^T^ cope with UV radiation.

### Effect of Radiation on Nucleotides and Transcription

UV radiation causes pyrimidine dimer photoproducts on the DNA strand that could prematurely terminate transcription (Michalke and Bremer, 1969). In this treatment, the *xthA* gene encoding for exodeoxyribonuclease III for base excision repair was up-regulated +1.95 fold (*p* = 0.0008). The upregulation of the *pyrH* gene (+2.55 fold; *p* = 0.04) encoding for uridylate kinase points to an enhanced supply of pyrimidine bases (thymine and cytosine) because this gene participates in *de novo* pyrimidine biosynthesis (Bucurenci et al., 1998).

Beyond *de novo* nucleotide biosynthesis, RNAse G overexpression (+1.58 fold, *p* = 0.001) suggests RNA processing and degradation of transcripts due to its endoribonuclease activity toward the 5′ end of rRNA and mRNA, respectively (Aït-Bara and Carpousis, 2015). Similarly, RhlE RNA helicase has been reported to facilitate RNA degradation by PNPase, i.e., polynucleotide phosphorylase (Khemici et al., 2004). Both genes (Pnuc_1342 and Pnuc_1222) were up-regulated (+2.19 fold; *p* = 0.0003, +3.84 fold; *p* = 1.78934 × 10^–5^, respectively), suggesting nucleotide recycling by complementing RNAse G function. Furthermore, HrpA RNA helicase was down-regulated (−4.99 fold; *p* = 4.45 × 10^–6^), which could otherwise enhance transcript stability (Salman-Dilgimen et al., 2013). If the RNA polymerase is blocked during the transcription process, it may be rescued by UvrD helicase (+1.29 fold; *p* = 0.001), which can backtrack the polymerase enzyme (Epshtein, 2015).

### Defense Against Oxidative Stress

Cell protection is apparently a high priority for UV-treated cells because periplasmic catalase and cytosolic rubrerythrin were significantly enriched (+5.25 fold; *p* = 1.7 × 10^–6^ and +6.52 fold; *p* = 0.008, respectively) (Table 1 and Supplementary Table S3). The upregulation of *sod*, encoding for the antioxidant superoxide dismutase, also signifies the superoxide-combating capability of a cell; such superoxide compounds could otherwise damage DNA bases and iron–sulfur clusters in proteins (Keyer and Imlay, 1996). The engagement of cells in guarding the iron–sulfur cluster under radiation has been described for *Deinococcus radiodurans* (Slade and Radman, 2011). Additionally, three PFAM predicted Ahp (alkyl hydroperoxide reductase) enzymes were detected that were mildly up-regulated, i.e., Pnuc_0173, Pnuc_0429, and Pnuc_1534 (+1.34; *p* = 0.003, +0.65; *p* = 0.019, +0.77; *p* = 0.0002, respectively). Multiple hydrogen peroxide- (H_2_O_2_) scavenging enzymes are not uncommon in bacteria (Mishra and Imlay, 2012). Based on that review, we found eleven H_2_O_2_-scavenging enzyme-encoding genes in the genome of the present bacterium (Pnuc_0173, Pnuc_0212, Pnuc_0429, Pnuc_1344, Pnuc_1376, Pnuc_1512, Pnuc_1534, Pnuc_1930, Pnuc_1970, Pnuc_2044, Pnuc_2053). Interestingly, the proteins expressed by six of these genes contain predicted signal peptide sequences, suggesting their active role in periplasmic space. Hydrogen peroxide can originate intracellularly or exogenously (Mishra and Imlay, 2012). Moreover, humic matter and chromophoric dissolved organic substances are important sources of H_2_O_2_, which is produced photochemically (Glaeser et al., 2010; Zhang et al., 2012). This makes it very relevant for this strain to maintain several anti-oxidative enzymes because the natural, humic-matter-rich habitat is shallow and strongly exposed to sunlight in summer (Hahn et al., 2012). Other than catalase (+5.25 fold; *p* = 1.76 × 10^–6^), we also detected glutathione-*S*-transferase (+3.40 fold; *p* = 0.005), which could protect the cell under a UV-induced oxidative damage scenario (Rastogi et al., 2014).

Oxidative stress can seriously impair bacterial cells at the macromolecular level, e.g., oxidative species can directly target the bacterial translation machinery (Katz and Orellana, 2012). Another feature we observed was the reassessment of correct protein synthesis at the level of translation (Supplementary Table S3). Slight enrichment of tmRNA (+0.6 fold; *p* = 0.03) indicates the importance of recycling stalled ribosomes that are stuck during incomplete protein biosynthesis and that have attached a tag on incomplete nascent polypeptides for further proteolysis (Janssen and Hayes, 2012).

We recorded another up-regulated candidate (Pnuc_0887, tRNA 5-hydroxyuridine modification protein YegQ, +1.1 fold; *p* = 0.003) for efficient translation in the UV-treated sample. YegQ is a peptidase U32 family protein involved in modification (hydroxylation) of uridine nucleoside in tRNA (Lauhon, 2019). Base pairing between the mRNA codon and tRNA anticodon is flexible at the third codon base position, which is known as wobble base pairing (Crick, 1966). Moreover, post-transcriptional modification of tRNA’s anticodon loop nucleotide allows expanded base-pairing possibilities (Agris, 1991). Hence, we expect reading and expanded decoding of degenerate mRNA codons by modified tRNAs when the cells are under defensive mode.

In the UV irradiation experiment, we detected slight upregulation of polyphosphate kinase 2 (Pnuc_1582, +1.11 fold; *p* = 0.0002), which utilizes polyP to produce GTP and could also inversely synthesize polyP chains by using either GTP or ATP (Achbergerová and Nahálka, 2011). Poly P accumulation in the cell is associated with stress endurance, and *ppk* (polyphosphate kinase) gene disruption is responsible for UV sensitivity in *Pseudomonas putida* KT2440 (Nikel et al., 2013). Poly P could also act as a chaperone by avoiding damaged protein aggregation such as heat-shock chaperones (Gray et al., 2014). *dnaK* encoding for the Hsp70 molecular chaperone and *groE* encoding for the Hsp60 GroEL chaperone were also up-regulated (+2.1 fold; *p* = 0.006 and +2.4 fold; *p* = 0.03, respectively). This affirms the smooth operation of protein-folding processes when the translation has been successfully completed (Castanié-Cornet et al., 2014). Poly P forms a complex with the ATP-dependent protease Lon, which could eventually degrade ribosomal proteins (Kuroda et al., 2001). Incidentally, we detected S16: Lon domain peptidase (+2.22 fold; *p* = 1.7 × 10^–5^), which is known to increase tolerance for UV (Tsilibaris et al., 2006). Combined, these processes will ultimately make free amino acids available for immediate use in the cell. Complimentarily, the signal-peptide-containing dipeptidyl aminopeptidase (Pnuc_1435) is +6.24 fold (*p* = 5.64 × 10^–7^) was up-regulated, apparently in an effort to obtain extracellularly available amino acids instead of biosynthesizing them.

### Cell Structure Maintenance

UV radiation may cause bacterial cell wall damage and cytoplasmic leakage (Krishnamurthy et al., 2008). Furthermore, Jagger (1983) proposed that the cell wall may deflect near-UV photons, reducing the overall dosage received by the cellular components. Moderate increase of peptidoglycan-related protein signals may indicate protective measures adapted by irradiated bacterial cells [UDP-MurNAc-tripeptide synthetase (+1.71 fold; *p* = 2.4 × 10^–5^), D-alanylalanine synthetase (+1.38 fold; *p* = 1.9 × 10^–3^), murein peptide ligase (+0.41 fold; *p* = 9 × 10^–3^), UDP-*N*-acetylmuramate dehydrogenase (+3.47 fold; *p* = 0.2), anhydro-*N*-acetylmuramic acid kinase (+0.5 fold; *p* = 5.8 × 10^–3^), D-alanyl-D-alanine carboxypeptidase/D-alanyl-D-alanine-endopeptidase (+4.7 fold; *p* = 1.8 × 10^–4^)].

### Metabolic Adaptation of the Cell

We also observed a lower abundance of the pyruvate dehydrogenase subunit gene and citrate synthase gene, which may hinder reactions running toward the citric acid cycle. Moreover, a pirin interaction with pyruvate dehydrogenase inhibits conversion of pyruvate into acetyl-coA. Enrichment of the pirin-encoding gene (+3.4 fold; *p* = 0.03) suggests that glycolytic pathway may not proceed into the citric acid cycle because pirin interacts with pyruvate dehydrogenase and inhibits its activity. Krebs cycle-related enzymes were poorly expressed.

The operation of the glyoxylate shunt under radiation toxicity has been reviewed elsewhere for *Deinococcus radiodurans* (Slade and Radman, 2011). This pathway required a closer examination in our irradiated samples. The glyoxylate shunt is a two-step bypass pathway in which decarboxylation of the Krebs cycle is avoided by the conversion of isocitrate into glyoxylate (with succinate) and further into malate (Dolan and Welch, 2018). Both steps are catalyzed by isocitrate lyase and malate synthase, respectively. In our case, however, the *iclR* (isocitrate lyase repressor) gene was slightly down-regulated (−0.50 fold; *p* = 0.04), which may explain the higher expression of isocitrate lyase (+1.25 fold; *p* = 0002) for triggering the glyoxylate shunt under the radiation-based stress scenario. Interestingly, the transcript of *glcB* (Pnuc_1280, malate synthase G) is overexpressed under UV treatment (+1.75 fold; *p* = 0.04). The corresponding enzyme abundance, however, was under-represented (−0.23 fold; *p* = 0.05). This raised a dilemma as to whether the processing of glyoxylate is low in the cell via the glyoxylate shunt and, if yes, what is the fate of non-processed glyoxylate? We did detect alanine dehydrogenase (Pnuc_0666) (+1.54 fold; *p* = 1.8 × 10^–5^), which can possibly catalyze glyoxylate amination to glycine (other than conversion of pyruvate to L-alanine) (Giffin et al., 2012). Alternatively, glyoxylate also behaves as a chromophore that can absorb light under 400 nm (Bersenkowitsch et al., 2018). Therefore, we cannot rule out a protective role of glyoxylate against UV radiation. Furthermore, Eugene et al. (2016) showed experimentally that glyoxylate could be photolyzed into glyoxal and that a total of 8 h of irradiation can lead to the formation of low molecular weight photoproducts, e.g., formic acid, oxalic acid, tartaric acid, and carbon dioxide. This potential should be experimentally tested to verify a protective and nutritional role of glyoxylate under UV radiation to the stressed cells.

Most cells harvested at 26°C appeared to be dividing because DNA replication, segregation, and cell division genes were positively expressed (Figure 2B and Supplementary Table S5). Upregulation of TRAP dicarboxylate transporter subunits of DctP and TAXI families (Pnuc_0468, Pnuc_0528, Pnuc_0628, Pnuc_1105, Pnuc_1145, Pnuc_1509, Pnuc_1539) at warmer temperature supports the previous interpretation by Hahn et al. (2012) that this strain mainly utilizes low-molecular-weight photooxidation products of humic substances in the lake.

All pathways are interpreted based upon our experiments tabulated in Supplementary Tables S2, S3. Importantly, we measured only transcript and protein changes, which provide an indication of the possible functions being carried out in the cells under varying stress scenarios. This leaves future scope for further experimental validation of our observations.

### Methylation Features in the Bacterial Genome Under Different Treatments

**FIGURE 3:**
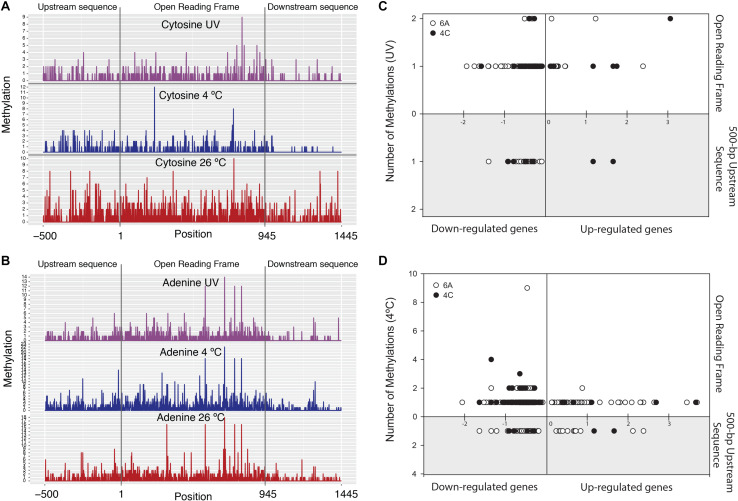
Left panel plots show number of methylations on: **(A)** cytosine and **(B)** adenine nucleotides belonging to average length of all genes in the genome (with averaged 500-bp up- and downstream sequences) in three different treatments. Right panel plots show total number of methylations coinciding with transcriptional profiles in: **(C)** UV-irradiated sample and **(D)** 4°C-incubated sample. *X*-axis: up-regulated genes (positive) and down-regulated genes (negative). *Y*-axis: number of adenine (o) and cytosine (•) methylations. Upper panel *X*-axis: methylation count within the genes. Lower, gray-shaded panel: number of methylations in 500-bp upstream region of genes.

We found that ∼21%, ∼25%, and ∼16% of the strain’s genes were methylated at 4°C, 26°C, and 26°C^∗^ conditions, respectively (Figure 2C and Supplementary Table S6). Methylation in the prokaryotic world is widespread and participates in phage defense as well as epigenetic gene regulation (Adhikari and Curtis, 2016). In fact, the steps involved in the bacterial gene expression process (until protein biosynthesis) include various levels of regulation: DNA supercoiling, *cis* elements and *trans* regulatory factors, DNA methylation, post-transcriptional regulatory mechanisms, mRNA conformation and modification, and post-translational modifications (Willenbrock and Ussery, 2004; Casadesus and Low, 2006; Richards et al., 2008; Balleza et al., 2009; Geissmann et al., 2009; Grangeasse et al., 2015). This section covers only potential methylation-associated gene expression in the strain QLW-P1DMWA-1^T^. Nearly half of the detected methylations (on adenine and cytosine together) in our three treatments was found inside the gene body and their 500-bp upstream region, i.e., 49%, 52%, and 47% in 4°C-, 26°C-, and 26°C^∗^-incubated samples, respectively (Figures 3A,B). The remaining half of the methylations was detected in other intergenic regions of the genome. The presence of methylation at sites other than the gene body and promoter elements is not uncommon (Suzuki and Bird, 2008). For example, intergenic methylation has been cited in the termination of transcription by limiting any aberrant read-through in *Arabidopsis thaliana* (Yan et al., 2016). Moreover, the role of such broadly distributed base modifications no doubt goes beyond gene expression regulation to include phage defense. It may also help avoid unintended transcription initiation or even accidental elongation processes at non-specific sites. The promoter region is known to contribute < 2% of a bacterial genome, and RNA polymerase may therefore have to conduct random walk along DNA strands or three-dimensional diffusion in order to form promoter-bound complexes (Wang et al., 2013). Accordingly, methylation within the coding region could be an additional prevention mechanism in the event of leaky gene expression due to aberrant transcription initiation of supposedly turned-off genes.

**FIGURE 4:**
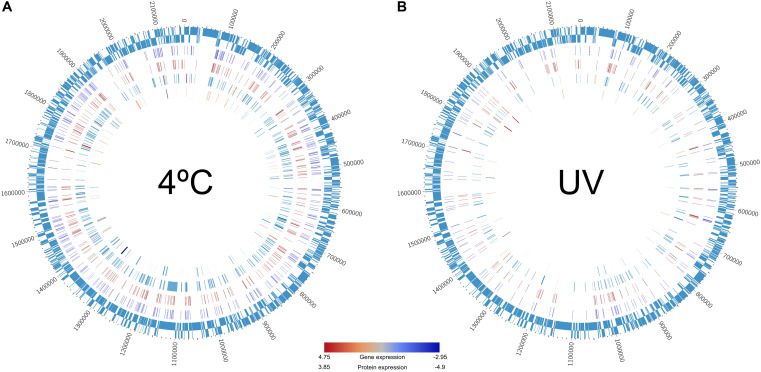
Circos plots showing the direct link between methylome (IPD ratio), transcriptome, and proteome data recorded in the *Polynucleobacter asymbioticus* strain QLW-P1DMWA-1^T^. The remaining data have been omitted to highlight only one-to-one relationships across the three levels. Genes painted in the plots are enlisted in Supplementary Table S8. Outermost ring: the 2.15-Mb genome of this strain. Second internal ring: methylation pattern recorded at **(A)** 4°C and **(B)** 26°C*-incubation. Third ring: methylation recorded at 26°C-incubation. Here, methylations only within the open reading frame and 500-bp upstream of the genes are shown. Fourth ring: the transcribed genes in **(A)** 4°C and **(B)** 26°C*-incubation. Fifth internal ring: protein expression profile in **(A)** 4°C and **(B)** 26°C*-incubation. Color gradient bar: red = higher expression, blue = lower expression at transcriptome and proteome levels.

**FIGURE 5:**
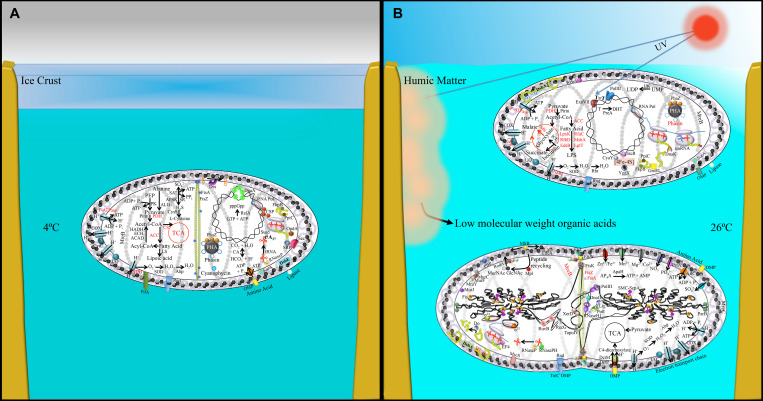
Pictorial representation of transcriptomic and proteomic data obtained from **(A)** 4°C-incubated bacterial sample and **(B)** UV-irradiated (upper cell), 26°C-incubated bacterial sample (lower dividing cell). This figure is a lifestyle inference and shows possible physiological changes together with the involved genetics in *P. asymbioticus* strain QLW-P1DMWA-1^T^ during winter and summer. Black font: up-regulated genes/protein; red font: down-regulated genes/proteins. Abbreviations explained in Supplementary Table S5. Model of humic matter photolysis into low-molecular-weight photo-oxidation products and its utilization by these bacteria has been modified after Hahn et al. (2012).

In our study, ∼13% and ∼6% of the genes were down-regulated when adenine (m6A) and cytosine (m4C) sites were methylated within genes or on +500-bp upstream gene sequences in the 4°C and 26°C^∗^ cultures (considering the 26°C treatment as control), respectively. Very small sets of genes (∼3% and 1% in 4°C and 26°C^∗^ cultures, respectively) were also up-regulated under methylation conditions (Figures 3C,D, Supplementary Figures S3a,b, and Supplementary Tables S7a,b). Clearly, the accepted paradigm that methylation represses genes does not hold true in some cases. For example, genes are generally repressed (when the gene-body or its promoter is methylated), but methylation may also be involved in gene activation as described in *Escherichia coli*, *Salmonella enterica*, and *Caulobacter crescentus* (Braun and Wright, 1986; Balbontin et al., 2006; Fioravanti et al., 2013). Most of the gene expressions associated with methylation states were subtle (i.e., lower expression level), an observation noted by Adhikari and Curtis (2016) in a review article covering many dam (DNA adenine methyltransferase) mutant studies in bacteria. Lastly, in our study, the expression link between methylation state, transcription, and proteome status suggested that only ∼5% and ∼1.7% of the genes were differentially expressed (in the 4°C and 26°C^∗^ cultures, respectively) and could be traced at all three levels (Figure 4 and Supplementary Table S8). Therefore, summary figure (Figure 5) describing major metabolic pathways are broadly based upon transcript and protein expression profiles, and with few methylation-associated expressed genes (Supplementary Tables S5, S8). The fact that we found a trend within our small set of genes that were detected at the three levels indicates that methylation (mainly adenine modification) at low temperature is associated with down-regulated genes (78%) (Table 2A). In contrast, the majority of down-regulated genes (83%) under UV radiation treatment had less association with adenine and cytosine methylation when compared to the 26°C treatment (Table 2A). We examined gene expression because it can be directly affected by methylation, whereas the protein profile might be influenced by post-transcriptional and translational regulations.

**TABLE 2:** Table showing relative variation in the transcriptome, proteome, and in the adenine and cytosine methylation counts as compared to the control (26°C incubation); **(B)** Tabulation of adenine and cytosine methylation counts in the genome (as percentage) in three treatments (i.e., warm, cold, and UV irradiation, i.e., 26°C*); **(C)** adenine and cytosine methylation counts as percentage but on sense and antisense strands in three treatments.

(A)								
Treatments	Transcriptome		Proteome		Methylation			
	
	4°C	UV	4°C	UV	4°C		UV	
					m6A	m4C	m6A	m4C
**Up-regulated**	0.22	0.17	0.39	0.52	0.40	0.68	0.63	0.68
**Down-regulated**	0.78	0.83	0.61	0.48	0.60	0.32	0.37	0.31

**(B)**								

			**Sample (treatments)**					
			**26°C**		**4°C**		**UV**	
	
**Total methylation (%)**	m6A		0.057		0.075		0.042	
	m4C		0.084		0.038		0.038	

**(C)**								

			**Sample (treatments)**					
			**26°C**		**4°C**		**UV**	

**Strand-specific methylation (%)**	m6A	+	0.057		0.078		0.042	
		−	0.057		0.072		0.042	
	m4C	+	0.088		0.038		0.042	
		−	0.081		0.039		0.034	

In the *Polynucleobacter asymbioticus* genome, 0.069% (1504 sites) of the adenine plus cytosine sites were methylated at 26°C, 0.058% (1273 sites) at 4°C, and a mere 0.040% (873 sites) at 26°C^∗^ (Figure 2C and Supplementary Table S6). These values lie in the lower range of recorded methylated bases in tested bacterial genomes: earlier findings revealed a genome methylation range between 0.03% and ∼4% (Fang et al., 2012; Murray et al., 2012; Krebes et al., 2013; Lluch-Senar et al., 2013; Lee et al., 2015; Hiraoka et al., 2019) (Supplementary Table S9). The restriction enzymes database REBASE^®^ lists PacBio-based sequencing information on the genomes of a large number of organisms^5^ and suggests genomes devoid of methylated motifs. This raised the question whether a low methylation level can still participate in gene regulation. Our detailed analysis revealed that the m4C count dropped to about half when the culture was transferred from 26°C to 4°C or exposed to UV-irradiation. In the m6A count, however, we observed ∼25% more modifications when shifting the sample from warmer to cold temperature (4°C) (Figure 2C and Supplementary Table S6). The N6-adenine-specific DNA methylase enzyme (Pnuc_1133) was marginally enriched (+1.15-fold; *p* = 0.0004) at cold incubation, suggesting its role in the DNA methylation process. We detected two ‘16 nucleotides long’ conserved motifs (CT**A**YNNNNNNNNTRTC and GAY**A**NNNNNNNNRTAG) that are complementary to each other (Supplementary Figure S4 and Supplementary Table S6). Bold letter adenine residues in the motifs are modified with a methyl group. We detected 196 motifs of both kinds in the sequenced genome of strain QLW-P1DMWA-1^T^. Only 92% of the motifs were methylated when the bacteria were incubated at 26°C^∗^, but this value approached 97% at 26°C and 4°C. Note that we also observed DNA methylation at sites other than identifiable motifs. The presence of non-specific DNA methyltransferase (for adenine residue modification) in *E. coli* O104:H4 C227-11 might be helpful against phage infection (Murray et al., 2018). Furthermore, we detected only one copy of the type I restriction modification (R-M) system in the genome (Supplementary Figure S5) and two candidate genes (Pnuc_0029, Pnuc_0929) possibly involved in the type III restriction modification process. Many other prokaryotes are known to deploy type I and type III R-M systems to defend their genome against phage infection (Ershova et al., 2015).

At the gene expression level, down-regulated genes encoding for the MscS mechanosensitive ion channel (−1.53 fold; *p* = 0.05) and ComE DNA binding protein (−1.22 fold; *p* = 0.02) had methyl group modifications within the gene body on adenine bases at 4°C, but such a methylation pattern was missing from the samples recovered from the 26°C treatment (Figure 2C and Supplementary Table S6). The methylation status of the *relA* gene is also remarkable considering that this gene is down-regulated in cold treatment: the 26°C sample shows no methylation but becomes methylated (two adenine residues) during 4°C treatment. It is tempting to speculate that the co-occurrence of methylation at 4°C may epigenetically control the expression of the *relA* gene and consequently many other metabolic pathways. Methylation might be involved in the expression of genes, and such observations need to be verified by mutational analysis in the future. Moreover, experiments with methyltransferase such as gene cloning, overexpression, and enzyme purification are required to confirm the actual binding sites on DNA by electrophoretic mobility shift assays. Such experiments are necessary because variation in the DNA polymerase kinetics signal during SMRT sequencing and a weak signal-to-noise ratio (mainly for cytosine modifications) may introduce false positive modifications being overcalled by the PacBio program (Feng et al., 2013).

In terms of base modification symmetry on DNA strands, when the bacterium was shifted from its optimal growth condition (26°C) to either 4°C or 26°C^∗^, the m6A and m4C counts remained at similar ratios between both chromosomal strands. This, however, is not evident when considering only total count (Tables 2B,C). One interpretation is that the bacteria try to maintain a balanced methylation pattern on both strands regardless of experimental conditions. No such observation (and therefore no explanation) has ever been reported before in any system. Nonetheless, human epigenetic studies have demonstrated that a balance between methylation and demethylation process maintains regulatory features. At the same time, over- or under-methylation may cause developmental defects or genetic instability, respectively (Martin and Fry, 2018). This might also hold true in a simpler bacterial model system, and the balance of the methylation pattern at both strands appears to reflect an equilibrium between a cell’s methylating and demethylating enzymatic processes.

### Natural Selection Process

Earlier studies on *Polynucleobacter* bacteria have already revealed their relationship with the environment they inhabit (Hahn et al., 2012, 2015). Our study went one step further by investigating the metabolic and genetic potentials of the strain in question. Moreover, apart from the three-tier study (covering methylation, transcriptomic, and proteomics), we conducted additional analyses on the adaptive process operating due to evolutionary pressure.

**FIGURE 6:**
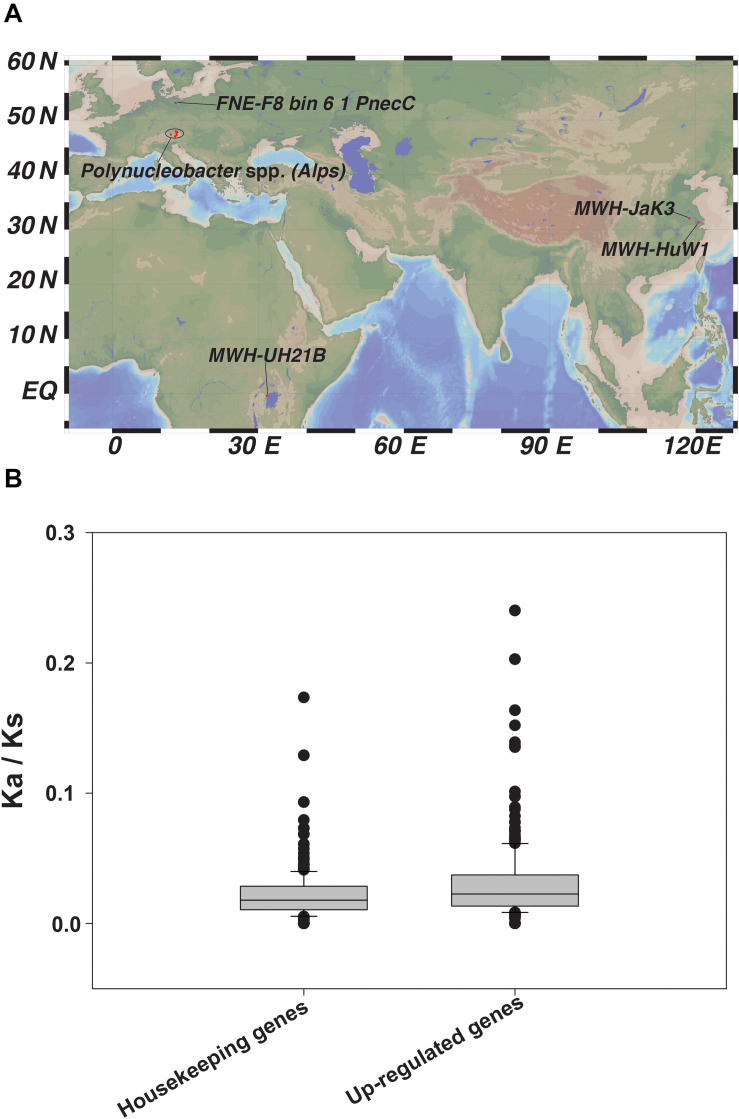
**(A)** Geographical distribution of the 20 *Polynucleobacter* spp. used in our study and **(B)** Ratio of non-synonymous (*K*a) to synonymous (*K*s) nucleotide substitution represented as box-plot for selected housekeeping genes and other selected up-regulated genes chosen from our -omics dataset.

To investigate which selection processes generally operate in this group of bacteria, we expanded our analysis to include twenty other genomes of *Polynucleobacter* spp. (Hoetzinger et al., 2017) (Figure 6A and Supplementary Table S1). To study molecular evolution at the population genetics level, we used a standard method that compares the numbers of non-synonymous and synonymous substitution rates in protein-coding genes (Ina, 1996). Twenty highly expressed genes from the transcriptome or proteome analysis and ten housekeeping genes were selected to estimate the selection imposed upon them. The ratios of non-synonymous to synonymous substitution rates (*K*a/*K*s) revealed that all tested genes underwent purifying selection, i.e., the values were <1 (Figure 6B and Supplementary Tables S10–S12). This indicates that deleterious amino acid replacements have been removed via negative selection (Hurst, 2002). Purifying selection could help explain the moderately streamlined genomes present in all studied *Polynucleobacter* spp. Genome streamlining due to purifying selection followed by ecological niche specialization in the marine free-living picocyanobacteria *Prochlorococcus* have already been reported as important reasons for their global dominance (Sun and Blanchard, 2014). The habitats or previous environments of currently studied freshwater strains and their common ancestors remain undefined. Therefore, the nucleotide sequence divergence that we observe reveals – at best – the operation of purifying selection. However, the signals of any potential positive selection in the past will be masked by the dominance of purifying selection.

Populations of a particular species living in different habitats are naturally selected, leading to local adaptation and genetic divergence (Barton, 2010). Furthermore, strong purifying selection may prevail in species inhabiting diverse geographical locations, as reported for several wild varieties of South American tomatoes (Tellier et al., 2011). Importantly, the genomes of enteric bacteria such as *Escherichia coli* and *Salmonella enterica* that reside in similar habitats exhibit strong positive selection (Chattopadhyay et al., 2012). Exploiting the environmental situations and sustaining large effective population sizes by maintaining the functional sequences in the genome may have promoted adaptive diversification among various *Polynucleobacter* spp. occupying distinct biogeographical regions.

## Conclusion

UV irradiation and temperature were the only distinguishing aspects of our experimental setups. Expectedly, strain QLW-P1DMWA-1^T^ senses thermal disparity in its immediate environment and expresses a multitude of genes accordingly. Small cells with a streamlined genome appear to follow a trend of small-scale changes in gene expression and methylation, significantly contributing to bacterial acclimatization. Bacterial and mammalian epigenetic studies report methylation-induced repression and gene activation (Casadesus and Low, 2006; Udali et al., 2015). Here, we document that methylation patterns in the bacterial genome are constantly revised in response to rapid environmental changes, possibly via *de novo* methylation (Figures 2C, 3A–D and Supplementary Figure S3). This strategy, along with the gene regulatory network, might significantly improve the strain’s survival ability. A direct mechanistic relationship between gene expression and methylation status cannot be determined by conducting mutational analysis in the DNA methylase-encoding gene. Nonetheless, the signals obtained for m6A and m4C were consistent with reports elsewhere (Clark et al., 2012). The simultaneous presence or absence of the methyl group signature patterns of various genes and intergenic regions (Figure 3, Supplementary Figure S3, and Supplementary Table S6) indicates their potential in rapid acclimatization to changing environmental conditions. Moreover, local regulatory factors also contribute in organismal responses to environmental disturbances. We have summarized bacterial acclimatization to changing stressors (Figure 5). This summary includes low-temperature-related stress to the cells, which is well known. However, the increased expression of myriad GTPases suggested regulation of wide-ranging global to local processes. Finally, preserving energy reserves appears to be an important feature for withstanding stressed cold phases. In contrast, UV irradiated cells face oxidative stress and become defensive. Not only do they reduce key energy-generating pathways, but they also focus on protein production for important repair systems. This involves enhanced accuracy at the translational level and expanding mRNA decoding with limited sets of tRNAs.

Our proposal is that overall regulatory changes, together with programming of the DNA methylome in parental cells living in a fluctuating environment, are equally important for daughter cells. This is because the latter cells possibly inherit the exact epigenomic information as an epigenetic memory as proposed in other model system (Gaydos et al., 2014). Although not tested here, this strategy potentially gives bacterial cells a head start in a given natural setting despite undergoing natural selection. In this vein, two recent papers have indicated that *E. coli* daughter cells can be pre-adapted based upon the long-term epigenetic memory they retain upon previous exposure of their parental cells to a particular environment (Ronin et al., 2017; Govers et al., 2018). Determining whether our findings are generally valid across successful species with streamlined genome and smaller cell volumes calls for conducting similar experiments with other environmental bacterial isolates. Some examples to initiate testing would be small-sized prokaryotes with streamlined genomes that are highly abundant in freshwater and marine systems, including more strains of the genus *Polynucleobacter*, acI *Actinobacteria*, and *Prochlorococcus*. Here, epigenetic modifications may play a greater role in cellular functioning than previously recognized.

The strain tested here in the three-tier study (methylome, transcriptome, proteome) is not the complete picture of the success of *Polynucleobacter* spp. in freshwater systems. It does, however, provide insight into various possibilities that might contribute to rapid adjustments in fluctuating environments. Our natural selection pressure study points toward purifying selection in all 20 *P. asymbioticus* strains, and the process is associated with deleterious mutation removal from the genome. Combined, we can conclude that exploiting the environmental situations to maintain effective population size is the best way to compensate for such deleterious mutation load. The extinction episodes of other species are compelling examples of a reduced efficiency of purifying selection that led to the accumulation of detrimental mutations in their genomes. The result was that their populations could not be sustained over the long term and declined (Sánchez-Quinto and Lalueza-Fox, 2015; Rogers and Slatkin, 2017).

We believe that conducting experiments in the natural habitat might disclose more shades of epigenetic and regulatory networks than shown in this report using artificial media and an incubator-based set-up. Moreover, diurnal temperature dynamics are more pronounced in small and shallow ponds. It will be interesting to compare the methylation response of shallow pond-dwellers against the methylome pattern obtained from large and deeper lake inhabitants, i.e., from less dynamic systems. We also foresee that epigenetics studies in aquatic microbial ecology can be used as an indicator of environmental fluctuations reflected in microbial epigenome changes.

## Data Availability Statement

The datasets presented in this study can be found in online repositories. The names of the repository/repositories and accession number(s) can be found in the article/Supplementary Material.

## Author Contributions

AS conceived and designed the details of the project, performed the experiments, analyzed the data, and wrote the manuscript. H-PG designed the overall project, provided mentorship and intellectual input. JM designed the proteomics study, and collected and analyzed data. ME and CW conducted MS analyses. UR contributed with proteomics study designing. JG and YK performed the bioinformatics analyses. DD and MH contributed with statistical analysis. MWH provided the time series data and contributed intellectually. All authors contributed to the article and approved the submitted version.

## Conflict of Interest

YK is employed at GATC Biotech AG. The remaining authors declare that the research was conducted in the absence of any commercial or financial relationships that could be construed as a potential conflict of interest.

## Footnotes

1 https://www.ncbi.nlm.nih.gov/nuccore/CP000655

2 http://www.pacbiodevnet.com/SMRT-Analysis/Algorithms/BLASR

3 http://www.smrtcommunity.com/SMRT-Analysis/Software/SMRT-Analysis

4 http://circos.ca

5 http://rebase.neb.com/cgi-bin/pacbiolist?0
