# Supplementary material for: Combined Methylome, Transcriptome and Proteome Analyses Document Rapid Acclimatization of a Bacterium to Environmental Changes
Source: Front Microbiol. 2020 Sep 15;11:544785. doi: 10.3389/fmicb.2020.544785 (PMC7522526; doi:10.3389/fmicb.2020.544785)
Supplement: Supplementary file 1 [file Table_1.pdf]

Supplementary Table S1.

Strain	Species	Isolation Date	Habitat of Origin	Coordinates of Origin	GenBank/IMG Genome Accession	References
QLW-P1DMWA-1	*Polynucleobacter asymbioticus*	Oct 03	Pond-1	N 47.740° E 13.302°	CP000655	1, 2, 3
P1-4-10KL*	*Polynucleobacter asymbioticus*	Oct 07	Pond-1	N 47.740° E 13.302°	LVJO00000000	2, 3
P1-Kol8	*Polynucleobacter asymbioticus*	Jul-04	Pond-1	N 47.740° E 13.302°	LVLD00000000	2, 3
MWH-Tro7-1-4	*Polynucleobacter asymbioticus*	Oct 07	Pond Trog-7	N 47.250° E 13.267°	LVLF00000000	3
MWH-Tro8-2-9	*Polynucleobacter asymbioticus*	Oct 07	Pond Trog-8	N 47.249° E 13.266°	LVLG00000000	3
Tro8F10W22	*Polynucleobacter asymbioticus*	Aug 14	Pond Trog-8	N 47.249° E 13.266°	CP015018	3
MWH-RechtKolB	*Polynucleobacter asymbioticus*	Oct 06	Pond Rechteckteich	N 47.076° E 12.995°	CP015016	3, 6
MWH-RechtKol4	*Polynucleobacter asymbioticus*	Oct 06	Pond Rechteckteich	N 47.076° E 12.995°	CP015017	3
MWH-Recht1	*Polynucleobacter asymbioticus*	Oct 07	Pond Rechteckteich	N 47.076° E 12.995°	LVLE00000000	3
QLW-P1FAT50C-4	*Polynucleobacter wuianus*	Oct 03	Pond-1	N 47.740° E 13.302°	CP015922	4, 3
QLW-P1DATA-2	*Polynucleobacter* sp.	Oct 03	Pond-1	N 47.740° E 13.302°	LZMQ00000000	3
MWH-Tro8-2-5gr	*Polynucleobacter* sp.	Oct 07	Pond-1	N 47.740° E 13.302°	LZMR00000000	3
MWH-Adler-W8	*Polynucleobacter* sp.	Aug 08	Pond Adlerlacke	N 47.373° E 12.093°	LZFI00000000	3
MWH-JaK3	*Polynucleobacter yangtzensis*	Nov 02	Yangtze River	N 32.114° E 118.732°	LOJI00000000	5
MWH-HuW1	*Polynucleobacter sinensis*	Nov 02	Tiger Hill Pond	N 31.337° 120.576°	LOJJ00000000	5
MWH-MoK4	*Polynucleobacter duraquae*	Sep 02	Lake Mondsee	N 47.830° E 13.376°	CP007501	5
MWH-Weng1-1	*Polynucleobacter sphagniphilus*	Nov 07	Pond Wenger Moor	N 47.928° E 13.176°	MPIY00000000	7
MWH-UH21B	*Polynucleobacter* sp.	Nov 06	Kagona Wetland in Uganda	N -0.343° E 31.875°	not public	
STIR1 (Endosymbiont)	*Polynucleobacter necessarius*	-	Host: *Euplotes aediculatus*	-	CP001010	8
beta proteobacterium CB	*Polynucleobacter* sp.	-	-	-	CP004348	9
FNE-F8 bin 6 1 PnecC	*Polynucleobacter* sp.	-	Lake Grosse Fuchskuhle	N 53.167° E 13.033°	IMG Genome ID: 2596583565	10

*P1-4-10-KL is almost identical to QLW-P1DMWA-1 (22SNPs genome-wide).

References

1. Meincke L, Copeland A, Lapidus A, Lucas S, Berry KW, Del Rio TG, et al. Complete genome sequence of *Polynucleobacter necessarius* subsp. *asymbioticus* type strain (QLW-P1DMWA-1(T)). Stand Genomic Sci. 2012;6:74-83.
2. Hahn MW, Scheuerl T, Jezberová J, Koll U, Jezbera J, Šimek K, et al. The passive yet successful way of planktonic life: genomic and experimental analysis of the ecology of a free-living *Polynucleobacter* population. PLoS One 2012;7:e32772.
3. Hoetzinger, M., Schmidt, J., Jezberová, J., Koll, U. & Hahn, M. W. Microdiversification of a pelagic *Polynucleobacter* species is mainly driven by acquisition of genomic islands from a partially interspecific gene pool. Appl Environ Microbiol. 2017:83:e02266-16.
4. Hahn MW, Huymann LR, Koll U, Schmidt J, Lang E, Hoetzinger M. *Polynucleobacter wuianus* sp. nov., a free-living freshwater bacterium affiliated with the cryptic species complex PnecC. Int J Syst Evol Microbiol. 2017;67:379-385.
5. Hahn, M. W., Schmidt, J., Pitt, A., Taipale, S. J., & Lang, E. Reclassification of four *Polynucleobacter necessarius* strains as *Polynucleobacter asymbioticus comb*. nov., *Polynucleobacter duraquae* sp. nov., *Polynucleobacter yangtzensis* sp. nov., and *Polynucleobacter sinensis* sp. nov., and emended description of the species Polynucleobacter necessarius. Int J Syst Evol Microbiol. 2016b;66:2883-2892.

6. Hahn, M.W., Jezberová, J., Koll, U., Saueressig-Beck, T., & Schmidt, J. Complete ecological isolation and cryptic diversity in Polynucleobacter bacteria not resolved by 16S rRNA gene sequences. ISME J. 2016a;10: 1642-1655.
7. Hahn MW, Karbon G, Koll U, Schmidt J, Lang E. *Polynucleobacter sphagniphilus* sp. nov. a planktonic freshwater bacterium isolated from an acidic and humic freshwater habitat. Int J Syst Evol Microbiol. 2017;67: 3261-3267.
8. Vannini C, Pöckl M, Petroni G, Wu QL, Lang E, Stackebrandt E, *et al*. Endosymbiosis in statu nascendi: close phylogenetic relationship between obligately endosymbiotic and obligately free-living *Polynucleobacter* strains (Betaproteobacteria). Environ. Microbiol. 2007;9:347-359.
9. Hao Z, Li L, Liu J, Ren Y, Wang L, Bartlam M, et al. Genome Sequence of a Freshwater Low-Nucleic-Acid-Content Bacterium, Betaproteobacterium Strain CB. Genome Announc. 2013;1:e00135-00113.
10. Garcia, S.L., McMahon, K.D., Grossart, H-P., Warnecke, F. Successful enrichment of the ubiquitous freshwater acI *Actinobacteria*. Environmental Microbiol Rep. 2014;6:21-27.

Supplementary Table S1. Details belonging to the strains of the genus *Polynucleobacter* used in this study.
