# Supplementary material for: Combined Methylome, Transcriptome and Proteome Analyses Document Rapid Acclimatization of a Bacterium to Environmental Changes
Source: Front Microbiol. 2020 Sep 15;11:544785. doi: 10.3389/fmicb.2020.544785 (PMC7522526; doi:10.3389/fmicb.2020.544785)
Supplement: Supplementary file 4 [file Table_4.pdf]

Supplementary Table S4.

Locus tag	Gene product	Log2 fold changes, 4ºC	p-value, 4ºC	Log2 fold changes, 26ºC*	p-value, 26ºC*
Pnuc_0763	Citrate synthase	-0.3	0.0470334	-0.32	0.0279037
Pnuc_0942	Aconitate hydratase B	0.42	0.00586863	0.22	0.0122051
Pnuc_0366	Isocitrate dehydrogenase [NADP]	0.41	0.000379697	0.39	7.12561E-05
Pnuc_0840	2-oxoglutarate dehydrogenase, E1 subunit	-0.85	0.000238405	-1.11	0.000146018
Pnuc_0841	2-oxoglutarate dehydrogenase complex component E2	-0.84	0.000496529	-0.93	0.00022814
Pnuc_0842	Dihydrolipoyl dehydrogenase	-0.89	0.000745269	-0.97	0.000568912
Pnuc_1830	Succinyl-CoA synthetase subunit alpha	-0.12	0.517745	-0.2	0.202775
Pnuc_1831	Succinyl-CoA synthetase subunit beta	0.069	0.680308	-0.35	0.0459234
Pnuc_0760	Succinate dehydrogenase flavoprotein subunit	-0.32	0.0129625	-0.49	8.07913E-05
Pnuc_0761	Succinate dehydrogenase subunit B	-0.66	0.000023	-0.78	0.0006707
Pnuc_1139	Fumarate hydratase class I	-0.34	0.107649	-0.35	0.0114142
Pnuc_1618	Fumarate hydratase class II	-0.05	0.814663	0.41	0.0339648
Pnuc_1616	Malate:quinone oxidoreductase	-3.05	0.18391	-0.88	0.00848289
Pnuc_0756	Malate dehydrogenase	-0.44	0.00128864	0.0004	0.99599
Pnuc_1043	NADH-quinone oxidoreductase subunit I	-5.38	0.0208763	-2.46	0.000255728
Pnuc_1045	NADH-quinone oxidoreductase subunit G	0.47	0.0121389	0.28	0.014716
Pnuc_1046	NADH-quinone oxidoreductase subunit F	-0.55	0.0268552	-0.75	0.00444586
Pnuc_1048	NADH-quinone oxidoreductase subunit D	-0.56	0.0039125	-0.76	0.000154393
Pnuc_1049	NADH-quinone oxidoreductase subunit C	-0.59	0.00825663	-0.37	0.018118
Pnuc_1050	NADH-quinone oxidoreductase subunit B	-1.85	0.456000	1.02	0.577000
Pnuc_1936	Cytochrome c oxidase, subunit III	-2.42	0.179170	-0.67	0.014248
Pnuc_1939	Cytochrome c oxidase subunit 1	-2.93	0.305045	-0.71	0.000522
Pnuc_1940	Cytochrome c oxidase subunit 2	-0.74	0.016616	-0.84	0.000684
Pnuc_0033	Cytochrome c, class I	-1.00	0.002600	-0.36	0.021062
Pnuc_0022	ATP synthase subunit b	-0.50	0.002802	-0.50	0.002976
Pnuc_0023	ATP synthase subunit delta	-0.73	0.012487	-0.73	0.001111
Pnuc_0024	ATP synthase subunit alpha	-0.40	0.002058	-0.64	0.000566
Pnuc_0025	ATP synthase gamma chain	-0.93	0.018958	-0.85	0.004344
Pnuc_0026	ATP synthase subunit beta	-0.50	0.021835	-0.62	0.000378
Pnuc_0027	ATP synthase epsilon chain	-0.33	0.039365	-0.35	0.007643

Supplementary Table S4. Tabulation of differentially expressed proteins obtained from 4ºC incubated / UV irradiated (26ºC*) grown cells participating in TCA cycle and respiratory chain.

Supplementary Table S5.

UV treated sample (26ºC incubated)

Locus Tag	Abbreviation / Gene product	Function	References
Pnuc_0173	Ahp: Alkyl hydroperoxide reductase (peroxiredoxin)	Cytosolic protein that can sense peroxide and protect other proteins by acting as an anti-oxidant.	Dubbs, J. M., and Mongkolsuk, S. (2007). Peroxiredoxins in bacterial antioxidant defense. *Subcell. Biochem*. 44, 143-193.
Pnuc_0196	Obg	Stringent response GTPase that prevents ribosome assembly by binding to peptidyl transferase center of 50S subunit.	Feng, B., Mandava, C. S., Guo, Q., Wang, J., Cao, W., Li, N., et al. (2014). Structural and functional insights into the mode of action of a universally conserved Obg GTPase. *PLoS Biol*. 12, e1001866.
Pnuc_0254	RfbD: dTDP-4-dehydro rhamnose reductase	Lipopolysaccharide core biosynthesis.	Jofré, E., Lagares, A., and Mori, G. (2004). Disruption of dTDP-rhamnose biosynthesis modifies lipopolysaccharide core, exopolysaccharide production, and root colonization in *Azospirillum brasilense*. *FEMS Microbiol. Lett*. 231, 267-275.
Pnuc_0257	RfaC: Lipopolysaccharide heptosyltransferase I	Participates in outer membrane lipid core oligosaccharide building-up.	Kadrmas, J. L., Raetz, C. R. (1998). Enzymatic properties of lipopolysaccharide in *Escherichia coli*. Purification and properties of heptosyltransferase I. *J. Biol. Chem*. 273, 2799–2807.
Pnuc_0282	KdsB: 3-deoxy-D-manno-octulosonate cytidylyltransferase	Lipopolysaccharide biosynthesis.	Kohlbrenner, W. E., Nuss, M. M., Fesik, S. W. (1987). ^{31}P and ^{13}C NMR studies of oxygen transfer during catalysis by 3-deoxy-D-manno-octulosonate cytidylyltransferase from *Escherichia coli*. *J. Biol. Chem*. 262, 4534-4537.
Pnuc_0284	LpxK: Tetraacyldisaccharide 4'-kinase	Lipopolysaccharide core biosynthesis.	Garrett, T. A., Que, N. L., Raetz, C. R. (1998). Accumulation of a lipid A precursor lacking the 4'-phosphate following inactivation *of the Escherichia coli lpxK* gene. *J. Biol. Chem*. 273, 12457-12465

Pnuc_0379	PpiC: PpiC-type peptidyl-prolyl cis-trans isomerase	Catalyzes protein folding. Mutant carrying defective *ppiC* gene is sensitive to oxidative stress.	Rahfeld, J. U., Schierhorn, A., Mann, K., Fischer, G. (1994). A novel peptidyl-prolyl cis/trans isomerase from *Escherichia coli*. *FEBS Lett*. 343,65-69.
Pnuc_0429	Ahp: Alkyl hydroperoxide reductase (peroxiredoxin)	Cytosolic enzyme that can detoxify various peroxides.	Parsonage, D., Karplus, P. A., Poole, L. B. (2008). Substrate specificity and redox potential of AhpC, a bacterial peroxiredoxin. *Proc. Natl. Acad. Sci. USA*. 105, 8209-8214.
Pnuc_0487	Pirin	Interacts with the pyruvate dehydrogenase and subsequently inhibits its activity.	Soo, P. C., Horng, Y. T., Lai, M. J., Wei, J. R., Hsieh, S. C., Chang, Y. L., et al. (2007). Pirin regulates pyruvate catabolism by interacting with the pyruvate dehydrogenase E1 subunit and modulating pyruvate dehydrogenase activity. *J. Bacteriol*. 189, 109-118.
Pnuc_0610	MsbA: Lipid A ABC exporter	Inner membrane lipid flippase.	Zhang, Y. M., Rock, C. O. (2008). Membrane lipid homeostasis in bacteria. *Nat. Rev. Microbiol*. 6, 222-233.
Pnuc_0611	RNAse G	Endoribonuclease that participates in the maturation of tRNA, processing of rRNA and decay of mRNA.	Bernardini, A., Martínez, J. L. (2017). Genome-wide analysis shows that RNase G plays a global role in the stability of mRNAs in *Stenotrophomonas maltophilia*. *Sci. Rep*. 7, 16016.
Pnuc_0734	PDH: Pyruvate dehydrogenase E1 component	Catalyzes the oxidative decarboxylation of pyruvate to form acetyl-CoA and CO_2.	Patel, M. S., Nemeria, N. S., Furey, W., and Jordan, F. (2014). The pyruvate dehydrogenase complexes: structure-based function and regulation. *J. Biol. Chem*. 289, 16615-16623.
Pnuc_0735	PDH: Pyruvate dehydrogenase E2 component		
Pnuc_0736	PDH: Pyruvate dehydrogenase E3 component		
Pnuc_0740	IclR: Isocitrate lyase repressor	Represses the operon of *aceBAK* by binding directly to the promoter (aceB: malate synthase; aceA: isocitrate lyase monomer; aceK: isocitrate dehydrogenase kinase-phosphatase)	Pan, B., Unnikrishnan, I., LaPorte, D. C. (1996). The binding site of the IclR repressor protein overlaps the promoter of *aceBAK*. *J. Bacteriol*. 178, 3982-3984.

Pnuc_0786<br>Pnuc_0787	CytC: Cytochrome C complex	Heme protein that transfers electron between complex II and complex IV in the electron transport chain. Iron ions are released from CytC during peroxide exposure that causes reactive ferryl-heme species based lipid peroxidation and reactive oxygen species cause DNA damage.	Kim, N. H., and Kang, J. H. (2006). Oxidative damage of DNA induced by the cytochrome C and hydrogen peroxide system. *J. Biochem. Mol. Biol*. 39, 452-456.
Pnuc_0918	LplT: Lysophospholipid transporter	Flippase	Zhang, Y. M., and Rock, C. O. (2008). Membrane lipid homeostasis in bacteria. *Nat. Rev. Microbiol*. 6, 222-233.
Pnuc_0949	Hsp33: 33 kDa heat shock protein	Chaperone function is activated during oxidative stress, senses oxidizing condition e.g. H2O2 and prevents protein aggregation.	Graf, P. C., and Jakob, U. (2002). Redox-regulated molecular chaperones. *Cell Mol. Life Sci.* 59, 1624-1631.
Pnuc_1044	NDH: respiratory-chain NADH dehydrogenase subunit 1	Respiratory chain enzyme that catalyzes the transfer of electron from NADH to coenzyme Q10.	Brandt, U. (2006). Energy converting NADH:quinone oxidoreductase (complex I). *Annu. Rev. Biochem*. 75, 69-92.
Pnuc_1131	AlpA: Phage positive transcriptional regulator	DNA binding regulator that suppresses cellular sensitivity towards UV radiation in lon mutant cells.	Trempy, J. E., Kirby, J. E., and Gottesman, S. (1994). Alp suppression of Lon: dependence on the *slpA* gene. *J. Bacteriol*. 176, 2061-2067.
Pnuc_1263	YggX: Fe(II) trafficking protein	Fe-S cluster protection protein. Mediates the repairing of iron-sulfur clusters effected by oxidative damage.	Velayudhan, J., Karlinsey, J. E., Frawley, E. R., Becker, L. A., Nartea, M., and Fang, F. C. (2014). Distinct roles of the *Salmonella enterica* serovar *Typhimurium* CyaY and YggX proteins in the biosynthesis and repair of iron-sulfur clusters. *Infect. Immun*. 82, 1390-1401.
Pnuc_1280	MS: Malate synthase G	Glyoxylate shunt enzyme that catalyzes the condensation of acetyl-CoA and glyoxylate to yield malate and CoA. Response towards reactive oxygen species.	Slade, D., and Radman, M. (2011). Oxidative stress resistance in *Deinococcus radiodurans*. *Microbiol. Mol. Biol. Rev*. 75, 133-191 .

Pnuc_1376	Rbr: Rubrerythrin	Cytosolic enzyme that is activated during oxidative stress. Converts harmful peroxide into water.	Lehmann, Y., Meile, L., and Teuber, M. (1996). Rubrerythrin from *Clostridium perfringens*: cloning of the gene, purification of the protein, and characterization of its superoxide dismutase function. *J. Bacteriol*. 178, 7152-7158.
Pnuc_1435	DAP: Dipeptidyl aminopeptidase	Extracellular enzyme that catalyse the hydrolysis and sequential release of N-terminal dipeptides of polypeptides.	Gonzales, T., and Robert-Baudouy, J. (1996). Bacterial aminopeptidases: properties and functions. *FEMS Microbiol. Rev*. 18, 319-344.
Pnuc_1449	UK: Uridylate kinase	Phosphorylation of UMP to UDP (that eventually forms thymine and cytosine via pyrimidine biosynthetic pathway).	Serina, L., Blondin, C., Krin E, Sismeiro, O., Danchin, A., Sakamoto, H., et al. (1995). *Escherichia coli* UMP-kinase, a member of the aspartokinase family, is a hexamer regulated by guanine nucleotides and UTP. *Biochemistry*. 34, 5066-5074.
Pnuc_1452	Map: Methionine aminopeptidase	Catalyzes the hydrolysis of N-terminal methionine from newly translated polypeptide chain.	Xiao, Q., Zhang, F., Nacev, B. A., Liu, J. O., and Pei, D. (2010). Protein N-terminal processing: substrate specificity of *Escherichia coli* and human methionine aminopeptidases. *Biochemistry*. 49, 5588-5599.
Pnuc_1489	HscA: Heat shock cognate 66-kDa	Chaperone protein involved in the maturation of Fe-S proteins.	Chandramouli, K., and Johnson, M. K. (2006). HscA and HscB stimulate [2Fe-2S] cluster transfer from IscU to apo-ferredoxin in an ATP-dependent reaction. *Biochemistry*. 45, 11087-11095.
Pnuc_1490	HscB: Heat shock cognate 20-kDa	Co-chaperone protein involved in the maturation of Fe-S proteins.	Chandramouli, K., and Johnson, M. K. (2006). HscA and HscB stimulate [2Fe-2S] cluster transfer from IscU to apo-ferredoxin in an ATP-dependent reaction. *Biochemistry*. 45, 11087-11095.
Pnuc_1494	IscR: Iron-Sulfur cluster regulator	Repressor of *isc* gene cluster (i.e. iron-sulfur biogenesis) and is induced during oxidative stress.	Crack, J. C., Green, J., Thomson, A. J., and Le Brun, N. E. (2012). Iron-sulfur cluster sensor-regulators. *Curr. Opin. Chem. Biol*. 16, 35-44
Pnuc_1506	ICL: Isocitrate lyase	Glyoxylate pathway enzyme. Catalyses cleavage of isocitrate into glyoxylate and succinate	Slade, D., and Radman, M. (2011). Oxidative stress resistance in *Deinococcus radiodurans*. *Microbiol. Mol. Biol. Rev*. 75, 133-191.

		thereby bypassing TCA cycle. Response towards reactive oxygen species.	
Pnuc_1522	WrbA: Tryptophan (W) repressor-binding flavodoxin-like protein	Defense against oxidative stress and has NAD(P)H :quinone oxidoreductases-like activity that allows quinone detoxification.	Patridge, E. V., and Ferry, J. G. (2006). WrbA from *Escherichia coli* and *Archaeoglobus fulgidus* is an NAD(P)H:quinone oxidoreductase. *J. Bacteriol*. 188, 3498-3506.
Pnuc_1534	Ahp: Alkyl hydroperoxide reductase	Cytosolic enzyme that can reduce organic peroxides.	Poole, L. B., and Ellis, H. R. (1996). Flavin-dependent alkyl hydroperoxide reductase from *Salmonella typhimurium*. 1. purification and enzymatic activities of overexpressed AhpF and AhpC proteins. *Biochemistry*. 35, 56-64.
Pnuc_1626	SOD: Superoxide dismutase, copper/zinc binding protein	Periplasmic enzyme (signal peptide predicted). Dismutation of superoxide into hydrogen peroxide and oxygen. Superoxides can otherwise attack DNA bases and Fe-S clusters of various enzymes.	De Groote, M. A., Ochsner, U. A., Shiloh, M. U., Nathan, C., McCord, J. M., Dinauer, M. C., et al. (1997). Periplasmic superoxide dismutase protects *Salmonella* from products of phagocyte NADPH-oxidase and nitric oxide synthase. *Proc. Natl. Acad. Sci. USA*. 94, 13997-14001
Pnuc_1650	S16: lon domain peptidase	Intracellular Lon ATP-dependent protease (related to MEROPS peptidase family S16) that degrades damaged poplypeptides. Lon protease increases tolerance towards UV sensitivity.	Tsilibaris, V., Maenhaut-Michel, G., and Van Melderen, L. (2006). Biological roles of the Lon ATP-dependent protease. *Res. Microbiol*. 157, 701-713.
Pnuc_1715	UvrD: ATP-dependent DNA helicase	Transcription-coupled DNA repair enzyme. Directly rescues the replication fork blocked at transcription level by backtracking RNA polymerase.	Epshtein, V. (2015). UvrD helicase: an old dog with a new trick: how one step backward leads to many steps forward. *Bioessays*. 37, 12-19.
Pnuc_1732	FkpB: Peptidyl-prolyl cis-trans isomerase	Cytosolic protein and has chaperone activity.	Jo, G. A., Lee, J. M., No, G., Kang, D. S., Kim, S. H., Ahn, S. H., et al. (2015). Isolation and characterization of a 17-kDa FKBP-type peptidyl-prolyl cis/trans isomerase from *Vibrio anguillarum*. *Prot. Express Purif*. 110, 130-137.
Pnuc_1769	DnaK: Heat shock protein	Molecular chaperone that binds to the newly	Tomoyasu, T., Ogura, T., Tatsuta, T., and Bukau, B. (1998). Levels

	encoded by *dnaK* locus	synthesized protein and mediates its folding. Unfolded peptides are transferred to GroEL chaperone.	of DnaK and DnaJ provide tight control of heat shock gene expression and protein repair in *Escherichia coli*. *Mol. Microbiol*. 30, 567-581.
Pnuc_1805	GroEL: Large polypeptide chaperone encoded by *groE* locus	Molecular chaperone. Mutation in *E. coli groL* gene makes them susceptible to UV-radiation.	Sargentini, N. J., Gularte, N. P., and Hudman, D. A. (2016). Screen for genes involved in radiation survival of *Escherichia coli* and construction of a reference database. *Mutat Res*. 793-794, 1-14.
Pnuc_1823	Ste24p: MEROPS peptidase family M47	Integral membrane endopeptidase.	Pryor, E. E. Jr., Horanyi, P. S., Clark, K. M., Fedoriw, N., Connelly, S. M., Koszelak-Rosenblum, M., et al. (2013). Structure of the integral membrane protein CAAX protease Ste24p. *Science*. 339, 1600-1604.
Pnuc_1869	SurA: PpiC-type peptidyl-prolyl cis-trans isomerase	Periplasmic chaperone responsible for correct folding of outer membrane proteins.	Xu, X., Wang, S., Hu, Y. X., and McKay, D. B. (2007). The periplasmic bacterial molecular chaperone SurA adapts its structure to bind peptides in different conformations to assert a sequence preference for aromatic residues. *J. Mol. Biol*. 373, 367-381.
Pnuc_2008	ExoIII: Exodeoxyribonuclease III	Involved in base excision repair using endonucleolytic activity at apurinic/ apyrimidinic site. *E. coli* mutant in this gene display sensitivity towards UV treatment.	Sammartano, L. J., Tuveson, R. W. (1983). *Escherichia coli xthA* mutants are sensitive to inactivation by broad-spectrum near-UV (300- to 400-nm) radiation. *J. Bacteriol*. 156, 904-906.
Pnuc_2021	MreB: Rod shape-determining protein	Cytoskeletal protein that determines bacterial cell shape. Active movement of MreB creates fluidity in the cell membrane.	Strahl, H., Bürmann, F., Hamoen, L. W. (2014). The actin homologue MreB organizes the bacterial cell membrane. *Nat. Commun*. 5, 3442
Pnuc_2053	Cat: Catalase	Periplasmic enzyme (signal peptide predicted). Possesses catalase and peroxidase activity. Scavenges harmful peroxide at high concentration and converts it into water and oxygen.	Heimberger, A., Eisenstark, A. (1988). Compartmentalization of catalases in *Escherichia coli*. *Biochem. Biophys. Res. Commun*. 154, 392-397.

Pnuc_R0014	tmRNA: transfer-messenger RNA	Ribonucleic acid with tRNA and mRNA properties. Rescues and recycles stalled translation complexes by tagging incompletely synthesized peptides for eventual proteolysis.	Janssen, B. D., and Hayes, C. S. (2012). The tmRNA ribosome rescue system. *Adv. Protein Chem. Struct. Biol*. 86, 151-191.

4ºC incubated sample

Locus Tag	Abbreviation / Gene product	Function	References
Pnuc_0032	Des: Fatty acid desaturase	Polyunsaturated fatty acid biosynthesis enzyme that introduces double bond in saturated fatty acids.	Mansilla, M. C., Cybulski, L. E., Albanesi, D., and de Mendoza, D. (2004). Control of membrane lipid fluidity by molecular thermosensors. *J. Bacteriol*. 186, 6681-6688.
Pnuc_0132	Phasin	Proteins surrounding PHA granules. Phasin has been shown to be expressed in other cold-adapted bacteria.	Ting, L., Williams, T. J., Cowley, M. J., Lauro, F. M., Guilhaus, M., Raftery, M. J., et al. (2010). Cold adaptation in the marine bacterium, *Sphingopyxis alaskensis*, assessed using quantitative proteomics. *Environ. Microbiol*. 12, 2658-2676.
Pnuc_0173	Ahp: Alkyl hydroperoxide reductase (peroxiredoxin)	Cytosolic protein that can sense peroxide and protect other proteins by acting as an anti-oxidant.	Dubbs, J. M., and Mongkolsuk, S. (2007). Peroxiredoxins in bacterial antioxidant defense. *Subcell. Biochem*. 44, 143-93.
Pnuc_0429	Ahp: Alkyl hydroperoxide reductase (peroxiredoxin)	Cytosolic enzyme that can detoxify various peroxides.	Parsonage, D., Karplus, P. A., and Poole, L. B. (2008). Substrate specificity and redox potential of AhpC, a bacterial peroxiredoxin. *Proc. Natl. Acad. Sci. USA*. 105, 8209-8214.
Pnuc_0586	CSP: Cold-shock DNA-binding protein	Destabilizes secondary structures of RNA during cold-shock and acts as an RNA chaperone.	Jiang, W., Hou, Y., and Inouye, M. (1997). CspA, the major cold-shock protein of *Escherichia coli*, is an RNA chaperone. *J. Biol. Chem*. 272, 196-202.

Pnuc_0611	RNAse G	Endoribonuclease that participates in the maturation of tRNA, processing of rRNA and decay of mRNA.	Ow, M. C., Perwez, T., and Kushner, S. R. (2003). RNase G of *Escherichia coli* exhibits only limited functional overlap with its essential homologue, RNase E. *Mol. Microbiol*. 49, 607-622.
Pnuc_0666	Alanine dehydrogenase	Interconversion of L-alanine and pyruvate by reversible oxidative deamination activity / glyoxylate reductive aminase activity.	Giffin, M. M., Modesti, L., Raab, R. W., Wayne, L. G., and Sohaskey, C. D. (2012). *ald* of *Mycobacterium tuberculosis* encodes both the alanine dehydrogenase and the putative glycine dehydrogenase. *J. Bacteriol*. 194, 1045–1054.
Pnuc_0670	Cyanophycin synthetase	Catalyzes the synthesis of biopolymer reserve multi-L-arginyl-poly-L-aspartic acid, i.e. cyanophycin.	Ziegler, K., Diener, A., Herpin, C., Richter, R., Deutzmann, R., and Lockau, W. (1998). Molecular characterization of cyanophycin synthetase, the enzyme catalyzing the biosynthesis of the cyanobacterial reserve material multi-L-arginyl-poly-L-aspartate (cyanophycin). *Eur. J. Biochem*. 254, 154-159.
Pnuc_0774<br>Pnuc_0913	ACC: Acetyl-coenzyme A carboxylase carboxyl transferase subunit beta<br>ACC: Acetyl-CoA carboxylase, biotin carboxylase	Catalyzes the first step of long-chain fatty acid biosynthesis, i.e. conversion of acetyl-CoA into malonyl-CoA.	Zhang, Y-M., and Rock, C. O. (2008). Membrane lipid homeostasis in bacteria. *Nat. Rev. Microbiol*. 6, 222-233.
Pnuc_1489	HscA: Heat shock cognate 66-kDa	Chaperone protein involved in the maturation of Fe-S proteins. Can be synthesized during cold-shock response.	Lelivelt, M. J., and Kawula, T. H. (1995). Hsc66, an Hsp70 homolog in *Escherichia coli*, is induced by cold shock but not by heat shock. *J. Bacteriol*. 177, 4900-4907.
Pnuc_1490	HscB: Heat shock cognate 20-kDa	Co-chaperone protein involved in the maturation of Fe-S proteins.	Chandramouli, K., and Johnson, M. K. (2006). HscA and HscB stimulate [2Fe-2S] cluster transfer from IscU to apo-ferredoxin in an ATP-dependent reaction. *Biochemistry*. 45, 11087-11095.
Pnuc_1497	PhaZ: Polyhydroxyalkanoate depolymerase (intracellular)	Increased polyhydroxyalkanoate (PHA) degradation for extracting energy and carbon.	Knoll, M., Hamm, T. M., Wagner, F., Martínez, V., and Pleiss, J. (2009). The PHA depolymerase engineering database: a systematic analysis tool for the diverse family of polyhydroxyalkanoate (PHA) depolymerases. *BMC Bioinform*. 10, 89.

Pnuc_1534	Ahp: Alkyl hydroperoxide reductase	Cytosolic enzyme that can reduce organic peroxides.	Poole, L. B., and Ellis, H. R. (1996). Flavin-dependent alkyl hydroperoxide reductase from *Salmonella typhimurium*. 1. purification and enzymatic activities of overexpressed AhpF and AhpC proteins. *Biochemistry*. 35, 56-64.
Pnuc_1626	SOD: Superoxide dismutase, copper/zinc binding protein	Periplasmic enzyme (signal peptide predicted). Dismutation of superoxide into hydrogen peroxide and oxygen. Superoxides can otherwise attack DNA bases and Fe-S clusters of various enzymes.	De Groote, M. A., Ochsner, U. A., Shiloh, M. U., Nathan, C., McCord, J.M., Dinauer, M. C., et al. (1997). Periplasmic superoxide dismutase protects *Salmonella* from products of phagocyte NADPH-oxidase and nitric oxide synthase. *Proc. Natl. Acad. Sci. USA*. 94, 13997-14001.
Pnuc_1732	FkpB: Peptidyl-prolyl cis-trans isomerase	Cytosolic protein and has chaperone activity. Possibly involved in cold adaptation.	Suzuki, Y., Haruki, M., Takano, K., Morikawa, M., and Kanaya, S. (2004). Possible involvement of an FKBP family member protein from a psychrotrophic bacterium *Shewanella* sp. SIB1 in cold-adaptation. *Eur. J. Biochem*. 271, 1372-1381.
Pnuc_1811	Sua5: Translation factor	Required for threonylcarbamoyl group modification on adenine (t6A37) of the tRNA to recognize ANN codons thereby helping in accurate translation by stabilizing tRNA codon interactions with the ribosome.	El Yacoubi, B., Lyons, B., Cruz, Y., Reddy, R., Nordin, B., Agnelli, F., et al. (2009). The universal YrdC/Sua5 family is required for the formation of threonylcarbamoyladenosine in tRNA. *Nucleic Acids Res*. 37, 2894–2909.
Pnuc_1869	SurA: PpiC-type peptidyl-prolyl cis-trans isomerase	Periplasmic chaperone responsible for correct folding of outer membrane proteins.	Xu, X., Wang, S., Hu, Y. X., and McKay, D. B. (2007). The periplasmic bacterial molecular chaperone SurA adapts its structure to bind peptides in different conformations to assert a sequence preference for aromatic residues. *J. Mol. Biol*. 373, 367-381.
Pnuc_1961	LS: Lipoyl synthase	Cofactor lipoic acid biosynthetic enzyme. Lipoic acid has been reported as a key contributor in defending against oxidative stress in bacteria.	Bryk, R., Lima, C. D., Erdjument-Bromage, H., Tempst, P., and Nathan, C. (2002). Metabolic enzymes of mycobacteria linked to antioxidant defense by a thioredoxin-like protein. *Science*. 295, 1073-1077.

Pnuc_2021	MreB: Rod shape-determining protein	Cytoskeletal protein that determines bacterial cell shape. MreB was upregulated during cold-shock treatment of *Vibrio parahaemolyticus*. MreB-ADP bound state may save ATP for cellular activities.	Chiu, S. W., Chen, S. Y., and Wong, H. C. (2008). Localization and expression of MreB in *Vibrio parahaemolyticus* under different stresses. *Appl. Environ. Microbiol*. 74, 7016-7022.
Pnuc_2030	CA: Carbonate dehydratase	This gene encodes for a cytosolic enzyme that catalyzes the reversible hydration of carbon dioxide.	Smith, K. S., and Ferry, J. G. (2000). Prokaryotic carbonic anhydrases. *FEMS Microbiol. Rev*. 24, 335-366.

26ºC incubated sample

Locus Tag	Abbreviation / Gene product	Function	References
Pnuc_0161<br>Pnuc_0163<br>Pnuc_0164<br>Pnuc_0166<br>Pnuc_0168<br>Pnuc_0171<br>Pnuc_0172	FtsI: Peptidoglycan synthetase<br>MurF: UDP-N-acetylmuramoy lalanyl-D-glutamyl-2, 6-diaminopimelate--D-alanyl-D-alanyl ligase<br>MraY: PhosphoN-acetylmuramoyl-pentapeptide-transferase<br>FtsW: Cell cycle protein<br>MurC: UDP-N-acetylmuramate--L-alanine ligase<br>FtsA: Cell division protein<br>FtsZ: Cell division protein	Cell wall biosynthesis and cell division proteins.	Typas, A., Banzhaf, M., Gross, C. A., and Vollmer, W. (2012). From the regulationof peptidoglycan synthesis to bacterial growth and morphology. *Nat. Rev. Microbiol*. 10, 123-136.<br><br>O'Donnell, M., Langston, L., and Stillman, B. (2013). Principles and concepts of DNA replication in bacteria, archaea, and eukarya. *Cold Spring Harb. Perspect. Biol*. 5,a010108.
Pnuc_0173	Ahp: Alkyl hydroperoxide reductase (peroxiredoxin)	Cytosolic protein that can sense peroxide and protect other proteins by acting as an anti-oxidant.	Dubbs, J. M., and Mongkolsuk, S. (2007). Peroxiredoxins in bacterial antioxidant defense. *Subcell. Biochem*. 44, 143-93.

Pnuc_0289	MurB: UDP-N-acetylenolpyruvoyl glucosamine reductase	Cell wall biosynthesis	Typas, A., Banzhaf, M., Gross, C. A., and Vollmer, W. (2012). From the regulationof peptidoglycan synthesis to bacterial growth and morphology. *Nat. Rev. Microbiol*. 10, 123-136.
Pnuc_0292	XerD: Recombinase	Catalyzes recombination event and keeps DNA molecule in monomeric state.	Wang, X., Llopis, P. M., and Rudner, D. Z. (2013). Organization and segregation of bacterial chromosomes. *Nat. Rev. Genet*. 14, 191-203.
Pnuc_0403	Do: Protease	Periplasmic serine protease that degrades cellular proteins and is important in cell survival at elevated temperature.	Seol, J. H., Woo, S. K., Jung, E. M., Yoo, S. J., Lee, C.S., Kim, K. J., et al. (1991). Protease Do is essential for survival of *Escherichia coli* at high temperatures: its identity with the htrA gene product. *Biochem. Biophys. Res. Commun*. 176, 730–736.
Pnuc_0404	EF4: GTP-binding protein LepA	EF4 can back-translocate the ribosome during stalling of protein synthesis under stress scenario.	Starosta, A. L., Lassak, J., Jung, K., and Wilson, D. N. (2014). The bacterial translation stress response. *FEMS Microbiol. Rev*. 38, 1172-1201.
Pnuc_0424	DnaB: Helicase	Catalyzes the opening and propagation of replication fork.	Biswas, E. E., and Biswas, S. B. (1999). Mechanism of DnaB helicase of *Escherichia coli*: structural domains involved in ATP hydrolysis, DNA binding, and oligomerization. *Biochemistry*. 38, 10919-10928.
Pnuc_0429	Ahp: Alkyl hydroperoxide reductase (peroxiredoxin)	Cytosolic enzyme that can detoxify various peroxides.	Parsonage, D., Karplus, P. A., and Poole, L. B. (2008). Substrate specificity and redox potential of AhpC, a bacterial peroxiredoxin. *Proc. Natl. Acad. Sci. USA*. 105, 8209-8214.
Pnuc_0509	MltB: Lytic murein trans-glycosylase B	Cell wall recycling protein.	Suvorov, M., Lee, M., Hesek, D., Boggess, B., and Mobashery, S. (2008). Lytic transglycosylase MltB of *Escherichia coli* and its role in recycling of peptidoglycan strands of bacterial cell wall. *J. Am. Chem Soc*. 130, 11878-11879.
Pnuc_0528	DctP: TRAP dicarboxylate transporter subunit	Tripartite ATP-independent periplasmic carrier that transports C4- dicarboxylates, e.g. malate,	Mulligan, C., Fischer, M., and Thomas, G. H. (2011). Tripartite ATP-independent periplasmic (TRAP) transporters in bacteria

		succinate and fumarate.	and archaea. *FEMS Microbiol. Rev*. 35, 68-86.
Pnuc_0624	PolIII: DNA polymerase III chi subunit, HolC	DNA replication.	Witte, G., Urbanke, C., and Curth, U. (2003). DNA polymerase III chi subunit ties single-stranded DNA binding protein to the bacterial replication machinery. *Nucleic Acids Res*. 31, 4434-4440
Pnuc_0645	ScpA: Chromosome segregation and condensation protein	Interacts with SMC and participates in the partitioning of chromosome during cell division. Mutation in *scpA* gene shows temperature-sensitive growth in *Bacillus subtilis*.	Soppa, J., Kobayashi, K., Noirot-Gros, M. F., Oesterhelt, D., Ehrlich, S. D., Dervyn, E., et al. (2002). Discovery of two novel families of proteins that are proposed to interact with prokaryotic SMC proteins, and characterization of the *Bacillus subtilis* family members ScpA and ScpB. *Mol. Microbiol*. 45, 59-71.
Pnuc_0690	FtsK: DNA translocase	Motor protein located at the cell division septum and moves double strand DNA towards recombination site.	Crozat, E., and Grainge, I. (2010). FtsK DNA translocase: the fast motor that knows where it's going. *Chembiochem*. 11, 2232-2243
Pnuc_0747	DsbA: DSBA oxidoreductase	Periplasmic enzyme that catalyzes the disulfide bond formation and helps in correct protein folding.	Paxman, J. J., Borg, N. A., Horne, J., Thompson, P. E., Chin, Y., Sharma, P., et al. (2009). The structure of the bacterial oxidoreductase enzyme DsbA in complex with a peptide reveals a basis for substrate specificity in the catalytic cycle of DsbA enzymes. *J. Biol. Chem*. 284, 17835-17845.
Pnuc_0900<br>Pnuc_0901	DNA topoisomerase IV subunit B<br>DNA topoisomerase IV subunit A	Disentangles the intertwined DNA after replication process, by creating double-stranded break.	Rawdon, E. J., Dorier, J., Racko, D., Millett, K. C., and Stasiak, A. (2016). How topoisomerase IV can efficiently unknot and decatenate negatively supercoiled DNA molecules without causing their torsional relaxation. *Nucleic Acids Res*. 44, 4528-4538
Pnuc_1064	σ32: RNA polymerase, sigma 32 subunit, RpoH	Sigma subunit of RNA polymerase required for the expression of genes during heat shock response.	Grainger, D. C., and Busby, S. J. (2008). Global regulators of transcription in *Escherichia coli*: mechanisms of action and methods for study. *Adv. Appl. Microbiol*. 65, 93-113.

Pnuc_1080	Ribonuclease PH	Exoribonuclease that participates in the maturation of 3' side of tRNA by removing -CCA sequence.	Kelly, K. O, and Deutscher, M. P. (1992). Characterization of *Eschericia coli* RNase PH. *J. Biol. Chem*. 267, 17153-17158.
Pnuc_1344	AhpC: alkyl hydroperoxide reductase (peroxiredoxin)	Periplasm residing and H2O2 scavenging enzyme.	LeBlanc, J. J., Davidson, R. J., and Hoffman, P. S. (2006). Compensatory functions of two alkyl hydroperoxide reductases in the oxidative defense system of *Legionella pneumophila*. *J. Bacteriol*. 188, 6235-6244.
Pnuc_1376	Rbr: Rubrerythrin	Cytosolic enzyme that is activated during oxidative stress. Converts harmful peroxide into water.	Lehmann, Y., Meile, L., and Teuber, M. (1996). Rubrerythrin from *Clostridium perfringens*: cloning of the gene, purification of the protein, and characterization of its superoxide dismutase function. *J. Bacteriol*. 178, 7152-7158.
Pnuc_1385	SOD: Manganese and iron superoxide dismutase	Cytosolic enzyme that catalyzes the conversion of superoxide radicals into oxygen.	Miller, A-F. (2012). Superoxide dismutases: Ancient enzymes and new insights. *FEBS Lett*. 586, 585-595.
Pnuc_1415	LdcA: Murein tetrapeptidase LD-carboxypeptidase	Peptidoglycan recycling enzyme. Catabolize GlcNAc-MurNAc(anhydro)-tetrapeptide to tripeptide.*	Das, D., Hervé, M., Elsliger, M. A., Kadam, R. U., Grant, J. C., Chiu, H. J., et al. (2013). Structure and function of a novel LD-carboxypeptidase a involved in peptidoglycan recycling. *J. Bacteriol*. 195, 5555-5566.
Pnuc_1457	SMC: Structural maintenance of chromosomes protein	Protein involved in compacting replicated DNA.	Graumann, P. L. (2001). SMC proteins in bacteria: condensation motors for chromosome segregation? *Biochimie*. 83, 53-59.
Pnuc_1534	Ahp: Alkyl hydroperoxide reductase	Cytosolic enzyme that can reduce organic peroxides.	Poole, L. B., and Ellis, H. R. (1996). Flavin-dependent alkyl hydroperoxide reductase from *Salmonella typhimurium*. 1. purification and enzymatic activities of overexpressed AhpF and AhpC proteins. *Biochemistry*. 35, 56-64.
Pnuc_1699	DnaG: DNA primase	Synthesizes the RNA primer that is used by DNA polymerase during replication process.	Rowen, L., and Kornberg, A. (1978). Primase, the *dnaG* protein of *Escherichia coli*. An enzyme which starts DNA chains. *J. Biol. Chem*. 253, 758-764.

Pnuc_1711	PolI: DNA polymerase I	Prokaryotic DNA replication enzyme (polymerase, proofreading and nick translation properties).	Patel, P. H., Suzuki, M., Adman, E., Shinkai, A., and Loeb, L. A. (2001). Prokaryotic DNA polymerase I: evolution, structure, and "base flipping" mechanism for nucleotide selection. *J. Mol. Biol.* 308, 823-837.
Pnuc_1757	MurJ: Lipid II flippase	Peptidoglycan biosynthesis.	Sham, L. T., Butler, E. K., Lebar, M. D., Kahne, D., Bernhardt, T. G., and Ruiz, N. (2014). MurJ is the flippase of lipid-linked precursors for peptidoglycan biogenesis. *Science*. 345, 220-222.
Pnuc_1787	RecG: ATP-dependent DNA helicase	Promotes double-strand break repair and branch migration in homologous recombination event.	Toseland, C. P., Powell, B., and Webb, M. R. (2012). ATPase cycle and DNA unwinding kinetics of RecG helicase. *PLoS One*. 7, e38270.
Pnuc_1880	RuvB: Holliday junction DNA (ruv mutant/recombinant sensitive to UV)	Promotes branch migration of the Holliday junction during genetic recombination event.	Smith, G. R. (1988). Homologous recombination in procaryotes. *Microbiol. Rev*. 52, 1-28.
Pnuc_1929	σ24: RNA polymerase, sigma-24 subunit, RpoE	Sigma subunit of RNA polymerase required for the expression of genes during heat shock response and in response to misfolded proteins.	Grainger, D. C., and Busby, S. J. (2008). Global regulators of transcription in *Escherichia coli*: mechanisms of action and methods for study. *Adv. Appl. Microbiol*. 65, 93-113.
Pnuc_2021	MreB: Rod shape-determining protein		
Pnuc_2022	MreC: Cell shape-determining protein	Cell shape maintenance proteins.	Typas, A., Banzhaf, M., Gross, C. A., and Vollmer, W. (2012). From the regulationof peptidoglycan synthesis to bacterial growth and morphology. *Nat. Rev. Microbiol*. 10, 123-136.
Pnuc_2024	MrdA: Cell elongation-specific peptidoglycan D,D-transpeptidase		
Pnuc_R0042	RNase P	Endonucleolytic ribozyme that participates in the maturation of 5' side of tRNA.	Kirsebom, L. A. (2002). RNase P RNA-mediated catalysis. *Biochem. Soc. Trans*. 30, 1153-1158.

Supplementary Table S5. Tabulation and explanation of abbreviations used in Fig. 5. Transcriptome and proteome data were used to prepare Fig. 5.

Upregulated genes or proteins in black font. Red font depicts downregulated genes or proteins.
*MurNAc: N- acetylmuramic acid, GlcNAc: N-acetylglucosamine.

Supplementary Table S6.

4°C					
Methylation	Location	Strand	Motif	Gene / Intergenic region	Count
m6A	1453	+		Pnuc_0001, chromosomal replication initiator protein DnaA	652(+)
m6A	2464	-		upstream Pnuc_2088, LSU ribosomal protein L34P	621(-)
m6A	7722	+		Pnuc_0008, amino acid/amide ABC transporter substrate-binding protein, HAAT family SIG	**Total** = 1273 (m6A + m4C)
m6A	10210	+		Pnuc_0010, amino acid/amide ABC transporter membrane protein 2, HAAT family	
m4C	10734	+		upstream Pnuc_0014, protein of unknown function DUF81	466(+)
m4C	13299	+		upstream Pnuc_0014, protein of unknown function DUF81	433(-)
m4C	13306	-		Pnuc_0013, acyl-CoA dehydrogenase domain protein	**Total** = 899 (m6A)
m6A	13367	-		Pnuc_0013, acyl-CoA dehydrogenase domain protein	
m6A	13470	-		Pnuc_0013, acyl-CoA dehydrogenase domain protein	186(+)
m6A	14643	+		upstream Pnuc_0015, glucose inhibited division protein A	188(-)
m6A	17573	+		Pnuc_0017, chromosome segregation ATPase	**Total** = 374 (m4C)
m6A	18130	+		Pnuc_0018, chromosome segregation DNA-binding protein	
m4C	18360	-		upstream Pnuc_0013, acyl-CoA dehydrogenase domain protein	Methylated genes = 438 (20.97%)
m4C	18392	+		Pnuc_0018, chromosome segregation DNA-binding protein	
m6A	18941	+		Pnuc_0018, chromosome segregation DNA-binding protein	
m6A	19246	+		Pnuc_0019, putative ATP synthase protein I AtpI	
m6A	20464	-		upstream Pnuc_0013, acyl-CoA dehydrogenase domain protein	
m4C	21005	+		Pnuc_0022, ATP synthase F0 subcomplex B subunit	
m4C	21561	-		upstream Pnuc_0013, acyl-CoA dehydrogenase domain protein	
m4C	23579	+		Pnuc_0025, ATP synthase F1 subcomplex gamma subunit	
m6A	24745	-		upstream Pnuc_0013, acyl-CoA dehydrogenase domain protein	
m6A	28723	+		Pnuc_0029, replication restart DNA helicase PriA	
m4C	28814	+		Pnuc_0029, replication restart DNA helicase PriA	
m4C	28879	+		Pnuc_0029, replication restart DNA helicase PriA	
m4C	32583	-		upstream Pnuc_R0001, tRNA-Arg	
m4C	32592	-		upstream Pnuc_R0001, tRNA-Arg	
m4C	36190	-		Pnuc_0038, GCN5-related N-acetyltransferase	
m6A	36462	+		upstream Pnuc_R0002, 16S ribosomal RNA	
m6A	37807	+	CTAYNNNNNNNNTRTC	Pnuc_R0002, 16S ribosomal RNA	
m6A	37817	-	GAYANNNNNNNNRTAG	upstream Pnuc_0038, GCN5-related N-acetyltransferase	
m4C	38568	-		upstream Pnuc_0038, GCN5-related N-acetyltransferase	
m6A	41947	+		Pnuc_R0006, 5S ribosomal RNA	
m6A	46043	+		upstream Pnuc_0044, LSU ribosomal protein L10P	
m4C	47540	-		upstream Pnuc_0038, GCN5-related N-acetyltransferase	
m4C	48179	-		upstream Pnuc_0038, GCN5-related N-acetyltransferase	
m4C	49107	-		upstream Pnuc_0038, GCN5-related N-acetyltransferase	
m6A	50868	+		Pnuc_0046, DNA-directed RNA polymerase subunit beta	
m6A	52629	-		upstream Pnuc_0038, GCN5-related N-acetyltransferase	
m4C	52724	+		Pnuc_0047, DNA-directed RNA polymerase subunit beta	

m4C	54355	+		Pnuc_0047, DNA-directed RNA polymerase subunit beta		
m6A	54908	+		Pnuc_0047, DNA-directed RNA polymerase subunit beta		
m4C	54915	-		upstream Pnuc_0038, GCN5-related N-acetyltransferase		
m4C	54966	-		upstream Pnuc_0038, GCN5-related N-acetyltransferase		
m6A	55842	+		upstream Pnuc_0048, SSU ribosomal protein S12P		
m6A	56187	+		Pnuc_0048, SSU ribosomal protein S12P		
m6A	57571	+		Pnuc_0050, translation elongation factor 2 (EF-2/EF-G)		
m4C	57861	-		upstream Pnuc_0038, GCN5-related N-acetyltransferase		
m4C	65434	+		Pnuc_0061, LSU ribosomal protein L29P		
m4C	68155	-		upstream Pnuc_0038, GCN5-related N-acetyltransferase		
m6A	68815	+		Pnuc_0069, LSU ribosomal protein L18P		
m4C	71592	-		upstream Pnuc_0038, GCN5-related N-acetyltransferase		
m4C	74844	+		upstream Pnuc_0080, CutA1 divalent ion tolerance protein		
m6A	75131	-		upstream Pnuc_0038, GCN5-related N-acetyltransferase		
m4C	76577	-		upstream Pnuc_0038, GCN5-related N-acetyltransferase		
m4C	77241	-		upstream Pnuc_0038, GCN5-related N-acetyltransferase		
m6A	79279	-		upstream Pnuc_0084, cell division checkpoint GTPase YihA		
m4C	82329	-		Pnuc_0088, 3-dehydroquinate synthase		
m6A	83003	+	GAYANNNNNNNRTAG	upstream Pnuc_0092, penicillin-binding protein, 1A family		
m6A	83013	-	CTAYNNNNNNNTRTC	Pnuc_0088, 3-dehydroquinate synthase		
m6A	84386	+	CTAYNNNNNNNTRTC	upstream Pnuc_0092, penicillin-binding protein, 1A family		
m6A	84396	-	GAYANNNNNNNRTAG	Pnuc_0090, type II and III secretion system protein		
m6A	84996	-		Pnuc_0091, hypothetical protein		
m4C	86659	-		upstream Pnuc_0091, hypothetical protein		
m4C	87028	+		Pnuc_0092, penicillin-binding protein, 1A family		
m4C	87343	+		Pnuc_0092, penicillin-binding protein, 1A family		
m6A	87781	-		upstream Pnuc_0091, hypothetical protein		
m6A	93455	-		upstream Pnuc_0091, hypothetical protein		
m4C	95506	-		upstream Pnuc_0091, hypothetical protein		
m4C	97538	+		Pnuc_0099, VacJ family lipoprotein		
m6A	97671	-		upstream Pnuc_0091, hypothetical protein		
m4C	97977	-		upstream Pnuc_0091, hypothetical protein		
m4C	99225	-		upstream Pnuc_0091, hypothetical protein		
m6A	101158	-		upstream Pnuc_0091, hypothetical protein		
m6A	102779	+	GAYANNNNNNNRTAG	Pnuc_0107, histidinol dehydrogenase		
m6A	102789	-	CTAYNNNNNNNTRTC	upstream Pnuc_0091, hypothetical protein		
m6A	103179	+	CTAYNNNNNNNTRTC	Pnuc_0107, histidinol dehydrogenase		
m6A	103189	-	GAYANNNNNNNRTAG	upstream Pnuc_0091, hypothetical protein		
m6A	106752	+		Pnuc_0111, 1-(5-phosphoribosyl)-5-[(5-phosphoribosylamino)methylideneamino]imidazole-4-carboxamide isomerase		
m6A	108116	-		upstream Pnuc_0108, histidinol phosphate aminotransferase apoenzyme		
m6A	111954	-		Pnuc_0119, pseudo		
m4C	112192	-		Pnuc_0119, pseudo		
m6A	112314	+		upstream Pnuc_0120, porin, Gram-negative type		
m6A	113863	-		upstream Pnuc_0119, pseudo		

m4C	114745	+		Pnuc_0121, TPR repeat-containing protein		
m6A	115995	-		upstream Pnuc_0119, pseudo		
m6A	117881	-		Pnuc_0122, Tetratricopeptide TPR_2 repeat protein		
m4C	117995	-		Pnuc_0122, Tetratricopeptide TPR_2 repeat protein		
m6A	118329	-		Pnuc_0123, peptidase S1 and S6, chymotrypsin/Hap		
m6A	119609	+		upstream Pnuc_0125, ubiquinol-cytochrome c reductase, iron-sulfur subunit		
m4C	119836	+		upstream Pnuc_0125, ubiquinol-cytochrome c reductase, iron-sulfur subunit		
m4C	119892	-		upstream Pnuc_0124, large conductance mechanosensitive channel protein		
m6A	119969	+		Pnuc_0125, ubiquinol-cytochrome c reductase, iron-sulfur subunit		
m6A	122705	+	CTAYNNNNNNNNTRTC	Pnuc_0127, cytochrome c1		
m6A	122715	-	GAYANNNNNNNNRTAG	upstream Pnuc_0124, large conductance mechanosensitive channel protein		
m6A	123799	+		Pnuc_0129, Stringent starvation protein B		
m6A	130121	+	GAYANNNNNNNNRTAG	upstream Pnuc_0142, GTP-binding protein YchF		
m6A	130131	-	CTAYNNNNNNNNTRTC	Pnuc_0136, bacterial peptide chain release factor 1(bRF-1)		
m4C	131167	-		Pnuc_0137, glutamyl-tRNA reductase		
m4C	135156	+		upstream Pnuc_0142, GTP-binding protein YchF		
m6A	145576	-		Pnuc_0152, amidohydrolase		
m6A	145707	+		upstream Pnuc_0156, biotin synthase		
m6A	147876	+		upstream Pnuc_0156, biotin synthase		
m6A	149067	+	CTAYNNNNNNNNTRTC	upstream Pnuc_0156, biotin synthase		
m4C	149837	+		upstream Pnuc_0156, biotin synthase		
m4C	154466	-		upstream Pnuc_0155, aminotransferase		
m6A	157993	+	GAYANNNNNNNNRTAG	Pnuc_0163, UDP-N-acetylmuramoylalanyl-D-glutamyl-2,6-diaminopimelate-D-alanyl-D-alanyl ligase		
m6A	158003	-	CTAYNNNNNNNNTRTC	upstream Pnuc_0155, aminotransferase		
m6A	159967	+		Pnuc_0165, UDP-N-acetylmuramoylalanine--D-glutamate ligase		
m6A	162336	-		upstream Pnuc_0155, aminotransferase		
m4C	164580	-		upstream Pnuc_0155, aminotransferase		
m6A	167650	+		Pnuc_0171, cell division protein FtsA		
m4C	168631	+		Pnuc_0172, cell division protein FtsZ		
m6A	170853	+		Pnuc_0174, UDP-3-O-[3-hydroxymyristoyl] N-acetylglucosamine deacetylase		
m4C	170901	+		Pnuc_0174, UDP-3-O-[3-hydroxymyristoyl] N-acetylglucosamine deacetylase		
m6A	171824	+		Pnuc_0176, protein translocase subunit secA		
m6A	175537	+		Pnuc_0178, aldehyde oxidase and xanthine dehydrogenase,molybdopterin binding protein		
m4C	179581	-		upstream Pnuc_0175, hypothetical protein		
m6A	180449	-		Pnuc_0183, dephospho-CoA kinase		
m4C	180979	+		upstream Pnuc_0188, methylated-DNA--protein-cysteine methyltransferase		
m6A	183387	+		upstream Pnuc_0188, methylated-DNA--protein-cysteine methyltransferase		
m6A	190277	+		upstream Pnuc_0194, LSU ribosomal protein L21P		
m4C	190717	+		upstream Pnuc_0194, LSU ribosomal protein L21P		
m4C	192640	-		upstream Pnuc_0193, Farnesyltranstransferase		
m6A	196749	-		upstream Pnuc_0199, NUDIX hydrolase, downstream Pnuc_0201, signal recognition particle subunit FFH/SRP54 (srp54)		
m6A	196752	+		upstream Pnuc_0202, cytochrome c assembly protein		
m6A	197824	+		upstream Pnuc_0202, cytochrome c assembly protein		
m6A	200762	+	CTAYNNNNNNNNTRTC	Pnuc_0204, ribonucleoside-diphosphate reductase, alpha subunit		

m6A	200772	-	GAYANNNNNNNRTAG	upstream Pnuc_0201, signal recognition particle subunit FFH/SRP54 (srp54)	
m6A	200911	-		upstream Pnuc_0201, signal recognition particle subunit FFH/SRP54 (srp54)	
m6A	201518	-		upstream Pnuc_0201, signal recognition particle subunit FFH/SRP54 (srp54)	
m4C	202691	-		upstream Pnuc_0201, signal recognition particle subunit FFH/SRP54 (srp54)	
m6A	202867	-		upstream Pnuc_0201, signal recognition particle subunit FFH/SRP54 (srp54)	
m6A	204236	+		Pnuc_0205, Ribonucleoside-diphosphate reductase	
m6A	204565	+		upstream Pnuc_0206, hypothetical protein	
m6A	204937	+		Pnuc_0206, hypothetical protein	
m6A	207217	-		Pnuc_0208, [LSU ribosomal protein L11P]-lysine N-methyltransferase	
m6A	209668	+	GAYANNNNNNNRTAG	upstream Pnuc_0213, UDP-N-acetylmuramate	
m6A	209678	-	CTAYNNNNNNNTRTC	Pnuc_0211, 3-dehydroquinate dehydratase	
m6A	210167	+		upstream Pnuc_0213, UDP-N-acetylmuramate	
m6A	210170	+		upstream Pnuc_0213, UDP-N-acetylmuramate	
m4C	210233	-		upstream Pnuc_0211, 3-dehydroquinate dehydratase	
m4C	210234	-		upstream Pnuc_0211, 3-dehydroquinate dehydratase	
m6A	210241	+		upstream Pnuc_0213, UDP-N-acetylmuramate	
m6A	210710	-		Pnuc_0212, alkyl hydroperoxide reductase/ Thiol specific antioxidant/ Mal allergen	
m6A	213423	+		Pnuc_0215, ribonuclease II	
m6A	215142	-		upstream Pnuc_0212, alkyl hydroperoxide reductase/ Thiol specific antioxidant/ Mal allergen	
m6A	215145	-		upstream Pnuc_0212, alkyl hydroperoxide reductase/ Thiol specific antioxidant/ Mal allergen	
m6A	215821	-		upstream Pnuc_0212, alkyl hydroperoxide reductase/ Thiol specific antioxidant/ Mal allergen	
m4C	216905	-		upstream Pnuc_0212, alkyl hydroperoxide reductase/ Thiol specific antioxidant/ Mal allergen	
m4C	218122	+		upstream Pnuc_0220, magnesium and cobalt transport protein CorA	
m6A	220559	+		upstream Pnuc_0224, Malate dehydrogenase (oxaloacetate-decarboxylating) (NADP(+))., Phosphate acetyltransferase	
m6A	220716	-		Pnuc_0223, thiamine-phosphate kinase	
m6A	220989	-		Pnuc_0223, thiamine-phosphate kinase	
m6A	224087	-		upstream Pnuc_0223, thiamine-phosphate kinase	
m6A	227606	+		Pnuc_0228, glyceraldehyde-3-phosphate dehydrogenase	
m6A	227673	+	GAYANNNNNNNRTAG	Pnuc_0228, glyceraldehyde-3-phosphate dehydrogenase	
m6A	227683	-	CTAYNNNNNNNTRTC	upstream Pnuc_0226, protein of unknown function DUF558	
m6A	227895	+		Pnuc_0228, glyceraldehyde-3-phosphate dehydrogenase	
m6A	228520	+		Pnuc_0228, glyceraldehyde-3-phosphate dehydrogenase	
m6A	228630	-		upstream Pnuc_0226, protein of unknown function DUF558	
m4C	228649	-		upstream Pnuc_0226, protein of unknown function DUF558	
m4C	231697	+		Pnuc_0232, leucyl-tRNA synthetase	
m6A	231802	+		Pnuc_0232, leucyl-tRNA synthetase	
m4C	232073	-		upstream Pnuc_0229, ferric uptake regulator, Fur family	
m6A	238728	+		Pnuc_0239, glutamate-1-semialdehyde 2,1-aminomutase	
m4C	238745	-		upstream Pnuc_0229, ferric uptake regulator, Fur family	
m4C	240934	+		upstream Pnuc_0242, protein of unknown function DUF218	
m6A	242771	+		Pnuc_0242, protein of unknown function DUF218	
m4C	245419	-		upstream Pnuc_0241, deoxyribodipyrimidine photo-lyase type I	
m6A	248429	+	CTAYNNNNNNNTRTC	Pnuc_0249, glycosyl transferase, family 25	
m6A	248439	-	GAYANNNNNNNRTAG	upstream Pnuc_0241, deoxyribodipyrimidine photo-lyase type I	

m4C	250123	-		Pnuc_0251, hypothetical protein
m6A	250899	+	CTAYNNNNNNNNTRTC	Pnuc_0252, hypothetical protein
m6A	250909	-	GAYANNNNNNNNRTAG	upstream Pnuc_0251, hypothetical protein
m6A	251656	+	GAYANNNNNNNNRTAG	upstream Pnuc_0253, dTDP-glucose 4,6-dehydratase
m6A	251666	-	CTAYNNNNNNNNTRTC	upstream Pnuc_0251, hypothetical protein
m6A	263341	+		upstream Pnuc_0267, hypothetical protein
m4C	263605	+		upstream Pnuc_0267, hypothetical protein
m4C	265118	-		upstream Pnuc_0266, 3,4-dihydroxy-2-butanone 4-phosphate synthase
m6A	269370	+	GAYANNNNNNNNRTAG	upstream Pnuc_0279, serine hydroxymethyltransferase
m6A	269380	-	CTAYNNNNNNNNTRTC	Pnuc_0272 (start codon), preQ(0) biosynthesis protein QueC
m4C	270928	-		Pnuc_0275, TolB, N-terminal domain protein
m6A	271013	+	GAYANNNNNNNNRTAG	upstream Pnuc_0279, serine hydroxymethyltransferase
m6A	271023	-	CTAYNNNNNNNNTRTC	Pnuc_0275, TolB, N-terminal domain protein
m6A	271901	+		upstream Pnuc_0279, serine hydroxymethyltransferase
m6A	271994	+		upstream Pnuc_0279, serine hydroxymethyltransferase
m6A	272665	-		Pnuc_0276, Cell division and transport-associated protein TolA
m6A	272704	-		Pnuc_0276, Cell division and transport-associated protein TolA
m6A	276373	+		upstream Pnuc_0287, Exodeoxyribonuclease VII large subunit
m6A	277009	+	CTAYNNNNNNNNTRTC	upstream Pnuc_0287, Exodeoxyribonuclease VII large subunit
m6A	277019	-	GAYANNNNNNNNRTAG	Pnuc_0282, 3-deoxy-D-manno-octulosonate cytidylyltransferase
m4C	278708	+		upstream Pnuc_0287, Exodeoxyribonuclease VII large subunit
m6A	283189	+	GAYANNNNNNNNRTAG	upstream Pnuc_0290, protein of unknown function DUF520
m6A	283199	-	CTAYNNNNNNNNTRTC	Pnuc_0289, UDP-N-acetylmuramate dehydrogenase
m6A	287569	+		upstream Pnuc_0296, High potential iron-sulfur protein
m6A	287750	-		upstream Pnuc_0289, UDP-N-acetylmuramate dehydrogenase
m6A	297004	+		Pnuc_0306, glycosyl transferase, family 2
m4C	299064	+		upstream Pnuc_0311, polysaccharide biosynthesis protein
m6A	305645	+		upstream Pnuc_0314, transferase hexapeptide repeat containing protein
m6A	310366	+	CTAYNNNNNNNNTRTC	Pnuc_0318, ABC-2 type transporter
m6A	310376	-	GAYANNNNNNNNRTAG	upstream Pnuc_0316, glycosyl transferase, family 4
m6A	312933	+	GAYANNNNNNNNRTAG	Pnuc_0320, Methyltransferase type 11
m6A	312943	-	CTAYNNNNNNNNTRTC	upstream Pnuc_0316, glycosyl transferase, family 4
m6A	313175	+	GAYANNNNNNNNRTAG	Pnuc_0320, Methyltransferase type 11
m6A	313185	-	CTAYNNNNNNNNTRTC	upstream Pnuc_0316, glycosyl transferase, family 4
m6A	317235	+		Pnuc_0322, hypothetical protein
m6A	318999	+	CTAYNNNNNNNNTRTC	upstream Pnuc_0324, NAD-dependent epimerase/dehydratase
m6A	319009	-	GAYANNNNNNNNRTAG	upstream Pnuc_0316, glycosyl transferase, family 4
m6A	320925	+	GAYANNNNNNNNRTAG	Pnuc_0325, amine oxidase
m6A	320935	-	CTAYNNNNNNNNTRTC	upstream Pnuc_0316, glycosyl transferase, family 4
m6A	321154	+	CTAYNNNNNNNNTRTC	Pnuc_0325, amine oxidase
m6A	321164	-	GAYANNNNNNNNRTAG	upstream Pnuc_0316, glycosyl transferase, family 4
m6A	324292	+	GAYANNNNNNNNRTAG	Pnuc_0329, UbiA prenyltransferase
m6A	324302	-	CTAYNNNNNNNNTRTC	upstream Pnuc_0328, FAD linked oxidase domain protein
m4C	325168	-		upstream Pnuc_0328, FAD linked oxidase domain protein

m6A	331725	+		upstream Pnuc_0337, acyltransferase 3		
m4C	331986	+		upstream Pnuc_0337, acyltransferase 3		
m6A	335858	+	CTAYNNNNNNNNTRTC	Pnuc_0338, hypothetical protein		
m6A	335868	-	GAYANNNNNNNNRTAG	upstream Pnuc_0336, hypothetical protein		
m6A	345459	+	GAYANNNNNNNNRTAG	upstream Pnuc_0351, acyltransferase 3		
m6A	345469	-	CTAYNNNNNNNNTRTC	upstream Pnuc_0349, hypothetical protein		
m6A	347772	+	GAYANNNNNNNNRTAG	Pnuc_0353, Choline dehydrogenase		
m6A	347782	-	CTAYNNNNNNNNTRTC	upstream Pnuc_0352, protein of unknown function DUF615		
m6A	349550	+		Pnuc_0354, protein of unknown function DUF6, transmembrane		
m4C	351661	-		upstream Pnuc_0352, protein of unknown function DUF615		
m6A	372082	+		Pnuc_0379, PpiC-type peptidyl-prolyl cis-trans isomerase		
m6A	374721	+		upstream Pnuc_0384, D-isomer specific 2-hydroxyacid dehydrogenase, NAD-binding protein		
m6A	375651	+		upstream Pnuc_0384, D-isomer specific 2-hydroxyacid dehydrogenase, NAD-binding protein		
m4C	376339	-		upstream Pnuc_0383, 4Fe-4S ferredoxin, iron-sulfur binding domain protein		
m4C	381037	-		Pnuc_0389, RNAse E		
m6A	381129	-		Pnuc_0389, RNAse E		
m6A	382455	-		Pnuc_0389, RNAse E		
m6A	382815	+		upstream Pnuc_0390, ribosomal large subunit pseudouridine synthase C		
m6A	383532	+		Pnuc_0390, ribosomal large subunit pseudouridine synthase C		
m4C	387658	-		upstream Pnuc_0394, maf protein		
m4C	387721	+		Pnuc_0395, protein of unknown function DUF177		
m4C	387959	-		upstream Pnuc_0394, maf protein		
m6A	389530	+		Pnuc_0398, 3-oxoacyl-[acyl-carrier-protein] synthase III		
m6A	389684	+		Pnuc_0398, 3-oxoacyl-[acyl-carrier-protein] synthase III		
m6A	396752	+		Pnuc_0404, GTP-binding protein LepA		
m4C	396765	+		Pnuc_0404, GTP-binding protein LepA		
m4C	396769	+		Pnuc_0404, GTP-binding protein LepA		
m4C	396777	+		Pnuc_0404, GTP-binding protein LepA		
m4C	396778	+		Pnuc_0404, GTP-binding protein LepA		
m6A	397041	+	CTAYNNNNNNNNTRTC	Pnuc_0404, GTP-binding protein LepA		
m6A	397051	-	GAYANNNNNNNNRTAG	upstream Pnuc_0394, maf protein		
m4C	398437	+		Pnuc_0406, RNAse III		
m6A	398446	+		Pnuc_0406, RNAse III		
m6A	401086	+	CTAYNNNNNNNNTRTC	Pnuc_0409, pyridoxine 5'-phosphate synthase		
m6A	401096	-	GAYANNNNNNNNRTAG	upstream Pnuc_0394, maf protein		
m6A	401232	+	GAYANNNNNNNNRTAG	Pnuc_0410, holo-acyl-carrier-protein synthase		
m6A	401242	-	CTAYNNNNNNNNTRTC	upstream Pnuc_0394, maf protein		
m4C	402615	-		upstream Pnuc_0394, maf protein		
m4C	404201	-		upstream Pnuc_0413, translation elongation factor P (EF-P)		
m4C	407370	-		upstream Pnuc_0414, conserved hypothetical protein		
m4C	407402	-		upstream Pnuc_0414, conserved hypothetical protein		
m6A	407449	-		upstream Pnuc_0414, conserved hypothetical protein		
m6A	407712	-		upstream Pnuc_0414, conserved hypothetical protein		
m6A	415368	-		Pnuc_0426, MscS Mechanosensitive ion channel		

m6A	418091	+		upstream Pnuc_0437, aminotransferase		
m6A	420341	+		upstream Pnuc_0437, aminotransferase		
m6A	421455	+	GAYANNNNNNNRTAG	upstream Pnuc_0437, aminotransferase		
m6A	421465	-	CTAYNNNNNNNTRTC	Pnuc_0432, formyl transferase domain protein		
m6A	422836	+	CTAYNNNNNNNTRTC	upstream Pnuc_0437, aminotransferase		
m6A	422846	-	GAYANNNNNNNRTAG	Pnuc_0434, DegT/DnrJ/EryC1/StrS aminotransferase		
m6A	424440	+		upstream Pnuc_0437, aminotransferase		
m6A	428162	-		upstream Pnuc_0436, protein of unknown function DUF498		
m4C	433725	+		Pnuc_0443, Carboxylesterase, type B		
m4C	433790	-		upstream Pnuc_0436, protein of unknown function DUF498		
m4C	436964	+		upstream Pnuc_0447, ATPase AAA-2 domain protein		
m6A	439040	+	GAYANNNNNNNRTAG	Pnuc_0447, ATPase AAA-2 domain protein		
m6A	439050	-	CTAYNNNNNNNTRTC	upstream Pnuc_0446, Excinuclease ABC, C subunit domain protein		
m6A	440012	+	GAYANNNNNNNRTAG	Pnuc_0447, ATPase AAA-2 domain protein		
m6A	440022	-	CTAYNNNNNNNTRTC	upstream Pnuc_0446, Excinuclease ABC, C subunit domain protein		
m6A	442569	+	GAYANNNNNNNRTAG	Pnuc_0450, major facilitator superfamily MFS_1		
m6A	442579	-	CTAYNNNNNNNTRTC	upstream Pnuc_0449, Uncharacterized protein UPF0065		
m4C	442618	-		upstream Pnuc_0449, Uncharacterized protein UPF0065		
m6A	443389	-		upstream Pnuc_0449, Uncharacterized protein UPF0065		
m6A	445114	+		Pnuc_0451, heavy metal translocating P-type ATPase		
m6A	445942	+	CTAYNNNNNNNTRTC	upstream Pnuc_0453, cytochrome c oxidase, cbb3-type, subunit I		
m6A	445952	-	GAYANNNNNNNRTAG	upstream Pnuc_0449, Uncharacterized protein UPF0065		
m6A	445992	-		upstream Pnuc_0449, Uncharacterized protein UPF0065		
m4C	447945	-		upstream Pnuc_0449, Uncharacterized protein UPF0065		
m6A	450432	+		Pnuc_0457, 4Fe-4S ferredoxin, iron-sulfur binding domain protein		
m6A	452613	-		upstream Pnuc_0460, putative transcriptional regulator, Crp/Fnr family		
m6A	453512	+	CTAYNNNNNNNTRTC	Pnuc_0463, UspA domain protein		
m6A	453522	-	GAYANNNNNNNRTAG	upstream Pnuc_0462, conserved hypothetical protein		
m6A	456340	+		upstream Pnuc_0466, Mandelate racemase/muconate lactonizing enzyme, C-terminal domain protein		
m6A	456402	-		upstream Pnuc_0465, 3-hydroxyacyl-CoA dehydrogenase		
m6A	457988	-		Pnuc_0467, TRAP transporter, 4TM/12TM fusion protein		
m6A	460630	-		upstream Pnuc_0468, TRAP transporter solute receptor, TAXI family		
m4C	460892	-		Pnuc_0469, dihydrodipicolinate synthetase		
m6A	461835	+	GAYANNNNNNNRTAG	upstream Pnuc_0471, 3-hydroxyacyl-CoA dehydrogenase		
m6A	461845	-	CTAYNNNNNNNTRTC	Pnuc_0470, D-isomer specific 2-hydroxyacid dehydrogenase, NAD-binding protein		
m6A	462740	-		upstream Pnuc_0470, D-isomer specific 2-hydroxyacid dehydrogenase, NAD-binding protein		
m4C	463424	-		upstream Pnuc_0470, D-isomer specific 2-hydroxyacid dehydrogenase, NAD-binding protein		
m6A	465489	-		Pnuc_0474, cytochrome c, class II		
m4C	466672	+		upstream Pnuc_0476, Extradiol ring-cleavage dioxygenase, class III enzyme, subunit B		
m4C	467171	+		Pnuc_0476, Extradiol ring-cleavage dioxygenase, class III enzyme, subunit B		
m6A	474614	+	CTAYNNNNNNNTRTC	upstream Pnuc_0485, Uncharacterized protein UPF0065		
m6A	474624	-	GAYANNNNNNNRTAG	upstream Pnuc_0484, Rhodanese domain protein		
m4C	478450	+		Pnuc_0489, TPR repeat-containing protein		
m6A	482125	-		Pnuc_0492, OmpA/MotB domain protein		

m4C	483340	-		upstream Pnuc_0492, OmpA/MotB domain protein	
m6A	487192	+		Pnuc_0495, chorismate mutase	
m4C	487707	-		upstream Pnuc_0492, OmpA/MotB domain protein	
m4C	488286	-		upstream Pnuc_0492, OmpA/MotB domain protein	
m4C	489439	-		upstream Pnuc_0492, OmpA/MotB domain protein	
m6A	493196	+		Pnuc_0500, SSU ribosomal protein S1P	
m6A	495425	-		upstream Pnuc_0492, OmpA/MotB domain protein	
m6A	499450	+		upstream Pnuc_0507, helix-hairpin-helix motif protein	
m4C	499573	+		Pnuc_0507, helix-hairpin-helix motif protein	
m4C	499947	-		upstream Pnuc_0492, OmpA/MotB domain protein	
m4C	502073	-		Pnuc_0509, lytic murein transglycosylase B	
m4C	507808	+		Pnuc_0514, acyl-CoA dehydrogenase domain protein	
m4C	508502	-		upstream Pnuc_0509, lytic murein transglycosylase B	
m6A	508522	-		upstream Pnuc_0509, lytic murein transglycosylase B	
m4C	508567	+		upstream Pnuc_0517, SSU ribosomal protein S16P	
m6A	511656	+	GAYANNNNNNNRTAG	Pnuc_0520, LSU ribosomal protein L19P	
m6A	511666	-	CTAYNNNNNNNTRTC	upstream Pnuc_0516, protein of unknown function DUF306, Meta and HslJ	
m6A	512017	+		upstream Pnuc_0521, NUDIX hydrolase	
m6A	512339	+		Pnuc_0521, NUDIX hydrolase	
m6A	513793	+	CTAYNNNNNNNTRTC	upstream Pnuc_0525, Exonuclease, RNase T and DNA polymerase III	
m6A	513803	-	GAYANNNNNNNRTAG	Pnuc_0523, ribosome small subunit-dependent GTPase A	
m6A	515484	+		upstream Pnuc_0525, Exonuclease, RNase T and DNA polymerase III	
m6A	515507	+		upstream Pnuc_0525, Exonuclease, RNase T and DNA polymerase III	
m4C	515722	+		upstream Pnuc_0525, Exonuclease, RNase T and DNA polymerase III	
m6A	516835	+	GAYANNNNNNNRTAG	upstream Pnuc_0527, microcin-processing peptidase 1, Unknown type peptidase, MEROPS family U62	
m6A	516845	-	CTAYNNNNNNNTRTC	Pnuc_0526, molybdopterin adenylyltransferase	
m4C	518061	-		upstream Pnuc_0526, molybdopterin adenylyltransferase	
m6A	529410	+	GAYANNNNNNNRTAG	upstream Pnuc_0541, Glutathione S-transferase, N-terminal domain protein	
m6A	529420	-	CTAYNNNNNNNTRTC	upstream Pnuc_0538, hypothetical protein	
m6A	539761	-		upstream Pnuc_0547, putative phytochelatin synthase	
m6A	540300	-		upstream upstream Pnuc_0547, putative phytochelatin synthase	
m4C	548217	+		Pnuc_0556, DoxX family protein	
m6A	553023	+	CTAYNNNNNNNTRTC	upstream Pnuc_0565, hypothetical protein	
m6A	553033	-	GAYANNNNNNNRTAG	Pnuc_0563, integral membrane sensor hybrid histidine kinase	
m6A	555985	+		Pnuc_0566, hypothetical protein	
m6A	556714	+		upstream Pnuc_0570, cation diffusion facilitator family transporter	
m6A	557691	+		upstream Pnuc_0570, cation diffusion facilitator family transporter	
m4C	558569	-		Pnuc_0569, GCN5-related N-acetyltransferase	
m6A	560162	+	GAYANNNNNNNRTAG	Pnuc_0570, cation diffusion facilitator family transporter	
m6A	560172	-	CTAYNNNNNNNTRTC	upstream Pnuc_0569, GCN5-related N-acetyltransferase	
m6A	561977	-		upstream Pnuc_0572, Methyltransferase type 11	
m6A	563811	-		upstream Pnuc_0572, Methyltransferase type 12	
m6A	563878	+		Pnuc_0574, pseudo	
m4C	570856	+		upstream Pnuc_0583, hypothetical protein	

m4C	570932	-		Pnuc_0582, hypothetical protein		
m4C	570962	+		upstream Pnuc_0583, hypothetical protein		
m6A	571162	+		Pnuc_0583, hypothetical protein		
m6A	573053	-		upstream Pnuc_0582, hypothetical protein		
m4C	576206	-		Pnuc_0591, conserved hypothetical protein		
m6A	578571	-		Pnuc_0593, 2-keto-3-deoxygluconate permease		
m6A	587229	+	GAYANNNNNNNNRTAG	upstream Pnuc_0602, Serine--glyoxylate transaminase		
m6A	587239	-	CTAYNNNNNNNNTRTC	Pnuc_0600, FAD linked oxidase domain protein		
m6A	587602	+		upstream Pnuc_0602, Serine--glyoxylate transaminase		
m6A	588730	-		upstream Pnuc_0601, transcriptional regulator, GntR family		
m6A	590985	+		Pnuc_0604, DEAD/DEAH box helicase domain protein		
m6A	597730	+		upstream Pnuc_0610, lipid A ABC exporter, fused ATPase and inner membrane subunits MsbA		
m6A	598039	+		upstream Pnuc_0610, lipid A ABC exporter, fused ATPase and inner membrane subunits MsbA		
m6A	598315	+		upstream Pnuc_0610, lipid A ABC exporter, fused ATPase and inner membrane subunits MsbA		
m6A	598932	+	GAYANNNNNNNNRTAG	upstream Pnuc_0610, lipid A ABC exporter, fused ATPase and inner membrane subunits MsbA		
m6A	598942	-	CTAYNNNNNNNNTRTC	Pnuc_0609, glycosyl transferase, family 2		
m6A	601334	+		upstream Pnuc_R0016, tRNA-Leu		
m4C	602184	-		Pnuc_0611, RNAse G		
m6A	606107	-		Pnuc_0617, phosphoribosylamine-glycine ligase		
m6A	606694	+		upstream Pnuc_R0016, tRNA-Leu		
m6A	607185	+		upstream Pnuc_R0016, tRNA-Leu		
m6A	607706	+		upstream Pnuc_R0016, tRNA-Leu		
m6A	610532	-		Pnuc_0621, permease YjgP/YjgQ family protein		
m6A	610775	-		Pnuc_0621, permease YjgP/YjgQ family protein		
m4C	612570	-		upstream Pnuc_0622, permease YjgP/YjgQ family protein		
m6A	613102	+		Pnuc_0623, aminopeptidase A, Metallo peptidase, MEROPS family M17		
m4C	616738	-		upstream Pnuc_0622, permease YjgP/YjgQ family protein		
m6A	616740	-		upstream Pnuc_0622, permease YjgP/YjgQ family protein		
m6A	618648	+	GAYANNNNNNNNRTAG	Pnuc_0628, TRAP dicarboxylate transporter- DctP subunit		
m6A	618658	-	CTAYNNNNNNNNTRTC	upstream Pnuc_0622, permease YjgP/YjgQ family protein		
m6A	621941	+	GAYANNNNNNNNRTAG	Pnuc_0631, AMP-dependent synthetase and ligase		
m6A	621951	-	CTAYNNNNNNNNTRTC	upstream Pnuc_0622, permease YjgP/YjgQ family protein		
m6A	624339	-		Pnuc_0635, TonB-dependent receptor, plug		
m6A	626322	-		adenosylcobalamin binding moiety (RF00174)		
m4C	626581	-		upstream Pnuc_0635, TonB-dependent receptor, plug		
m6A	627186	-		upstream Pnuc_0635, TonB-dependent receptor, plug		
m4C	628238	-		upstream Pnuc_0635, TonB-dependent receptor, plug		
m6A	632692	+		Pnuc_0641, Methionyl-tRNA formyltransferase		
m6A	633870	+		Pnuc_0643, penicillin amidase, Cysteine peptidase, MEROPS family C59		
m4C	638228	-		Pnuc_0647, methionyl-tRNA synthetase		
m6A	639054	+	GAYANNNNNNNNRTAG	Pnuc_0648, formate dehydrogenase, subunit FdhD		
m6A	639064	-	CTAYNNNNNNNNTRTC	upstream Pnuc_0647, methionyl-tRNA synthetase		
m6A	639443	+		Pnuc_0648, formate dehydrogenase, subunit FdhD		
m6A	640505	-		upstream Pnuc_0647, methionyl-tRNA synthetase		

m6A	640962	-		upstream Pnuc_0647, methionyl-tRNA synthetase	
m4C	641546	-		upstream Pnuc_0647, methionyl-tRNA synthetase	
m6A	642290	+	CTAYNNNNNNNNTRTC	Pnuc_0651, Lysine decarboxylase	
m6A	642300	-	GAYANNNNNNNNRTAG	upstream Pnuc_0647, methionyl-tRNA synthetase	
m4C	648813	+		Pnuc_0657, Uroporphyrinogen III synthase HEM4	
m4C	651326	-		Pnuc_0659, DEAD/DEAH box helicase domain protein	
m4C	653254	+		upstream Pnuc_0662, peptide methionine sulfoxide reductase	
m6A	653833	+		Pnuc_0662, peptide methionine sulfoxide reductase	
m6A	656037	+		upstream Pnuc_0665, amino acid/polyamine/organocation transporter, APC superfamily	
m6A	659995	-		Pnuc_0663, Pyridoxamine 5'-phosphate oxidase	
m6A	664401	+	CTAYNNNNNNNNTRTC	upstream Pnuc_0672, ABC transporter related protein	
m6A	664411	-	GAYANNNNNNNNRTAG	Pnuc_0670, cyanophycin synthetase	
m6A	671237	-		upstream Pnuc_0671, cyanophycin synthetase	
m6A	671297	-		upstream Pnuc_0671, cyanophycin synthetase	
m6A	673790	+		Pnuc_0676, TonB-dependent receptor	
m4C	678877	-		Pnuc_0680, dihydroxyacid dehydratase	
m4C	681991	+		upstream Pnuc_0686, V-type H(+)-translocating pyrophosphatase	
m6A	685642	+	CTAYNNNNNNNNTRTC	Pnuc_0686, V-type H(+)-translocating pyrophosphatase	
m6A	685652	-	GAYANNNNNNNNRTAG	upstream Pnuc_0685, Inorganic diphosphatase	
m6A	686516	+		upstream Pnuc_0687, conserved hypothetical protein	
m4C	688473	+		upstream Pnuc_0690, DNA translocase FtsK	
m6A	688785	+		Pnuc_0690, DNA translocase FtsK	
m6A	688807	-		upstream Pnuc_0689, thioredoxin reductase	
m6A	693622	+		upstream Pnuc_R0018, tRNA-Ser	
m6A	696582	-		upstream Pnuc_0694, transcriptional regulator/antitoxin, MazE	
m4C	699999	-		upstream Pnuc_0699, Exonuclease, RNase T and DNA polymerase III	
m4C	702532	-		upstream Pnuc_R0019, SRP RNA; RNA component of signal recognition particle	
m6A	706399	+		Pnuc_0705, DNA-directed DNA polymerase	
m6A	706438	+		upstream Pnuc_0706, Ferric reductase domain protein protein transmembrane component, N-terminal domain protein	
m6A	706486	-		upstream Pnuc_R0019, SRP RNA; RNA component of signal recognition particle	
m6A	708158	-		Pnuc_0708, hypothetical protein	
m6A	708630	+		upstream Pnuc_0709, protein of unknown function DUF465	
m6A	709971	+		Pnuc_0711, AsmA family protein	
m6A	716204	-		upstream Pnuc_0716, YaeQ family protein	
m6A	716815	+	CTAYNNNNNNNNTRTC	Pnuc_0718, coproporphyrinogen III oxidase, anaerobic	
m6A	716825	-	GAYANNNNNNNNRTAG	upstream Pnuc_0716, YaeQ family protein	
m6A	717198	+	CTAYNNNNNNNNTRTC	upstream Pnuc_0719, OmpW family protein	
m6A	717208	-	GAYANNNNNNNNRTAG	upstream Pnuc_0716, YaeQ family protein	
m6A	720208	+		Pnuc_0721, ABC transporter related protein	
m4C	720366	+		Pnuc_0721, ABC transporter related protein	
m6A	723879	+		upstream Pnuc_0726, TRAP transporter solute receptor, TAXI family	
m6A	729769	-		Pnuc_0730, oligopeptidase A, Metallo peptidase, MEROPS family M03A	
m4C	729810	+		upstream Pnuc_0734, 2-oxo-acid dehydrogenase E1 subunit, homodimeric type	
m4C	729823	+		upstream Pnuc_0734, 2-oxo-acid dehydrogenase E1 subunit, homodimeric type	

m4C	730022	-		Pnuc_0730, oligopeptidase A, Metallo peptidase, MEROPS family M03A		
m6A	730423	+	CTAYNNNNNNNNTRTC	upstream Pnuc_0734, 2-oxo-acid dehydrogenase E1 subunit, homodimeric type		
m6A	730433	-	GAYANNNNNNNNRTAG	Pnuc_0731, methenyltetrahydrofolate cyclohydrolase / 5,10-methylenetetrahydrofolate dehydrogenase (NADP+)		
m6A	732539	-		Pnuc_0733, PAS/PAC sensor signal transduction histidine kinase		
m6A	732949	-		Pnuc_0733, PAS/PAC sensor signal transduction histidine kinase		
m4C	733221	+		upstream Pnuc_0734, 2-oxo-acid dehydrogenase E1 subunit, homodimeric type		
m4C	733398	+		upstream Pnuc_0734, 2-oxo-acid dehydrogenase E1 subunit, homodimeric type		
m6A	734167	+		upstream Pnuc_0734, 2-oxo-acid dehydrogenase E1 subunit, homodimeric type		
m4C	734248	-		Pnuc_0733, PAS/PAC sensor signal transduction histidine kinase		
m6A	739406	+	CTAYNNNNNNNNTRTC	Pnuc_0736, dihydrolipoamide dehydrogenase		
m6A	739416	-	GAYANNNNNNNNRTAG	upstream Pnuc_0733, PAS/PAC sensor signal transduction histidine kinase		
m6A	740234	-		upstream Pnuc_0733, PAS/PAC sensor signal transduction histidine kinase		
m4C	741368	+		upstream Pnuc_0738, protein of unknown function DUF6, transmembrane		
m6A	741643	+	CTAYNNNNNNNNTRTC	Pnuc_0738, protein of unknown function DUF6, transmembrane		
m6A	741653	-	GAYANNNNNNNNRTAG	upstream Pnuc_0737, phasin family protein		
m6A	744485	+		upstream Pnuc_0753, protein of unknown function DUF328		
m4C	745336	-		Pnuc_0741, dihydroorotate oxidase A		
m6A	749953	+		upstream Pnuc_0753, protein of unknown function DUF328		
m6A	750406	+	GAYANNNNNNNNRTAG	upstream Pnuc_0753, protein of unknown function DUF328		
m6A	750416	-	CTAYNNNNNNNNTRTC	Pnuc_0747, DSBA oxidoreductase		
m6A	751528	+		upstream Pnuc_0753, protein of unknown function DUF328		
m6A	754281	+		upstream Pnuc_0753, protein of unknown function DUF328		
m6A	755475	+		Pnuc_0753, protein of unknown function DUF328		
m6A	759180	+		Pnuc_0757,transcriptional regulator, GntR family		
m6A	762452	+	CTAYNNNNNNNNTRTC	Pnuc_0761, succinate dehydrogenase subunit B		
m6A	762462	-	GAYANNNNNNNNRTAG	upstream Pnuc_0756, malate dehydrogenase (NAD)		
m6A	762693	-		upstream Pnuc_0756, malate dehydrogenase (NAD)		
m6A	766548	-		upstream Pnuc_0756, malate dehydrogenase (NAD)		
m6A	768396	+		Pnuc_0768, aspartate semialdehyde dehydrogenase		
m6A	768513	+	CTAYNNNNNNNNTRTC	Pnuc_0768, aspartate semialdehyde dehydrogenase		
m6A	768523	-	GAYANNNNNNNNRTAG	upstream Pnuc_0756, malate dehydrogenase (NAD)		
m6A	770717	+		Pnuc_0769, Tfp pilus assembly protein FimV-like protein		
m6A	774540	+	CTAYNNNNNNNNTRTC	Pnuc_0774, acetyl-CoA carboxylase carboxyltransferase subunit alpha		
m6A	774550	-	GAYANNNNNNNNRTAG	upstream Pnuc_0756, malate dehydrogenase (NAD)		
m6A	777961	-		upstream Pnuc_0756, malate dehydrogenase (NAD)		
m6A	783973	+	GAYANNNNNNNNRTAG	Pnuc_0784, OmpA/MotB domain protein		
m6A	783983	-	CTAYNNNNNNNNTRTC	upstream Pnuc_0782, molybdate ABC transporter, inner membrane subunit		
m6A	786874	+	GAYANNNNNNNNRTAG	upstream Pnuc_0788, protein of unknown function DUF395, YeeE/YedE		
m6A	786884	-	CTAYNNNNNNNNTRTC	Pnuc_0787, cytochrome c family protein		
m4C	787697	-		upstream Pnuc_0787, cytochrome c family protein		
m6A	789389	+	GAYANNNNNNNNRTAG	Pnuc_0792,Rhodanese domain protein		
m6A	789399	-	CTAYNNNNNNNNTRTC	upstream Pnuc_0790, hypothetical protein		
m6A	793524	-		Pnuc_0798, protein of unknown function DUF81		
m6A	793988	+	CTAYNNNNNNNNTRTC	upstream Pnuc_0800, beta-lactamase domain protein		

m6A	793998	-	GAYANNNNNNNRTAG	Pnuc_0799, FAD-dependent pyridine nucleotide-disulfide oxidoreductase
m6A	795260	-		upstream Pnuc_0799, FAD-dependent pyridine nucleotide-disulfide oxidoreductase
m4C	796423	-		upstream Pnuc_0799, FAD-dependent pyridine nucleotide-disulfide oxidoreductase
m6A	797960	+		Pnuc_0802, sulfur dehydrogenase subunit SoxD
m6A	799290	+		Pnuc_0802, sulfur dehydrogenase subunit SoxD
m6A	800576	-		upstream Pnuc_0799, FAD-dependent pyridine nucleotide-disulfide oxidoreductase
m4C	801384	-		upstream Pnuc_0799, FAD-dependent pyridine nucleotide-disulfide oxidoreductase
m6A	802163	+	GAYANNNNNNNRTAG	Pnuc_0807, sulfate thiol esterase SoxB
m6A	802173	-	CTAYNNNNNNNTRTC	upstream Pnuc_0799, FAD-dependent pyridine nucleotide-disulfide oxidoreductase
m4C	804728	-		upstream Pnuc_0799, FAD-dependent pyridine nucleotide-disulfide oxidoreductase
m6A	807320	-		upstream Pnuc_0799, FAD-dependent pyridine nucleotide-disulfide oxidoreductase
m4C	810697	+		upstream Pnuc_0819, conserved hypothetical integral membrane protein
m6A	811058	-		Pnuc_0818, acyl-CoA dehydrogenase domain protein
m4C	811995	-		upstream Pnuc_0818, acyl-CoA dehydrogenase domain protein
m6A	815395	+	GAYANNNNNNNRTAG	upstream Pnuc_0825, 3-oxoacid CoA-transferase, A subunit
m6A	815405	-	CTAYNNNNNNNTRTC	Pnuc_0822, protein of unknown function DUF482
m6A	819321	-		upstream Pnuc_0824, Electron-transferring-flavoprotein dehydrogenase
m6A	820903	+	CTAYNNNNNNNTRTC	Pnuc_0827, cation diffusion facilitator family transporter
m6A	820913	-	GAYANNNNNNNRTAG	upstream Pnuc_0824, Electron-transferring-flavoprotein dehydrogenase
m4C	821539	+		Pnuc_0828, (p)ppGpp synthetase I, SpoT/RelA
m4C	822105	+		Pnuc_0828, (p)ppGpp synthetase I, SpoT/RelA
m6A	822198	+		Pnuc_0828, (p)ppGpp synthetase I, SpoT/RelA
m6A	823095	+		Pnuc_0828, (p)ppGpp synthetase I, SpoT/RelA
m4C	823200	+		Pnuc_0828, (p)ppGpp synthetase I, SpoT/RelA
m6A	829107	+	CTAYNNNNNNNTRTC	Pnuc_0834, phenylalanyl-tRNA synthetase beta subunit
m6A	829117	-	GAYANNNNNNNRTAG	upstream Pnuc_0824, Electron-transferring-flavoprotein dehydrogenase
m6A	834695	-		upstream Pnuc_0839, DNA binding domain, excisionase family
m6A	837877	+	GAYANNNNNNNRTAG	Pnuc_0842, dihydrolipoamide dehydrogenase
m6A	837887	-	CTAYNNNNNNNTRTC	upstream Pnuc_0839, DNA binding domain, excisionase family
m4C	840269	+		Pnuc_0844, PHP C-terminal domain protein
m4C	845973	-		Pnuc_0850, Cupin 4 family protein
m6A	856210	+	GAYANNNNNNNRTAG	Pnuc_0862, aspartate kinase
m6A	856220	-	CTAYNNNNNNNTRTC	upstream Pnuc_0857, Tetratricopeptide TPR_2 repeat protein
m6A	857075	-		upstream Pnuc_0857, Tetratricopeptide TPR_2 repeat protein
m6A	857209	-		upstream Pnuc_0857, Tetratricopeptide TPR_2 repeat protein
m4C	857909	+		Pnuc_0863, metallophosphoesterase
m6A	857935	-		upstream Pnuc_0857, Tetratricopeptide TPR_2 repeat protein
m6A	864766	+	CTAYNNNNNNNTRTC	Pnuc_0870, transporter, hydrophobe/amphiphile efflux-1(HAE1) family
m6A	864776	-	GAYANNNNNNNRTAG	upstream Pnuc_0865, transcriptional regulator, XRE family
m6A	865018	+	CTAYNNNNNNNTRTC	Pnuc_0870, transporter, hydrophobe/amphiphile efflux-1(HAE1) family
m6A	865028	-	GAYANNNNNNNRTAG	upstream Pnuc_0865, transcriptional regulator, XRE family
m6A	865032	-		upstream Pnuc_0865, transcriptional regulator, XRE family
m6A	865350	-		upstream Pnuc_0865, transcriptional regulator, XRE family
m6A	868410	+	GAYANNNNNNNRTAG	Pnuc_0872, Formyl-CoA transferase

m6A	868420	-	CTAYNNNNNNNTRTC	upstream Pnuc_0865, transcriptional regulator, XRE family		
m4C	868513	-		upstream Pnuc_0865, transcriptional regulator, XRE family		
m4C	871319	-		Pnuc_0875, hypothetical protein		
m4C	871406	+		upstream Pnuc_0876, hypothetical protein		
m6A	873168	+	CTAYNNNNNNNTRTC	Pnuc_0879, putative transmembrane protein		
m6A	873178	-	GAYANNNNNNNRTAG	upstream Pnuc_0875, hypothetical protein		
m4C	873322	+		Pnuc_0880, hypothetical protein		
m4C	874847	+		Pnuc_0883, AMP-dependent synthetase and ligase		
m4C	875451	+		Pnuc_0883, AMP-dependent synthetase and ligase		
m4C	878708	+		upstream Pnuc_0888, hypothetical protein		
m6A	881884	-		Pnuc_R0023, tRNA-Asn		
m6A	882224	-	CTAYNNNNNNNTRTC	upstream Pnuc_R0023, tRNA-Asn		
m6A	883131	-		upstream Pnuc_R0023, tRNA-Asn		
m6A	891700	-		Pnuc_0899, Formyl-CoA transferase		
m4C	891750	-		Pnuc_0899, Formyl-CoA transferase		
m6A	894883	+	GAYANNNNNNNRTAG	Pnuc_0901, DNA topoisomerase IV subunit A		
m6A	894893	-	CTAYNNNNNNNTRTC	upstream Pnuc_0899, Formyl-CoA transferase		
m6A	895617	+		Pnuc_0901, DNA topoisomerase IV subunit A		
m4C	896715	+		Pnuc_0901, DNA topoisomerase IV subunit A		
m6A	898234	+	CTAYNNNNNNNTRTC	upstream Pnuc_0903, conserved hypothetical protein		
m6A	898244	-	GAYANNNNNNNRTAG	upstream Pnuc_0902, predicted sulfurylase subunit, molybdopterin cytosine dinucleotide biosynthesis		
m6A	898893	-		upstream Pnuc_0902, predicted sulfurylase subunit, molybdopterin cytosine dinucleotide biosynthesis		
m6A	903152	+	GAYANNNNNNNRTAG	upstream Pnuc_0910, methylmalonyl-CoA mutase		
m6A	903162	-	CTAYNNNNNNNTRTC	upstream Pnuc_0909, transcriptional regulator, GntR family		
m4C	905948	+		Pnuc_0911, LAO/AO transport system ATPase		
m4C	906214	+		Pnuc_0911, LAO/AO transport system ATPase		
m6A	908367	+		Pnuc_0913, acetyl-CoA carboxylase, biotin carboxylase		
m6A	908555	+		Pnuc_0913, acetyl-CoA carboxylase, biotin carboxylase		
m6A	909186	-		upstream Pnuc_0909, transcriptional regulator, GntR family		
m6A	909439	+	CTAYNNNNNNNTRTC	Pnuc_0913, acetyl-CoA carboxylase, biotin carboxylase		
m6A	909449	-	GAYANNNNNNNRTAG	upstream Pnuc_0909, transcriptional regulator, GntR family		
m4C	920817	+		Pnuc_0925, polyhydroxyalkonate synthesis repressor, PhaR		
m4C	926448	-		Pnuc_0929, transcription-repair coupling factor		
m6A	927815	+		upstream Pnuc_0930, 2-C-methyl-D-erythritol 4-phosphate cytidylyltransferase		
m4C	927861	-		Pnuc_0929, transcription-repair coupling factor		
m6A	931352	-		upstream Pnuc_0929, transcription-repair coupling factor		
m6A	933367	+		Pnuc_0935, ATP-dependent Clp protease ATP-binding subunit ClpX		
m4C	933464	+		Pnuc_0935, ATP-dependent Clp protease ATP-binding subunit ClpX		
m6A	935451	+		Pnuc_0936, ATP-dependent proteinase, Serine peptidase, MEROPS family S16		
m4C	936279	-		upstream Pnuc_0929, transcription-repair coupling factor		
m6A	936991	+	CTAYNNNNNNNTRTC	Pnuc_0937, hypothetical protein		
m6A	937001	-	GAYANNNNNNNRTAG	upstream Pnuc_0929, transcription-repair coupling factor		
m6A	939732	+		Pnuc_0940, phosphoribosylformylglycinamidine synthase		
m6A	943496	+		Pnuc_0941, conserved hypothetical protein		

m6A	946091	+	GAYANNNNNNNRTAG	upstream Pnuc_0945, CTP synthase
m6A	946101	-	CTAYNNNNNNNTRTC	Pnuc_0942, aconitase
m4C	946664	+		upstream Pnuc_0945, CTP synthase
m4C	946768	+		upstream Pnuc_0945, CTP synthase
m4C	954511	-		upstream Pnuc_0949, Hsp33 protein
m6A	959740	+	GAYANNNNNNNRTAG	Pnuc_0958, 2-hydroxy-3-oxopropionate reductase
m6A	959750	-	CTAYNNNNNNNTRTC	upstream Pnuc_0957, hypothetical protein
m6A	959916	+		Pnuc_0958, 2-hydroxy-3-oxopropionate reductase
m6A	959987	-		upstream Pnuc_0957, hypothetical protein
m4C	962782	+		upstream Pnuc_0968, glycine cleavage T protein (aminomethyl transferase)
m6A	964248	+		upstream Pnuc_0968, glycine cleavage T protein (aminomethyl transferase)
m6A	964737	+		upstream Pnuc_0968, glycine cleavage T protein (aminomethyl transferase)
m6A	966942	+	CTAYNNNNNNNTRTC	upstream Pnuc_0968, glycine cleavage T protein (aminomethyl transferase)
m6A	966952	-	GAYANNNNNNNRTAG	Pnuc_0967, aminodeoxychorismate lyase
m6A	967979	+	CTAYNNNNNNNTRTC	Pnuc_0968, glycine cleavage T protein (aminomethyl transferase)
m6A	967989	-	GAYANNNNNNNRTAG	upstream Pnuc_0967, aminodeoxychorismate lyase
m6A	974583	+		upstream Pnuc_0980, flavodoxin/nitric oxide synthase
m6A	975403	-		Pnuc_0977, chorismate synthase
m6A	975704	+		upstream Pnuc_0980, flavodoxin/nitric oxide synthase
m4C	979951	-		upstream Pnuc_0979, tRNA-processing RNAse BN
m6A	980984	+	GAYANNNNNNNRTAG	upstream Pnuc_0999, ABC-type transporter, periplasmic component, NitT family
m6A	980994	-	CTAYNNNNNNNTRTC	Pnuc_0983, alpha/beta hydrolase fold protein
m6A	982286	+	CTAYNNNNNNNTRTC	upstream Pnuc_0999, ABC-type transporter, periplasmic component, NitT family
m6A	982296	-	GAYANNNNNNNRTAG	Pnuc_0986, hypothetical protein
m6A	988136	+	GAYANNNNNNNRTAG	upstream Pnuc_0999, ABC-type transporter, periplasmic component, NitT family
m6A	988146	-	CTAYNNNNNNNTRTC	Pnuc_0992, Choloylglycine hydrolase
m4C	993458	-		upstream Pnuc_0997, ABC transporter nitrate-binding protein
m6A	995404	+	CTAYNNNNNNNTRTC	Pnuc_0999, ABC-type transporter, periplasmic component, NitT family
m6A	995414	-	GAYANNNNNNNRTAG	upstream Pnuc_0998, uroporphyrin-III C-methyltransferase
m6A	997866	+		Pnuc_1000, assimilatory nitrite reductase (NAD(P)H) large subunit precursor
m6A	999078	-		upstream Pnuc_0998, uroporphyrin-III C-methyltransferase
m6A	1000022	+		Pnuc_1002, assimilatory nitrate reductase (NADH) alpha subunit apoprotein
m6A	1000889	+		Pnuc_1002, assimilatory nitrate reductase (NADH) alpha subunit apoprotein
m4C	1001341	-		upstream Pnuc_0998, uroporphyrin-III C-methyltransferase
m6A	1001443	-		upstream Pnuc_0998, uroporphyrin-III C-methyltransferase
m4C	1003514	+		upstream Pnuc_1007, protein of unknown function DUF1348
m4C	1005364	+		Pnuc_1008, nitroreductase
m6A	1008651	+	CTAYNNNNNNNTRTC	Pnuc_1010, TonB-dependent receptor
m6A	1008661	-	GAYANNNNNNNRTAG	upstream Pnuc_R0031, tRNA-Pro2
m4C	1009132	+		upstream Pnuc_1015, protein of unknown function UPF0044
m6A	1010336	+		upstream Pnuc_1015, protein of unknown function UPF0044
m6A	1013228	+		upstream Pnuc_1015, protein of unknown function UPF0044
m4C	1016761	+		upstream Pnuc_1023, Methyltransferase type 11
m6A	1019053	+	CTAYNNNNNNNTRTC	upstream Pnuc_1023, Methyltransferase type 11

m6A	1019063	-	GAYANNNNNNNRTAG	Pnuc_1019, carbamoyl-phosphate synthase small subunit	
m6A	1019396	-		Pnuc_1019, carbamoyl-phosphate synthase small subunit	
m6A	1020266	-	GAYANNNNNNNRTAG	upstream Pnuc_1019, carbamoyl-phosphate synthase small subunit	
m4C	1021239	+		upstream Pnuc_1023, Methyltransferase type 11	
m6A	1021303	+	CTAYNNNNNNNTRTC	upstream Pnuc_1023, Methyltransferase type 11	
m6A	1021313	-	GAYANNNNNNNRTAG	Pnuc_1020, propionyl-CoA synthetase	
m4C	1021746	+		upstream Pnuc_1023, Methyltransferase type 11	
m6A	1027364	+		Pnuc_1026, acriflavin resistance protein	
m6A	1027462	+	GAYANNNNNNNRTAG	Pnuc_1026, acriflavin resistance protein	
m6A	1027472	-	CTAYNNNNNNNTRTC	upstream Pnuc_1022, Hydroxyacylglutathione hydrolase	
m4C	1030880	+		Pnuc_1027, DNA polymerase III, epsilon subunit	
m6A	1032141	+		upstream Pnuc_1032, Amidase	
m4C	1033093	-		Pnuc_1030, hypothetical protein	
m6A	1033357	+		upstream Pnuc_1032, Amidase	
m6A	1034059	-		Pnuc_1031, hypothetical protein	
m6A	1034060	+	CTAYNNNNNNNTRTC	upstream Pnuc_1032, Amidase	
m6A	1034070	-	GAYANNNNNNNRTAG	Pnuc_1031, hypothetical protein	
m4C	1036386	-		Pnuc_1033, Uncharacterized protein UPF0065	
m6A	1038226	+		upstream Pnuc_1035, methylmalonate-semialdehyde dehydrogenase (acylating)	
m6A	1042338	+		upstream Pnuc_1057, putative lipoprotein	
m4C	1043542	+		upstream Pnuc_1057, putative lipoprotein	
m4C	1046690	+		upstream Pnuc_1057, putative lipoprotein	
m6A	1050989	+		upstream Pnuc_1057, putative lipoprotein	
m6A	1055538	+	GAYANNNNNNNRTAG	upstream Pnuc_1057, putative lipoprotein	
m6A	1055548	-	CTAYNNNNNNNTRTC	Pnuc_1051, NADH dehydrogenase subunit A	
m4C	1055615	-		Pnuc_1051, NADH dehydrogenase subunit A	
m6A	1061186	-		upstream Pnuc_1056, SSU ribosomal protein S15P	
m6A	1061342	-		upstream Pnuc_1056, SSU ribosomal protein S15P	
m4C	1064577	+		upstream Pnuc_1064, RNA polymerase, sigma-24 subunit, RpoE	
m4C	1068864	+		Pnuc_1064, RNA polymerase, sigma-24 subunit, RpoE	
m4C	1068952	-		upstream Pnuc_1063, acetolactate synthase, large subunit	
m4C	1068979	+		Pnuc_1064, RNA polymerase, sigma-24 subunit, RpoE	
m4C	1071149	-		Pnuc_1068, protein of unknown function DUF214	
m4C	1071207	+		upstream Pnuc_1075, transcription elongation factor GreB	
m6A	1073342	+		upstream Pnuc_1075, transcription elongation factor GreB	
m6A	1074889	-		Pnuc_1070, Uncharacterized protein UPF0065	
m4C	1075611	+		upstream Pnuc_1075, transcription elongation factor GreB	
m6A	1075780	+		upstream Pnuc_1075, transcription elongation factor GreB	
m6A	1075790	-		Pnuc_1071, hypothetical protein	
m6A	1076951	+	CTAYNNNNNNNTRTC	upstream Pnuc_1075, transcription elongation factor GreB	
m6A	1076961	-	GAYANNNNNNNRTAG	Pnuc_1073, ABC transporter related protein	
m6A	1077434	+		upstream Pnuc_1075, transcription elongation factor GreB	
m6A	1082384	+		upstream Pnuc_1080, RNAse PH	
m6A	1082427	+		upstream Pnuc_1080, RNAse PH	

m6A	1088310	-		Pnuc_1084, beta-lactamase domain protein		
m6A	1090450	+		upstream Pnuc_1086, Patatin		
m6A	1093084	+	GAYANNNNNNNRTAG	Pnuc_1087, hypothetical protein		
m6A	1093094	-	CTAYNNNNNNNTRTC	upstream Pnuc_1085, integral membrane sensor signal transduction histidine kinase		
m6A	1095712	+	CTAYNNNNNNNTRTC	upstream Pnuc_1091, glucose-6-phosphate isomerase		
m6A	1095722	-	GAYANNNNNNNRTAG	upstream Pnuc_1089, hypothetical protein		
m6A	1101440	+		upstream Pnuc_1097, hypothetical protein		
m6A	1108023	+	GAYANNNNNNNRTAG	upstream Pnuc_1097, hypothetical protein		
m6A	1108033	-	CTAYNNNNNNNTRTC	Pnuc_1095, outer membrane autotransporter barrel domain protein		
m6A	1109515	+	CTAYNNNNNNNTRTC	upstream Pnuc_1097, hypothetical protein		
m6A	1109525	-	GAYANNNNNNNRTAG	Pnuc_1095, outer membrane autotransporter barrel domain protein		
m6A	1111707	+	GAYANNNNNNNRTAG	upstream Pnuc_1097, hypothetical protein		
m6A	1111717	-	CTAYNNNNNNNTRTC	Pnuc_1095, outer membrane autotransporter barrel domain protein		
m6A	1111938	+		upstream Pnuc_1097, hypothetical protein		
m4C	1112185	-		Pnuc_1095, outer membrane autotransporter barrel domain protein		
m6A	1112942	+		upstream Pnuc_1097, hypothetical protein		
m4C	1114862	+		upstream Pnuc_1097, hypothetical protein		
m6A	1115655	-		Pnuc_1095, outer membrane autotransporter barrel domain protein		
m6A	1115916	+	GAYANNNNNNNRTAG	upstream Pnuc_1097, hypothetical protein		
m6A	1115926	-	CTAYNNNNNNNTRTC	Pnuc_1095, outer membrane autotransporter barrel domain protein		
m4C	1116418	+		upstream Pnuc_1097, hypothetical protein		
m6A	1117112	+		upstream Pnuc_1097, hypothetical protein		
m4C	1117990	+		upstream Pnuc_1097, hypothetical protein		
m6A	1120412	+		upstream Pnuc_1097, hypothetical protein		
m6A	1121807	+		upstream Pnuc_1097, hypothetical protein		
m6A	1122497	-		Pnuc_1095, outer membrane autotransporter barrel domain protein		
m6A	1124061	-		Pnuc_1095, outer membrane autotransporter barrel domain protein		
m6A	1124249	+		upstream Pnuc_1097, hypothetical protein		
m6A	1125273	+		upstream Pnuc_1097, hypothetical protein		
m6A	1129263	-		Pnuc_1095, outer membrane autotransporter barrel domain protein		
m6A	1129364	-		Pnuc_1095, outer membrane autotransporter barrel domain protein		
m6A	1130061	+		upstream Pnuc_1097, hypothetical protein		
m4C	1138027	-		Pnuc_1096, sulfotransferase		
m6A	1138538	-		Pnuc_1096, sulfotransferase		
m6A	1139780	-		upstream Pnuc_1096, sulfotransferase		
m6A	1145089	+		Pnuc_1103, quinolinate synthetase A		
m6A	1150445	+	GAYANNNNNNNRTAG	upstream Pnuc_1112, protein of unknown function DUF1080		
m6A	1150455	-	CTAYNNNNNNNTRTC	Pnuc_1107, transcriptional modulator of MazE/toxin, MazF		
m6A	1151487	+	GAYANNNNNNNRTAG	upstream Pnuc_1112, protein of unknown function DUF1080		
m6A	1151497	-	CTAYNNNNNNNTRTC	Pnuc_1109, NAD-dependent formate dehydrogenase iron-sulfur protein (catalytic activity)		
m6A	1156346	+	CTAYNNNNNNNTRTC	upstream Pnuc_1112, protein of unknown function DUF1080		
m6A	1156356	-	GAYANNNNNNNRTAG	Pnuc_1111, protein of unknown function DUF748		
m6A	1157974	-		Pnuc_1111, protein of unknown function DUF748		
m6A	1159304	+	GAYANNNNNNNRTAG	Pnuc_1112, protein of unknown function DUF1080		

m6A	1159314	-	CTAYNNNNNNNTRTC	upstream Pnuc_1111, protein of unknown function DUF748		
m6A	1159365	+		Pnuc_1112, protein of unknown function DUF1080		
m6A	1160589	-		upstream Pnuc_1115, YCII-related protein		
m6A	1160818	+		Pnuc_1116, phage integrase family protein		
m6A	1162259	-		Pnuc_1117, hypothetical protein		
m6A	1164942	-		upstream Pnuc_1119, hypothetical protein		
m6A	1167044	+	CTAYNNNNNNNTRTC	Pnuc_1122, Exonuclease, RNase T and DNA polymerase III		
m6A	1167054	-	GAYANNNNNNNRTAG	upstream Pnuc_1121, hypothetical protein		
m6A	1170355	-		Pnuc_1124, hypothetical protein		
m4C	1173713	+		upstream Pnuc_1127, hypothetical protein		
m6A	1173977	+	GAYANNNNNNNRTAG	Pnuc_1127, hypothetical protein		
m6A	1173987	-	CTAYNNNNNNNTRTC	upstream Pnuc_1126, hypothetical protein		
m4C	1176445	-		upstream Pnuc_1129, conserved hypothetical protein		
m6A	1177319	+	GAYANNNNNNNRTAG	upstream Pnuc_1131, phage transcriptional regulator, AlpA		
m6A	1177329	-	CTAYNNNNNNNTRTC	Pnuc_1130, protein of unknown function DUF1376		
m6A	1177333	-		Pnuc_1130, protein of unknown function DUF1376		
m4C	1177393	+		upstream Pnuc_1131, phage transcriptional regulator, AlpA		
m6A	1180795	-		Pnuc_1133, N-6 DNA methylase		
m4C	1187078	+		upstream Pnuc_1139, fumarase		
m4C	1188896	+		upstream Pnuc_1139, fumarase		
m6A	1189256	+		Pnuc_1139, fumarase		
m6A	1191165	+	CTAYNNNNNNNTRTC	Pnuc_1140, glutamate racemase		
m6A	1191175	-	GAYANNNNNNNRTAG	upstream Pnuc_1138, acetyl-coenzyme A synthetase		
m4C	1197227	+		upstream Pnuc_1150, Penicillin amidase		
m6A	1199103	+		upstream Pnuc_1150, Penicillin amidase		
m4C	1199796	+		upstream Pnuc_1150, Penicillin amidase		
m6A	1199870	+	GAYANNNNNNNRTAG	upstream Pnuc_1150, Penicillin amidase		
m6A	1199880	-	CTAYNNNNNNNTRTC	Pnuc_1149, filamentous hemagglutinin family outer membrane protein		
m6A	1201836	-		Pnuc_1149, filamentous hemagglutinin family outer membrane protein		
m4C	1204187	+		upstream Pnuc_1150, Penicillin amidase		
m6A	1205143	-		upstream Pnuc_R0036, tRNA-Leu		
m6A	1206854	+	CTAYNNNNNNNTRTC	Pnuc_1150, Penicillin amidase		
m6A	1206864	-	GAYANNNNNNNRTAG	upstream Pnuc_R0036, tRNA-Leu		
m6A	1211099	-		Pnuc_1158, aldehyde oxidase and xanthine dehydrogenase, molybdopterin binding protein		
m6A	1218238	+	CTAYNNNNNNNTRTC	upstream Pnuc_1167, Cellulase		
m6A	1218248	-	GAYANNNNNNNRTAG	Pnuc_1164, conserved hypothetical protein		
m6A	1221438	+		upstream Pnuc_1167, Cellulase		
m4C	1221658	-		Pnuc_1166, metal dependent phosphohydrolase		
m6A	1224126	+		Pnuc_1168, conserved hypothetical protein		
m6A	1227335	+		upstream Pnuc_1176, hypothetical protein		
m6A	1228000	+	GAYANNNNNNNRTAG	upstream Pnuc_1176, hypothetical protein		
m6A	1228010	-	CTAYNNNNNNNTRTC	Pnuc_1170, cellulose synthase operon C domain protein		
m6A	1228683	+	GAYANNNNNNNRTAG	upstream Pnuc_1176, hypothetical protein		
m6A	1228693	-	CTAYNNNNNNNTRTC	Pnuc_1170, cellulose synthase operon C domain protein		

m6A	1228990	+	CTAYNNNNNNNTRTC	upstream Pnuc_1176, hypothetical protein
m6A	1229000	-	GAYANNNNNNNRTAG	Pnuc_1171, Cellulose synthase (UDP-forming)
m6A	1229564	+		upstream Pnuc_1176, hypothetical protein
m6A	1235057	+		Pnuc_1176, hypothetical protein
m6A	1236712	+	CTAYNNNNNNNTRTC	upstream Pnuc_1184, Amidase
m6A	1239968	+	GAYANNNNNNNRTAG	upstream Pnuc_1184, Amidase
m6A	1239978	-	CTAYNNNNNNNTRTC	Pnuc_1180, putative ABC transporter, periplasmic protein
m6A	1240127	+	GAYANNNNNNNRTAG	upstream Pnuc_1184, Amidase
m6A	1240137	-	CTAYNNNNNNNTRTC	Pnuc_1180, putative ABC transporter, periplasmic protein
m4C	1244461	+		Pnuc_1185, ABC transporter, substrate binding protein
m6A	1245022	+		Pnuc_1186, binding-protein-dependent transport systems inner membrane component
m6A	1247136	+		upstream Pnuc_1210, Glutamate synthase (NADPH)
m6A	1247968	+	CTAYNNNNNNNTRTC	upstream Pnuc_1210, Glutamate synthase (NADPH)
m6A	1247978	-	GAYANNNNNNNRTAG	Pnuc_1190, urease accessory protein UreG
m4C	1248316	-		Pnuc_1191, Urease accessory protein UreF
m6A	1252367	+		upstream Pnuc_1210, Glutamate synthase (NADPH)
m6A	1252457	+	GAYANNNNNNNRTAG	upstream Pnuc_1210, Glutamate synthase (NADPH)
m6A	1252467	-	CTAYNNNNNNNTRTC	Pnuc_1196, urease, gamma subunit
m4C	1253909	-		Pnuc_1198, amino acid/amide ABC transporter ATP-binding protein 2, HAAT family
m4C	1262269	-		Pnuc_1205, protein of unknown function DUF1501
m6A	1262425	+	CTAYNNNNNNNTRTC	upstream Pnuc_1210, Glutamate synthase (NADPH)
m6A	1262435	-	GAYANNNNNNNRTAG	Pnuc_1205, protein of unknown function DUF1501
m4C	1262636	-		Pnuc_1205, protein of unknown function DUF1501
m4C	1267728	-		Pnuc_1211, diguanylate cyclase
m4C	1273950	-		upstream Pnuc_1217, Glutathione S-transferase, N-terminal domain protein
m6A	1278338	-		upstream Pnuc_1217, Glutathione S-transferase, N-terminal domain protein
m6A	1280531	-		Pnuc_1222, RNA binding S1 domain protein
m4C	1282187	-		Pnuc_1222, RNA binding S1 domain protein
m6A	1285244	-		Pnuc_1224, GTP-binding protein TypA
m6A	1285533	-		Pnuc_1224, GTP-binding protein TypA
m6A	1286269	+		upstream Pnuc_1232, Patatin
m6A	1286462	+		upstream Pnuc_1232, Patatin
m6A	1289563	+		upstream Pnuc_1232, Patatin
m4C	1290028	+		upstream Pnuc_1232, Patatin
m6A	1291635	-		Pnuc_1229, protein of unknown function DUF150
m6A	1296429	+	CTAYNNNNNNNTRTC	upstream Pnuc_1238, Enoyl-[acyl-carrier-protein] reductase (NADH)
m6A	1296439	-	GAYANNNNNNNRTAG	upstream Pnuc_1233, NLP/P60 protein
m6A	1297321	+		upstream Pnuc_1238, Enoyl-[acyl-carrier-protein] reductase (NADH)
m6A	1300658	+		upstream Pnuc_1238, Enoyl-[acyl-carrier-protein] reductase (NADH)
m6A	1304342	-		upstream Pnuc_1237, extracellular solute-binding protein, family 5
m6A	1306971	+		Pnuc_1242, MmgE/PrpD family protein
m6A	1307149	+	GAYANNNNNNNRTAG	Pnuc_1242, MmgE/PrpD family protein
m6A	1307159	-	CTAYNNNNNNNTRTC	upstream Pnuc_1237, extracellular solute-binding protein, family 5
m6A	1307337	+		Pnuc_1242, MmgE/PrpD family protein

m6A	1311414	+	CTAYNNNNNNNNTRTC	upstream Pnuc_1249, integrase, catalytic region	
m6A	1311424	-	GAYANNNNNNNNRTAG	Pnuc_1246, BNR/Asp-box repeat protein	
m4C	1313449	+		upstream Pnuc_1249, integrase, catalytic region	
m6A	1317420	+	CTAYNNNNNNNNTRTC	upstream Pnuc_1262, N-acetylglutamate synthase	
m6A	1317430	-	GAYANNNNNNNNRTAG	Pnuc_1252, Exodeoxyribonuclease III	
m6A	1319635	-		Pnuc_1254, signal transduction histidine kinase, nitrogen specific, NtrB	
m4C	1322023	-		upstream Pnuc_1255, L-glutamine synthetase	
m6A	1328635	-		Pnuc_1261, ATP-dependent helicase HrpA	
m6A	1330744	-		upstream Pnuc_1261, ATP-dependent helicase HrpA	
m6A	1338830	+		upstream Pnuc_R0038, tRNA-Leu	
m6A	1339251	+		upstream Pnuc_R0038, tRNA-Leu	
m6A	1343384	+		Pnuc_1272, amino acid permease-associated region	
m4C	1346506	-		Pnuc_1276, Carboxymethylenebutenolidase	
m6A	1347380	-		Pnuc_1277, major facilitator superfamily MFS_1	
m6A	1347471	+		upstream Pnuc_1278, hypothetical protein	
m4C	1351779	-		Pnuc_1281, Adenylosuccinate synthetase	
m6A	1353636	+		upstream Pnuc_1294, protein of unknown function UPF0005	
m4C	1359614	+		upstream Pnuc_1294, protein of unknown function UPF0005	
m6A	1362325	+		upstream Pnuc_1294, protein of unknown function UPF0005	
m6A	1362988	+		upstream Pnuc_1294, protein of unknown function UPF0005	
m6A	1365158	+		upstream Pnuc_1294, protein of unknown function UPF0005	
m4C	1365230	+		upstream Pnuc_1294, protein of unknown function UPF0005	
m4C	1367524	-		Pnuc_1295, 23S rRNA m(5)U-1939 methyltransferase	
m6A	1373200	+		upstream Pnuc_1307, thioredoxin	
m4C	1373904	-		Pnuc_1300, heavy metal efflux pump, CzcA family	
m4C	1374186	-		Pnuc_1300, heavy metal efflux pump, CzcA family	
m6A	1375845	-		Pnuc_1302, outer membrane efflux protein	
m4C	1377231	+		upstream Pnuc_1307, thioredoxin	
m6A	1377446	-		Pnuc_1302, outer membrane efflux protein	
m4C	1378511	-		Pnuc_1304, Tetratricopeptide TPR_2 repeat protein	
m4C	1380280	+		upstream Pnuc_1307, thioredoxin	
m4C	1382305	+		upstream Pnuc_1307, thioredoxin	
m6A	1383404	+		upstream Pnuc_1307, thioredoxin	
m6A	1383845	+		upstream Pnuc_1307, thioredoxin	
m4C	1389663	+		Pnuc_1310, putative inner membrane transmembrane protein	
m6A	1389671	-		upstream Pnuc_1306, DNA helicase/exodeoxyribonuclease V, subunit B	
m6A	1399354	+		upstream Pnuc_1323, Uncharacterized protein UPF0065	
m6A	1408563	-		upstream Pnuc_1330, FeoA family protein	
m6A	1408639	+		Pnuc_1331, Carbohydrate-selective porin OprB	
m6A	1410626	+	GAYANNNNNNNNRTAG	Pnuc_1332, negative transcriptional regulator	
m6A	1410636	-	CTAYNNNNNNNNTRTC	upstream Pnuc_1330, FeoA family protein	
m6A	1412582	+		upstream Pnuc_1336, putative sulfate transport system substrate-binding protein	
m6A	1413827	+		upstream Pnuc_1336, putative sulfate transport system substrate-binding protein	
m4C	1413951	-		Pnuc_1335, transcriptional regulator of molybdate metabolism, LysR family	

m6A	1413960	-		Pnuc_1335, transcriptional regulator of molybdate metabolism, LysR family		
m6A	1415967	-		Pnuc_1338, hypothetical protein		
m6A	1416772	-	GAYANNNNNNNRTAG	upstream Pnuc_1338, hypothetical protein		
m6A	1417579	+	GAYANNNNNNNRTAG	Pnuc_1341, conserved hypothetical protein		
m6A	1417589	-	CTAYNNNNNNNTRTC	upstream Pnuc_1338, hypothetical protein		
m6A	1419263	+	CTAYNNNNNNNTRTC	Pnuc_1343, transglutaminase domain protein		
m6A	1422263	+	CTAYNNNNNNNTRTC	upstream Pnuc_1347, protein of unknown function DUF1330		
m6A	1422273	-	GAYANNNNNNNRTAG	upstream Pnuc_1346, conserved hypothetical protein		
m4C	1425242	+		Pnuc_1350, Rhodanese domain protein		
m4C	1427115	+		Pnuc_1352, hypothetical protein		
m4C	1432162	-		upstream Pnuc_1359, conserved hypothetical protein 730		
m6A	1434445	+	CTAYNNNNNNNTRTC	upstream Pnuc_1363, carbohydrate kinase, YjeF related protein		
m6A	1434455	-	GAYANNNNNNNRTAG	Pnuc_1362, RNA-metabolising metallo-beta-lactamase		
m4C	1435865	-		upstream Pnuc_1362, RNA-metabolising metallo-beta-lactamase		
m6A	1437655	-		Pnuc_1367, aminotransferase, class V		
m6A	1438918	-		Pnuc_1368, Uncharacterized protein UPF0065		
m6A	1439629	-		Pnuc_1368, Uncharacterized protein UPF0065		
m6A	1439659	-		Pnuc_1368, Uncharacterized protein UPF0065		
m4C	1440473	-		Pnuc_1369, 5-carboxymethyl-2-hydroxymuconate Delta-isomerase		
m4C	1444140	-		upstream Pnuc_1370, protein of unknown function DUF6, transmembrane		
m4C	1446510	-		Pnuc_1375, transcriptional regulator		
m4C	1447384	+		Pnuc_1376, Rubrerythrin		
m4C	1447784	-		upstream Pnuc_1375, transcriptional regulator		
m6A	1448329	+		Pnuc_1378, FAD-dependent pyridine nucleotide-disulfide oxidoreductase		
m6A	1451338	+		upstream Pnuc_1382, DSBA oxidoreductase		
m6A	1453284	+		upstream Pnuc_1382, DSBA oxidoreductase		
m6A	1461662	-		Pnuc_1390, cardiolipin synthetase 2		
m6A	1464729	+	GAYANNNNNNNRTAG	upstream Pnuc_1407, heavy metal translocating P-type ATPase		
m6A	1464739	-	CTAYNNNNNNNTRTC	Pnuc_1395, formate dehydrogenase gamma subunit		
m4C	1466782	+		upstream Pnuc_1407, heavy metal translocating P-type ATPase		
m6A	1467035	+		upstream Pnuc_1407, heavy metal translocating P-type ATPase		
m6A	1473645	+	GAYANNNNNNNRTAG	upstream Pnuc_1407, heavy metal translocating P-type ATPase		
m6A	1473655	-	CTAYNNNNNNNTRTC	Pnuc_1402, conserved hypothetical protein		
m6A	1473786	+		upstream Pnuc_1407, heavy metal translocating P-type ATPase		
m4C	1474986	-		upstream Pnuc_1403, protein of unknown function DUF59		
m4C	1475177	+		upstream Pnuc_1407, heavy metal translocating P-type ATPase		
m4C	1478223	-		upstream Pnuc_1406, Heavy metal transport/detoxification protein		
m4C	1478440	+		Pnuc_1407, heavy metal translocating P-type ATPase		
m4C	1480546	+		Pnuc_1409, 4-amino-4-deoxy-L-arabinose transferase and related glycosyltransferase of PMT family-like protein		
m6A	1483806	-		upstream Pnuc_1408, conserved hypothetical protein		
m6A	1484850	-		upstream Pnuc_1408, conserved hypothetical protein		
m6A	1487423	-		Pnuc_1416, CMP/dCMP deaminase, zinc-binding protein		
m6A	1488814	+	CTAYNNNNNNNTRTC	upstream Pnuc_1426, SsrA-binding protein		
m6A	1488824	-	GAYANNNNNNNRTAG	Pnuc_1418, Radical SAM domain protein		

m6A	1489493	+		upstream Pnuc_1426, SsrA-binding protein		
m4C	1490700	+		upstream Pnuc_1426, SsrA-binding protein		
m6A	1494931	-		Pnuc_1423, inosine-5'-monophosphate dehydrogenase		
m4C	1496081	+		upstream Pnuc_1426, SsrA-binding protein		
m6A	1496168	+	CTAYNNNNNNNNTRTC	upstream Pnuc_1426, SsrA-binding protein		
m6A	1496178	-	GAYANNNNNNNNRTAG	Pnuc_1425, cyclase/dehydrase		
m6A	1496760	+		Pnuc_1426, SsrA-binding protein		
m4C	1496925	+		upstream Pnuc_1427, protein of unknown function DUF404		
m6A	1498558	+		Pnuc_1428, protein of unknown function DUF403		
m4C	1498741	+		Pnuc_1428, protein of unknown function DUF403		
m6A	1499062	-		upstream Pnuc_1425, cyclase/dehydrase		
m6A	1504352	-		Pnuc_1433, phosphoenolpyruvate synthase		
m6A	1505598	+	CTAYNNNNNNNNTRTC	Pnuc_1434, protein of unknown function DUF299		
m6A	1505608	-	GAYANNNNNNNNRTAG	Pnuc_1433, phosphoenolpyruvate synthase		
m4C	1505738	+		Pnuc_1434, protein of unknown function DUF299		
m4C	1507547	+		upstream Pnuc_1452, methionine aminopeptidase, type I		
m4C	1508926	-		Pnuc_1438, lipid-A-disaccharide synthase		
m6A	1517912	+		upstream Pnuc_1452, methionine aminopeptidase, type I		
m6A	1519356	+	CTAYNNNNNNNNTRTC	upstream Pnuc_1452, methionine aminopeptidase, type I		
m6A	1519366	-	GAYANNNNNNNNRTAG	Pnuc_1448, ribosome recycling factor		
m6A	1520796	-		Pnuc_1450, translation elongation factor Ts (EF-Ts)		
m6A	1520804	+		upstream Pnuc_1452, methionine aminopeptidase, type I		
m4C	1523178	+		Pnuc_1452, methionine aminopeptidase, type I		
m6A	1526171	+		upstream Pnuc_1458, 2,3,4,5-tetrahydropyridine-2,6-dicarboxylate N-succinyltransferase		
m6A	1526426	+		upstream Pnuc_1458, 2,3,4,5-tetrahydropyridine-2,6-dicarboxylate N-succinyltransferase		
m6A	1528113	-		Pnuc_1455, DNA ligase, NAD-dependent		
m4C	1528219	+		upstream Pnuc_1458, 2,3,4,5-tetrahydropyridine-2,6-dicarboxylate N-succinyltransferase		
m6A	1530405	-		Pnuc_1457, chromosome segregation protein SMC		
m6A	1548614	-		upstream Pnuc_1468, lipoprotein releasing system, transmembrane protein, LolC/E family		
m6A	1548948	-		upstream Pnuc_1468, lipoprotein releasing system, transmembrane protein, LolC/E family		
m6A	1552414	+	GAYANNNNNNNNRTAG	upstream Pnuc_1477, peptide deformylase		
m6A	1552424	-	CTAYNNNNNNNNTRTC	Pnuc_1473, hypothetical protein		
m4C	1554275	-		Pnuc_1476, cysteine synthase A		
m4C	1555954	+		upstream Pnuc_1479, Ferredoxin--NADP(+) reductase		
m6A	1557612	+	CTAYNNNNNNNNTRTC	Pnuc_1480, protein of unknown function DUF81		
m6A	1557622	-	GAYANNNNNNNNRTAG	upstream Pnuc_1478, disulfide bond formation protein DsbB		
m6A	1558540	-		upstream Pnuc_1478, disulfide bond formation protein DsbB		
m6A	1560951	+		Pnuc_1483, sulfate adenylyltransferase subunit 1		
m6A	1561444	+		Pnuc_1484, hypothetical protein		
m6A	1561655	+		Pnuc_1484, hypothetical protein		
m6A	1564637	+		upstream Pnuc_1496, aminotransferase		
m6A	1568411	+		upstream Pnuc_1496, aminotransferase		
m6A	1570108	+		upstream Pnuc_1496, aminotransferase		
m6A	1570379	-		Pnuc_1495, Excinuclease ABC subunit B		

m4C	1570421	-		Pnuc_1495, Excinuclease ABC subunit B		
m6A	1570842	+	CTAYNNNNNNNTRTC	upstream Pnuc_1496, aminotransferase		
m6A	1570852	-	GAYANNNNNNNRTAG	Pnuc_1495, Excinuclease ABC subunit B		
m4C	1572033	-		Pnuc_1495, Excinuclease ABC subunit B		
m6A	1572464	-		upstream Pnuc_1495, Excinuclease ABC subunit B		
m4C	1572536	+		upstream Pnuc_1496, aminotransferase		
m4C	1572718	-		upstream Pnuc_1495, Excinuclease ABC subunit B		
m4C	1575927	-		upstream Pnuc_1495, Excinuclease ABC subunit B		
m4C	1581403	-		Pnuc_1506, isocitrate lyase		
m6A	1582960	+	GAYANNNNNNNRTAG	upstream Pnuc_1528, conserved hypothetical protein		
m6A	1582970	-	CTAYNNNNNNNTRTC	Pnuc_1507, major facilitator superfamily MFS_1		
m6A	1591897	+	CTAYNNNNNNNTRTC	upstream Pnuc_1528, conserved hypothetical protein		
m6A	1591907	-	GAYANNNNNNNRTAG	Pnuc_1518, heme exporter protein CcmA		
m4C	1595424	+		upstream Pnuc_1528, conserved hypothetical protein		
m6A	1599496	+	CTAYNNNNNNNTRTC	Pnuc_1529, histone deacetylase superfamily		
m6A	1599506	-	GAYANNNNNNNRTAG	upstream Pnuc_1527, hypothetical protein		
m6A	1601203	+	GAYANNNNNNNRTAG	upstream Pnuc_1534, alkyl hydroperoxide reductase/ Thiol specific antioxidant/ Mal allergen		
m6A	1601213	-	CTAYNNNNNNNTRTC	Pnuc_1531, putative lipoprotein		
m6A	1602159	+		upstream Pnuc_1534, alkyl hydroperoxide reductase/ Thiol specific antioxidant/ Mal allergen		
m6A	1602318	-		Pnuc_1533, hypothetical protein		
m6A	1606433	+	GAYANNNNNNNRTAG	upstream Pnuc_1544, transcriptional regulator, GntR family		
m6A	1606443	-	CTAYNNNNNNNTRTC	Pnuc_1538, TRAP C4-dicarboxylate transport system permease DctM subunit		
m6A	1609714	-		Pnuc_1540, short-chain dehydrogenase/reductase SDR		
m4C	1613302	+		upstream Pnuc_1544, transcriptional regulator, GntR family		
m6A	1616212	-		upstream Pnuc_1545, class II aldolase/adducin family protein		
m6A	1617376	-		upstream Pnuc_1545, class II aldolase/adducin family protein		
m6A	1617388	+	GAYANNNNNNNRTAG	Pnuc_1547, protein of unknown function DUF6, transmembrane		
m6A	1617398	-	CTAYNNNNNNNTRTC	upstream Pnuc_1545, class II aldolase/adducin family protein		
m6A	1620862	+		upstream Pnuc_1553, transcriptional regulator, AsnC family		
m4C	1623161	+		upstream Pnuc_1553, transcriptional regulator, AsnC family		
m6A	1625171	+		upstream Pnuc_1553, transcriptional regulator, AsnC family		
m6A	1628517	+	GAYANNNNNNNRTAG	upstream Pnuc_1560, transcriptional regulator, LysR family		
m6A	1628527	-	CTAYNNNNNNNTRTC	Pnuc_1556, Enoyl-CoA hydratase/isomerase		
m6A	1638119	+	CTAYNNNNNNNTRTC	Pnuc_1564, FMN-binding domain protein		
m6A	1638129	-	GAYANNNNNNNRTAG	upstream Pnuc_1563, conserved hypothetical protein		
m6A	1640591	-		upstream Pnuc_1567, Hydroxypyruvate isomerase		
m6A	1647158	+		upstream Pnuc_1581, hypothetical protein		
m6A	1647640	-		Pnuc_1575, anti-ECFsigma factor, ChrR		
m6A	1651758	-		Pnuc_1579, protein tyrosine phosphatase		
m6A	1652331	+		upstream Pnuc_1581, hypothetical protein		
m6A	1654135	-		Pnuc_1583, protein of unknown function DUF344		
m6A	1654835	-		Pnuc_1584, lyso-ornithine lipid acyltransferase		
m6A	1657698	+		Pnuc_1588, ornithine-acyl[acyl carrier protein]N-acyltransferase		
m6A	1658012	+	GAYANNNNNNNRTAG	Pnuc_1588, ornithine-acyl[acyl carrier protein]N-acyltransferase		

m6A	1658022	-	CTAYNNNNNNNNTRTC	upstream Pnuc_1584, lyso-ornithine lipid acyltransferase		
m6A	1660810	+	CTAYNNNNNNNNTRTC	upstream Pnuc_1595, MgtC/SapB transporter		
m6A	1660820	-	GAYANNNNNNNNRTAG	Pnuc_1591, MgtC/SapB transporter		
m6A	1667562	+		upstream Pnuc_1606, short-chain dehydrogenase/reductase SDR		
m4C	1668273	+		upstream Pnuc_1606, short-chain dehydrogenase/reductase SDR		
m4C	1668542	+		upstream Pnuc_1606, short-chain dehydrogenase/reductase SDR		
m6A	1670909	+		upstream Pnuc_1606, short-chain dehydrogenase/reductase SDR		
m4C	1673647	-		Pnuc_1604, conserved hypothetical protein		
m6A	1676774	-		Pnuc_1608, deoxyribodipyrimidine photolyase-related protein		
m6A	1677038	-		Pnuc_1609, conserved hypothetical protein		
m4C	1678665	+		upstream Pnuc_1622, hypothetical protein		
m6A	1690825	-		upstream Pnuc_1621, Mg2+ transporter protein, CorA family protein		
m4C	1691114	+		upstream Pnuc_1627, putative phosphohistidine phosphatase, SixA		
m4C	1697565	+		upstream Pnuc_1636, transcriptional regulator, GntR family		
m4C	1698510	-		Pnuc_1631, Hydroxypyruvate isomerase		
m6A	1699838	+	CTAYNNNNNNNNTRTC	upstream Pnuc_1636, transcriptional regulator, GntR family		
m6A	1699848	-	GAYANNNNNNNNRTAG	Pnuc_1633, type III effector Hrp-dependent outer domain protein		
m6A	1701867	+		upstream Pnuc_1636, transcriptional regulator, GntR family		
m6A	1702881	+		upstream Pnuc_1636, transcriptional regulator, GntR family		
m4C	1705118	-		upstream Pnuc_1637, NAD-dependent epimerase/dehydratase		
m6A	1705954	+	CTAYNNNNNNNNTRTC	Pnuc_1638, protein of unknown function DUF6, transmembrane		
m6A	1705964	-	GAYANNNNNNNNRTAG	upstream Pnuc_1637, NAD-dependent epimerase/dehydratase		
m6A	1709911	-		upstream Pnuc_1641, conserved hypothetical protein		
m6A	1712078	+	CTAYNNNNNNNNTRTC	upstream Pnuc_1649, hypothetical protein		
m6A	1712822	+	CTAYNNNNNNNNTRTC	upstream Pnuc_1649, hypothetical protein		
m6A	1712832	-	GAYANNNNNNNNRTAG	Pnuc_1647, DNA polymerase III, epsilon subunit		
m6A	1722847	+		Pnuc_1660, putative uncharacterized conserved protein		
m4C	1725615	+		upstream Pnuc_1664, spermine/spermidine N-acetyltransferase		
m6A	1726717	+		upstream Pnuc_1664, spermine/spermidine N-acetyltransferase		
m6A	1729661	-		Pnuc_1666, two component transcriptional regulator, winged helix family		
m6A	1730416	-		Pnuc_1667, osmosensitive K+ channel signal transduction histidine kinase		
m4C	1731262	-		Pnuc_1667, osmosensitive K+ channel signal transduction histidine kinase		
m6A	1736729	+		upstream Pnuc_1678, putative transcriptional regulator, MerR family		
m6A	1736959	+	GAYANNNNNNNNRTAG	upstream Pnuc_1678, putative transcriptional regulator, MerR family		
m6A	1736969	-	CTAYNNNNNNNNTRTC	Pnuc_1670, potassium-transporting ATPase, A subunit		
m6A	1737694	-		Pnuc_1670, potassium-transporting ATPase, A subunit		
m4C	1737703	+		upstream Pnuc_1678, putative transcriptional regulator, MerR family		
m6A	1738403	+	CTAYNNNNNNNNTRTC	upstream Pnuc_1678, putative transcriptional regulator, MerR family		
m6A	1738413	-	GAYANNNNNNNNRTAG	Pnuc_1672, Heavy metal transport/detoxification protein		
m6A	1740542	-		Pnuc_1676, conserved secreted protein with internal repeats		
m6A	1741194	-		Pnuc_1677, heavy metal translocating P-type ATPase		
m4C	1748197	+		upstream Pnuc_1688, hypothetical protein		
m6A	1748827	+	GAYANNNNNNNNRTAG	upstream Pnuc_1688, hypothetical protein		
m6A	1748837	-	CTAYNNNNNNNNTRTC	Pnuc_1681, efflux transporter, RND family, MFP subunit		

m4C	1749518	+		upstream Pnuc_1688, hypothetical protein
m6A	1749645	+		upstream Pnuc_1688, hypothetical protein
m6A	1750197	+		upstream Pnuc_1688, hypothetical protein
m6A	1756678	+		Pnuc_1692, hypothetical protein
m6A	1758014	+	GAYANNNNNNNNRTAG	upstream Pnuc_1702, O-sialoglycoprotein endopeptidase
m6A	1758024	-	CTAYNNNNNNNNTRTC	Pnuc_1694, phage integrase family protein
m4C	1761275	+		upstream Pnuc_1702, O-sialoglycoprotein endopeptidase
m4C	1764648	-		Pnuc_1698, RNA polymerase, sigma 70 subunit, RpoD
m6A	1765462	-		upstream Pnuc_1698, RNA polymerase, sigma 70 subunit, RpoD
m6A	1765623	-		upstream Pnuc_1698, RNA polymerase, sigma 70 subunit, RpoD
m6A	1765631	-		upstream Pnuc_1698, RNA polymerase, sigma 70 subunit, RpoD
m4C	1765714	-		Pnuc_1699, DNA primase
m4C	1768466	-		upstream Pnuc_1701, SSU ribosomal protein S21P
m4C	1768677	-		upstream Pnuc_1701, SSU ribosomal protein S21P
m6A	1774025	+		Pnuc_1707, Rieske (2Fe-2S) domain protein
m4C	1774605	-		upstream Pnuc_1706, Exonuclease VII, small subunit
m4C	1776410	+		upstream Pnuc_1712, putative transmembrane protein
m6A	1782114	-		upstream Pnuc_1711, DNA polymerase I
m4C	1782990	-		Pnuc_1715, ATP-dependent DNA helicase UvrD
m4C	1783268	-		Pnuc_1715, ATP-dependent DNA helicase UvrD
m4C	1784851	+		Pnuc_1716, valyl-tRNA synthetase
m6A	1784984	+		Pnuc_1716, valyl-tRNA synthetase
m4C	1786759	-		upstream Pnuc_1715, ATP-dependent DNA helicase UvrD
m6A	1787865	+		Pnuc_1717, UDP-glucose pyrophosphorylase
m6A	1787955	-		upstream Pnuc_1715, ATP-dependent DNA helicase UvrD
m6A	1788038	-		upstream Pnuc_1715, ATP-dependent DNA helicase UvrD
m4C	1788326	-		upstream Pnuc_1715, ATP-dependent DNA helicase UvrD
m4C	1789887	-		Pnuc_1719, alanyl-tRNA synthetase
m6A	1792308	-		upstream Pnuc_1719, alanyl-tRNA synthetase
m6A	1793894	+		Pnuc_1722, glutaminyl-tRNA synthetase
m6A	1797750	+	CTAYNNNNNNNNTRTC	Pnuc_1725, acetylornithine aminotransferase apoenzyme
m6A	1797760	-	GAYANNNNNNNNRTAG	upstream Pnuc_1723, NAD-dependent epimerase/dehydratase
m4C	1799103	+		upstream Pnuc_1733, DNA replication and repair protein RadC
m4C	1799398	-		Pnuc_1726, amino acid/amide ABC transporter ATP-binding protein 2, HAAT family
m6A	1803154	-		Pnuc_1730, amino acid/amide ABC transporter substrate-binding protein, HAAT family
m4C	1805662	-		upstream Pnuc_1732, peptidylprolyl isomerase, FKBP-type
m4C	1806580	+		upstream Pnuc_1739, FMN adenylyltransferase / riboflavin kinase
m6A	1807138	-		Pnuc_1736, fatty acid desaturase
m6A	1807884	-		Pnuc_1737, Fmu (Sun) domain protein
m6A	1808749	-		Pnuc_1737, Fmu (Sun) domain protein
m6A	1808930	-		upstream Pnuc_1737, Fmu (Sun) domain protein
m6A	1809803	+		Pnuc_1739, FMN adenylyltransferase / riboflavin kinase
m6A	1815162	-		upstream Pnuc_1738, formyltetrahydrofolate-dependent phosphoribosylglycinamide formyltransferase
m4C	1815678	-		upstream Pnuc_1738, formyltetrahydrofolate-dependent phosphoribosylglycinamide formyltransferase

m4C	1816159	-		upstream Pnuc_1738, formyltetrahydrofolate-dependent phosphoribosylglycinamide formyltransferase		
m6A	1819565	-		upstream Pnuc_1747, hypothetical protein		
m6A	1821583	-		Pnuc_1748, ATP-dependent Clp protease ATP-binding subunit ClpA		
m4C	1822297	+		upstream Pnuc_1750, cold-shock DNA-binding protein family		
m4C	1825922	-		Pnuc_1754, ornithine carbamoyltransferase		
m6A	1826804	+		upstream Pnuc_1757, integral membrane protein MviN		
m4C	1827004	-		upstream Pnuc_1756, SSU ribosomal protein S20P		
m4C	1827065	-		upstream Pnuc_1756, SSU ribosomal protein S20P		
m4C	1827074	-		upstream Pnuc_1756, SSU ribosomal protein S20P		
m4C	1827283	-		upstream Pnuc_1756, SSU ribosomal protein S20P		
m4C	1827964	+		Pnuc_1757, integral membrane protein MviN		
m6A	1828079	-		upstream Pnuc_1756, SSU ribosomal protein S20P		
m4C	1830997	+		upstream Pnuc_1762, protein of unknown function UPF0118		
m6A	1832665	+	CTAYNNNNNNNNTRTC	Pnuc_1762, protein of unknown function UPF0118		
m6A	1832675	-	GAYANNNNNNNNRTAG	upstream Pnuc_1761, phosphoribosylformylglycinamidine cyclo-ligase		
m4C	1833663	+		Pnuc_1763, regulatory inactivation of DnaA Hda protein		
m6A	1836536	+		Pnuc_1766, 2-amino-4-hydroxy-6-hydroxymethyldihydropteridine pyrophosphokinase		
m4C	1837776	-		Pnuc_1768, chaperone protein DnaJ		
m4C	1837887	-		Pnuc_1768, chaperone protein DnaJ		
m6A	1840618	+		upstream Pnuc_1773, NAD(+) kinase		
m4C	1840727	-		upstream Pnuc_1769, chaperone protein DnaK		
m6A	1843285	+	GAYANNNNNNNNRTAG	upstream Pnuc_1773, NAD(+) kinase		
m6A	1843295	-	CTAYNNNNNNNNTRTC	Pnuc_1772, heat-inducible transcription repressor HrcA		
m6A	1843408	-		Pnuc_1772, heat-inducible transcription repressor HrcA		
m6A	1844781	+		Pnuc_1774, DNA replication and repair protein RecN		
m4C	1846378	+		upstream Pnuc_1776, membrane protein-like protein		
m6A	1850256	+		upstream Pnuc_1776, membrane protein-like protein		
m4C	1850611	-		upstream Pnuc_1775, (Glutamate--ammonia-ligase) adenylyltransferase		
m4C	1852602	-		upstream Pnuc_1775, (Glutamate--ammonia-ligase) adenylyltransferase		
m6A	1853226	+	CTAYNNNNNNNNTRTC	Pnuc_1777, Nitrilase/cyanide hydratase and apolipoprotein N-acyltransferase		
m6A	1853236	-	GAYANNNNNNNNRTAG	upstream Pnuc_1775, (Glutamate-ammonia-ligase) adenylyltransferase		
m4C	1855317	+		Pnuc_1778, microcin-processing peptidase 2, Unknown type peptidase, MEROPS family U62		
m6A	1857619	-		upstream Pnuc_1780, ATP:cob(I)alamin adenosyltransferase		
m4C	1860209	-		upstream Pnuc_1780, ATP:cob(I)alamin adenosyltransferase		
m6A	1861824	+	GAYANNNNNNNNRTAG	Pnuc_1784, alanine racemase domain protein		
m6A	1861834	-	CTAYNNNNNNNNTRTC	upstream Pnuc_1780, ATP:cob(I)alamin adenosyltransferase		
m6A	1862840	-		upstream Pnuc_1780, ATP:cob(I)alamin adenosyltransferase		
m6A	1863024	-		Pnuc_1786, 4-hydroxybenzoate octaprenyltransferase		
m4C	1863093	+		upstream Pnuc_1788, S-adenosylmethionine-tRNA-ribosyltransferase-isomerase		
m6A	1865372	+	GAYANNNNNNNNRTAG	upstream Pnuc_1788, S-adenosylmethionine-tRNA-ribosyltransferase-isomerase		
m6A	1865382	-	CTAYNNNNNNNNTRTC	Pnuc_1787, ATP-dependent DNA helicase RecG		
m4C	1865556	-		Pnuc_1787, ATP-dependent DNA helicase RecG		
m4C	1865946	+		Pnuc_1788, S-adenosylmethionine-tRNA-ribosyltransferase-isomerase		
m6A	1866093	+		Pnuc_1788, S-adenosylmethionine-tRNA-ribosyltransferase-isomerase		

m4C	1867492	-		upstream Pnuc_1787, ATP-dependent DNA helicase RecG
m6A	1867542	+	CTAYNNNNNNNNTRTC	Pnuc_1789, tRNA-guanine transglycosylase
m6A	1867552	-	GAYANNNNNNNNRTAG	upstream Pnuc_1787, ATP-dependent DNA helicase RecG
m6A	1868132	+		Pnuc_1790, protein translocase subunit yajC
m4C	1872268	+		upstream Pnuc_1795, Glutathione S-transferase, N-terminal domain protein
m6A	1874872	+	GAYANNNNNNNNRTAG	upstream Pnuc_1797, alanine dehydrogenase/PNT domain protein
m6A	1874879	+		upstream Pnuc_1797, alanine dehydrogenase/PNT domain protein
m6A	1874882	-	CTAYNNNNNNNNTRTC	Pnuc_1796, tRNA (5-methylaminomethyl-2-thiouridylate)-methyltransferase
m4C	1875999	+		upstream Pnuc_1797, alanine dehydrogenase/PNT domain protein
m6A	1876112	+		upstream Pnuc_1797, alanine dehydrogenase/PNT domain protein
m4C	1878427	+		Pnuc_1799, NAD(P) transhydrogenase, beta subunit
m6A	1879092	-		upstream Pnuc_1796, tRNA (5-methylaminomethyl-2-thiouridylate)-methyltransferase
m6A	1880808	+	GAYANNNNNNNNRTAG	upstream Pnuc_1802, hypothetical protein
m6A	1880818	-	CTAYNNNNNNNNTRTC	Pnuc_1801, glycosyl transferase, family 11
m6A	1881762	+	CTAYNNNNNNNNTRTC	Pnuc_1802, hypothetical protein
m6A	1881772	-	GAYANNNNNNNNRTAG	upstream Pnuc_1801, glycosyl transferase, family 11
m6A	1882788	-		Pnuc_1804, conserved hypothetical protein
m6A	1884638	-		Pnuc_1805, chaperonin GroEL
m6A	1887649	+		Pnuc_1809, amino acid-binding domain sensor hybrid histidine kinase
m6A	1887784	+		Pnuc_1809, amino acid-binding domain sensor hybrid histidine kinase
m6A	1892173	-		Pnuc_1811, translation factor SUA5
m6A	1894654	-		Pnuc_1814, phosphoribosylaminoimidazole-succinocarboxamide synthase
m6A	1894804	+	GAYANNNNNNNNRTAG	upstream Pnuc_1818, branched chain amino acid aminotransferase apoenzyme
m6A	1894814	-	CTAYNNNNNNNNTRTC	Pnuc_1814, phosphoribosylaminoimidazole-succinocarboxamide synthase
m4C	1895131	-		Pnuc_1815, fructose-bisphosphate aldolase
m4C	1895880	+		upstream Pnuc_1818, branched chain amino acid aminotransferase apoenzyme
m4C	1895881	+		upstream Pnuc_1818, branched chain amino acid aminotransferase apoenzyme
m4C	1896166	+		upstream Pnuc_1818, branched chain amino acid aminotransferase apoenzyme
m6A	1897475	-		Pnuc_1817, phosphoglycerate kinase
m6A	1899165	+	CTAYNNNNNNNNTRTC	Pnuc_1818, branched chain amino acid aminotransferase apoenzyme
m6A	1899175	-	GAYANNNNNNNNRTAG	upstream Pnuc_1817, phosphoglycerate kinase
m6A	1902175	-		upstream Pnuc_1817, phosphoglycerate kinase
m4C	1903822	+		upstream Pnuc_1824, GTP cyclohydrolase subunit MoaC
m6A	1904068	+		Pnuc_1824, GTP cyclohydrolase subunit MoaC
m4C	1914844	+		upstream Pnuc_1835, hypothetical protein
m6A	1914998	+		upstream Pnuc_1835, hypothetical protein
m6A	1916434	+	GAYANNNNNNNNRTAG	Pnuc_1835, hypothetical protein
m6A	1916444	-	CTAYNNNNNNNNTRTC	upstream Pnuc_1834, conserved hypothetical protein
m6A	1918050	+	GAYANNNNNNNNRTAG	Pnuc_1838, hypothetical protein
m6A	1918060	-	CTAYNNNNNNNNTRTC	upstream Pnuc_1834, conserved hypothetical protein
m6A	1921973	+	GAYANNNNNNNNRTAG	upstream Pnuc_1847, secreted protein
m6A	1921983	-	CTAYNNNNNNNNTRTC	upstream Pnuc_1843, hypothetical protein
m6A	1923748	+		upstream Pnuc_1847, secreted protein
m6A	1924410	+		upstream Pnuc_1847, secreted protein

m4C	1927470	+		upstream Pnuc_1851, maleylacetoacetate isomerase		
m6A	1929095	-		Pnuc_1852, transferase hexapeptide repeat protein		
m6A	1931420	-		upstream Pnuc_1857, Rhodanese domain protein		
m6A	1934625	-		upstream Pnuc_1857, Rhodanese domain protein		
m6A	1935097	+	GAYANNNNNNNRTAG	Pnuc_1861, CBS domain containing protein		
m6A	1935107	-	CTAYNNNNNNNTRTC	upstream Pnuc_1857, Rhodanese domain protein		
m6A	1936096	-		upstream Pnuc_1857, Rhodanese domain protein		
m6A	1936488	-		upstream Pnuc_1857, Rhodanese domain protein		
m6A	1936868	-		upstream Pnuc_1857, Rhodanese domain protein		
m6A	1937416	+		Pnuc_1863, glycyl-tRNA synthetase alpha chain		
m4C	1937691	+		Pnuc_1863, glycyl-tRNA synthetase alpha chain		
m6A	1940032	+	CTAYNNNNNNNTRTC	Pnuc_1865, D-alpha,beta-D-heptose 1,7-bisphosphate phosphatase		
m6A	1940042	-	GAYANNNNNNNRTAG	upstream Pnuc_1857, Rhodanese domain protein		
m6A	1943461	+		upstream Pnuc_1871, aminoglycoside phosphotransferase		
m6A	1954638	-		upstream Pnuc_1870, Organic solvent tolerance protein		
m6A	1954913	-		upstream Pnuc_1870, Organic solvent tolerance protein		
m6A	1956898	-		Pnuc_1881, tyrosyl-tRNA synthetase		
m4C	1962595	-		upstream Pnuc_R0042, RNase P		
m6A	1968003	+	CTAYNNNNNNNTRTC	upstream Pnuc_1900, L-threonine ammonia-lyase		
m6A	1968013	-	GAYANNNNNNNRTAG	Pnuc_1892, aspartyl-tRNA synthetase		
m6A	1968185	+	GAYANNNNNNNRTAG	upstream Pnuc_1900, L-threonine ammonia-lyase		
m6A	1968195	-	CTAYNNNNNNNTRTC	Pnuc_1892, aspartyl-tRNA synthetase		
m4C	1968481	+		upstream Pnuc_1900, L-threonine ammonia-lyase		
m6A	1968822	+		upstream Pnuc_1900, L-threonine ammonia-lyase		
m4C	1969703	+		upstream Pnuc_1900, L-threonine ammonia-lyase		
m6A	1974979	+	GAYANNNNNNNRTAG	upstream Pnuc_1900, L-threonine ammonia-lyase		
m6A	1974989	-	CTAYNNNNNNNTRTC	Pnuc_1899, FAD linked oxidase domain protein		
m6A	1981269	-		upstream Pnuc_1899, FAD linked oxidase domain protein		
m4C	1982013	-		Pnuc_1903, single-strand binding protein		
m6A	1982693	+		upstream Pnuc_1905, Excinuclease ABC subunit A		
m4C	1983946	-		upstream Pnuc_1904, major facilitator superfamily MFS_1		
m6A	1985406	-		upstream Pnuc_1904, major facilitator superfamily MFS_2		
m6A	1988342	+		Pnuc_1907, KpsF/GutQ family protein		
m4C	1990900	-		upstream Pnuc_1906, Kef-type potassium/proton antiporter, CPA2 family		
m4C	1994023	-		upstream Pnuc_1906, Kef-type potassium/proton antiporter, CPA2 family		
m6A	1994031	+		Pnuc_1915, Uncharacterized P-loop ATPase protein UPF0042		
m4C	1998579	-		upstream Pnuc_1917, DNA-(apurinic or apyrimidinic site) lyase		
m6A	1999753	+	GAYANNNNNNNRTAG	Pnuc_1920, ribose-phosphate pyrophosphokinase		
m6A	1999763	-	CTAYNNNNNNNTRTC	upstream Pnuc_1917, DNA-(apurinic or apyrimidinic site) lyase		
m4C	1999808	+		Pnuc_1920, ribose-phosphate pyrophosphokinase		
m4C	2000189	+		upstream Pnuc_1921, LSU ribosomal protein L25P		
m6A	2000287	-		upstream Pnuc_1917, DNA-(apurinic or apyrimidinic site) lyase		
m6A	2000847	+		Pnuc_1921, LSU ribosomal protein L25P		
m4C	2002810	-		Pnuc_1925, putative methyltransferase		

m4C	2004211	-		Pnuc_1926, peptidase M16 domain protein		
m6A	2006000	+		Pnuc_1928, signal recognition particle-docking protein FtsY		
m6A	2008414	+	GAYANNNNNNNRTAG	upstream Pnuc_1935, conserved hypothetical protein		
m6A	2008424	-	CTAYNNNNNNNTRTC	Pnuc_1931, protoheme IX farnesyltransferase		
m6A	2008780	+		upstream Pnuc_1935, conserved hypothetical protein		
m6A	2010428	-		Pnuc_1933, putative transmembrane protein		
m6A	2012994	-		upstream Pnuc_1936, cytochrome c oxidase, subunit III		
m4C	2013806	-		upstream Pnuc_1938, cytochrome c oxidase assembly protein CtaG/Cox11		
m6A	2014025	-		Pnuc_1939, Cytochrome-c oxidase		
m6A	2014353	-		Pnuc_1939, Cytochrome-c oxidase		
m6A	2014371	+	CTAYNNNNNNNTRTC	upstream Pnuc_1942, phosphoribosyltransferase		
m6A	2014381	-	GAYANNNNNNNRTAG	Pnuc_1939, Cytochrome-c oxidase		
m6A	2014641	+	GAYANNNNNNNRTAG	upstream Pnuc_1942, phosphoribosyltransferase		
m6A	2014651	-	CTAYNNNNNNNTRTC	Pnuc_1939, Cytochrome-c oxidase		
m6A	2019976	+		upstream Pnuc_1948, phosphoglycerate mutase		
m4C	2020099	+		upstream Pnuc_1948, phosphoglycerate mutase		
m4C	2020195	+		upstream Pnuc_1948, phosphoglycerate mutase		
m6A	2021719	+		Pnuc_1948, phosphoglycerate mutase		
m4C	2023023	+		Pnuc_1949, carboxyl-terminal protease		
m4C	2023875	+		Pnuc_1950, [sulfur carrier protein ThiS]adenylyltransferase		
m6A	2035852	+		upstream Pnuc_1967, biotin--acetyl-CoA-carboxylase ligase		
m4C	2039264	-		upstream Pnuc_1969, rfaE bifunctional protein		
m4C	2040106	-		upstream Pnuc_1969, rfaE bifunctional protein		
m4C	2045187	+		upstream Pnuc_1978, conserved hypothetical protein		
m4C	2046520	+		upstream Pnuc_1978, conserved hypothetical protein		
m6A	2050688	+		upstream Pnuc_1981		
m6A	2054081	-		upstream Pnuc_1984, Alcohol dehydrogenase, zinc-binding domain protein		
m6A	2054201	+	CTAYNNNNNNNTRTC	Pnuc_1985, hypothetical protein		
m4C	2055912	+		upstream Pnuc_1996, protein of unknown function DUF185		
m4C	2057298	-		Pnuc_1988, acriflavin resistance protein		
m4C	2060192	-		Pnuc_1990, RND efflux system, outer membrane lipoprotein, NodT family		
m4C	2060483	+		upstream Pnuc_1996, protein of unknown function DUF185		
m6A	2063777	+	CTAYNNNNNNNTRTC	upstream Pnuc_1996, protein of unknown function DUF185		
m6A	2063787	-	GAYANNNNNNNRTAG	Pnuc_1992, UDP-N-acetylglucosamine pyrophosphorylase / glucosamine-1-phosphate N-acetyltransferase		
m6A	2066312	+	GAYANNNNNNNRTAG	upstream Pnuc_1996, protein of unknown function DUF185		
m6A	2066322	-	CTAYNNNNNNNTRTC	Pnuc_1994, dihydroneopterin aldolase		
m6A	2072803	-		upstream Pnuc_1999, Lytic transglycosylase, catalytic		
m6A	2074411	-		Pnuc_2002, adenosylhomocysteinase		
m6A	2077839	-		upstream Pnuc_2003, methionine adenosyltransferase		
m4C	2081025	+		Pnuc_2008, exodeoxyribonuclease III Xth		
m6A	2081485	+		upstream Pnuc_2021, rod shape-determining protein MreB		
m6A	2082597	+		upstream Pnuc_2021, rod shape-determining protein MreB		
m6A	2085911	-		upstream Pnuc_2013, N-acetylglutamate kinase		
m6A	2085972	+	GAYANNNNNNNRTAG	upstream Pnuc_2021, rod shape-determining protein MreB		

m6A	2085982	-	CTAYNNNNNNNTRTC	Pnuc_2014, transcriptional regulator, TraR/DksA family		
m4C	2089737	+		upstream Pnuc_2021, rod shape-determining protein MreB		
m4C	2091198	-		Pnuc_2019, aspartyl/glutamyl-tRNA(Asn/Gln) amidotransferase subunit A		
m4C	2092910	+		Pnuc_2021, rod shape-determining protein MreB		
m6A	2093215	-		upstream Pnuc_2020, aspartyl/glutamyl-tRNA(Asn/Gln) amidotransferase subunit C		
m4C	2094451	+		Pnuc_2022, rod shape-determining protein MreC		
m4C	2094517	+		Pnuc_2022, rod shape-determining protein MreC		
m4C	2095554	+		Pnuc_2024, cell elongation-specific peptidoglycan D,D-transpeptidase		
m6A	2098007	+		Pnuc_2025, cell elongation-specific peptidoglycan biosynthesis regulator RodA		
m6A	2098082	-		upstream Pnuc_2020, aspartyl/glutamyl-tRNA(Asn/Gln) amidotransferase subunit C		
m4C	2098226	+		Pnuc_2025, cell elongation-specific peptidoglycan biosynthesis regulator RodA		
m4C	2098267	+		upstream Pnuc_2028, Aspartyl/Asparaginyl beta-hydroxylase		
m6A	2100138	-		upstream Pnuc_2027, conserved hypothetical protein		
m6A	2100782	-		upstream Pnuc_2029, protein of unknown function DUF167		
m4C	2101468	+		Pnuc_2031, lipid A biosynthesis acyltransferase		
m6A	2102026	+	CTAYNNNNNNNTRTC	Pnuc_2031, lipid A biosynthesis acyltransferase		
m6A	2102036	-	GAYANNNNNNNRTAG	upstream Pnuc_2029, protein of unknown function DUF167		
m4C	2102300	-		upstream Pnuc_2029, protein of unknown function DUF167		
m6A	2104651	+		upstream Pnuc_2035, ribosomal large subunit pseudouridine synthase F		
m4C	2108072	-		upstream Pnuc_2034, sodium:dicarboxylate symporter		
m4C	2108776	+		upstream Pnuc_2038, GCN5-related N-acetyltransferase		
m6A	2109837	-		upstream Pnuc_2037, conserved hypothetical protein		
m6A	2110796	+		Pnuc_2040, MltA-interacting MipA family protein		
m4C	2118082	+		upstream Pnuc_2056, protein of unknown function DUF897		
m6A	2118474	+	GAYANNNNNNNRTAG	upstream Pnuc_2056, protein of unknown function DUF897		
m6A	2118484	-	CTAYNNNNNNNTRTC	Pnuc_2049, hypothetical protein		
m6A	2121455	+		upstream Pnuc_2056, protein of unknown function DUF897		
m6A	2122486	-		Pnuc_2054, CHRD domain containing protein		
m6A	2127286	-		Pnuc_2060, Patatin		
m4C	2128344	-		Pnuc_2060, Patatin		
m4C	2128565	+		upstream Pnuc_2062, hypothetical protein		
m6A	2128606	+		upstream Pnuc_2062, hypothetical protein		
m6A	2129286	-		upstream Pnuc_2060, Patatin		
m6A	2133944	+	GAYANNNNNNNRTAG	Pnuc_2065, hypothetical protein		
m6A	2133954	-	CTAYNNNNNNNTRTC	upstream Pnuc_2064, hypothetical protein		
m6A	2141211	-		Pnuc_2073, protein of unknown function DUF181		
m6A	2141500	+		upstream Pnuc_2080, DNA protecting protein DprA		
m6A	2141510	-		Pnuc_2073, protein of unknown function DUF181		
m6A	2144251	+		upstream Pnuc_2080, DNA protecting protein DprA		
m6A	2145421	-		Pnuc_2076, putative periplasmic ligand-binding sensor protein		
m6A	2146256	+	CTAYNNNNNNNTRTC	upstream Pnuc_2080, DNA protecting protein DprA		
m6A	2146266	-	GAYANNNNNNNRTAG	Pnuc_2077, sun protein		
m4C	2149663	-		upstream Pnuc_2079, peptide deformylase		
m4C	2151298	+		Pnuc_2081, DNA topoisomerase III		

m4C	2152087	-		upstream Pnuc_2079, peptide deformylase	
m4C	2155660	-		Pnuc_2084, tRNA modification GTPase trmE	

26°C					
**Methylation**	**Location**	**Strand**	**Motif**	**Gene / Intergenic region**	**Count**
m6A	901	-		upstream Pnuc_2088, LSU ribosomal protein L34P	769(+)
m4C	1132	+		Pnuc_0001,chromosomal replication initiator protein DnaA	735(-)
m4C	6952	+		Pnuc_0007, amino acid/amide ABC transporter ATP-binding protein 2, HAAT family	**Total** = 1504 (m6A + m4C)
m4C	7696	-		upstream Pnuc_2088, LSU ribosomal protein L34P	
m4C	14758	+		Pnuc_0015, glucose inhibited division protein A	341(+)
m4C	19205	-		upstream Pnuc_0013, acyl-CoA dehydrogenase domain protein	343(-)
m4C	22089	-		upstream Pnuc_0013, acyl-CoA dehydrogenase domain protein	**Total** = 684 (m6A)
m4C	22150	-		upstream Pnuc_0013, acyl-CoA dehydrogenase domain protein	
m4C	26361	+		Pnuc_0028, uroporphyrinogen decarboxylase	428(+)
m6A	26488	+		Pnuc_0028, uroporphyrinogen decarboxylase	392(-)
m6A	28723	+		Pnuc_0029, replication restart DNA helicase PriA	**Total** = 820 (m4C)
m4C	31637	-		Pnuc_0032, fatty acid desaturase	
m6A	32663	+		Pnuc_0033, cytochrome c, class I	Methylated genes = 512 (24.52%)
m4C	33233	+		upstream Pnuc_0035, phosphoheptose isomerase	
m6A	35133	+		Pnuc_0036, transport-associated protein	
m4C	37437	-		upstream Pnuc_0038, GCN5-related N-acetyltransferase	
m6A	37807	+	CTAYNNNNNNNTRTC	Pnuc_R0002, 16S ribosomal RNA	
m6A	37817	-	GAYANNNNNNNRTAG	upstream Pnuc_0038, GCN5-related N-acetyltransferase	
m4C	39095	-		upstream Pnuc_0038, GCN5-related N-acetyltransferase	
m4C	40789	-		upstream Pnuc_0038, GCN5-related N-acetyltransferase	
m4C	44092	+		Pnuc_0041, transcription antitermination protein nusG	
m4C	44677	-		upstream Pnuc_0038, GCN5-related N-acetyltransferase	
m4C	47634	+		Pnuc_0046, DNA-directed RNA polymerase subunit beta	
m4C	47980	-		upstream Pnuc_0038, GCN5-related N-acetyltransferase	
m4C	47982	-		upstream Pnuc_0038, GCN5-related N-acetyltransferase	
m4C	49472	-		upstream Pnuc_0038, GCN5-related N-acetyltransferase	
m4C	52984	+		Pnuc_0047, DNA-directed RNA polymerase subunit beta	
m4C	53025	+		Pnuc_0047, DNA-directed RNA polymerase subunit beta	
m4C	54887	+		Pnuc_0047, DNA-directed RNA polymerase subunit beta	
m4C	57126	-		upstream Pnuc_0038, GCN5-related N-acetyltransferase	
m4C	57143	-		upstream Pnuc_0038, GCN5-related N-acetyltransferase	
m4C	58975	+		Pnuc_0050, translation elongation factor 2 (EF-2/EF-G)	
m4C	59477	-		upstream Pnuc_0038, GCN5-related N-acetyltransferase	
m4C	64043	-		upstream Pnuc_0038, GCN5-related N-acetyltransferase	
m4C	65373	+		Pnuc_0061, LSU ribosomal protein L29P	
m4C	65930	-		upstream Pnuc_0038, GCN5-related N-acetyltransferase	
m4C	65949	-		upstream Pnuc_0038, GCN5-related N-acetyltransferase	
m4C	66764	+		Pnuc_0065, LSU ribosomal protein L5P	

m4C	68018	+		Pnuc_0068, LSU ribosomal protein L6P
m6A	68025	+		Pnuc_0068, LSU ribosomal protein L6P
m4C	70249	+		Pnuc_0073, protein translocase subunit secY/sec61 alpha
m4C	71180	-		upstream Pnuc_0038, GCN5-related N-acetyltransferase
m4C	71592	-		upstream Pnuc_0038, GCN5-related N-acetyltransferase
m4C	76642	+		Pnuc_0081, Protein-disulfide reductase
m4C	76864	-		upstream Pnuc_0038, GCN5-related N-acetyltransferase
m4C	80624	-		Pnuc_0086, diaminopimelate decarboxylase
m6A	82350	-		Pnuc_0088, 3-dehydroquinate synthase
m6A	83003	+	GAYANNNNNNNRTAG	upstream Pnuc_0092, penicillin-binding protein, 1A family
m6A	83013	-	CTAYNNNNNNNTRTC	Pnuc_0088, 3-dehydroquinate synthase
m6A	83216	+		upstream Pnuc_0092, penicillin-binding protein, 1A family
m6A	83832	-		Pnuc_0090, type II and III secretion system protein
m6A	84386	+	CTAYNNNNNNNTRTC	upstream Pnuc_0092, penicillin-binding protein, 1A family
m6A	84396	-	GAYANNNNNNNRTAG	Pnuc_0090, type II and III secretion system protein
m4C	84866	+		upstream Pnuc_0092, penicillin-binding protein, 1A family
m6A	85182	-		Pnuc_0091, hypothetical protein
m6A	87597	+		Pnuc_0092, penicillin-binding protein, 1A family
m4C	88649	-		upstream Pnuc_0091, hypothetical protein
m4C	90112	+		Pnuc_0094, glutamate synthase (NADH) large subunit
m4C	96037	+		Pnuc_0097, protein of unknown function DUF140
m4C	96490	+		Pnuc_0097, protein of unknown function DUF140
m4C	96752	-		upstream Pnuc_0091, hypothetical protein
m4C	98127	+		Pnuc_0100, toluene tolerance family protein
m4C	100046	+		Pnuc_0103, ABC-2 type transporter
m4C	100149	-		upstream Pnuc_0091, hypothetical protein
m6A	102779	+	GAYANNNNNNNRTAG	Pnuc_0107, histidinol dehydrogenase
m6A	102789	-	CTAYNNNNNNNTRTC	upstream Pnuc_0091, hypothetical protein
m6A	103179	+	CTAYNNNNNNNTRTC	Pnuc_0107, histidinol dehydrogenase
m6A	103189	-	GAYANNNNNNNRTAG	upstream Pnuc_0091, hypothetical protein
m6A	105053	-		Pnuc_0108, histidinol phosphate aminotransferase apoenzyme
m4C	112062	+		upstream Pnuc_0120, porin, Gram-negative type
m6A	112153	-		Pnuc_0119, pseudo
m4C	112352	-		Pnuc_0119, pseudo
m4C	112487	+		upstream Pnuc_0120, porin, Gram-negative type
m4C	112495	-		upstream Pnuc_0119, pseudo
m6A	113017	-		upstream Pnuc_0119, pseudo
m4C	113636	-		upstream Pnuc_0119, pseudo
m4C	114280	+		Pnuc_0121, TPR repeat-containing protein
m4C	115403	-		upstream Pnuc_0119, pseudo
m4C	117662	-		Pnuc_0122, Tetratricopeptide TPR_2 repeat protein
m4C	117690	+		upstream Pnuc_0125, ubiquinol-cytochrome c reductase, iron-sulfur subunit
m4C	119521	+		upstream Pnuc_0125, ubiquinol-cytochrome c reductase, iron-sulfur subunit
m6A	119660	+		upstream Pnuc_0125, ubiquinol-cytochrome c reductase, iron-sulfur subunit

m6A	122705	+	CTAYNNNNNNNTRTC	Pnuc_0127, cytochrome c1		
m6A	122715	-	GAYANNNNNNNRTAG	upstream Pnuc_0124, large conductance mechanosensitive channel protein		
m4C	128274	-		Pnuc_0134, glutaredoxin-like protein		
m4C	128600	+		upstream Pnuc_0142, GTP-binding protein YchF		
m6A	130121	+	GAYANNNNNNNRTAG	upstream Pnuc_0142, GTP-binding protein YchF		
m6A	130131	-	CTAYNNNNNNNTRTC	Pnuc_0136, bacterial peptide chain release factor 1(bRF-1)		
m4C	130852	+		upstream Pnuc_0142, GTP-binding protein YchF		
m6A	131767	+		upstream Pnuc_0142, GTP-binding protein YchF		
m4C	134617	+		upstream Pnuc_0142, GTP-binding protein YchF		
m4C	135742	+		upstream Pnuc_0142, GTP-binding protein YchF		
m4C	136496	-		Pnuc_0141, Ubiquinone biosynthesis hydroxylase, UbiH/UbiF/VisC/COQ6 family		
m6A	137333	+		Pnuc_0142, GTP-binding protein YchF		
m4C	137852	+		Pnuc_0142, GTP-binding protein YchF		
m6A	139555	-		Pnuc_0145, indole-3-glycerol phosphate synthase		
m4C	142160	+		upstream Pnuc_0150, ApaG domain protein		
m4C	144075	-		upstream Pnuc_0149, ribulose-5-phosphate 3-epimerase		
m6A	149067	+	CTAYNNNNNNNTRTC	upstream Pnuc_0156, biotin synthase		
m6A	149077	-	GAYANNNNNNNRTAG	Pnuc_0155, aminotransferase		
m4C	149940	+		Pnuc_0156, biotin synthase		
m4C	151549	-		upstream Pnuc_0155, aminotransferase		
m4C	154698	+		Pnuc_0161, peptidoglycan synthetase FtsI		
m6A	157261	-		upstream Pnuc_0155, aminotransferase		
m6A	157993	+	GAYANNNNNNNRTAG	Pnuc_0163, UDP-N-acetylmuramoylalanyl-D-glutamyl-2,6-diaminopimelate--D-alanyl-D-alanyl ligase		
m6A	158003	-	CTAYNNNNNNNTRTC	upstream Pnuc_0155, aminotransferase		
m4C	160218	-		upstream Pnuc_0155, aminotransferase		
m6A	163647	+		Pnuc_0168, UDP-N-acetylmuramate--L-alanine ligase		
m4C	165541	+		Pnuc_0169, D-alanine--D-alanine ligase		
m4C	165703	-		upstream Pnuc_0155, aminotransferase		
m4C	165797	-		upstream Pnuc_0155, aminotransferase		
m4C	168341	-		upstream Pnuc_0155, aminotransferase		
m6A	171550	+		Pnuc_0176, protein translocase subunit secA		
m4C	173940	-		upstream Pnuc_0175, hypothetical protein		
m6A	174286	+		upstream Pnuc_0177, (2Fe-2S)-binding domain protein		
m4C	175181	-		upstream Pnuc_0175, hypothetical protein		
m4C	175458	-		upstream Pnuc_0175, hypothetical protein		
m6A	175924	-		upstream Pnuc_0175, hypothetical protein		
m4C	176761	-		upstream Pnuc_0175, hypothetical protein		
m4C	177012	+		Pnuc_0178, aldehyde oxidase and xanthine dehydrogenase,molybdopterin binding protein		
m6A	177242	-		upstream Pnuc_0175, hypothetical protein		
m4C	179707	-		Pnuc_0182, protein of unknown function DUF1342		
m4C	179903	-		Pnuc_0182, protein of unknown function DUF1342		
m4C	180511	-		Pnuc_0183, dephospho-CoA kinase		
m4C	181009	+		upstream Pnuc_0188, methylated-DNA--protein-cysteine methyltransferase		
m4C	181048	+		upstream Pnuc_0188, methylated-DNA--protein-cysteine methyltransferase		

m4C	182559	-		Pnuc_0185, type II secretion system protein		
m6A	183048	+		upstream Pnuc_0188, methylated-DNA--protein-cysteine methyltransferase		
m4C	188018	+		Pnuc_0190, Na+/solute symporter		
m6A	190987	-		Pnuc_0193, Farnesyltranstransferase		
m4C	191212	+		Pnuc_0194, LSU ribosomal protein L21P		
m4C	193735	+		Pnuc_0197, glutamate 5-kinase		
m4C	196593	-		upstream Pnuc_0199, NUDIX hydrolase		
m4C	197657	+		upstream Pnuc_0202, cytochrome c assembly protein		
m6A	199926	+		upstream Pnuc_0204, ribonucleoside-diphosphate reductase, alpha subunit		
m6A	200409	-		upstream Pnuc_0201, signal recognition particle subunit FFH/SRP54 (srp54)		
m6A	200762	+	CTAYNNNNNNNNTRTC	Pnuc_0204, ribonucleoside-diphosphate reductase, alpha subunit		
m6A	200772	-	GAYANNNNNNNNRTAG	upstream Pnuc_0201, signal recognition particle subunit FFH/SRP54 (srp54)		
m4C	201940	-		upstream Pnuc_0201, signal recognition particle subunit FFH/SRP54 (srp54)		
m4C	202708	-		upstream Pnuc_0201, signal recognition particle subunit FFH/SRP54 (srp54)		
m4C	202934	+		Pnuc_0204, ribonucleoside-diphosphate reductase, alpha subunit		
m6A	203706	-		upstream Pnuc_0201, signal recognition particle subunit FFH/SRP54 (srp54)		
m4C	204062	-		upstream Pnuc_0201, signal recognition particle subunit FFH/SRP54 (srp54)		
m4C	205116	-		upstream Pnuc_0201, signal recognition particle subunit FFH/SRP54 (srp54)		
m4C	205165	+		upstream Pnuc_0213, UDP-N-acetylmuramate		
m6A	208466	+		upstream Pnuc_0213, UDP-N-acetylmuramate		
m6A	209668	+	GAYANNNNNNNNRTAG	upstream Pnuc_0213, UDP-N-acetylmuramate		
m6A	209678	-	CTAYNNNNNNNNTRTC	Pnuc_0211, 3-dehydroquinate dehydratase		
m6A	210232	+		upstream Pnuc_0213, UDP-N-acetylmuramate		
m4C	211553	+		Pnuc_0213, UDP-N-acetylmuramate		
m6A	216842	-		upstream Pnuc_0212, alkyl hydroperoxide reductase/ Thiol specific antioxidant/ Mal allergen		
m4C	217176	-		upstream Pnuc_0212, alkyl hydroperoxide reductase/ Thiol specific antioxidant/ Mal allergen		
m4C	218942	+		Pnuc_0220, magnesium and cobalt transport protein CorA		
m4C	219189	+		Pnuc_0220, magnesium and cobalt transport protein CorA		
m4C	221772	-		upstream Pnuc_0223, thiamine-phosphate kinase		
m4C	224128	-		upstream Pnuc_0223, thiamine-phosphate kinase		
m4C	224476	-		upstream Pnuc_0223, thiamine-phosphate kinase		
m4C	226644	+		Pnuc_0227, transketolase		
m4C	227034	-		upstream Pnuc_0226, protein of unknown function DUF558		
m6A	227673	+	GAYANNNNNNNNRTAG	Pnuc_0228, glyceraldehyde-3-phosphate dehydrogenase		
m6A	227683	-	CTAYNNNNNNNNTRTC	upstream Pnuc_0226, protein of unknown function DUF558		
m6A	228520	+		Pnuc_0228, glyceraldehyde-3-phosphate dehydrogenase		
m6A	228733	-		Pnuc_0229, ferric uptake regulator, Fur family		
m4C	230196	+		Pnuc_0231, dihydrodipicolinate reductase		
m6A	236488	+		Pnuc_0235, glutamate-5-semialdehyde dehydrogenase		
m4C	236619	-		upstream Pnuc_0229, ferric uptake regulator, Fur family		
m6A	238728	+		Pnuc_0239, glutamate-1-semialdehyde 2,1-aminomutase		
m4C	238745	-		upstream Pnuc_0229, ferric uptake regulator, Fur family		
m4C	239533	+		Pnuc_0239, glutamate-1-semialdehyde 2,1-aminomutase		
m4C	241062	+		upstream Pnuc_0242, protein of unknown function DUF218		

m4C	241500	+		upstream Pnuc_0242, protein of unknown function DUF218		
m4C	243937	+		Pnuc_0244, Holliday junction resolvase YqgF		
m4C	243991	+		Pnuc_0244, Holliday junction resolvase YqgF		
m4C	244876	-		upstream Pnuc_0241, deoxyribodipyrimidine photo-lyase type I		
m6A	246363	-		upstream Pnuc_0241, deoxyribodipyrimidine photo-lyase type I		
m6A	246364	-		upstream Pnuc_0241, deoxyribodipyrimidine photo-lyase type I		
m4C	247712	-		upstream Pnuc_0241, deoxyribodipyrimidine photo-lyase type I		
m6A	248439	-	GAYANNNNNNNRTAG	upstream Pnuc_0241, deoxyribodipyrimidine photo-lyase type I		
m4C	250708	-		Pnuc_0251, hypothetical protein		
m6A	250899	+	CTAYNNNNNNNTRTC	Pnuc_0252, hypothetical protein		
m6A	250909	-	GAYANNNNNNNRTAG	upstream Pnuc_0251, hypothetical protein		
m6A	251656	+	GAYANNNNNNNRTAG	upstream Pnuc_0253, dTDP-glucose 4,6-dehydratase		
m6A	251666	-	CTAYNNNNNNNTRTC	upstream Pnuc_0251, hypothetical protein		
m4C	255335	-		upstream Pnuc_0251, hypothetical protein		
m4C	256362	-		Pnuc_0258, type I secretion outer membrane protein, TolC family		
m4C	256533	+		upstream Pnuc_0259, Undecaprenyl-diphosphatase		
m4C	257450	-		Pnuc_0258, type I secretion outer membrane protein, TolC family		
m4C	258503	+		Pnuc_0259, Undecaprenyl-diphosphatase		
m4C	262450	+		upstream Pnuc_0267, hypothetical protein		
m6A	264991	+		upstream Pnuc_0267, hypothetical protein		
m4C	268392	-		upstream Pnuc_0266, 3,4-dihydroxy-2-butanone 4-phosphate synthase		
m4C	269161	-		Pnuc_0272, preQ(0) biosynthesis protein QueC		
m6A	269370	+	GAYANNNNNNNRTAG	upstream Pnuc_0279, serine hydroxymethyltransferase		
m6A	269380	-	CTAYNNNNNNNTRTC	Pnuc_0272 (start codon), preQ(0) biosynthesis protein QueC		
m4C	270060	+		upstream Pnuc_0279, serine hydroxymethyltransferase		
m4C	270570	+		upstream Pnuc_0279, serine hydroxymethyltransferase		
m6A	271023	-	CTAYNNNNNNNTRTC	Pnuc_0275, TolB, N-terminal domain protein		
m4C	271970	+		upstream Pnuc_0279, serine hydroxymethyltransferase		
m4C	272119	-		Pnuc_0276, Cell division and transport-associated protein TolA		
m6A	277009	+	CTAYNNNNNNNTRTC	upstream Pnuc_0287, Exodeoxyribonuclease VII large subunit		
m6A	277019	-	GAYANNNNNNNRTAG	Pnuc_0282, 3-deoxy-D-manno-octulosonate cytidylyltransferase		
m6A	277383	+		upstream Pnuc_0287, Exodeoxyribonuclease VII large subunit		
m4C	279494	+		upstream Pnuc_0287, Exodeoxyribonuclease VII large subunit		
m4C	280973	-		upstream Pnuc_0286, MotA/TolQ/ExbB proton channel		
m4C	281999	+		Pnuc_0288, conserved hypothetical protein		
m4C	282108	-		upstream Pnuc_0286, MotA/TolQ/ExbB proton channel		
m6A	283189	+	GAYANNNNNNNRTAG	upstream Pnuc_0290, protein of unknown function DUF520		
m6A	283199	-	CTAYNNNNNNNTRTC	Pnuc_0289, UDP-N-acetylmuramate dehydrogenase		
m4C	283507	+		upstream Pnuc_0290, protein of unknown function DUF520		
m4C	284893	-		upstream Pnuc_0289, UDP-N-acetylmuramate dehydrogenase		
m4C	285687	-		upstream Pnuc_0289, UDP-N-acetylmuramate dehydrogenase		
m4C	295143	-		upstream Pnuc_0300, hypothetical protein		
m4C	300803	+		upstream Pnuc_0311, polysaccharide biosynthesis protein		
m4C	301640	+		upstream Pnuc_0311, polysaccharide biosynthesis protein		

m6A	310366	+	CTAYNNNNNNNTRTC	Pnuc_0318, ABC-2 type transporter		
m6A	310376	-	GAYANNNNNNNRTAG	upstream Pnuc_0316, glycosyl transferase, family 4		
m6A	312933	+	GAYANNNNNNNRTAG	Pnuc_0320, Methyltransferase type 11		
m6A	312943	-	CTAYNNNNNNNTRTC	upstream Pnuc_0316, glycosyl transferase, family 4		
m6A	313175	+	GAYANNNNNNNRTAG	Pnuc_0320, Methyltransferase type 11		
m6A	313185	-	CTAYNNNNNNNTRTC	upstream Pnuc_0316, glycosyl transferase, family 4		
m4C	318996	+		Pnuc_0323, Phytanoyl-CoA dioxygenase		
m6A	319009	-	GAYANNNNNNNRTAG	upstream Pnuc_0316, glycosyl transferase, family 4		
m4C	320324	+		Pnuc_0325, amine oxidase		
m4C	320845	+		Pnuc_0325, amine oxidase		
m6A	320925	+	GAYANNNNNNNRTAG	Pnuc_0325, amine oxidase		
m6A	320935	-	CTAYNNNNNNNTRTC	upstream Pnuc_0316, glycosyl transferase, family 4		
m6A	321154	+	CTAYNNNNNNNTRTC	Pnuc_0325, amine oxidase		
m6A	321164	-	GAYANNNNNNNRTAG	upstream Pnuc_0316, glycosyl transferase, family 4		
m4C	322076	+		upstream Pnuc_0329, UbiA prenyltransferase		
m6A	322610	+		upstream Pnuc_0329, UbiA prenyltransferase		
m6A	324282	+		Pnuc_0329, UbiA prenyltransferase		
m6A	324292	+	GAYANNNNNNNRTAG	Pnuc_0329, UbiA prenyltransferase		
m6A	324302	-	CTAYNNNNNNNTRTC	upstream Pnuc_0328, FAD linked oxidase domain protein		
m4C	328401	-		upstream Pnuc_0332, hypothetical protein		
m6A	329369	-		Pnuc_0334, Adenylyl-sulfate kinase		
m4C	329501	-		upstream Pnuc_0334, Adenylyl-sulfate kinase		
m4C	329877	+		Pnuc_0335, glycosyl transferase, family 2		
m6A	332882	-		upstream Pnuc_0336, hypothetical protein		
m4C	334253	+		Pnuc_0337, acyltransferase 3		
m6A	335211	-		upstream Pnuc_0336, hypothetical protein		
m6A	335858	+	CTAYNNNNNNNTRTC	Pnuc_0338, hypothetical protein		
m6A	335868	-	GAYANNNNNNNRTAG	upstream Pnuc_0336, hypothetical protein		
m4C	336301	+		upstream Pnuc_0340, cobalamin (vitamin B12) biosynthesis CbiX protein		
m4C	338125	+		Pnuc_0342, transcriptional regulator, ArsR family		
m6A	345459	+	GAYANNNNNNNRTAG	upstream Pnuc_0351, acyltransferase 3		
m6A	345469	-	CTAYNNNNNNNTRTC	upstream Pnuc_0349, hypothetical protein		
m6A	347772	+	GAYANNNNNNNRTAG	Pnuc_0353, Choline dehydrogenase		
m6A	347782	-	CTAYNNNNNNNTRTC	upstream Pnuc_0352, protein of unknown function DUF615		
m4C	349093	+		Pnuc_0354, protein of unknown function DUF6, transmembrane		
m4C	349184	-		upstream Pnuc_0352, protein of unknown function DUF615		
m4C	354187	+		Pnuc_0358, amino acid/amide ABC transporter ATP-binding protein 2, HAAT family		
m4C	354926	+		upstream Pnuc_0362, conserved hypothetical protein		
m6A	357990	+		upstream Pnuc_0364, Lytic transglycosylase, catalytic		
m6A	358119	-		Pnuc_0363, DNA/RNA non-specific endonuclease		
m4C	363047	+		upstream Pnuc_0367, BLUF domain protein		
m4C	367208	+		Pnuc_0371, transcriptional regulator, TetR family		
m4C	367793	+		Pnuc_0372, isochorismatase hydrolase		
m6A	371430	+		upstream Pnuc_0379, PpiC-type peptidyl-prolyl cis-trans isomerase		

m4C	373857	+		upstream Pnuc_0384, D-isomer specific 2-hydroxyacid dehydrogenase, NAD-binding protein
m6A	374462	+		upstream Pnuc_0384, D-isomer specific 2-hydroxyacid dehydrogenase, NAD-binding protein
m4C	375418	-		Pnuc_0383, 4Fe-4S ferredoxin, iron-sulfur binding domain protein
m4C	376773	-		Pnuc_0385, conserved hypothetical protein
m4C	378167	+		upstream Pnuc_0390, ribosomal large subunit pseudouridine synthase C
m4C	379643	+		upstream Pnuc_0390, ribosomal large subunit pseudouridine synthase C
m4C	380414	+		upstream Pnuc_0390, ribosomal large subunit pseudouridine synthase C
m6A	381442	-		Pnuc_0389, RNAse E
m6A	382815	+		upstream Pnuc_0390, ribosomal large subunit pseudouridine synthase C
m6A	387862	-		upstream Pnuc_0394, maf protein
m4C	387959	-		upstream Pnuc_0394, maf protein
m4C	388854	-		upstream Pnuc_0394, maf protein
m4C	391943	+		Pnuc_0400, 3-oxoacyl-[acyl-carrier-protein] reductase
m4C	392231	-		upstream Pnuc_0394, maf protein
m4C	395248	-		upstream Pnuc_0394, maf protein
m6A	395250	-		upstream Pnuc_0394, maf protein
m6A	397041	+	CTAYNNNNNNNTRTC	Pnuc_0404, GTP-binding protein LepA
m6A	397051	-	GAYANNNNNNNRTAG	upstream Pnuc_0394, maf protein
m4C	398900	-		upstream Pnuc_0394, maf protein
m6A	400511	-		upstream Pnuc_0394, maf protein
m6A	401086	+	CTAYNNNNNNNTRTC	Pnuc_0409, pyridoxine 5'-phosphate synthase
m6A	401096	-	GAYANNNNNNNRTAG	upstream Pnuc_0394, maf protein
m6A	401232	+	GAYANNNNNNNRTAG	Pnuc_0410, holo-acyl-carrier-protein synthase
m6A	401242	-	CTAYNNNNNNNTRTC	upstream Pnuc_0394, maf protein
m6A	401354	+		Pnuc_0410, holo-acyl-carrier-protein synthase
m4C	407582	+		Pnuc_0416, CDP-diacylglycerol-glycerol-3-phosphate-3-phosphatidyltransferase
m4C	408512	-		upstream Pnuc_0414, conserved hypothetical protein
m4C	410693	+		upstream Pnuc_0420, SSU ribosomal protein S6P
m6A	413274	+		Pnuc_0424, DnaB domain protein helicase, C-terminal domain protein
m4C	414298	+		Pnuc_0424, DnaB domain protein helicase, C-terminal domain protein
m4C	417659	-		Pnuc_0428, PhoH family protein
m4C	418723	+		upstream Pnuc_0437, aminotransferase
m6A	419827	-		upstream Pnuc_0430, polysaccharide deacetylase
m6A	420798	-		Pnuc_0431, NAD-dependent epimerase/dehydratase
m4C	421451	+		upstream Pnuc_0437, aminotransferase
m6A	421455	+	GAYANNNNNNNRTAG	upstream Pnuc_0437, aminotransferase
m6A	421465	-	CTAYNNNNNNNTRTC	Pnuc_0432, formyl transferase domain protein
m4C	422526	-		Pnuc_0433, glycosyl transferase, family 2
m6A	422836	+	CTAYNNNNNNNTRTC	upstream Pnuc_0437, aminotransferase
m6A	422846	-	GAYANNNNNNNRTAG	Pnuc_0434, DegT/DnrJ/EryC1/StrS aminotransferase
m4C	425783	-		Pnuc_0436, protein of unknown function DUF498
m4C	425791	-		Pnuc_0436, protein of unknown function DUF499
m4C	434948	+		Pnuc_0444, major facilitator superfamily MFS_1
m4C	436608	+		upstream Pnuc_0447, ATPase AAA-2 domain protein

m4C	438249	+		Pnuc_0447, ATPase AAA-2 domain protein		
m6A	438837	+		Pnuc_0447, ATPase AAA-2 domain protein		
m6A	439040	+	GAYANNNNNNNRTAG	Pnuc_0447, ATPase AAA-2 domain protein		
m6A	439050	-	CTAYNNNNNNNTRTC	upstream Pnuc_0446, Excinuclease ABC, C subunit domain protein		
m4C	439112	+		Pnuc_0447, ATPase AAA-2 domain protein		
m6A	440012	+	GAYANNNNNNNRTAG	Pnuc_0447, ATPase AAA-2 domain protein		
m6A	440022	-	CTAYNNNNNNNTRTC	upstream Pnuc_0446, Excinuclease ABC, C subunit domain protein		
m6A	442569	+	GAYANNNNNNNRTAG	Pnuc_0450, major facilitator superfamily MFS_1		
m6A	442579	-	CTAYNNNNNNNTRTC	upstream Pnuc_0449, Uncharacterized protein UPF0065		
m4C	445233	-		upstream Pnuc_0449, Uncharacterized protein UPF0065		
m6A	445942	+	CTAYNNNNNNNTRTC	upstream Pnuc_0453, cytochrome c oxidase, cbb3-type, subunit I		
m6A	445952	-	GAYANNNNNNNRTAG	upstream Pnuc_0449, Uncharacterized protein UPF0065		
m4C	449523	-		upstream Pnuc_0449, Uncharacterized protein UPF0066		
m4C	451434	+		upstream Pnuc_0461, putative transmembrane protein		
m6A	453512	+	CTAYNNNNNNNTRTC	Pnuc_0463, UspA domain protein		
m6A	453522	-	GAYANNNNNNNRTAG	upstream Pnuc_0462, conserved hypothetical protein		
m6A	454717	+		upstream Pnuc_0466, Mandelate racemase/muconate lactonizing enzyme, C-terminal domain protein		
m4C	457971	+		upstream Pnuc_0471, 3-hydroxyacyl-CoA dehydrogenase		
m4C	458223	+		upstream Pnuc_0471, 3-hydroxyacyl-CoA dehydrogenase		
m4C	461032	+		upstream Pnuc_0471, 3-hydroxyacyl-CoA dehydrogenase		
m6A	461835	+	GAYANNNNNNNRTAG	upstream Pnuc_0471, 3-hydroxyacyl-CoA dehydrogenase		
m6A	461845	-	CTAYNNNNNNNTRTC	Pnuc_0470, D-isomer specific 2-hydroxyacid dehydrogenase, NAD-binding protein		
m4C	465062	-		upstream Pnuc_0472, major facilitator superfamily MFS_1		
m4C	465452	+		Pnuc_0473, cytochrome B561		
m4C	472152	+		upstream Pnuc_0485, Uncharacterized protein UPF0065		
m6A	472803	+		upstream Pnuc_0485, Uncharacterized protein UPF0066		
m4C	474531	+		upstream Pnuc_0485, Uncharacterized protein UPF0067		
m6A	474614	+	CTAYNNNNNNNTRTC	upstream Pnuc_0485, Uncharacterized protein UPF0068		
m4C	474619	-		upstream Pnuc_0484, Rhodanese domain protein		
m6A	474624	-	GAYANNNNNNNRTAG	upstream Pnuc_0484, Rhodanese domain protein		
m4C	477739	-		upstream Pnuc_0484, Rhodanese domain protein		
m4C	479258	-		upstream Pnuc_0484, Rhodanese domain protein		
m4C	481144	+		upstream Pnuc_0493, DNA gyrase subunit A		
m4C	482087	-		upstream Pnuc_0491, 3-demethylubiquinone-9 3-methyltransferase		
m4C	489516	+		Pnuc_0497, prephenate dehydrogenase		
m4C	489659	+		Pnuc_0497, prephenate dehydrogenase		
m4C	489973	-		upstream Pnuc_0492, OmpA/MotB domain protein		
m6A	490233	+		Pnuc_0498, 3-phosphoshikimate 1-carboxyvinyltransferase		
m4C	491263	+		Pnuc_0499, cytidylate kinase		
m4C	491303	-		upstream Pnuc_0492, OmpA/MotB domain protein		
m6A	491847	+		upstream Pnuc_0500, SSU ribosomal protein S1P		
m4C	492724	-		upstream Pnuc_0492, OmpA/MotB domain protein		
m4C	496985	+		Pnuc_0504, D-alpha,beta-D-heptose 7-phosphate 1-kinase		
m4C	501868	+		upstream Pnuc_0510, histone deacetylase superfamily		

m4C	504222	+		Pnuc_0511, AMP-dependent synthetase and ligase		
m6A	506888	-		upstream Pnuc_0509, lytic murein transglycosylase B		
m4C	508533	-		upstream Pnuc_0509, lytic murein transglycosylase B		
m6A	511656	+	GAYANNNNNNNRTAG	Pnuc_0520, LSU ribosomal protein L19P		
m6A	511666	-	CTAYNNNNNNNTRTC	upstream Pnuc_0516, protein of unknown function DUF306, Meta and HslJ		
m6A	513793	+	CTAYNNNNNNNTRTC	upstream Pnuc_0525, Exonuclease, RNase T and DNA polymerase III		
m6A	513803	-	GAYANNNNNNNRTAG	Pnuc_0523, ribosome small subunit-dependent GTPase A		
m4C	515473	+		upstream Pnuc_0525, Exonuclease, RNase T and DNA polymerase III		
m4C	515660	+		upstream Pnuc_0525, Exonuclease, RNase T and DNA polymerase III		
m4C	515722	+		upstream Pnuc_0525, Exonuclease, RNase T and DNA polymerase III		
m4C	516276	+		Pnuc_0525, Exonuclease, RNase T and DNA polymerase III		
m4C	516409	+		Pnuc_0525, Exonuclease, RNase T and DNA polymerase III		
m6A	516835	+	GAYANNNNNNNRTAG	upstream Pnuc_0527, microcin-processing peptidase 1, Unknown type peptidase, MEROPS family U62		
m6A	516845	-	CTAYNNNNNNNTRTC	Pnuc_0526, molybdopterin adenylyltransferase		
m6A	517316	-		upstream Pnuc_0526, molybdopterin adenylyltransferase		
m4C	523583	+		upstream Pnuc_0534, protein of unknown function DUF485		
m4C	524625	-		upstream Pnuc_0531, phospholipase/Carboxylesterase		
m6A	525416	-		upstream Pnuc_0531, phospholipase/Carboxylesterase		
m4C	525674	+		Pnuc_0535, SSS sodium solute transporter superfamily		
m4C	525690	-		upstream Pnuc_0531, phospholipase/Carboxylesterase		
m6A	529410	+	GAYANNNNNNNRTAG	upstream Pnuc_0541, Glutathione S-transferase, N-terminal domain protein		
m6A	529420	-	CTAYNNNNNNNTRTC	upstream Pnuc_0538, hypothetical protein		
m4C	529465	+		upstream Pnuc_0541, Glutathione S-transferase, N-terminal domain protein		
m4C	532022	+		upstream Pnuc_0541, Glutathione S-transferase, N-terminal domain protein		
m4C	534332	-		upstream Pnuc_0540, GCN5-related N-acetyltransferase		
m4C	535051	-		upstream Pnuc_0540, GCN5-related N-acetyltransferase		
m6A	540218	-		upstream Pnuc_0547, putative phytochelatin synthase		
m4C	541005	+		Pnuc_0550, secretory lipase		
m4C	543554	+		Pnuc_0552, Cl- channel, voltage-gated family protein		
m4C	548812	-		upstream Pnuc_0555, cytochrome c, class I		
m6A	549517	+		upstream Pnuc_0559, putative transmembrane protein		
m6A	553023	+	CTAYNNNNNNNTRTC	upstream Pnuc_0565, hypothetical protein		
m6A	553033	-	GAYANNNNNNNRTAG	Pnuc_0563, integral membrane sensor hybrid histidine kinase		
m6A	555606	-		upstream Pnuc_0564, two component transcriptional regulator, LuxR family		
m4C	556422	+		upstream Pnuc_0570, cation diffusion facilitator family transporter		
m4C	557356	+		upstream Pnuc_0570, cation diffusion facilitator family transporter		
m6A	560162	+	GAYANNNNNNNRTAG	Pnuc_0570, cation diffusion facilitator family transporter		
m6A	560172	-	CTAYNNNNNNNTRTC	upstream Pnuc_0569, GCN5-related N-acetyltransferase		
m6A	561102	-		Pnuc_0571, beta-lactamase domain protein		
m4C	572159	-		upstream Pnuc_0582, hypothetical protein		
m4C	572808	+		upstream Pnuc_0586, cold-shock DNA-binding protein family		
m6A	580739	+		upstream Pnuc_0597, putative iron-sulfur cluster binding protein		
m6A	584889	-		Pnuc_0600, FAD linked oxidase domain protein		
m6A	587239	-	CTAYNNNNNNNTRTC	Pnuc_0600, FAD linked oxidase domain protein		

m4C	591454	-		upstream Pnuc_0601, transcriptional regulator, GntR family		
m4C	597394	-		Pnuc_0607, glycosyl transferase, family 9		
m6A	598039	+		upstream Pnuc_0610, lipid A ABC exporter, fused ATPase and inner membrane subunits MsbA		
m4C	598167	-		Pnuc_0608, glycosyl transferase, family 2		
m4C	598618	+		upstream Pnuc_0610, lipid A ABC exporter, fused ATPase and inner membrane subunits MsbA		
m6A	598932	+	GAYANNNNNNNRTAG	upstream Pnuc_0610, lipid A ABC exporter, fused ATPase and inner membrane subunits MsbA		
m6A	598942	-	CTAYNNNNNNNTRTC	Pnuc_0609, glycosyl transferase, family 2		
m6A	599429	+		Pnuc_0610, lipid A ABC exporter, fused ATPase and inner membrane subunits MsbA		
m4C	599511	+		Pnuc_0610, lipid A ABC exporter, fused ATPase and inner membrane subunits MsbA		
m4C	602158	-		Pnuc_0611, RNAse G		
m6A	604916	+		upstream Pnuc_R0016, tRNA-Leu		
m6A	605773	-		Pnuc_0617, phosphoribosylamine-glycine ligase		
m6A	606385	-		Pnuc_0617, phosphoribosylamine-glycine ligase		
m4C	612590	-		upstream Pnuc_0622, permease YjgP/YjgQ family protein		
m6A	616740	-		upstream Pnuc_0622, permease YjgP/YjgQ family protein		
m4C	616828	+		Pnuc_0627, Malonyl-CoA decarboxylase		
m6A	618648	+	GAYANNNNNNNRTAG	Pnuc_0628, TRAP dicarboxylate transporter- DctP subunit		
m6A	618658	-	CTAYNNNNNNNTRTC	upstream Pnuc_0622, permease YjgP/YjgQ family protein		
m4C	618674	+		Pnuc_0628, TRAP dicarboxylate transporter- DctP subunit		
m4C	620884	+		Pnuc_0631, AMP-dependent synthetase and ligase		
m6A	621941	+	GAYANNNNNNNRTAG	Pnuc_0631, AMP-dependent synthetase and ligase		
m6A	621951	-	CTAYNNNNNNNTRTC	upstream Pnuc_0622, permease YjgP/YjgQ family protein		
m6A	624339	-		Pnuc_0635, TonB-dependent receptor, plug		
m6A	629963	+		Pnuc_0638, L-carnitine dehydratase/bile acid-inducible protein F		
m4C	631605	+		Pnuc_0640, L-carnitine dehydratase/bile acid-inducible protein F		
m6A	632692	+		Pnuc_0641, Methionyl-tRNA formyltransferase		
m4C	634706	-		upstream Pnuc_0642, hypothetical protein		
m4C	634852	-		upstream Pnuc_0642, hypothetical protein		
m4C	636470	+		upstream Pnuc_0648, formate dehydrogenase, subunit FdhD		
m6A	637029	+		upstream Pnuc_0648, formate dehydrogenase, subunit FdhD		
m4C	637138	+		upstream Pnuc_0648, formate dehydrogenase, subunit FdhD		
m4C	637356	-		Pnuc_0647, methionyl-tRNA synthetase		
m4C	637984	-		Pnuc_0647, methionyl-tRNA synthetase		
m6A	638441	+		upstream Pnuc_0648, formate dehydrogenase, subunit FdhD		
m6A	639054	+	GAYANNNNNNNRTAG	Pnuc_0648, formate dehydrogenase, subunit FdhD		
m6A	639064	-	CTAYNNNNNNNTRTC	upstream Pnuc_0647, methionyl-tRNA synthetase		
m4C	639945	+		Pnuc_0649, dCTP deaminase		
m4C	640774	-		upstream Pnuc_0647, methionyl-tRNA synthetase		
m6A	642290	+	CTAYNNNNNNNTRTC	Pnuc_0651, Lysine decarboxylase		
m6A	642300	-	GAYANNNNNNNRTAG	upstream Pnuc_0647, methionyl-tRNA synthetase		
m4C	644088	-		Pnuc_0653, TRAP dicarboxylate transporter, DctM subunit		
m4C	645924	-		Pnuc_0654, Tripartite ATP-independent periplasmic transporter, DctQ component		
m4C	648447	+		Pnuc_0656, porphobilinogen deaminase		
m4C	650385	+		upstream Pnuc_0660, Lysine exporter protein (LYSE/YGGA)		

m4C	650696	+		upstream Pnuc_0660, Lysine exporter protein (LYSE/YGGA)		
m4C	653141	+		upstream Pnuc_0662, peptide methionine sulfoxide reductase		
m4C	653204	+		upstream Pnuc_0662, peptide methionine sulfoxide reductase		
m4C	654796	-		Pnuc_0663, Pyridoxamine 5'-phosphate oxidase		
m6A	656037	+		upstream Pnuc_0665, amino acid/polyamine/organocation transporter, APC superfamily		
m6A	656304	-		upstream Pnuc_0663, Pyridoxamine 5'-phosphate oxidase		
m4C	656646	-		upstream Pnuc_0663, Pyridoxamine 5'-phosphate oxidase		
m4C	661291	+		Pnuc_0668, aminopeptidase N		
m4C	663672	+		upstream Pnuc_0672, ABC transporter related protein		
m4C	664099	+		upstream Pnuc_0672, ABC transporter related protein		
m6A	664401	+	CTAYNNNNNNNNTRTC	upstream Pnuc_0672, ABC transporter related protein		
m4C	664610	-		Pnuc_0670, cyanophycin synthetase		
m4C	668365	-		upstream Pnuc_0671, cyanophycin synthetase		
m4C	669887	+		Pnuc_0672, ABC transporter related protein		
m6A	670881	-		upstream Pnuc_0671, cyanophycin synthetase		
m4C	673061	-		upstream Pnuc_0671, cyanophycin synthetase		
m4C	673851	-		upstream Pnuc_0671, cyanophycin synthetase		
m4C	676557	-		upstream Pnuc_0671, cyanophycin synthetase		
m6A	679130	+		upstream Pnuc_0681, response regulator receiver protein		
m4C	683590	+		upstream Pnuc_0686, V-type H(+)-translocating pyrophosphatase		
m4C	683682	-		upstream Pnuc_0684, NAD+ synthetase		
m6A	685642	+	CTAYNNNNNNNNTRTC	Pnuc_0686, V-type H(+)-translocating pyrophosphatase		
m6A	685652	-	GAYANNNNNNNNRTAG	upstream Pnuc_0685, Inorganic diphosphatase		
m4C	688295	+		upstream Pnuc_0690, DNA translocase FtsK		
m4C	689350	+		Pnuc_0690, DNA translocase FtsK		
m4C	690307	-		upstream Pnuc_0689, thioredoxin reductase		
m4C	692254	-		upstream Pnuc_0689, thioredoxin reductase		
m6A	694070	-		upstream Pnuc_0694, transcriptional regulator/antitoxin, MazE		
m4C	695098	+		Pnuc_0695, ATP-dependent DNA helicase RecQ		
m4C	702329	-		upstream Pnuc_R0019, SRP RNA; RNA component of signal recognition particle		
m4C	702948	-		upstream Pnuc_R0019, SRP RNA; RNA component of signal recognition particle		
m4C	704404	+		Pnuc_0703, Carbohydrate-selective porin OprB		
m4C	704548	+		upstream Pnuc_0704, SOS response UmuD protein, Serine peptidase,MEROPS family S24		
m4C	706374	-		upstream Pnuc_R0019, SRP RNA; RNA component of signal recognition particle		
m6A	706486	-		upstream Pnuc_R0019, SRP RNA; RNA component of signal recognition particle		
m6A	708630	+		upstream Pnuc_0709, protein of unknown function DUF465		
m6A	711386	-		Pnuc_0712, membrane protein of unknown function		
m4C	712379	-		upstream Pnuc_0713, SlyX family protein		
m4C	712948	+		Pnuc_0714, luciferase family protein		
m4C	713493	-		Pnuc_0716, YaeQ family protein		
m4C	714147	-		upstream Pnuc_0716, YaeQ family protein		
m6A	715576	-		upstream Pnuc_0716, YaeQ family protein		
m6A	716042	+		Pnuc_0718, coproporphyrinogen III oxidase, anaerobic		
m6A	716815	+	CTAYNNNNNNNNTRTC	Pnuc_0718, coproporphyrinogen III oxidase, anaerobic		

m6A	716825	-	GAYANNNNNNNRTAG	upstream Pnuc_0716, YaeQ family protein
m6A	717198	+	CTAYNNNNNNNTRTC	upstream Pnuc_0719, OmpW family protein
m6A	717208	-	GAYANNNNNNNRTAG	upstream Pnuc_0716, YaeQ family protein
m4C	719965	+		Pnuc_0721, ABC transporter related protein
m4C	720667	-		Pnuc_0722, conserved hypothetical protein
m4C	724646	+		Pnuc_0726, TRAP transporter solute receptor, TAXI family
m6A	725043	-		upstream Pnuc_0725, Pirin domain protein
m6A	726854	-		Pnuc_0728, sulfide dehydrogenase (flavocytochrome), flavoprotein subunit
m4C	727058	+		upstream Pnuc_0734, 2-oxo-acid dehydrogenase E1 subunit, homodimeric type
m4C	727350	+		upstream Pnuc_0734, 2-oxo-acid dehydrogenase E1 subunit, homodimeric type
m4C	727751	+		upstream Pnuc_0734, 2-oxo-acid dehydrogenase E1 subunit, homodimeric type
m4C	728120	+		upstream Pnuc_0734, 2-oxo-acid dehydrogenase E1 subunit, homodimeric type
m4C	729810	+		upstream Pnuc_0734, 2-oxo-acid dehydrogenase E1 subunit, homodimeric type
m6A	730423	+	CTAYNNNNNNNTRTC	upstream Pnuc_0734, 2-oxo-acid dehydrogenase E1 subunit, homodimeric type
m6A	730433	-	GAYANNNNNNNRTAG	Pnuc_0731, methenyltetrahydrofolate cyclohydrolase / 5,10-methylenetetrahydrofolate dehydrogenase (NADP+)
m4C	734318	-		upstream Pnuc_0733, PAS/PAC sensor signal transduction histidine kinase
m4C	738410	+		Pnuc_0734, 2-oxo-acid dehydrogenase E1 subunit, homodimeric type
m6A	739406	+	CTAYNNNNNNNTRTC	Pnuc_0736, dihydrolipoamide dehydrogenase
m6A	739416	-	GAYANNNNNNNRTAG	upstream Pnuc_0733, PAS/PAC sensor signal transduction histidine kinase
m4C	739572	-		upstream Pnuc_0733, PAS/PAC sensor signal transduction histidine kinase
m6A	741643	+	CTAYNNNNNNNTRTC	Pnuc_0738, protein of unknown function DUF6, transmembrane
m6A	741653	-	GAYANNNNNNNRTAG	upstream Pnuc_0737, phasin family protein
m4C	742273	+		Pnuc_0738, protein of unknown function DUF6, transmembrane
m4C	742983	+		Pnuc_0739, murein-DD-endopeptidase, Serine peptidase,MEROPS family S11
m4C	743138	-		upstream Pnuc_0737, phasin family protein
m4C	746050	-		Pnuc_0742, Arginyltransferase
m4C	746222	-		Pnuc_0743, Leucyltransferase
m6A	746554	+		upstream Pnuc_0753, protein of unknown function DUF328
m4C	746934	-		Pnuc_0744, NUDIX hydrolase
m4C	747724	-		Pnuc_0745, Anthranilate synthase component I and chorismate binding protein
m4C	749359	+		upstream Pnuc_0753, protein of unknown function DUF328
m4C	749722	-		Pnuc_0746, peptidyl-prolyl cis-trans isomerase, cyclophilin type
m6A	750406	+	GAYANNNNNNNRTAG	upstream Pnuc_0753, protein of unknown function DUF328
m6A	750416	-	CTAYNNNNNNNTRTC	Pnuc_0747, DSBA oxidoreductase
m6A	751830	+		upstream Pnuc_0753, protein of unknown function DUF328
m6A	762452	+	CTAYNNNNNNNTRTC	Pnuc_0761, succinate dehydrogenase subunit B
m6A	762462	-	GAYANNNNNNNRTAG	upstream Pnuc_0756, malate dehydrogenase (NAD)
m6A	763654	+		Pnuc_0763, citrate synthase
m4C	763691	-		upstream Pnuc_0756, malate dehydrogenase (NAD)
m4C	763891	-		upstream Pnuc_0756, malate dehydrogenase (NAD)
m4C	763981	-		upstream Pnuc_0756, malate dehydrogenase (NAD)
m4C	764280	+		Pnuc_0763, citrate synthase
m4C	764981	+		Pnuc_0764, 3-isopropylmalate dehydratase, large subunit
m6A	767120	+		upstream Pnuc_0767, 3-isopropylmalate dehydrogenase

m4C	767735	+		Pnuc_0767, 3-isopropylmalate dehydrogenase		
m6A	768513	+	CTAYNNNNNNNTRTC	Pnuc_0768, aspartate semialdehyde dehydrogenase		
m6A	768523	-	GAYANNNNNNNRTAG	upstream Pnuc_0756, malate dehydrogenase (NAD)		
m4C	768661	+		Pnuc_0768, aspartate semialdehyde dehydrogenase		
m4C	768983	+		Pnuc_0768, aspartate semialdehyde dehydrogenase		
m6A	771107	+		Pnuc_0770, tRNA pseudouridine synthase A		
m4C	771705	-		upstream Pnuc_0756, malate dehydrogenase (NAD)		
m4C	774306	-		upstream Pnuc_0756, malate dehydrogenase (NAD)		
m4C	774401	-		upstream Pnuc_0756, malate dehydrogenase (NAD)		
m6A	774540	+	CTAYNNNNNNNTRTC	Pnuc_0774, acetyl-CoA carboxylase carboxyltransferase subunit alpha		
m6A	774550	-	GAYANNNNNNNRTAG	upstream Pnuc_0756, malate dehydrogenase (NAD)		
m4C	775167	-		upstream Pnuc_0756, malate dehydrogenase (NAD)		
m4C	775467	-		upstream Pnuc_0756, malate dehydrogenase (NAD)		
m4C	775663	-		upstream Pnuc_0756, malate dehydrogenase (NAD)		
m4C	775894	+		Pnuc_0775, FolC bifunctional protein		
m4C	776520	-		upstream Pnuc_0756, malate dehydrogenase (NAD)		
m4C	779460	-		upstream Pnuc_0756, malate dehydrogenase (NAD)		
m6A	783630	-		upstream Pnuc_0782, molybdate ABC transporter, inner membrane subunit		
m6A	783973	+	GAYANNNNNNNRTAG	Pnuc_0784, OmpA/MotB domain protein		
m6A	783983	-	CTAYNNNNNNNTRTC	upstream Pnuc_0782, molybdate ABC transporter, inner membrane subunit		
m6A	784667	-		upstream Pnuc_0782, molybdate ABC transporter, inner membrane subunit		
m4C	786811	-		Pnuc_0787, cytochrome c family protein		
m6A	786874	+	GAYANNNNNNNRTAG	upstream Pnuc_0788, protein of unknown function DUF395, YeeE/YedE		
m6A	786884	-	CTAYNNNNNNNTRTC	Pnuc_0787, cytochrome c family protein		
m6A	789389	+	GAYANNNNNNNRTAG	Pnuc_0792, Rhodanese domain protein		
m6A	789399	-	CTAYNNNNNNNTRTC	upstream Pnuc_0790, hypothetical protein		
m4C	792064	+		Pnuc_0796, cyclic nucleotide-binding protein		
m6A	793322	+		upstream Pnuc_0800, beta-lactamase domain protein		
m6A	793988	+	CTAYNNNNNNNTRTC	upstream Pnuc_0800, beta-lactamase domain protein		
m6A	793998	-	GAYANNNNNNNRTAG	Pnuc_0799, FAD-dependent pyridine nucleotide-disulfide oxidoreductase		
m6A	794087	+		upstream Pnuc_0800, beta-lactamase domain protein		
m4C	801055	+		Pnuc_0807, sulfate thiol esterase SoxB		
m4C	801332	-		upstream Pnuc_0799, FAD-dependent pyridine nucleotide-disulfide oxidoreductase		
m6A	802163	+	GAYANNNNNNNRTAG	Pnuc_0807, sulfate thiol esterase SoxB		
m6A	802173	-	CTAYNNNNNNNTRTC	upstream Pnuc_0799, FAD-dependent pyridine nucleotide-disulfide oxidoreductase		
m4C	804214	-		upstream Pnuc_0799, FAD-dependent pyridine nucleotide-disulfide oxidoreductase		
m6A	811058	-		Pnuc_0818, acyl-CoA dehydrogenase domain protein		
m4C	812378	-		upstream Pnuc_0818, acyl-CoA dehydrogenase domain protein		
m4C	812493	-		upstream Pnuc_0818, acyl-CoA dehydrogenase domain protein		
m6A	815395	+	GAYANNNNNNNRTAG	upstream Pnuc_0825, 3-oxoacid CoA-transferase, A subunit		
m6A	815405	-	CTAYNNNNNNNTRTC	Pnuc_0822, protein of unknown function DUF482		
m4C	818014	+		Pnuc_0825, 3-oxoacid CoA-transferase, A subunit		
m6A	820903	+	CTAYNNNNNNNTRTC	Pnuc_0827, cation diffusion facilitator family transporter		
m6A	820913	-	GAYANNNNNNNRTAG	upstream Pnuc_0824, Electron-transferring-flavoprotein dehydrogenase		

m4C	822831	-		upstream Pnuc_0824, Electron-transferring-flavoprotein dehydrogenase		
m4C	825735	-		upstream Pnuc_0824, Electron-transferring-flavoprotein dehydrogenase		
m6A	829107	+	CTAYNNNNNNNTRTC	Pnuc_0834, phenylalanyl-tRNA synthetase beta subunit		
m6A	829117	-	GAYANNNNNNNRTAG	upstream Pnuc_0824, Electron-transferring-flavoprotein dehydrogenase		
m6A	829240	-		upstream Pnuc_0824, Electron-transferring-flavoprotein dehydrogenase		
m4C	831183	+		Pnuc_0837, thioesterase superfamily protein		
m4C	832785	+		upstream Pnuc_0840, 2-oxoglutarate dehydrogenase, E1 subunit		
m6A	837877	+	GAYANNNNNNNRTAG	Pnuc_0842, dihydrolipoamide dehydrogenase		
m6A	837887	-	CTAYNNNNNNNTRTC	upstream Pnuc_0839, DNA binding domain, excisionase family		
m4C	839527	+		Pnuc_0843, AFG1-family ATPase		
m4C	839567	+		Pnuc_0843, AFG1-family ATPase		
m4C	840271	-		upstream Pnuc_0839, DNA binding domain, excisionase family		
m4C	840802	-		upstream Pnuc_0839, DNA binding domain, excisionase family		
m4C	841897	-		upstream Pnuc_0839, DNA binding domain, excisionase family		
m6A	842339	-		upstream Pnuc_0839, DNA binding domain, excisionase family		
m4C	843787	+		Pnuc_0848, NlpBDapX family lipoprotein		
m4C	846473	-		upstream Pnuc_0850, Cupin 4 family protein		
m6A	846578	+		Pnuc_0851, putative FkbP-type peptidyl-prolyl cis-trans isomerase		
m4C	850566	-		Pnuc_0857, Tetratricopeptide TPR_2 repeat protein		
m6A	852895	-		upstream Pnuc_0857, Tetratricopeptide TPR_2 repeat protein		
m4C	855639	+		Pnuc_0861, tRNA(Ile)-lysidine synthetase		
m6A	856043	+		Pnuc_0862, aspartate kinase		
m6A	856210	+	GAYANNNNNNNRTAG	Pnuc_0862, aspartate kinase		
m6A	856220	-	CTAYNNNNNNNTRTC	upstream Pnuc_0857, Tetratricopeptide TPR_2 repeat protein		
m4C	856296	+		Pnuc_0862, aspartate kinase		
m4C	856710	+		Pnuc_0862, aspartate kinase		
m4C	859723	-		Pnuc_0865, transcriptional regulator, XRE family		
m6A	860021	-		upstream Pnuc_0865, transcriptional regulator, XRE family		
m4C	860044	-		upstream Pnuc_0865, transcriptional regulator, XRE family		
m4C	862259	-		upstream Pnuc_0865, transcriptional regulator, XRE family		
m4C	862685	-		upstream Pnuc_0865, transcriptional regulator, XRE family		
m4C	863254	-		upstream Pnuc_0865, transcriptional regulator, XRE family		
m4C	864375	+		Pnuc_0870, transporter, hydrophobe/amphiphile efflux-1(HAE1) family		
m6A	864766	+	CTAYNNNNNNNTRTC	Pnuc_0870, transporter, hydrophobe/amphiphile efflux-1(HAE1) family		
m6A	864776	-	GAYANNNNNNNRTAG	upstream Pnuc_0865, transcriptional regulator, XRE family		
m6A	865018	+	CTAYNNNNNNNTRTC	Pnuc_0870, transporter, hydrophobe/amphiphile efflux-1(HAE1) family		
m6A	865028	-	GAYANNNNNNNRTAG	upstream Pnuc_0865, transcriptional regulator, XRE family		
m4C	865461	-		upstream Pnuc_0865, transcriptional regulator, XRE family		
m6A	868410	+	GAYANNNNNNNRTAG	Pnuc_0872, Formyl-CoA transferase		
m6A	868420	-	CTAYNNNNNNNTRTC	upstream Pnuc_0865, transcriptional regulator, XRE family		
m4C	868581	-		upstream Pnuc_0865, transcriptional regulator, XRE family		
m4C	870431	+		Pnuc_0874, hypothetical protein		
m4C	870709	-		upstream Pnuc_0865, transcriptional regulator, XRE family		
m4C	871483	-		upstream Pnuc_0875, hypothetical protein		

m6A	872853	-		upstream Pnuc_0875, hypothetical protein		
m6A	873168	+	CTAYNNNNNNNNTRTC	Pnuc_0879, putative transmembrane protein		
m6A	873178	-	GAYANNNNNNNNRTAG	upstream Pnuc_0875, hypothetical protein		
m4C	877940	+		upstream Pnuc_0888, hypothetical protein		
m4C	879366	-		Pnuc_0886, conserved hypothetical protein		
m4C	880969	+		upstream Pnuc_0888, hypothetical protein		
m6A	882214	+	GAYANNNNNNNNRTAG	Pnuc_0889, phosphomethylpyrimidine kinase		
m6A	882224	-	CTAYNNNNNNNNTRTC	upstream Pnuc_R0023, tRNA-Asn		
m4C	882503	+		Pnuc_0889, phosphomethylpyrimidine kinase		
m4C	887087	+		upstream Pnuc_0900, DNA topoisomerase IV subunit B		
m4C	887655	+		upstream Pnuc_0900, DNA topoisomerase IV subunit B		
m4C	889137	-		Pnuc_0897, NUDIX hydrolase		
m6A	889846	-		upstream Pnuc_0897, NUDIX hydrolase		
m6A	891433	+		upstream Pnuc_0900, DNA topoisomerase IV subunit B		
m4C	894196	+		Pnuc_0900, DNA topoisomerase IV subunit B		
m6A	894883	+	GAYANNNNNNNNRTAG	Pnuc_0901, DNA topoisomerase IV subunit A		
m6A	894893	-	CTAYNNNNNNNNTRTC	upstream Pnuc_0899, Formyl-CoA transferase		
m6A	895256	-		upstream Pnuc_0899, Formyl-CoA transferase		
m4C	896466	+		Pnuc_0901, DNA topoisomerase IV subunit A		
m6A	896992	-		upstream Pnuc_0899, Formyl-CoA transferase		
m6A	898234	+	CTAYNNNNNNNNTRTC	upstream Pnuc_0903, conserved hypothetical protein		
m6A	898244	-	GAYANNNNNNNNRTAG	upstream Pnuc_0902, predicted sulfurylase subunit, molybdopterin cytosine dinucleotide biosynthesis		
m6A	901432	+		upstream Pnuc_0907, putative periplasmic cytochrome type-c oxidoreductase signal peptide protein		
m6A	903152	+	GAYANNNNNNNNRTAG	upstream Pnuc_0910, methylmalonyl-CoA mutase		
m6A	903162	-	CTAYNNNNNNNNTRTC	upstream Pnuc_0909, transcriptional regulator, GntR family		
m6A	905160	+		Pnuc_0910, methylmalonyl-CoA mutase		
m6A	909439	+	CTAYNNNNNNNNTRTC	Pnuc_0913, acetyl-CoA carboxylase, biotin carboxylase		
m6A	909449	-	GAYANNNNNNNNRTAG	upstream Pnuc_0909, transcriptional regulator, GntR family		
m4C	911262	+		Pnuc_0915, peptidase M22, glycoprotease		
m4C	912107	+		Pnuc_0917, hypothetical protein		
m4C	913241	+		upstream Pnuc_0919, alanine racemase		
m4C	917021	-		upstream Pnuc_0920, putative transmembrane protein		
m6A	918248	+		Pnuc_0923, poly(R)-hydroxyalkanoic acid synthase, class I		
m6A	921978	+		Pnuc_0926, SSU ribosomal protein S12P methylthiotransferase		
m4C	925623	-		Pnuc_0929, transcription-repair coupling factor		
m4C	926986	-		Pnuc_0929, transcription-repair coupling factor		
m4C	929165	-		upstream Pnuc_0929, transcription-repair coupling factor		
m4C	929591	+		Pnuc_0932, nitroreductase		
m4C	932124	-		upstream Pnuc_0929, transcription-repair coupling factor		
m4C	933953	+		Pnuc_0936, ATP-dependent proteinase, Serine peptidase,MEROPS family S16		
m6A	936437	-		upstream Pnuc_0929, transcription-repair coupling factor		
m6A	936991	+	CTAYNNNNNNNNTRTC	Pnuc_0937, hypothetical protein		
m6A	937001	-	GAYANNNNNNNNRTAG	upstream Pnuc_0929, transcription-repair coupling factor		
m4C	937618	-		upstream Pnuc_0929, transcription-repair coupling factor		

m4C	940174	+		Pnuc_0940, phosphoribosylformylglycinamidine synthase	
m6A	945908	+		upstream Pnuc_0945, CTP synthase	
m6A	946091	+	GAYANNNNNNNRTAG	upstream Pnuc_0945, CTP synthase	
m6A	946101	-	CTAYNNNNNNNTRTC	Pnuc_0942, aconitase	
m4C	946523	-		Pnuc_0942, aconitase	
m4C	946770	+		upstream Pnuc_0945, CTP synthase	
m4C	948299	+		Pnuc_0945, CTP synthase	
m4C	949510	+		Pnuc_0946, 2-dehydro-3-deoxyphosphooctonate aldolase	
m4C	951235	-		upstream Pnuc_0944, conserved hypothetical protein	
m4C	954331	+		Pnuc_0950, glutamyl-tRNA synthetase	
m4C	959380	+		upstream Pnuc_0958, 2-hydroxy-3-oxopropionate reductase	
m6A	959740	+	GAYANNNNNNNRTAG	Pnuc_0958, 2-hydroxy-3-oxopropionate reductase	
m6A	959750	-	CTAYNNNNNNNTRTC	upstream Pnuc_0957, hypothetical protein	
m4C	960743	+		Pnuc_0959, protein of unknown function UPF0227	
m6A	962004	+		Pnuc_0961, hypothetical protein	
m4C	963866	+		upstream Pnuc_0968, glycine cleavage T protein (aminomethyl transferase)	
m4C	964205	-		Pnuc_0964, hydrolase, TatD family	
m4C	965166	-		Pnuc_0965, DNA polymerase III, delta prime subunit	
m6A	966942	+	CTAYNNNNNNNTRTC	upstream Pnuc_0968, glycine cleavage T protein (aminomethyl transferase)	
m6A	966952	-	GAYANNNNNNNRTAG	Pnuc_0967, aminodeoxychorismate lyase	
m6A	967979	+	CTAYNNNNNNNTRTC	Pnuc_0968, glycine cleavage T protein (aminomethyl transferase)	
m6A	967989	-	GAYANNNNNNNRTAG	upstream Pnuc_0967, aminodeoxychorismate lyase	
m6A	968424	+		Pnuc_0968, glycine cleavage T protein (aminomethyl transferase)	
m4C	969819	-		Pnuc_0970, thioesterase superfamily protein	
m6A	971675	-		upstream Pnuc_0970, thioesterase superfamily protein	
m4C	975518	+		upstream Pnuc_0980, flavodoxin/nitric oxide synthase	
m4C	976238	-		Pnuc_0978, CBS domain containing protein	
m6A	976646	-		Pnuc_0979, tRNA-processing RNAse BN	
m6A	979842	-		upstream Pnuc_0979, tRNA-processing RNAse BN	
m6A	980618	+		upstream Pnuc_0999, ABC-type transporter, periplasmic component, NitT family	
m6A	980984	+	GAYANNNNNNNRTAG	upstream Pnuc_0999, ABC-type transporter, periplasmic component, NitT family	
m6A	980994	-	CTAYNNNNNNNTRTC	Pnuc_0983, alpha/beta hydrolase fold protein	
m6A	982286	+	CTAYNNNNNNNTRTC	upstream Pnuc_0999, ABC-type transporter, periplasmic component, NitT family	
m6A	982296	-	GAYANNNNNNNRTAG	Pnuc_0986, hypothetical protein	
m6A	984361	+		upstream Pnuc_0999, ABC-type transporter, periplasmic component, NitT family	
m6A	984510	+		upstream Pnuc_0999, ABC-type transporter, periplasmic component, NitT family	
m4C	986213	-		Pnuc_0991, NADH dehydrogenase	
m6A	988136	+	GAYANNNNNNNRTAG	upstream Pnuc_0999, ABC-type transporter, periplasmic component, NitT family	
m6A	988146	-	CTAYNNNNNNNTRTC	Pnuc_0992, Choloylglycine hydrolase	
m6A	990429	-		Pnuc_0995, nitrate ABC transporter, ATPase subunits C and D	
m4C	993131	+		upstream Pnuc_0999, ABC-type transporter, periplasmic component, NitT family	
m4C	994692	-		upstream Pnuc_0998, uroporphyrin-III C-methyltransferase	
m6A	995404	+	CTAYNNNNNNNTRTC	Pnuc_0999, ABC-type transporter, periplasmic component, NitT family	
m4C	995853	-		upstream Pnuc_0998, uroporphyrin-III C-methyltransferase	

m6A	997866	+		Pnuc_1000, assimilatory nitrite reductase (NAD(P)H) large subunit precursor		
m6A	1006842	-		upstream Pnuc_R0031, tRNA-Pro		
m6A	1008651	+	CTAYNNNNNNNTRTC	Pnuc_1010, TonB-dependent receptor		
m6A	1008661	-	GAYANNNNNNNRTAG	upstream Pnuc_R0031, tRNA-Pro2		
m6A	1009080	+		upstream Pnuc_1015, protein of unknown function UPF0044		
m4C	1009251	-		Pnuc_1011, phosphoglucosamine mutase		
m4C	1009306	+		upstream Pnuc_1015, protein of unknown function UPF0044		
m4C	1010615	-		Pnuc_1012, Dihydropteroate synthase		
m4C	1011804	+		upstream Pnuc_1015, protein of unknown function UPF0044		
m4C	1013826	-		upstream Pnuc_1014, 23S rRNA Um-2552 2'-O-methyltransferase		
m4C	1015521	-		upstream Pnuc_1017, transcription elongation factor GreA		
m6A	1019053	+	CTAYNNNNNNNTRTC	upstream Pnuc_1023, Methyltransferase type 11		
m4C	1019054	+		upstream Pnuc_1023, Methyltransferase type 11		
m6A	1019063	-	GAYANNNNNNNRTAG	Pnuc_1019, carbamoyl-phosphate synthase small subunit		
m4C	1019978	+		upstream Pnuc_1023, Methyltransferase type 11		
m6A	1020256	+	CTAYNNNNNNNTRTC	upstream Pnuc_1023, Methyltransferase type 11		
m6A	1020266	-	GAYANNNNNNNRTAG	upstream Pnuc_1019, carbamoyl-phosphate synthase small subunit		
m4C	1020270	-		upstream Pnuc_1019, carbamoyl-phosphate synthase small subunit		
m4C	1020860	-		Pnuc_1020, propionyl-CoA synthetase		
m4C	1020960	-		Pnuc_1020, propionyl-CoA synthetase		
m6A	1021303	+	CTAYNNNNNNNTRTC	upstream Pnuc_1023, Methyltransferase type 11		
m6A	1021313	-	GAYANNNNNNNRTAG	Pnuc_1020, propionyl-CoA synthetase		
m4C	1021990	-		Pnuc_1020, propionyl-CoA synthetase		
m6A	1027462	+	GAYANNNNNNNRTAG	Pnuc_1026, acriflavin resistance protein		
m6A	1027472	-	CTAYNNNNNNNTRTC	upstream Pnuc_1022, Hydroxyacylglutathione hydrolase		
m6A	1027824	-		upstream Pnuc_1022, Hydroxyacylglutathione hydrolase		
m4C	1030879	-		upstream Pnuc_1022, Hydroxyacylglutathione hydrolase		
m4C	1032865	-		Pnuc_1029, hypothetical protein		
m6A	1033357	+		upstream Pnuc_1032, Amidase		
m6A	1034059	-		Pnuc_1031, hypothetical protein		
m6A	1034060	+	CTAYNNNNNNNTRTC	upstream Pnuc_1032, Amidase		
m6A	1034070	-	GAYANNNNNNNRTAG	Pnuc_1031, hypothetical protein		
m4C	1035106	-		upstream Pnuc_1031, hypothetical protein		
m4C	1036445	+		upstream Pnuc_1035, methylmalonate-semialdehyde dehydrogenase(acylating)		
m4C	1037210	+		upstream Pnuc_1035, methylmalonate-semialdehyde dehydrogenase(acylating)		
m4C	1037402	-		Pnuc_1034, NADH:flavin oxidoreductase/NADH oxidase		
m4C	1037699	-		Pnuc_1034, NADH:flavin oxidoreductase/NADH oxidase		
m4C	1039663	+		Pnuc_1035, methylmalonate-semialdehyde dehydrogenase(acylating)		
m6A	1039882	-		upstream Pnuc_1034, NADH:flavin oxidoreductase/NADH oxidase		
m4C	1040324	+		upstream Pnuc_1057, putative lipoprotein		
m4C	1042147	+		upstream Pnuc_1057, putative lipoprotein		
m4C	1042450	-		Pnuc_1038, NADH dehydrogenase subunit N		
m4C	1042803	+		upstream Pnuc_1057, putative lipoprotein		
m4C	1043974	-		Pnuc_1039, NADH dehydrogenase subunit M		

m4C	1044521	-		Pnuc_1040, NADH dehydrogenase subunit L
m4C	1045576	-		Pnuc_1040, NADH dehydrogenase subunit L
m4C	1047364	-		Pnuc_1043, NADH dehydrogenase subunit I
m4C	1050439	+		upstream Pnuc_1057, putative lipoprotein
m6A	1050989	+		upstream Pnuc_1057, putative lipoprotein
m4C	1054222	-		Pnuc_1048, NADH dehydrogenase subunit D
m6A	1055538	+	GAYANNNNNNNRTAG	upstream Pnuc_1057, putative lipoprotein
m6A	1055548	-	CTAYNNNNNNNTRTC	Pnuc_1051, NADH dehydrogenase subunit A
m4C	1057480	+		upstream Pnuc_1057, putative lipoprotein
m4C	1060960	+		upstream Pnuc_1057, putative lipoprotein
m4C	1062053	+		upstream Pnuc_1064, RNA polymerase, sigma-24 subunit, RpoE
m4C	1062431	+		upstream Pnuc_1064, RNA polymerase, sigma-24 subunit, RpoE
m4C	1062602	+		upstream Pnuc_1064, RNA polymerase, sigma-24 subunit, RpoE
m4C	1063494	+		upstream Pnuc_1064, RNA polymerase, sigma-24 subunit, RpoE
m4C	1063802	+		upstream Pnuc_1064, RNA polymerase, sigma-24 subunit, RpoE
m4C	1066303	-		Pnuc_1062, acetolactate synthase, small subunit
m4C	1067873	+		upstream Pnuc_1064, RNA polymerase, sigma-24 subunit, RpoE
m4C	1068154	+		upstream Pnuc_1064, RNA polymerase, sigma-24 subunit, RpoE
m4C	1068289	+		upstream Pnuc_1064, RNA polymerase, sigma-24 subunit, RpoE
m6A	1069023	-		upstream Pnuc_1063, acetolactate synthase, large subunit
m4C	1069538	-		upstream Pnuc_1063, acetolactate synthase, large subunit
m4C	1069654	+		upstream Pnuc_1066, hypothetical protein
m4C	1072487	-		Pnuc_1068, protein of unknown function DUF214
m6A	1075780	+	GAYANNNNNNNRTAG	upstream Pnuc_1075, transcription elongation factor GreB
m6A	1075790	-	CTAYNNNNNNNTRTC	Pnuc_1071, hypothetical protein
m6A	1076951	+	CTAYNNNNNNNTRTC	upstream Pnuc_1075, transcription elongation factor GreB
m6A	1076961	-	GAYANNNNNNNRTAG	Pnuc_1073, ABC transporter related protein
m4C	1080829	-		Pnuc_1074, protein of unknown function DUF140
m6A	1092756	-		upstream Pnuc_1085, integral membrane sensor signal transduction histidine kinase
m6A	1093084	+	GAYANNNNNNNRTAG	Pnuc_1087, hypothetical protein
m6A	1093094	-	CTAYNNNNNNNTRTC	upstream Pnuc_1085, integral membrane sensor signal transduction histidine kinase
m6A	1095712	+	CTAYNNNNNNNTRTC	upstream Pnuc_1091, glucose-6-phosphate isomerase
m6A	1095722	-	GAYANNNNNNNRTAG	upstream Pnuc_1089, hypothetical protein
m6A	1096119	-		upstream Pnuc_1089, hypothetical protein
m6A	1096606	-		Pnuc_1090, FAD dependent oxidoreductase
m4C	1106620	+		upstream Pnuc_1097, hypothetical protein
m4C	1107202	+		upstream Pnuc_1097, hypothetical protein
m6A	1108023	+	GAYANNNNNNNRTAG	upstream Pnuc_1097, hypothetical protein
m6A	1108033	-	CTAYNNNNNNNTRTC	Pnuc_1095, outer membrane autotransporter barrel domain protein
m4C	1108297	+		upstream Pnuc_1097, hypothetical protein
m6A	1108814	-		Pnuc_1095, outer membrane autotransporter barrel domain protein
m6A	1109515	+	CTAYNNNNNNNTRTC	upstream Pnuc_1097, hypothetical protein
m6A	1109525	-	GAYANNNNNNNRTAG	Pnuc_1095, outer membrane autotransporter barrel domain protein
m4C	1111018	+		upstream Pnuc_1097, hypothetical protein

m6A	1111532	+		upstream Pnuc_1097, hypothetical protein		
m6A	1111707	+	GAYANNNNNNNRTAG	upstream Pnuc_1097, hypothetical protein		
m6A	1111717	-	CTAYNNNNNNNTRTC	Pnuc_1095, outer membrane autotransporter barrel domain protein		
m6A	1111938	+		upstream Pnuc_1097, hypothetical protein		
m6A	1115916	+	GAYANNNNNNNRTAG	upstream Pnuc_1097, hypothetical protein		
m6A	1115926	-	CTAYNNNNNNNTRTC	Pnuc_1095, outer membrane autotransporter barrel domain protein		
m4C	1118871	-		Pnuc_1095, outer membrane autotransporter barrel domain protein		
m6A	1118964	-		Pnuc_1095, outer membrane autotransporter barrel domain protein		
m4C	1119946	+		upstream Pnuc_1097, hypothetical protein		
m6A	1120460	+		upstream Pnuc_1097, hypothetical protein		
m4C	1120603	+		upstream Pnuc_1097, hypothetical protein		
m6A	1124249	+		upstream Pnuc_1097, hypothetical protein		
m6A	1124256	-		Pnuc_1095, outer membrane autotransporter barrel domain protein		
m4C	1126657	-		Pnuc_1095, outer membrane autotransporter barrel domain protein		
m4C	1131493	+		upstream Pnuc_1097, hypothetical protein		
m6A	1131887	+		upstream Pnuc_1097, hypothetical protein		
m6A	1134204	-		upstream Pnuc_1095, outer membrane autotransporter barrel domain protein		
m4C	1141529	+		upstream Pnuc_1101, protein of unknown function DUF583		
m6A	1141757	-		Pnuc_1098, methyltransferase FkbM family		
m4C	1144057	+		Pnuc_1101, protein of unknown function DUF583		
m6A	1146350	+		Pnuc_1104, nicotinate-nucleotide pyrophosphorylase(carboxylating)		
m6A	1147304	-		Pnuc_1105, TRAP transporter solute receptor, TAXI family		
m6A	1150445	+	GAYANNNNNNNRTAG	upstream Pnuc_1112, protein of unknown function DUF1080		
m6A	1150455	-	CTAYNNNNNNNTRTC	Pnuc_1107, transcriptional modulator of MazE/toxin, MazF		
m6A	1151487	+	GAYANNNNNNNRTAG	upstream Pnuc_1112, protein of unknown function DUF1080		
m6A	1151497	-	CTAYNNNNNNNTRTC	Pnuc_1109, NAD-dependent formate dehydrogenase iron-sulfur protein (catalytic activity)		
m4C	1152128	-		Pnuc_1109, NAD-dependent formate dehydrogenase iron-sulfur protein (catalytic activity)		
m6A	1156346	+	CTAYNNNNNNNTRTC	upstream Pnuc_1112, protein of unknown function DUF1080		
m6A	1156356	-	GAYANNNNNNNRTAG	Pnuc_1111, protein of unknown function DUF748		
m6A	1156357	+		upstream Pnuc_1112, protein of unknown function DUF1080		
m6A	1156484	-		Pnuc_1111, protein of unknown function DUF748		
m4C	1156774	-		Pnuc_1111, protein of unknown function DUF749		
m6A	1159304	+	GAYANNNNNNNRTAG	Pnuc_1112, protein of unknown function DUF1080		
m6A	1159314	-	CTAYNNNNNNNTRTC	upstream Pnuc_1111, protein of unknown function DUF748		
m6A	1159459	+		upstream Pnuc_1116, phage integrase family protein		
m6A	1163127	-		Pnuc_1118, SMC domain protein		
m4C	1164252	-		upstream Pnuc_1118, SMC domain protein		
m6A	1165285	-		Pnuc_1120, hypothetical protein		
m6A	1167044	+	CTAYNNNNNNNTRTC	Pnuc_1122, Exonuclease, RNase T and DNA polymerase III		
m6A	1167054	-	GAYANNNNNNNRTAG	upstream Pnuc_1121, hypothetical protein		
m4C	1169095	-		upstream Pnuc_1121, hypothetical protein		
m4C	1169873	+		upstream Pnuc_1127, hypothetical protein		
m4C	1170203	+		upstream Pnuc_1127, hypothetical protein		
m4C	1173732	+		upstream Pnuc_1127, hypothetical protein		

m6A	1173977	+	GAYANNNNNNNRTAG	Pnuc_1127, hypothetical protein		
m6A	1173987	-	CTAYNNNNNNNTRTC	upstream Pnuc_1126, hypothetical protein		
m6A	1177319	+	GAYANNNNNNNRTAG	upstream Pnuc_1131, phage transcriptional regulator, AlpA		
m6A	1177329	-	CTAYNNNNNNNTRTC	Pnuc_1130, protein of unknown function DUF1376		
m4C	1177539	+		upstream Pnuc_1131, phage transcriptional regulator, AlpA		
m6A	1178648	-		Pnuc_1132, restriction modification system DNA specificity domain protein		
m4C	1188747	+		upstream Pnuc_1139, fumarase		
m4C	1189031	+		upstream Pnuc_1139, fumarase		
m6A	1189221	-		upstream Pnuc_1138, acetyl-coenzyme A synthetase		
m4C	1189269	+		Pnuc_1139, fumarase		
m4C	1190609	+		Pnuc_1139, fumarase		
m6A	1191165	+	CTAYNNNNNNNTRTC	Pnuc_1140, glutamate racemase		
m6A	1191175	-	GAYANNNNNNNRTAG	upstream Pnuc_1138, acetyl-coenzyme A synthetase		
m4C	1193460	+		upstream Pnuc_1150, Penicillin amidase		
m4C	1194068	+		upstream Pnuc_1150, Penicillin amidase		
m4C	1195742	-		Pnuc_1146, phosphate ABC transporter substrate-binding protein, PhoT family		
m6A	1199870	+	GAYANNNNNNNRTAG	upstream Pnuc_1150, Penicillin amidase		
m6A	1199880	-	CTAYNNNNNNNTRTC	Pnuc_1149, filamentous hemagglutinin family outer membrane protein		
m4C	1200717	+		upstream Pnuc_1150, Penicillin amidase		
m4C	1202402	+		upstream Pnuc_1150, Penicillin amidase		
m4C	1204358	-		Pnuc_R0036, tRNA-Leu		
m4C	1204364	-		Pnuc_R0036, tRNA-Leu		
m6A	1206854	+	CTAYNNNNNNNTRTC	Pnuc_1150, Penicillin amidase		
m6A	1206864	-	GAYANNNNNNNRTAG	upstream Pnuc_R0036, tRNA-Leu		
m4C	1209983	-		Pnuc_1156, hypothetical protein		
m4C	1214543	+		Pnuc_1161, LrgB family protein		
m4C	1216992	+		Pnuc_1163, gamma-glutamyltransferase 2, Threonine peptidase, MEROPS family T03		
m6A	1218238	+	CTAYNNNNNNNTRTC	upstream Pnuc_1167, Cellulase		
m6A	1218248	-	GAYANNNNNNNRTAG	Pnuc_1164, conserved hypothetical protein		
m4C	1223679	+		Pnuc_1168, conserved hypothetical protein		
m6A	1223697	+		Pnuc_1168, conserved hypothetical protein		
m4C	1225020	+		upstream Pnuc_1176, hypothetical protein		
m4C	1225259	+		upstream Pnuc_1176, hypothetical protein		
m4C	1225329	-		Pnuc_1170, cellulose synthase operon C domain protein		
m4C	1225578	+		upstream Pnuc_1176, hypothetical protein		
m6A	1228000	+	GAYANNNNNNNRTAG	upstream Pnuc_1176, hypothetical protein		
m6A	1228010	-	CTAYNNNNNNNTRTC	Pnuc_1170, cellulose synthase operon C domain protein		
m6A	1228683	+	GAYANNNNNNNRTAG	upstream Pnuc_1176, hypothetical protein		
m6A	1228693	-	CTAYNNNNNNNTRTC	Pnuc_1170, cellulose synthase operon C domain protein		
m6A	1228990	+	CTAYNNNNNNNTRTC	upstream Pnuc_1176, hypothetical protein		
m6A	1229362	-		Pnuc_1171, Cellulose synthase (UDP-forming)		
m4C	1229846	+		upstream Pnuc_1176, hypothetical protein		
m4C	1229864	+		upstream Pnuc_1176, hypothetical protein		
m4C	1230976	-		Pnuc_1171, Cellulose synthase (UDP-forming)		

m4C	1233413	+		upstream Pnuc_1176, hypothetical protein		
m4C	1235766	+		upstream Pnuc_1184, Amidase		
m4C	1236389	+		upstream Pnuc_1184, Amidase		
m6A	1236712	+	CTAYNNNNNNNNTRTC	upstream Pnuc_1184, Amidase		
m6A	1236722	-	GAYANNNNNNNNRTAG	upstream Pnuc_1177, tRNA-U16,U17-dihydrouridine synthase		
m6A	1239968	+	GAYANNNNNNNNRTAG	upstream Pnuc_1184, Amidase		
m6A	1239978	-	CTAYNNNNNNNNTRTC	Pnuc_1180, putative ABC transporter, periplasmic protein		
m6A	1240127	+	GAYANNNNNNNNRTAG	upstream Pnuc_1184, Amidase		
m6A	1240137	-	CTAYNNNNNNNNTRTC	Pnuc_1180, putative ABC transporter, periplasmic protein		
m4C	1241022	+		upstream Pnuc_1184, Amidase		
m4C	1245515	-		upstream Pnuc_1183, conserved hypothetical protein		
m6A	1247968	+	CTAYNNNNNNNNTRTC	upstream Pnuc_1210, Glutamate synthase (NADPH)		
m6A	1247978	-	GAYANNNNNNNNRTAG	Pnuc_1190, urease accessory protein UreG		
m4C	1250367	-		Pnuc_1193, urease, Metallo peptidase, MEROPS family M38		
m4C	1251140	-		Pnuc_1193, urease, Metallo peptidase, MEROPS family M39		
m6A	1252457	+	GAYANNNNNNNNRTAG	upstream Pnuc_1210, Glutamate synthase (NADPH)		
m6A	1252467	-	CTAYNNNNNNNNTRTC	Pnuc_1196, urease, gamma subunit		
m6A	1253887	+		upstream Pnuc_1210, Glutamate synthase (NADPH)		
m4C	1260810	-		Pnuc_1203, secretory lipase		
m6A	1262425	+	CTAYNNNNNNNNTRTC	upstream Pnuc_1210, Glutamate synthase (NADPH)		
m6A	1262435	-	GAYANNNNNNNNRTAG	Pnuc_1205, protein of unknown function DUF1501		
m6A	1263897	-		Pnuc_1206, conserved hypothetical protein		
m4C	1266245	+		Pnuc_1210, Glutamate synthase (NADPH)		
m6A	1268677	+		upstream Pnuc_1218, transglutaminase, N-terminal domain protein		
m6A	1269593	+		upstream Pnuc_1218, transglutaminase, N-terminal domain protein		
m4C	1270788	-		Pnuc_1214, intracellular polyhydroxyalkanoate depolymerase		
m6A	1273368	-		upstream Pnuc_1217, Glutathione S-transferase, N-terminal domain protein		
m4C	1278459	-		upstream Pnuc_1217, Glutathione S-transferase, N-terminal domain protein		
m6A	1278858	-		upstream Pnuc_1217, Glutathione S-transferase, N-terminal domain protein		
m4C	1280244	-		Pnuc_1221, conserved hypothetical protein		
m4C	1281881	+		upstream Pnuc_1223, major facilitator superfamily MFS_1		
m4C	1287845	+		upstream Pnuc_1232, Patatin		
m4C	1289753	+		upstream Pnuc_1232, Patatin		
m4C	1291441	-		Pnuc_1229, protein of unknown function DUF150		
m6A	1292600	+		upstream Pnuc_1232, Patatin		
m4C	1294041	-		Pnuc_1231, condensin subunit ScpB		
m4C	1294062	-		Pnuc_1231, condensin subunit ScpB		
m4C	1295793	-		Pnuc_1233, NLP/P60 protein		
m6A	1296429	+	CTAYNNNNNNNNTRTC	upstream Pnuc_1238, Enoyl-[acyl-carrier-protein] reductase (NADH)		
m6A	1296439	-	GAYANNNNNNNNRTAG	upstream Pnuc_1233, NLP/P60 protein		
m6A	1296443	-		upstream Pnuc_1233, NLP/P60 protein		
m6A	1296502	-		Pnuc_1234, ABC transporter related protein		
m4C	1296561	+		upstream Pnuc_1238, Enoyl-[acyl-carrier-protein] reductase (NADH)		
m4C	1296881	+		upstream Pnuc_1238, Enoyl-[acyl-carrier-protein] reductase (NADH)		

m4C	1302181	-		upstream Pnuc_1237, extracellular solute-binding protein, family 5		
m4C	1302290	+		Pnuc_1238, Enoyl-[acyl-carrier-protein] reductase (NADH)		
m4C	1302491	+		Pnuc_1238, Enoyl-[acyl-carrier-protein] reductase (NADH)		
m4C	1302831	-		upstream Pnuc_1237, extracellular solute-binding protein, family 5		
m6A	1307149	+	GAYANNNNNNNRTAG	Pnuc_1242, MmgE/PrpD family protein		
m6A	1307159	-	CTAYNNNNNNNTRTC	upstream Pnuc_1237, extracellular solute-binding protein, family 5		
m4C	1309811	+		upstream Pnuc_1249, integrase, catalytic region		
m6A	1311023	-		Pnuc_1246, BNR/Asp-box repeat protein		
m6A	1311414	+	CTAYNNNNNNNTRTC	upstream Pnuc_1249, integrase, catalytic region		
m6A	1311424	-	GAYANNNNNNNRTAG	Pnuc_1246, BNR/Asp-box repeat protein		
m4C	1315036	-		upstream Pnuc_1248, hypothetical protein		
m6A	1317420	+	CTAYNNNNNNNTRTC	upstream Pnuc_1262, N-acetylglutamate synthase		
m6A	1317430	-	GAYANNNNNNNRTAG	Pnuc_1252, Exodeoxyribonuclease III		
m4C	1318116	+		upstream Pnuc_1262, N-acetylglutamate synthase		
m4C	1320245	-		Pnuc_1254, signal transduction histidine kinase, nitrogen specific, NtrB		
m6A	1322292	-		Pnuc_1256, molybdopterin binding domain protein		
m6A	1330103	+		upstream Pnuc_1262, N-acetylglutamate synthase		
m4C	1330799	+		Pnuc_1262, N-acetylglutamate synthase		
m4C	1330809	+		Pnuc_1262, N-acetylglutamate synthase		
m4C	1330848	-		upstream Pnuc_1261, ATP-dependent helicase HrpA		
m4C	1332282	-		upstream Pnuc_1261, ATP-dependent helicase HrpA		
m4C	1336306	+		upstream Pnuc_R0038, tRNA-Leu3		
m4C	1336698	-		Pnuc_1267, propionate CoA-transferase		
m4C	1337345	+		upstream Pnuc_R0038, tRNA-Leu3		
m4C	1337444	-		Pnuc_1267, propionate CoA-transferase		
m4C	1344160	+		Pnuc_1272, amino acid permease-associated region		
m4C	1349236	+		upstream Pnuc_1294, protein of unknown function UPF0005		
m4C	1353321	+		upstream Pnuc_1294, protein of unknown function UPF0005		
m4C	1356193	-		Pnuc_1284, protease FtsH subunit HflK		
m4C	1357004	+		upstream Pnuc_1294, protein of unknown function UPF0005		
m4C	1357054	+		upstream Pnuc_1294, protein of unknown function UPF0005		
m4C	1357572	-		upstream Pnuc_1285, GTP-binding protein HflX		
m4C	1357629	+		upstream Pnuc_1294, protein of unknown function UPF0005		
m4C	1358238	-		Pnuc_1287, small GTP-binding protein		
m4C	1358779	-		Pnuc_1287, small GTP-binding protein		
m4C	1358810	-		Pnuc_1287, small GTP-binding protein		
m4C	1367849	+		upstream Pnuc_1307, thioredoxin		
m4C	1372071	+		upstream Pnuc_1307, thioredoxin		
m6A	1373052	+		upstream Pnuc_1307, thioredoxin		
m6A	1373136	+		upstream Pnuc_1307, thioredoxin		
m4C	1375771	-		Pnuc_1302, outer membrane efflux protein		
m4C	1376040	+		upstream Pnuc_1307, thioredoxin		
m4C	1376075	+		upstream Pnuc_1307, thioredoxin		
m4C	1377069	-		Pnuc_1302, outer membrane efflux protein		

m6A	1377692	+		upstream Pnuc_1307, thioredoxin		
m4C	1379860	+		upstream Pnuc_1307, thioredoxin		
m4C	1380179	+		upstream Pnuc_1307, thioredoxin		
m4C	1383112	+		upstream Pnuc_1307, thioredoxin		
m4C	1383881	-		Pnuc_1306, DNA helicase/exodeoxyribonuclease V, subunit B		
m6A	1384156	+		upstream Pnuc_1307, thioredoxin		
m4C	1387718	-		upstream Pnuc_1306, DNA helicase/exodeoxyribonuclease V, subunit B		
m6A	1387744	-		upstream Pnuc_1306, DNA helicase/exodeoxyribonuclease V, subunit B		
m4C	1387948	+		Pnuc_1309, LSU ribosomal protein L31P		
m4C	1391088	-		upstream Pnuc_1306, DNA helicase/exodeoxyribonuclease V, subunit B		
m4C	1391890	+		upstream Pnuc_1318, Enoyl-CoA hydratase/isomerase		
m4C	1392764	+		upstream Pnuc_1318, Enoyl-CoA hydratase/isomerase		
m4C	1393403	-		Pnuc_1314, conserved hypothetical protein		
m6A	1393669	-		Pnuc_1315, conserved hypothetical protein		
m4C	1396783	+		upstream Pnuc_1318, Enoyl-CoA hydratase/isomerase		
m6A	1399985	-		Pnuc_1321, fumarylacetoacetate (FAA) hydrolase		
m4C	1400775	+		Pnuc_1323, Uncharacterized protein UPF0065		
m4C	1405924	-		Pnuc_1328, 5-oxopent-3-ene-1,2,5-tricarboxylate decarboxylase		
m6A	1406779	-		Pnuc_1329, ferrous iron transport protein B		
m6A	1407240	+		upstream Pnuc_1331, Carbohydrate-selective porin OprB		
m6A	1410626	+	GAYANNNNNNNNRTAG	Pnuc_1332, negative transcriptional regulator		
m6A	1410636	-	CTAYNNNNNNNNTRTC	upstream Pnuc_1330, FeoA family protein		
m6A	1412582	+		upstream Pnuc_1336, putative sulfate transport system substrate-binding protein		
m6A	1416244	+		upstream Pnuc_1339, GCN5-related N-acetyltransferase		
m6A	1416333	+		Pnuc_1339, GCN5-related N-acetyltransferase		
m6A	1416762	+	CTAYNNNNNNNNTRTC	Pnuc_1340, thioesterase superfamily protein		
m6A	1416772	-	GAYANNNNNNNNRTAG	upstream Pnuc_1338, hypothetical protein		
m6A	1417579	+	GAYANNNNNNNNRTAG	Pnuc_1341, conserved hypothetical protein		
m6A	1417589	-	CTAYNNNNNNNNTRTC	upstream Pnuc_1338, hypothetical protein		
m4C	1419056	+		Pnuc_1342, DEAD/DEAH box helicase domain protein		
m6A	1419263	+	CTAYNNNNNNNNTRTC	Pnuc_1343, transglutaminase domain protein		
m6A	1419273	-	GAYANNNNNNNNRTAG	upstream Pnuc_1338, hypothetical protein		
m6A	1419805	+		Pnuc_1343, transglutaminase domain protein		
m4C	1419935	-		upstream Pnuc_1338, hypothetical protein		
m6A	1420785	-		upstream Pnuc_1338, hypothetical protein		
m6A	1421798	-		upstream Pnuc_1338, hypothetical protein		
m6A	1422263	+	CTAYNNNNNNNNTRTC	upstream Pnuc_1347, protein of unknown function DUF1330		
m6A	1422273	-	GAYANNNNNNNNRTAG	upstream Pnuc_1346, conserved hypothetical protein		
m6A	1422850	+		upstream Pnuc_1349, Uncharacterized protein UPF0065		
m4C	1427357	+		Pnuc_1353, hypothetical protein		
m4C	1429892	+		upstream Pnuc_1360, hypothetical protein		
m6A	1430406	-		Pnuc_1357, HI0933 family protein		
m4C	1433867	+		upstream Pnuc_1363, carbohydrate kinase, YjeF related protein		
m6A	1434445	+	CTAYNNNNNNNNTRTC	upstream Pnuc_1363, carbohydrate kinase, YjeF related protein		

m6A	1434455	-	GAYANNNNNNNRTAG	Pnuc_1362, RNA-metabolising metallo-beta-lactamase
m6A	1436527	+		Pnuc_1364, protein of unknown function DUF6, transmembrane
m4C	1438928	-		Pnuc_1368, Uncharacterized protein UPF0065
m4C	1440052	+		upstream Pnuc_1371, L-lactate dehydrogenase (cytochrome)
m4C	1444140	-		upstream Pnuc_1370, protein of unknown function DUF6, transmembrane
m6A	1444335	-		upstream Pnuc_1370, protein of unknown function DUF6, transmembrane
m6A	1460924	+		upstream Pnuc_1392, binding-protein-dependent transport systems inner membrane component
m4C	1461233	-		Pnuc_1390, cardiolipin synthetase 2
m4C	1462691	+		upstream Pnuc_1392, binding-protein-dependent transport systems inner membrane component
m6A	1463623	-		upstream Pnuc_1391, Endonuclease/exonuclease/phosphatase
m6A	1464729	+	GAYANNNNNNNRTAG	upstream Pnuc_1407, heavy metal translocating P-type ATPase
m6A	1464739	-	CTAYNNNNNNNTRTC	Pnuc_1395, formate dehydrogenase gamma subunit
m4C	1468422	-		Pnuc_1398, molybdopterin oxidoreductase
m4C	1470425	+		upstream Pnuc_1407, heavy metal translocating P-type ATPase
m4C	1470899	+		upstream Pnuc_1407, heavy metal translocating P-type ATPase
m6A	1471413	-		Pnuc_1400, 4Fe-4S ferredoxin, iron-sulfur binding domain protein
m6A	1471884	+		upstream Pnuc_1407, heavy metal translocating P-type ATPase
m6A	1473559	+		upstream Pnuc_1407, heavy metal translocating P-type ATPase
m6A	1473645	+	GAYANNNNNNNRTAG	upstream Pnuc_1407, heavy metal translocating P-type ATPase
m6A	1473655	-	CTAYNNNNNNNTRTC	Pnuc_1402, conserved hypothetical protein
m4C	1473939	-		Pnuc_1403, protein of unknown function DUF59
m4C	1475423	+		upstream Pnuc_1407, heavy metal translocating P-type ATPase
m4C	1475449	-		Pnuc_1404, phosphate transporter
m4C	1477177	+		Pnuc_1407, heavy metal translocating P-type ATPase
m6A	1478429	-		upstream Pnuc_1406, Heavy metal transport/detoxification protein
m4C	1483414	-		upstream Pnuc_1408, conserved hypothetical protein
m6A	1483806	-		upstream Pnuc_1408, conserved hypothetical protein
m4C	1484502	-		upstream Pnuc_1408, conserved hypothetical protein
m6A	1485343	+		Pnuc_1413, protein of unknown function DUF519
m4C	1488371	-		Pnuc_1418, Radical SAM domain protein
m6A	1488814	+		upstream Pnuc_1426, SsrA-binding protein
m4C	1493575	-		Pnuc_1422, GMP synthase (glutamine-hydrolyzing)
m4C	1495693	-		Pnuc_1424, protein of unknown function UPF0125
m6A	1496168	+	CTAYNNNNNNNTRTC	upstream Pnuc_1426, SsrA-binding protein
m6A	1496178	-	GAYANNNNNNNRTAG	Pnuc_1425, cyclase/dehydrase
m4C	1496785	-		upstream Pnuc_1425, cyclase/dehydrase
m4C	1498734	+		Pnuc_1428, protein of unknown function DUF403
m4C	1498747	+		Pnuc_1428, protein of unknown function DUF403
m4C	1500795	-		upstream Pnuc_1425, cyclase/dehydrase
m4C	1502448	+		Pnuc_1432, Integrase, catalytic region
m4C	1503975	-		Pnuc_1433, phosphoenolpyruvate synthase
m6A	1505598	+	CTAYNNNNNNNTRTC	Pnuc_1434, protein of unknown function DUF299
m6A	1505608	-	GAYANNNNNNNRTAG	Pnuc_1433, phosphoenolpyruvate synthase
m4C	1510301	+		upstream Pnuc_1452, methionine aminopeptidase, type I

m6A	1519356	+	CTAYNNNNNNNTRTC	upstream Pnuc_1452, methionine aminopeptidase, type I	
m6A	1519366	-	GAYANNNNNNNRTAG	Pnuc_1448, ribosome recycling factor	
m4C	1522785	+		Pnuc_1452, methionine aminopeptidase, type I	
m4C	1523178	+		Pnuc_1452, methionine aminopeptidase, type I	
m6A	1526556	+		upstream Pnuc_1458, 2,3,4,5-tetrahydropyridine-2,6-dicarboxylate N-succinyltransferase	
m4C	1529296	-		Pnuc_1456, ZipA, C-terminal FtsZ-binding region	
m4C	1530031	+		upstream Pnuc_1458, 2,3,4,5-tetrahydropyridine-2,6-dicarboxylate N-succinyltransferase	
m4C	1530327	+		upstream Pnuc_1458, 2,3,4,5-tetrahydropyridine-2,6-dicarboxylate N-succinyltransferase	
m4C	1530420	+		upstream Pnuc_1458, 2,3,4,5-tetrahydropyridine-2,6-dicarboxylate N-succinyltransferase	
m6A	1530582	-		Pnuc_1457, chromosome segregation protein SMC	
m6A	1532841	-		Pnuc_1457, chromosome segregation protein SMC	
m4C	1537844	+		upstream Pnuc_R0039, tRNA-Met1	
m4C	1538994	+		upstream Pnuc_R0039, tRNA-Met1	
m6A	1540249	+		upstream Pnuc_R0039, tRNA-Met1	
m4C	1541125	-		Pnuc_1465, DNA internalization-related competence protein ComEC/Rec2	
m4C	1544837	-		Pnuc_1467, ABC transporter related protein	
m6A	1550047	+		Pnuc_1471, bacterial peptide chain release factor 2(bRF-2)	
m6A	1550543	+		Pnuc_1472, lysyl-tRNA synthetase	
m4C	1551848	+		upstream Pnuc_1477, peptide deformylase	
m6A	1552414	+	GAYANNNNNNNRTAG	upstream Pnuc_1477, peptide deformylase	
m6A	1552424	-	CTAYNNNNNNNTRTC	Pnuc_1473, hypothetical protein	
m6A	1554164	-		Pnuc_1476, cysteine synthase A	
m6A	1557612	+	CTAYNNNNNNNTRTC	Pnuc_1480, protein of unknown function DUF81	
m6A	1557622	-	GAYANNNNNNNRTAG	upstream Pnuc_1478, disulfide bond formation protein DsbB	
m4C	1565765	-		Pnuc_1489, Fe-S protein assembly chaperone HscA	
m4C	1566604	-		Pnuc_1489, Fe-S protein assembly chaperone HscA	
m4C	1566777	+		upstream Pnuc_1496, aminotransferase	
m4C	1568086	+		upstream Pnuc_1496, aminotransferase	
m4C	1568266	-		upstream Pnuc_1492, FeS cluster assembly scaffold IscU	
m6A	1568411	+		upstream Pnuc_1496, aminotransferase	
m6A	1569027	+		upstream Pnuc_1496, aminotransferase	
m4C	1569924	-		Pnuc_1494, transcriptional regulator, BadM/Rrf2 family	
m4C	1570822	-		Pnuc_1495, Excinuclease ABC subunit B	
m6A	1570842	+	CTAYNNNNNNNTRTC	upstream Pnuc_1496, aminotransferase	
m6A	1570852	-	GAYANNNNNNNRTAG	Pnuc_1495, Excinuclease ABC subunit B	
m4C	1571584	-		Pnuc_1495, Excinuclease ABC subunit B	
m4C	1572033	-		Pnuc_1495, Excinuclease ABC subunit B	
m4C	1574196	-		upstream Pnuc_1495, Excinuclease ABC subunit B	
m4C	1575410	+		Pnuc_1498, electron transport complex, RnfABCDGE type, B subunit	
m4C	1578103	+		upstream Pnuc_1528, conserved hypothetical protein	
m4C	1579573	-		Pnuc_1504, 3-oxoadipate CoA-transferase alpha subunit	
m6A	1580069	+		upstream Pnuc_1528, conserved hypothetical protein	
m4C	1581325	+		upstream Pnuc_1528, conserved hypothetical protein	
m6A	1582960	+	GAYANNNNNNNRTAG	upstream Pnuc_1528, conserved hypothetical protein	

m6A	1582970	-	CTAYNNNNNNNTRTC	Pnuc_1507, major facilitator superfamily MFS_1
m4C	1583023	-		Pnuc_1507, major facilitator superfamily MFS_1
m4C	1586886	+		upstream Pnuc_1528, conserved hypothetical protein
m4C	1587174	-		Pnuc_1511, cytochrome C biogenesis protein
m4C	1588023	-		Pnuc_1513, cytochrome c-type biogenesis protein CcmF
m4C	1588814	-		upstream Pnuc_1513, cytochrome c-type biogenesis protein CcmF
m4C	1591196	-		Pnuc_1517, heme exporter protein CcmB
m4C	1591513	-		Pnuc_1517, heme exporter protein CcmB
m6A	1591897	+	CTAYNNNNNNNTRTC	upstream Pnuc_1528, conserved hypothetical protein
m6A	1591907	-	GAYANNNNNNNRTAG	Pnuc_1518, heme exporter protein CcmA
m4C	1592173	+		upstream Pnuc_1528, conserved hypothetical protein
m4C	1595365	+		upstream Pnuc_1528, conserved hypothetical protein
m6A	1595390	-		upstream Pnuc_1523, transcriptional regulator, MarR family
m4C	1595553	-		Pnuc_1524, beta-lactamase domain protein
m4C	1595665	-		Pnuc_1524, beta-lactamase domain protein
m4C	1595861	-		Pnuc_1524, beta-lactamase domain protein
m6A	1596988	+		upstream Pnuc_1528, conserved hypothetical protein
m4C	1598464	+		upstream Pnuc_1528, conserved hypothetical protein
m6A	1599496	+	CTAYNNNNNNNTRTC	Pnuc_1529, histone deacetylase superfamily
m6A	1599506	-	GAYANNNNNNNRTAG	upstream Pnuc_1527, hypothetical protein
m6A	1601203	+	GAYANNNNNNNRTAG	upstream Pnuc_1534, alkyl hydroperoxide reductase/ Thiol specific antioxidant/ Mal allergen
m6A	1601213	-	CTAYNNNNNNNTRTC	Pnuc_1531, putative lipoprotein
m6A	1602318	-		Pnuc_1533, hypothetical protein
m4C	1602809	+		upstream Pnuc_1534, alkyl hydroperoxide reductase/ Thiol specific antioxidant/ Mal allergen
m4C	1605257	+		Pnuc_1535, FAD-dependent pyridine nucleotide-disulfide oxidoreductase
m4C	1606133	+		upstream Pnuc_1544, transcriptional regulator, GntR family
m6A	1606433	+	GAYANNNNNNNRTAG	upstream Pnuc_1544, transcriptional regulator, GntR family
m6A	1606443	-	CTAYNNNNNNNTRTC	Pnuc_1538, TRAP C4-dicarboxylate transport system permease DctM subunit
m4C	1606519	-		Pnuc_1538, TRAP C4-dicarboxylate transport system permease DctM subunit
m6A	1606979	-		Pnuc_1538, TRAP C4-dicarboxylate transport system permease DctM subunit
m4C	1608288	-		Pnuc_1538, TRAP C4-dicarboxylate transport system permease DctM subunit
m4C	1609032	+		upstream Pnuc_1544, transcriptional regulator, GntR family
m4C	1611649	-		Pnuc_1542, ferredoxin
m4C	1615808	+		Pnuc_1546, transcriptional regulator, LysR family
m4C	1616536	+		Pnuc_1546, transcriptional regulator, LysR family
m6A	1617388	+	GAYANNNNNNNRTAG	Pnuc_1547, protein of unknown function DUF6, transmembrane
m6A	1617398	-	CTAYNNNNNNNTRTC	upstream Pnuc_1545, class II aldolase/adducin family protein
m4C	1619064	-		Pnuc_1549, amino acid/amide ABC transporter substrate-binding protein, HAAT family
m4C	1619386	+		upstream Pnuc_1553, transcriptional regulator, AsnC family
m6A	1622660	+		upstream Pnuc_1553, transcriptional regulator, AsnC family
m4C	1625078	+		upstream Pnuc_1553, transcriptional regulator, AsnC family
m4C	1627175	+		upstream Pnuc_1560, transcriptional regulator, LysR family
m6A	1628517	+	GAYANNNNNNNRTAG	upstream Pnuc_1560, transcriptional regulator, LysR family
m6A	1628527	-	CTAYNNNNNNNTRTC	Pnuc_1556, Enoyl-CoA hydratase/isomerase

m4C	1630294	+		upstream Pnuc_1560, transcriptional regulator, LysR family		
m4C	1635495	-		upstream Pnuc_1561, major facilitator superfamily MFS_1		
m6A	1638119	+	CTAYNNNNNNNNTRTC	Pnuc_1564, FMN-binding domain protein		
m6A	1638129	-	GAYANNNNNNNNRTAG	upstream Pnuc_1563, conserved hypothetical protein		
m4C	1645105	+		upstream Pnuc_1581, hypothetical protein		
m4C	1646478	+		upstream Pnuc_1581, hypothetical protein		
m4C	1647150	-		Pnuc_1574, Acetamidase/Formamidase		
m4C	1648753	-		Pnuc_1576, penicillin amidase, Cysteine peptidase, MEROPS family C59		
m4C	1649413	+		upstream Pnuc_1581, hypothetical protein		
m4C	1653477	-		upstream Pnuc_1580, transcriptional regulator, ArsR family		
m6A	1654685	+		upstream Pnuc_1585, metallophosphoesterase		
m6A	1656328	+		Pnuc_1586, glycosyl transferase, group 1		
m6A	1658012	+	GAYANNNNNNNNRTAG	Pnuc_1588, ornithine-acyl[acyl carrier protein]N-acyltransferase		
m6A	1658022	-	CTAYNNNNNNNNTRTC	upstream Pnuc_1584, lyso-ornithine lipid acyltransferase		
m6A	1660045	+		upstream Pnuc_1595, MgtC/SapB transporter		
m4C	1660384	+		upstream Pnuc_1595, MgtC/SapB transporter		
m6A	1660810	+	CTAYNNNNNNNNTRTC	upstream Pnuc_1595, MgtC/SapB transporter		
m6A	1660820	-	GAYANNNNNNNNRTAG	Pnuc_1591, MgtC/SapB transporter		
m4C	1663965	-		Pnuc_1593, conserved hypothetical protein		
m4C	1665691	+		Pnuc_1595, MgtC/SapB transporter		
m4C	1667725	+		upstream Pnuc_1606, short-chain dehydrogenase/reductase SDR		
m4C	1672413	+		upstream Pnuc_1606, short-chain dehydrogenase/reductase SDR		
m4C	1673396	+		upstream Pnuc_1606, short-chain dehydrogenase/reductase SDR		
m4C	1675386	-		Pnuc_1607, conserved hypothetical protein		
m4C	1683718	+		upstream Pnuc_1622, hypothetical protein		
m6A	1685160	-		Pnuc_1616, malate-quinone oxidoreductase		
m4C	1685584	-		Pnuc_1617, conserved hypothetical protein		
m4C	1687143	-		Pnuc_1618, fumarase		
m6A	1690825	-		upstream Pnuc_1621, Mg2+ transporter protein, CorA family protein		
m4C	1692780	-		Pnuc_1624, Nitrilase/cyanide hydratase and apolipoprotein N-acyltransferase		
m4C	1696150	+		upstream Pnuc_1636, transcriptional regulator, GntR family		
m4C	1698796	+		upstream Pnuc_1636, transcriptional regulator, GntR family		
m6A	1699838	+	CTAYNNNNNNNNTRTC	upstream Pnuc_1636, transcriptional regulator, GntR family		
m4C	1700632	+		upstream Pnuc_1636, transcriptional regulator, GntR family		
m4C	1701764	+		upstream Pnuc_1636, transcriptional regulator, GntR family		
m6A	1705954	+	CTAYNNNNNNNNTRTC	Pnuc_1638, protein of unknown function DUF6, transmembrane		
m6A	1705964	-	GAYANNNNNNNNRTAG	upstream Pnuc_1637, NAD-dependent epimerase/dehydratase		
m6A	1708596	-		upstream Pnuc_1641, conserved hypothetical protein		
m6A	1712078	+	CTAYNNNNNNNNTRTC	upstream Pnuc_1649, hypothetical protein		
m6A	1712088	-	GAYANNNNNNNNRTAG	Pnuc_1647, DNA polymerase III, epsilon subunit		
m6A	1712327	-		Pnuc_1647, DNA polymerase III, epsilon subunit		
m6A	1712464	-		Pnuc_1647, DNA polymerase III, epsilon subunit		
m6A	1712822	+	CTAYNNNNNNNNTRTC	upstream Pnuc_1649, hypothetical protein		
m6A	1712832	-	GAYANNNNNNNNRTAG	Pnuc_1647, DNA polymerase III, epsilon subunit		

m4C	1713392	-		Pnuc_1647, DNA polymerase III, epsilon subunit		
m4C	1715805	-		upstream Pnuc_1648, transcriptional regulator, BadM/Rrf2 family		
m6A	1716381	-		upstream Pnuc_1648, transcriptional regulator, BadM/Rrf2 family		
m4C	1717994	-		upstream Pnuc_1653, membrane-associated protein in eicosanoid and glutathione metabolism (MAPEG)		
m6A	1719842	-		upstream Pnuc_1653, membrane-associated protein in eicosanoid and glutathione metabolism (MAPEG)		
m4C	1731126	+		upstream Pnuc_1678, putative transcriptional regulator, MerR family		
m4C	1734371	+		upstream Pnuc_1678, putative transcriptional regulator, MerR family		
m6A	1734414	+		upstream Pnuc_1678, putative transcriptional regulator, MerR family		
m4C	1734534	-		Pnuc_1669, K+-transporting ATPase, B subunit		
m6A	1736959	+	GAYANNNNNNNRTAG	upstream Pnuc_1678, putative transcriptional regulator, MerR family		
m6A	1736969	-	CTAYNNNNNNNTRTC	Pnuc_1670, potassium-transporting ATPase, A subunit		
m6A	1738403	+	CTAYNNNNNNNTRTC	upstream Pnuc_1678, putative transcriptional regulator, MerR family		
m6A	1738413	-	GAYANNNNNNNRTAG	Pnuc_1672, Heavy metal transport/detoxification protein		
m4C	1739306	-		Pnuc_1673, conserved hypothetical protein		
m4C	1740979	+		upstream Pnuc_1678, putative transcriptional regulator, MerR family		
m4C	1742637	+		upstream Pnuc_1678, putative transcriptional regulator, MerR family		
m4C	1744638	-		Pnuc_1679, conserved hypothetical protein		
m4C	1748401	+		upstream Pnuc_1688, hypothetical protein		
m6A	1748827	+	GAYANNNNNNNRTAG	upstream Pnuc_1688, hypothetical protein		
m6A	1748837	-	CTAYNNNNNNNTRTC	Pnuc_1681, efflux transporter, RND family, MFP subunit		
m4C	1754380	-		Pnuc_1689, phage integrase family protein		
m4C	1756634	+		Pnuc_1692, hypothetical protein		
m6A	1758014	+	GAYANNNNNNNRTAG	upstream Pnuc_1702, O-sialoglycoprotein endopeptidase		
m4C	1758023	-		Pnuc_1694, phage integrase family protein		
m4C	1760544	-		Pnuc_1696, hypothetical protein		
m4C	1760689	+		upstream Pnuc_1702, O-sialoglycoprotein endopeptidase		
m6A	1764285	-		Pnuc_1698, RNA polymerase, sigma 70 subunit, RpoD		
m4C	1765765	-		Pnuc_1699, DNA primase		
m4C	1769633	+		upstream Pnuc_1707, Rieske (2Fe-2S) domain protein		
m6A	1770001	-		Pnuc_1703, GTP cyclohydrolase I		
m4C	1772603	+		upstream Pnuc_1707, Rieske (2Fe-2S) domain protein		
m4C	1773113	+		upstream Pnuc_1707, Rieske (2Fe-2S) domain protein		
m4C	1773158	+		upstream Pnuc_1707, Rieske (2Fe-2S) domain protein		
m4C	1773375	+		upstream Pnuc_1707, Rieske (2Fe-2S) domain protein		
m6A	1774091	+		Pnuc_1707, Rieske (2Fe-2S) domain protein		
m4C	1775687	-		Pnuc_1709, zinc/iron permease		
m4C	1778142	+		upstream Pnuc_1712, putative transmembrane protein		
m4C	1779168	-		Pnuc_1711, DNA polymerase I		
m4C	1782990	-		Pnuc_1715, ATP-dependent DNA helicase UvrD		
m6A	1785209	-		upstream Pnuc_1715, ATP-dependent DNA helicase UvrD		
m4C	1785614	+		Pnuc_1716, valyl-tRNA synthetase		
m4C	1788121	-		upstream Pnuc_1715, ATP-dependent DNA helicase UvrD		
m4C	1790241	+		upstream Pnuc_1720, protein of unknown function DUF1468		
m4C	1792420	-		upstream Pnuc_1719, alanyl-tRNA synthetase		

m6A	1792900	-		upstream Pnuc_1719, alanyl-tRNA synthetase		
m4C	1793918	-		upstream Pnuc_1719, alanyl-tRNA synthetase		
m6A	1797655	+		Pnuc_1725, acetylornithine aminotransferase apoenzyme		
m6A	1797750	+	CTAYNNNNNNNNTRTC	Pnuc_1725, acetylornithine aminotransferase apoenzyme		
m6A	1797760	-	GAYANNNNNNNNRTAG	upstream Pnuc_1723, NAD-dependent epimerase/dehydratase		
m4C	1797852	+		Pnuc_1725, acetylornithine aminotransferase apoenzyme		
m4C	1800152	-		Pnuc_1727, amino acid/amide ABC transporter ATP-binding protein 1, HAAT family		
m6A	1804734	+		upstream Pnuc_1733, DNA replication and repair protein RadC		
m4C	1805763	-		upstream Pnuc_1732, peptidylprolyl isomerase, FKBP-type		
m6A	1806482	-		Pnuc_1736, fatty acid desaturase		
m6A	1808144	+		upstream Pnuc_1739, FMN adenylyltransferase / riboflavin kinase		
m4C	1809413	-		Pnuc_1738, formyltetrahydrofolate-dependent phosphoribosylglycinamide formyltransferase		
m4C	1816551	+		upstream Pnuc_1750, cold-shock DNA-binding protein family		
m6A	1821620	-		Pnuc_1748, ATP-dependent Clp protease ATP-binding subunit ClpA		
m4C	1823682	-		Pnuc_1753, argininosuccinate synthase		
m4C	1824982	-		Pnuc_1754, ornithine carbamoyltransferase		
m4C	1827467	-		upstream Pnuc_1756, SSU ribosomal protein S20P		
m6A	1829138	-		upstream Pnuc_1756, SSU ribosomal protein S20P		
m4C	1832132	-		upstream Pnuc_1761, phosphoribosylformylglycinamidine cyclo-ligase		
m6A	1832665	+	CTAYNNNNNNNNTRTC	Pnuc_1762, protein of unknown function UPF0118		
m6A	1832675	-	GAYANNNNNNNNRTAG	upstream Pnuc_1761, phosphoribosylformylglycinamidine cyclo-ligase		
m6A	1832846	-		upstream Pnuc_1761, phosphoribosylformylglycinamidine cyclo-ligase		
m6A	1835641	+		Pnuc_1765, poly(A) polymerase		
m4C	1835657	-		upstream Pnuc_1761, phosphoribosylformylglycinamidine cyclo-ligase		
m6A	1836536	+		Pnuc_1766, 2-amino-4-hydroxy-6-hydroxymethyldihydropteridine pyrophosphokinase		
m4C	1836846	-		upstream Pnuc_1761, phosphoribosylformylglycinamidine cyclo-ligase		
m4C	1837887	-		Pnuc_1768, chaperone protein DnaJ		
m4C	1838959	+		upstream Pnuc_1773, NAD(+) kinase		
m4C	1839179	-		Pnuc_1769, chaperone protein DnaK		
m4C	1840063	+		upstream Pnuc_1773, NAD(+) kinase		
m4C	1840629	+		upstream Pnuc_1773, NAD(+) kinase		
m4C	1842862	-		Pnuc_1772, heat-inducible transcription repressor HrcA		
m6A	1843285	+	GAYANNNNNNNNRTAG	upstream Pnuc_1773, NAD(+) kinase		
m6A	1843295	-	CTAYNNNNNNNNTRTC	Pnuc_1772, heat-inducible transcription repressor HrcA		
m4C	1846249	+		upstream Pnuc_1776, membrane protein-like protein		
m4C	1849483	-		upstream Pnuc_1775, (Glutamate-ammonia-ligase) adenylyltransferase		
m6A	1852400	+		upstream Pnuc_1776, membrane protein-like protein		
m6A	1853226	+	CTAYNNNNNNNNTRTC	Pnuc_1777, Nitrilase/cyanide hydratase and apolipoprotein N-acyltransferase		
m6A	1853236	-	GAYANNNNNNNNRTAG	upstream Pnuc_1775, (Glutamate-ammonia-ligase) adenylyltransferase		
m4C	1855577	-		upstream Pnuc_1775, (Glutamate-ammonia-ligase) adenylyltransferase		
m6A	1861824	+	GAYANNNNNNNNRTAG	Pnuc_1784, alanine racemase domain protein		
m6A	1861834	-	CTAYNNNNNNNNTRTC	upstream Pnuc_1780, ATP:cob(I)alamin adenosyltransferase		
m4C	1862042	-		upstream Pnuc_1780, ATP:cob(I)alamin adenosyltransferase		
m6A	1862177	-		upstream Pnuc_1780, ATP:cob(I)alamin adenosyltransferase		

m4C	1864988	+		upstream Pnuc_1788, S-adenosylmethionine--tRNA-ribosyltransferase-isomerase		
m6A	1865372	+	GAYANNNNNNNRTAG	upstream Pnuc_1788, S-adenosylmethionine--tRNA-ribosyltransferase-isomerase		
m6A	1865382	-	CTAYNNNNNNNTRTC	Pnuc_1787, ATP-dependent DNA helicase RecG		
m6A	1865725	+		upstream Pnuc_1788, S-adenosylmethionine--tRNA-ribosyltransferase-isomerase		
m6A	1867542	+	CTAYNNNNNNNTRTC	Pnuc_1789, tRNA-guanine transglycosylase		
m6A	1867552	-	GAYANNNNNNNRTAG	upstream Pnuc_1787, ATP-dependent DNA helicase RecG		
m4C	1867847	-		upstream Pnuc_1787, ATP-dependent DNA helicase RecG		
m4C	1868106	-		upstream Pnuc_1787, ATP-dependent DNA helicase RecG		
m6A	1868132	+		upstream Pnuc_1790, protein translocase subunit yajC		
m4C	1874377	-		upstream Pnuc_1794, Adenylosuccinate lyase		
m4C	1874784	-		upstream Pnuc_1794, Adenylosuccinate lyase		
m6A	1874872	+	GAYANNNNNNNRTAG	upstream Pnuc_1797, alanine dehydrogenase/PNT domain protein		
m6A	1874882	-	CTAYNNNNNNNTRTC	Pnuc_1796, tRNA (5-methylaminomethyl-2-thiouridylate)-methyltransferase		
m6A	1880808	+	GAYANNNNNNNRTAG	upstream Pnuc_1802, hypothetical protein		
m6A	1880818	-	CTAYNNNNNNNTRTC	Pnuc_1801, glycosyl transferase, family 11		
m4C	1881748	+		upstream Pnuc_1802, hypothetical protein		
m6A	1881762	+	CTAYNNNNNNNTRTC	upstream Pnuc_1802, hypothetical protein		
m6A	1881772	-	GAYANNNNNNNRTAG	upstream Pnuc_1801, glycosyl transferase, family 11		
m6A	1883472	+		upstream Pnuc_1807, putative diguanylate phosphodiesterase		
m4C	1884110	-		Pnuc_1805, chaperonin GroEL		
m4C	1886632	+		Pnuc_1808, two component transcriptional regulator, LuxR family		
m4C	1887063	-		upstream Pnuc_1805, chaperonin GroEL		
m4C	1891141	-		upstream Pnuc_1806, chaperonin Cpn10		
m4C	1893231	+		upstream Pnuc_1818, branched chain amino acid aminotransferase apoenzyme		
m4C	1894041	+		upstream Pnuc_1818, branched chain amino acid aminotransferase apoenzyme		
m6A	1894804	+	GAYANNNNNNNRTAG	upstream Pnuc_1818, branched chain amino acid aminotransferase apoenzyme		
m6A	1894814	-	CTAYNNNNNNNTRTC	Pnuc_1814, phosphoribosylaminoimidazole-succinocarboxamide synthase		
m4C	1895880	+		upstream Pnuc_1818, branched chain amino acid aminotransferase apoenzyme		
m4C	1895901	+		upstream Pnuc_1818, branched chain amino acid aminotransferase apoenzyme		
m6A	1896432	+		upstream Pnuc_1818, branched chain amino acid aminotransferase apoenzyme		
m4C	1898142	+		upstream Pnuc_1818, branched chain amino acid aminotransferase apoenzyme		
m4C	1898342	-		Pnuc_1817, phosphoglycerate kinase		
m6A	1899165	+	CTAYNNNNNNNTRTC	Pnuc_1818, branched chain amino acid aminotransferase apoenzyme		
m6A	1899175	-	GAYANNNNNNNRTAG	upstream Pnuc_1817, phosphoglycerate kinase		
m4C	1899601	-		upstream Pnuc_1817, phosphoglycerate kinase		
m4C	1902681	+		upstream Pnuc_1824, GTP cyclohydrolase subunit MoaC		
m4C	1903168	+		upstream Pnuc_1824, GTP cyclohydrolase subunit MoaC		
m4C	1906490	-		upstream Pnuc_1825, TPR repeat-containing protein		
m4C	1907280	+		upstream Pnuc_1835, hypothetical protein		
m6A	1908212	-		Pnuc_1827, TPR repeat-containing protein		
m6A	1910712	+		upstream Pnuc_1835, hypothetical protein		
m4C	1910853	-		upstream Pnuc_1829, Integral membrane protein TerC		
m4C	1911260	+		upstream Pnuc_1835, hypothetical protein		
m4C	1911642	+		upstream Pnuc_1835, hypothetical protein		

m4C	1913021	+		upstream Pnuc_1835, hypothetical protein		
m6A	1915736	+		Pnuc_1835, hypothetical protein		
m6A	1916434	+	GAYANNNNNNNRTAG	Pnuc_1835, hypothetical protein		
m6A	1916444	-	CTAYNNNNNNNTRTC	upstream Pnuc_1834, conserved hypothetical protein		
m4C	1918023	-		upstream Pnuc_1834, conserved hypothetical protein		
m6A	1918050	+	GAYANNNNNNNRTAG	Pnuc_1838, hypothetical protein		
m6A	1918060	-	CTAYNNNNNNNTRTC	upstream Pnuc_1834, conserved hypothetical protein		
m4C	1919243	-		upstream Pnuc_1839, phage integrase family protein		
m6A	1921973	+	GAYANNNNNNNRTAG	upstream Pnuc_1847, secreted protein		
m6A	1921983	-	CTAYNNNNNNNTRTC	upstream Pnuc_1843, hypothetical protein		
m6A	1922021	-		upstream Pnuc_1843, hypothetical protein		
m4C	1924413	+		upstream Pnuc_1847, secreted protein		
m6A	1926071	-		Pnuc_1849, major facilitator superfamily MFS_1		
m4C	1928493	-		upstream Pnuc_1850, protein of unknown function UPF0126		
m6A	1930518	-		Pnuc_1856, OsmC family protein		
m4C	1931091	-		Pnuc_1857, Rhodanese domain protein		
m6A	1931420	-		upstream Pnuc_1857, Rhodanese domain protein		
m6A	1931430	-		upstream Pnuc_1857, Rhodanese domain protein		
m6A	1932597	+		Pnuc_1859, tRNA-i(6)A37 thiotransferase enzyme MiaB		
m6A	1934625	-		upstream Pnuc_1857, Rhodanese domain protein		
m6A	1935097	+	GAYANNNNNNNRTAG	Pnuc_1861, CBS domain containing protein		
m6A	1935107	-	CTAYNNNNNNNTRTC	upstream Pnuc_1857, Rhodanese domain protein		
m4C	1936507	+		Pnuc_1862, apolipoprotein N-acyltransferase		
m4C	1938110	+		Pnuc_1864, glycyl-tRNA synthetase beta chain		
m6A	1938699	-		upstream Pnuc_1857, Rhodanese domain protein		
m4C	1938967	-		upstream Pnuc_1857, Rhodanese domain protein		
m6A	1940032	+	CTAYNNNNNNNTRTC	Pnuc_1865,D-alpha,beta-D-heptose 1,7-bisphosphate phosphatase		
m6A	1940042	-	GAYANNNNNNNRTAG	upstream Pnuc_1857, Rhodanese domain protein		
m4C	1943052	-		Pnuc_1869, PpiC-type peptidyl-prolyl cis-trans isomerase		
m4C	1943876	+		upstream Pnuc_1871, aminoglycoside phosphotransferase		
m6A	1944178	+		upstream Pnuc_1871, aminoglycoside phosphotransferase		
m6A	1944517	+		upstream Pnuc_1871, aminoglycoside phosphotransferase		
m4C	1944681	+		upstream Pnuc_1871, aminoglycoside phosphotransferase		
m4C	1948306	-		upstream Pnuc_1870, Organic solvent tolerance protein		
m4C	1949187	-		upstream Pnuc_1870, Organic solvent tolerance protein		
m6A	1949603	-		upstream Pnuc_1870, Organic solvent tolerance protein		
m4C	1950025	+		Pnuc_1873, aminopeptidase P, Metallo peptidase, MEROPS family M24B		
m4C	1955548	-		upstream Pnuc_1870, Organic solvent tolerance protein		
m4C	1956019	-		upstream Pnuc_1870, Organic solvent tolerance protein		
m6A	1956076	+		Pnuc_1880, Holliday junction DNA helicase RuvB		
m6A	1961700	+		upstream Pnuc_1887, OsmC family protein		
m4C	1962595	-		upstream Pnuc_R0042, RNase P		
m6A	1962717	+		upstream Pnuc_1900, L-threonine ammonia-lyase		
m6A	1964679	+		upstream Pnuc_1900, L-threonine ammonia-lyase		

m6A	1968003	+	CTAYNNNNNNNTRTC	upstream Pnuc_1900, L-threonine ammonia-lyase		
m6A	1968013	-	GAYANNNNNNNRTAG	Pnuc_1892, aspartyl-tRNA synthetase		
m6A	1968185	+	GAYANNNNNNNRTAG	upstream Pnuc_1900, L-threonine ammonia-lyase		
m6A	1968195	-	CTAYNNNNNNNTRTC	Pnuc_1892, aspartyl-tRNA synthetase		
m6A	1968822	+		upstream Pnuc_1900, L-threonine ammonia-lyase		
m4C	1970066	+		upstream Pnuc_1900, L-threonine ammonia-lyase		
m4C	1972116	+		upstream Pnuc_1900, L-threonine ammonia-lyase		
m4C	1973150	-		upstream Pnuc_1896, import inner membrane translocase, subunit Tim44		
m4C	1973296	+		upstream Pnuc_1900, L-threonine ammonia-lyase		
m6A	1974979	+	GAYANNNNNNNRTAG	upstream Pnuc_1900, L-threonine ammonia-lyase		
m6A	1974989	-	CTAYNNNNNNNTRTC	Pnuc_1899, FAD linked oxidase domain protein		
m4C	1976243	+		upstream Pnuc_1900, L-threonine ammonia-lyase		
m6A	1978391	+		Pnuc_1900, L-threonine ammonia-lyase		
m4C	1980452	-		upstream Pnuc_1899, FAD linked oxidase domain protein		
m4C	1980584	+		Pnuc_1901, 5-nucleotidase		
m4C	1981472	+		Pnuc_1902, GTP cyclohydrolase I		
m4C	1983001	-		Pnuc_1904, major facilitator superfamily MFS_1		
m4C	1984330	+		Pnuc_1905, Excinuclease ABC subunit A		
m4C	1984737	-		upstream Pnuc_1904, major facilitator superfamily MFS_1		
m4C	1986308	-		upstream Pnuc_1904, major facilitator superfamily MFS_2		
m4C	1986900	+		upstream Pnuc_1907, KpsF/GutQ family protein		
m4C	1987093	-		Pnuc_1906, Kef-type potassium/proton antiporter, CPA2 family		
m4C	1987298	+		upstream Pnuc_1907, KpsF/GutQ family protein		
m6A	1992074	+		upstream Pnuc_1912, SSU ribosomal protein S30P / sigma 54 modulation protein		
m6A	1995448	-		Pnuc_1916, A/G-specific adenine glycosylase		
m6A	1995828	-		Pnuc_1916, A/G-specific adenine glycosylase		
m4C	1999392	+		Pnuc_1920, ribose-phosphate pyrophosphokinase		
m6A	1999753	+	GAYANNNNNNNRTAG	Pnuc_1920, ribose-phosphate pyrophosphokinase		
m6A	1999763	-	CTAYNNNNNNNTRTC	upstream Pnuc_1917, DNA-(apurinic or apyrimidinic site) lyase		
m4C	1999854	-		upstream Pnuc_1917, DNA-(apurinic or apyrimidinic site) lyase		
m4C	2000666	+		Pnuc_1921, LSU ribosomal protein L25P		
m4C	2002827	-		Pnuc_1925, putative methyltransferase		
m4C	2003070	+		upstream Pnuc_1928, signal recognition particle-docking protein FtsY		
m6A	2004271	+		upstream Pnuc_1928, signal recognition particle-docking protein FtsY		
m6A	2004383	+		upstream Pnuc_1928, signal recognition particle-docking protein FtsY		
m4C	2004652	+		upstream Pnuc_1928, signal recognition particle-docking protein FtsY		
m6A	2005336	-		Pnuc_1927, peptidase M16 domain protein		
m4C	2007198	+		Pnuc_1929, RNA polymerase, sigma 32 subunit, RpoH		
m6A	2008414	+	GAYANNNNNNNRTAG	upstream Pnuc_1935, conserved hypothetical protein		
m6A	2008424	-	CTAYNNNNNNNTRTC	Pnuc_1931, protoheme IX farnesyltransferase		
m4C	2011071	-		Pnuc_1934, SURF1 family protein		
m4C	2013906	+		upstream Pnuc_1942, phosphoribosyltransferase		
m6A	2014371	+	CTAYNNNNNNNTRTC	upstream Pnuc_1942, phosphoribosyltransferase		
m6A	2014381	-	GAYANNNNNNNRTAG	Pnuc_1939, Cytochrome-c oxidase		

m6A	2014641	+	GAYANNNNNNNRTAG	upstream Pnuc_1942, phosphoribosyltransferase		
m6A	2014651	-	CTAYNNNNNNNTRTC	Pnuc_1939, Cytochrome-c oxidase		
m6A	2016148	-		Pnuc_1940, cytochrome c oxidase, subunit II		
m4C	2016872	-		upstream Pnuc_1940, cytochrome c oxidase, subunit II		
m4C	2017520	+		upstream Pnuc_1942, phosphoribosyltransferase		
m6A	2020628	+		upstream Pnuc_1948, phosphoglycerate mutase		
m4C	2022461	+		Pnuc_1949, carboxyl-terminal protease		
m4C	2025658	+		upstream Pnuc_1959, protein of unknown function DUF526		
m4C	2027802	+		upstream Pnuc_1959, protein of unknown function DUF526		
m4C	2029132	-		Pnuc_1956, ammonium transporter		
m6A	2030432	-		Pnuc_1956, ammonium transporter		
m6A	2033708	-		Pnuc_1961, lipoic acid synthetase		
m4C	2034782	-		Pnuc_1963, protein of unknown function DUF493		
m6A	2035852	+		upstream Pnuc_1967, biotin--acetyl-CoA-carboxylase ligase		
m4C	2037041	-		upstream Pnuc_1966, VanZ family protein		
m6A	2040053	-		upstream Pnuc_1969, rfaE bifunctional protein		
m4C	2042759	+		Pnuc_1973, Cyclopropane-fatty-acyl-phospholipid synthase		
m4C	2045187	+		upstream Pnuc_1978, conserved hypothetical protein		
m4C	2052349	+		upstream Pnuc_1983, protein of unknown function DUF1289		
m4C	2053009	-		Pnuc_1984, Alcohol dehydrogenase, zinc-binding domain protein		
m6A	2054201	+	CTAYNNNNNNNTRTC	Pnuc_1985, hypothetical protein		
m6A	2054211	-	GAYANNNNNNNRTAG	upstream Pnuc_1984, Alcohol dehydrogenase, zinc-binding domain protein		
m4C	2054550	-		upstream Pnuc_1984, Alcohol dehydrogenase, zinc-binding domain protein		
m6A	2058112	+		upstream Pnuc_1996, protein of unknown function DUF185		
m4C	2060692	-		Pnuc_1990, RND efflux system, outer membrane lipoprotein, NodT family		
m4C	2061208	+		upstream Pnuc_1996, protein of unknown function DUF185		
m4C	2061233	+		upstream Pnuc_1996, protein of unknown function DUF185		
m6A	2062540	-		Pnuc_1991, glutamine-fructose-6-phosphate transaminase		
m4C	2063429	-		Pnuc_1991, glutamine-fructose-6-phosphate transaminase		
m4C	2063555	-		Pnuc_1992, UDP-N-acetylglucosamine pyrophosphorylase / glucosamine-1-phosphate N-acetyltransferase		
m6A	2063777	+	CTAYNNNNNNNTRTC	upstream Pnuc_1996, protein of unknown function DUF185		
m6A	2063787	-	GAYANNNNNNNRTAG	Pnuc_1992, UDP-N-acetylglucosamine pyrophosphorylase / glucosamine-1-phosphate N-acetyltransferase		
m6A	2066312	+	GAYANNNNNNNRTAG	upstream Pnuc_1996, protein of unknown function DUF185		
m6A	2066322	-	CTAYNNNNNNNTRTC	Pnuc_1994, dihydroneopterin aldolase		
m4C	2068726	-		Pnuc_1997, Polynucleotide adenylyltransferase region		
m6A	2071863	+		upstream Pnuc_2000, 5-formyltetrahydrofolate cyclo-ligase		
m4C	2072459	-		upstream Pnuc_1999, Lytic transglycosylase, catalytic		
m4C	2072686	+		Pnuc_2000, 5-formyltetrahydrofolate cyclo-ligase		
m4C	2072991	-		upstream Pnuc_1999, Lytic transglycosylase, catalytic		
m4C	2073910	+		upstream Pnuc_2004, lipid A biosynthesis acyltransferase		
m6A	2073919	+		upstream Pnuc_2004, lipid A biosynthesis acyltransferase		
m4C	2075118	-		Pnuc_2002, adenosylhomocysteinase		
m6A	2078652	-		upstream Pnuc_2003, methionine adenosyltransferase		
m4C	2081507	+		upstream Pnuc_2021, rod shape-determining protein MreB		

m4C	2083039	+		upstream Pnuc_2021, rod shape-determining protein MreB		
m6A	2083866	+		upstream Pnuc_2021, rod shape-determining protein MreB		
m4C	2085644	-		Pnuc_2013, N-acetylglutamate kinase		
m4C	2085776	-		Pnuc_2013, N-acetylglutamate kinase		
m6A	2085972	+	GAYANNNNNNNRTAG	upstream Pnuc_2021, rod shape-determining protein MreB		
m6A	2085982	-	CTAYNNNNNNNTRTC	Pnuc_2014, transcriptional regulator, TraR/DksA family		
m4C	2086807	+		upstream Pnuc_2021, rod shape-determining protein MreB		
m4C	2087642	-		upstream Pnuc_2015, cobalamin synthesis protein, P47K		
m4C	2088399	-		Pnuc_2016, tyrosine recombinase XerC subunit		
m4C	2089108	-		Pnuc_2017, protein of unknown function DUF484		
m6A	2091308	+		upstream Pnuc_2021, rod shape-determining protein MreB		
m6A	2091399	-		Pnuc_2019, aspartyl/glutamyl-tRNA(Asn/Gln) amidotransferase subunit A		
m4C	2092208	-		Pnuc_2019, aspartyl/glutamyl-tRNA(Asn/Gln) amidotransferase subunit A		
m4C	2092865	-		upstream Pnuc_2020, aspartyl/glutamyl-tRNA(Asn/Gln) amidotransferase subunit C		
m4C	2093583	-		upstream Pnuc_2020, aspartyl/glutamyl-tRNA(Asn/Gln) amidotransferase subunit C		
m4C	2094788	+		Pnuc_2023, rod shape-determining protein MreD		
m6A	2098763	-		Pnuc_2027, conserved hypothetical protein		
m4C	2098991	-		Pnuc_2027, conserved hypothetical protein		
m6A	2102026	+	CTAYNNNNNNNTRTC	Pnuc_2031, lipid A biosynthesis acyltransferase		
m6A	2102036	-	GAYANNNNNNNRTAG	upstream Pnuc_2029, protein of unknown function DUF167		
m4C	2104632	-		Pnuc_2034, sodium:dicarboxylate symporter		
m4C	2104656	+		upstream Pnuc_2035, ribosomal large subunit pseudouridine synthase F		
m6A	2106577	-		upstream Pnuc_2034, sodium:dicarboxylate symporter		
m6A	2107824	+		Pnuc_2036, bacterial peptide chain release factor 3(bRF-3)		
m6A	2115796	-		upstream Pnuc_2043, alpha/beta hydrolase fold protein		
m6A	2118474	+	GAYANNNNNNNRTAG	upstream Pnuc_2056, protein of unknown function DUF897		
m6A	2118484	-	CTAYNNNNNNNTRTC	Pnuc_2049, hypothetical protein		
m6A	2124960	-		upstream Pnuc_2055, transcriptional regulator, LysR family		
m4C	2125512	+		Pnuc_2057, conserved hypothetical protein		
m4C	2126829	+		upstream Pnuc_2062, hypothetical protein		
m6A	2127286	-		Pnuc_2060, Patatin		
m4C	2128347	+		upstream Pnuc_2062, hypothetical protein		
m6A	2129286	-		upstream Pnuc_2060, Patatin		
m6A	2129770	-		upstream Pnuc_2060, Patatin		
m6A	2132918	+		upstream Pnuc_2065, hypothetical protein		
m4C	2132997	+		upstream Pnuc_2065, hypothetical protein		
m4C	2133900	+		Pnuc_2065, hypothetical protein		
m6A	2133944	+	GAYANNNNNNNRTAG	Pnuc_2065, hypothetical protein		
m6A	2133954	-	CTAYNNNNNNNTRTC	upstream Pnuc_2064, hypothetical protein		
m6A	2136475	+		upstream Pnuc_2071, conserved hypothetical protein		
m4C	2137501	+		upstream Pnuc_2071, conserved hypothetical protein		
m4C	2138678	-		upstream Pnuc_2070, conserved hypothetical protein		
m4C	2139332	-		Pnuc_2072, hypothetical protein		
m6A	2142268	-		Pnuc_2073, protein of unknown function DUF181		

m6A	2143348	-		Pnuc_2075, multi-sensor signal transduction histidine kinase	
m6A	2145506	-		Pnuc_2076, putative periplasmic ligand-binding sensor protein	
m4C	2145808	+		upstream Pnuc_2080, DNA protecting protein DprA	
m4C	2146159	+		upstream Pnuc_2080, DNA protecting protein DprA	
m6A	2146256	+	CTAYNNNNNNNNTRTC	upstream Pnuc_2080, DNA protecting protein DprA	
m6A	2146266	-	GAYANNNNNNNNRTAG	Pnuc_2077, sun protein	
m4C	2147414	+		upstream Pnuc_2080, DNA protecting protein DprA	
m4C	2149995	+		Pnuc_2081, DNA topoisomerase III	
m4C	2150376	+		Pnuc_2081, DNA topoisomerase III	
m4C	2151878	+		Pnuc_2081, DNA topoisomerase III	
m4C	2152508	+		upstream Pnuc_0001, chromosomal replication initiator protein DnaA	
m4C	2154297	+		upstream Pnuc_0001, chromosomal replication initiator protein DnaA	
m4C	2154427	+		upstream Pnuc_0001, chromosomal replication initiator protein DnaA	
m4C	2154602	+		upstream Pnuc_0001, chromosomal replication initiator protein DnaA	

**26°C + UV**					
**Methylation**	**Location**	**Strand**	**Motif**	**Gene / Intergenic region**	**Count**
m4C	2036	+		Pnuc_0002, DNA polymerase III, beta subunit	457(+)
m6A	10210	+		Pnuc_0010, amino acid/amide ABC transporter membrane protein 2, HAAT family	416(-)
m4C	10516	+		Pnuc_0010, amino acid/amide ABC transporter membrane protein 2, HAAT family	**Total** = 873 (m6A + m4C)
m4C	19024	+		upstream Pnuc_0019, putative ATP synthase protein I AtpI	
m6A	22187	-		upstream Pnuc_0013, acyl-CoA dehydrogenase domain protein	253(+)
m4C	23579	+		Pnuc_0025, ATP synthase F1 subcomplex gamma subunit	251(-)
m6A	24264	+		Pnuc_0025, ATP synthase F1 subcomplex gamma subunit	**Total** = 504 (m6A)
m4C	29191	-		upstream Pnuc_0013, acyl-CoA dehydrogenase domain protein	
m4C	30971	+		upstream Pnuc_0033, cytochrome c, class I	204(+)
m4C	31989	+		upstream Pnuc_0033, cytochrome c, class I	165(-)
m4C	32567	+		Pnuc_0033, cytochrome c, class I	Total = 369 (m4C)
m4C	35794	+		upstream Pnuc_R0002, 16S ribosomal RNA	
m4C	37423	-		upstream Pnuc_0038, GCN5-related N-acetyltransferase	Methylated genes = 336 (16.09%)
m6A	37807	+	CTAYNNNNNNNNTRTC	Pnuc_R0002, 16S ribosomal RNA	
m6A	37817	-	GAYANNNNNNNNRTAG	upstream Pnuc_0038, GCN5-related N-acetyltransferase	
m4C	37977	-		upstream Pnuc_0038, GCN5-related N-acetyltransferase	
m4C	43404	-		upstream Pnuc_0038, GCN5-related N-acetyltransferase	
m4C	46285	+		Pnuc_0044, LSU ribosomal protein L10P	
m4C	47940	-		upstream Pnuc_0038, GCN5-related N-acetyltransferase	
m4C	48187	+		Pnuc_0046, DNA-directed RNA polymerase subunit beta	
m4C	54576	+		Pnuc_0047, DNA-directed RNA polymerase subunit beta	
m4C	57483	-		upstream Pnuc_0038, GCN5-related N-acetyltransferase	
m6A	58559	-		upstream Pnuc_0038, GCN5-related N-acetyltransferase	
m4C	61205	+		Pnuc_0053, LSU ribosomal protein L3P	
m4C	61904	+		Pnuc_0054, LSU ribosomal protein L4P	

m6A	69850	-		upstream Pnuc_0038, GCN5-related N-acetyltransferase
m4C	70523	-		upstream Pnuc_0038, GCN5-related N-acetyltransferase
m4C	70690	+		Pnuc_0073, protein translocase subunit secY/sec61 alpha
m4C	72955	-		upstream Pnuc_0038, GCN5-related N-acetyltransferase
m4C	74844	+		upstream Pnuc_0080, CutA1 divalent ion tolerance protein
m4C	75359	-		upstream Pnuc_0038, GCN5-related N-acetyltransferase
m6A	77105	-		upstream Pnuc_0038, GCN5-related N-acetyltransferase
m4C	78311	+		upstream Pnuc_0085, Cytochrome c553-like protein
m4C	80546	-		Pnuc_0086, diaminopimelate decarboxylase
m6A	83003	+	GAYANNNNNNNRTAG	upstream Pnuc_0092, penicillin-binding protein, 1A family
m6A	83013	-	CTAYNNNNNNNTRTC	Pnuc_0088, 3-dehydroquinate synthase
m6A	84386	+	CTAYNNNNNNNTRTC	upstream Pnuc_0092, penicillin-binding protein, 1A family
m6A	84396	-	GAYANNNNNNNRTAG	Pnuc_0090, type II and III secretion system protein
m6A	85182	-		Pnuc_0091, hypothetical protein
m6A	85246	-		Pnuc_0091, hypothetical protein
m4C	91931	+		Pnuc_0094, glutamate synthase (NADH) large subunit
m4C	101398	+		Pnuc_0105, UDP-N-acetylglucosamine 1-carboxyvinyltransferase
m6A	101539	+		Pnuc_0105, UDP-N-acetylglucosamine 1-carboxyvinyltransferase
m6A	102779	+	GAYANNNNNNNRTAG	Pnuc_0107, histidinol dehydrogenase
m6A	102789	-	CTAYNNNNNNNTRTC	upstream Pnuc_0091, hypothetical protein
m6A	103179	+	CTAYNNNNNNNTRTC	Pnuc_0107, histidinol dehydrogenase
m6A	103189	-	GAYANNNNNNNRTAG	upstream Pnuc_0091, hypothetical protein
m4C	117662	-		Pnuc_0122, Tetratricopeptide TPR_2 repeat protein
m6A	122705	+	CTAYNNNNNNNTRTC	Pnuc_0127, cytochrome c1
m6A	122715	-	GAYANNNNNNNRTAG	upstream Pnuc_0124, large conductance mechanosensitive channel protein
m6A	124671	+		Pnuc_0130, TPR domain protein
m4C	126750	-		upstream Pnuc_0131, General substrate transporter
m6A	130121	+	GAYANNNNNNNRTAG	upstream Pnuc_0142, GTP-binding protein YchF
m6A	130131	-	CTAYNNNNNNNTRTC	Pnuc_0136, bacterial peptide chain release factor 1(bRF-1)
m4C	130984	+		upstream Pnuc_0142, GTP-binding protein YchF
m4C	136421	-		upstream Pnuc_0141, Ubiquinone biosynthesis hydroxylase, UbiH/UbiF/VisC/COQ6 family
m6A	137802	-		Pnuc_0141, Ubiquinone biosynthesis hydroxylase, UbiH/UbiF/VisC/COQ6 family
m6A	139555	-		Pnuc_0145, indole-3-glycerol phosphate synthase
m4C	139957	+		upstream Pnuc_0150, ApaG domain protein
m6A	141313	-		Pnuc_0147, anthranilate synthase, component II
m4C	142593	+		upstream Pnuc_0150, ApaG domain protein
m4C	143233	-		Pnuc_0149, ribulose-5-phosphate 3-epimerase
m4C	143384	+		upstream Pnuc_0150, ApaG domain protein
m4C	144036	+		upstream Pnuc_0151, MltA domain protein
m4C	144075	-		upstream Pnuc_0149, ribulose-5-phosphate 3-epimerase
m4C	145779	+		upstream Pnuc_0156, biotin synthase
m6A	149067	+	CTAYNNNNNNNTRTC	upstream Pnuc_0156, biotin synthase
m6A	149077	-	GAYANNNNNNNRTAG	Pnuc_0155, aminotransferase
m4C	151293	+		Pnuc_0157, putative ubiquinone biosynthesis protein

m6A	157993	+	GAYANNNNNNNNRTAG	Pnuc_0163, UDP-N-acetylmuramoylalanyl-D-glutamyl-2,6-diaminopimelate--D-alanyl-D-alanyl ligase		
m6A	158003	-	CTAYNNNNNNNNTRTC	upstream Pnuc_0155, aminotransferase		
m4C	165880	-		upstream Pnuc_0155, aminotransferase		
m4C	167854	-		upstream Pnuc_0155, aminotransferase		
m4C	171063	-		Pnuc_0175, hypothetical protein		
m4C	178192	-		upstream Pnuc_0175, hypothetical protein		
m6A	182434	-		Pnuc_0185, type II secretion system protein		
m4C	186994	-		upstream Pnuc_0189, farnesyl-diphosphate farnesyltransferase		
m4C	190804	-		Pnuc_0193, Farnesyltranstransferase		
m4C	191543	+		Pnuc_0195, LSU ribosomal protein L27P		
m6A	199904	+		upstream Pnuc_0204, ribonucleoside-diphosphate reductase, alpha subunit		
m6A	199926	+		upstream Pnuc_0204, ribonucleoside-diphosphate reductase, alpha subunit		
m6A	200762	+	CTAYNNNNNNNNTRTC	Pnuc_0204, ribonucleoside-diphosphate reductase, alpha subunit		
m6A	200772	-	GAYANNNNNNNNRTAG	upstream Pnuc_0201, signal recognition particle subunit FFH/SRP54 (srp54)		
m4C	200904	+		Pnuc_0204, ribonucleoside-diphosphate reductase, alpha subunit		
m4C	205747	+		upstream Pnuc_0213, UDP-N-acetylmuramate		
m4C	206369	+		upstream Pnuc_0213, UDP-N-acetylmuramate		
m4C	206994	+		upstream Pnuc_0213, UDP-N-acetylmuramate		
m4C	208221	-		Pnuc_0209, acetyl-CoA carboxylase, biotin carboxylase		
m4C	208743	-		Pnuc_0209, acetyl-CoA carboxylase, biotin carboxylase		
m4C	209491	-		Pnuc_0210, biotin carboxyl carrier protein		
m6A	209668	+	GAYANNNNNNNNRTAG	upstream Pnuc_0213, UDP-N-acetylmuramate		
m6A	209678	-	CTAYNNNNNNNNTRTC	Pnuc_0211, 3-dehydroquinate dehydratase		
m4C	211180	+		Pnuc_0213, UDP-N-acetylmuramate		
m4C	214042	+		Pnuc_0215, ribonuclease II		
m4C	218189	-		Pnuc_0219, orotidine 5'-phosphate decarboxylase		
m4C	219189	+		Pnuc_0220, magnesium and cobalt transport protein CorA		
m4C	221772	-		upstream Pnuc_0223, thiamine-phosphate kinase		
m6A	227673	+	GAYANNNNNNNNRTAG	Pnuc_0228, glyceraldehyde-3-phosphate dehydrogenase		
m6A	227683	-	CTAYNNNNNNNNTRTC	upstream Pnuc_0226, protein of unknown function DUF558		
m6A	228733	-		Pnuc_0229, ferric uptake regulator, Fur family		
m4C	229043	-		Pnuc_0229, ferric uptake regulator, Fur family		
m4C	229114	+		upstream Pnuc_0230, SmpA/OmlA domain protein		
m4C	229124	+		upstream Pnuc_0230, SmpA/OmlA domain protein		
m6A	229942	-		upstream Pnuc_0229, ferric uptake regulator, Fur family		
m4C	237850	+		Pnuc_0237, putative RNA methylase		
m4C	245244	+		Pnuc_0246, aspartate carbamoyltransferase		
m4C	247712	-		upstream Pnuc_0241, deoxyribodipyrimidine photo-lyase type I		
m6A	248429	+		Pnuc_0249, glycosyl transferase, family 25		
m6A	248439	-	GAYANNNNNNNNRTAG	upstream Pnuc_0241, deoxyribodipyrimidine photo-lyase type I		
m4C	249513	-		Pnuc_0250, hypothetical protein		
m6A	249670	+		upstream Pnuc_0252, hypothetical protein		
m4C	250708	-		Pnuc_0251, hypothetical protein		
m6A	250899	+	CTAYNNNNNNNNTRTC	Pnuc_0252, hypothetical protein		

m6A	250909	-	GAYANNNNNNNRTAG	upstream Pnuc_0251, hypothetical protein		
m6A	251666	-	CTAYNNNNNNNTRTC	upstream Pnuc_0251, hypothetical protein		
m4C	257150	+		upstream Pnuc_0259, Undecaprenyl-diphosphatase		
m6A	269370	+	GAYANNNNNNNRTAG	upstream Pnuc_0279, serine hydroxymethyltransferase		
m6A	269380	-	CTAYNNNNNNNTRTC	Pnuc_0272 (start codon), preQ(0) biosynthesis protein QueC		
m6A	271013	+		upstream Pnuc_0279, serine hydroxymethyltransferase		
m6A	271023	-	CTAYNNNNNNNTRTC	Pnuc_0275, TolB, N-terminal domain protein		
m6A	277009	+	CTAYNNNNNNNTRTC	upstream Pnuc_0287, Exodeoxyribonuclease VII large subunit		
m6A	277019	-	GAYANNNNNNNRTAG	Pnuc_0282, 3-deoxy-D-manno-octulosonate cytidylyltransferase		
m6A	279077	-		Pnuc_0285, Biopolymer transport protein ExbD/TolR		
m6A	280100	+		upstream Pnuc_0287, Exodeoxyribonuclease VII large subunit		
m6A	283189	+	GAYANNNNNNNRTAG	upstream Pnuc_0290, protein of unknown function DUF520		
m6A	283199	-	CTAYNNNNNNNTRTC	Pnuc_0289, UDP-N-acetylmuramate dehydrogenase		
m4C	283631	+		upstream Pnuc_0290, protein of unknown function DUF520		
m4C	304396	-		upstream Pnuc_0310, sugar transferase		
m6A	305983	-		upstream Pnuc_0310, sugar transferase		
m4C	309403	+		Pnuc_0317, glycosyl transferase, family 2		
m6A	310366	+	CTAYNNNNNNNTRTC	Pnuc_0318, ABC-2 type transporter		
m6A	310376	-	GAYANNNNNNNRTAG	upstream Pnuc_0316, glycosyl transferase, family 4		
m6A	312933	+	GAYANNNNNNNRTAG	Pnuc_0320, Methyltransferase type 11		
m6A	312943	-	CTAYNNNNNNNTRTC	upstream Pnuc_0316, glycosyl transferase, family 4		
m6A	313185	-	CTAYNNNNNNNTRTC	upstream Pnuc_0316, glycosyl transferase, family 4		
m6A	318999	+		upstream Pnuc_0324, NAD-dependent epimerase/dehydratase		
m6A	319009	-	GAYANNNNNNNRTAG	upstream Pnuc_0316, glycosyl transferase, family 4		
m4C	320163	+		Pnuc_0325, amine oxidase		
m6A	320925	+	GAYANNNNNNNRTAG	Pnuc_0325, amine oxidase		
m6A	320935	-	CTAYNNNNNNNTRTC	upstream Pnuc_0316, glycosyl transferase, family 4		
m6A	321154	+	CTAYNNNNNNNTRTC	Pnuc_0325, amine oxidase		
m6A	321164	-	GAYANNNNNNNRTAG	upstream Pnuc_0316, glycosyl transferase, family 4		
m4C	322370	-		Pnuc_0327, short-chain dehydrogenase/reductase SDR		
m6A	324292	+	GAYANNNNNNNRTAG	Pnuc_0329, UbiA prenyltransferase		
m6A	324302	-	CTAYNNNNNNNTRTC	upstream Pnuc_0328, FAD linked oxidase domain protein		
m6A	335858	+	CTAYNNNNNNNTRTC	Pnuc_0338, hypothetical protein		
m6A	335868	-	GAYANNNNNNNRTAG	upstream Pnuc_0336, hypothetical protein		
m6A	345459	+	GAYANNNNNNNRTAG	upstream Pnuc_0351, acyltransferase 3		
m6A	345469	-	CTAYNNNNNNNTRTC	upstream Pnuc_0349, hypothetical protein		
m6A	345472	-		upstream Pnuc_0349, hypothetical protein		
m6A	347772	+	GAYANNNNNNNRTAG	Pnuc_0353, Choline dehydrogenase		
m6A	347782	-	CTAYNNNNNNNTRTC	upstream Pnuc_0352, protein of unknown function DUF615		
m4C	348956	+		Pnuc_0354, protein of unknown function DUF6, transmembrane		
m6A	349791	-		upstream Pnuc_0352, protein of unknown function DUF615		
m4C	353886	+		Pnuc_0357, amino acid/amide ABC transporter ATP-binding protein 1, HAAT family		
m4C	355021	-		Pnuc_0359, hypothetical protein		
m4C	355781	+		upstream Pnuc_0362, conserved hypothetical protein		

m4C	367208	+		Pnuc_0371, transcriptional regulator, TetR family		
m4C	375033	+		upstream Pnuc_0384, D-isomer specific 2-hydroxyacid dehydrogenase, NAD-binding protein		
m4C	378715	-		Pnuc_0387, molybdenum cofactor guanylyltransferase		
m4C	378809	-		Pnuc_0387, molybdenum cofactor guanylyltransferase		
m4C	380268	+		upstream Pnuc_0390, ribosomal large subunit pseudouridine synthase C		
m4C	382926	+		upstream Pnuc_0390, ribosomal large subunit pseudouridine synthase C		
m4C	387629	+		Pnuc_0395, protein of unknown function DUF177		
m4C	387941	+		Pnuc_0395, protein of unknown function DUF177		
m4C	395248	-		upstream Pnuc_0394, maf protein		
m6A	397041	+	CTAYNNNNNNNTRTC	Pnuc_0404, GTP-binding protein LepA		
m6A	401086	+	CTAYNNNNNNNTRTC	Pnuc_0409, pyridoxine 5'-phosphate synthase		
m6A	401096	-	GAYANNNNNNNRTAG	upstream Pnuc_0394, maf protein		
m6A	401232	+	GAYANNNNNNNRTAG	Pnuc_0410, holo-acyl-carrier-protein synthase		
m6A	401242	-	CTAYNNNNNNNTRTC	upstream Pnuc_0394, maf protein		
m6A	403790	-		Pnuc_0413, translation elongation factor P (EF-P)		
m6A	403974	-		Pnuc_0413, translation elongation factor P (EF-P)		
m4C	404185	+		upstream Pnuc_0415, Excinuclease ABC subunit C		
m4C	404201	-		upstream Pnuc_0413, translation elongation factor P (EF-P)		
m4C	405407	+		Pnuc_0415, Excinuclease ABC subunit C		
m4C	406883	-		upstream Pnuc_0414, conserved hypothetical protein		
m4C	412334	-		upstream Pnuc_0418, SOS-response transcriptional repressor, LexA		
m4C	413567	-		upstream Pnuc_0418, SOS-response transcriptional repressor, LexA		
m6A	421455	+	GAYANNNNNNNRTAG	upstream Pnuc_0437, aminotransferase		
m6A	421465	-	CTAYNNNNNNNTRTC	Pnuc_0432, formyl transferase domain protein		
m4C	421490	-		Pnuc_0432, formyl transferase domain protein		
m6A	422836	+	CTAYNNNNNNNTRTC	upstream Pnuc_0437, aminotransferase		
m6A	422846	-	GAYANNNNNNNRTAG	Pnuc_0434, DegT/DnrJ/EryC1/StrS aminotransferase		
m4C	424370	+		upstream Pnuc_0437, aminotransferase		
m4C	427493	-		upstream Pnuc_0436, protein of unknown function DUF498		
m6A	439040	+	GAYANNNNNNNRTAG	Pnuc_0447, ATPase AAA-2 domain protein		
m6A	439050	-	CTAYNNNNNNNTRTC	upstream Pnuc_0446, Excinuclease ABC, C subunit domain protein		
m6A	439614	-		upstream Pnuc_0446, Excinuclease ABC, C subunit domain protein		
m6A	440012	+	GAYANNNNNNNRTAG	Pnuc_0447, ATPase AAA-2 domain protein		
m6A	442569	+	GAYANNNNNNNRTAG	Pnuc_0450, major facilitator superfamily MFS_1		
m6A	442579	-	CTAYNNNNNNNTRTC	upstream Pnuc_0449, Uncharacterized protein UPF0065		
m6A	445942	+	CTAYNNNNNNNTRTC	upstream Pnuc_0453, cytochrome c oxidase, cbb3-type, subunit I		
m6A	445952	-	GAYANNNNNNNRTAG	upstream Pnuc_0449, Uncharacterized protein UPF0065		
m4C	451434	+		upstream Pnuc_0461, putative transmembrane protein		
m4C	452040	+		Pnuc_0461, putative transmembrane protein		
m6A	453512	+	CTAYNNNNNNNTRTC	Pnuc_0463, UspA domain protein		
m6A	453522	-	GAYANNNNNNNRTAG	upstream Pnuc_0462, conserved hypothetical protein		
m4C	458223	+		upstream Pnuc_0471, 3-hydroxyacyl-CoA dehydrogenase		
m6A	461835	+	GAYANNNNNNNRTAG	upstream Pnuc_0471, 3-hydroxyacyl-CoA dehydrogenase		
m4C	463015	-		upstream Pnuc_0470, D-isomer specific 2-hydroxyacid dehydrogenase, NAD-binding protein		

m4C	471398	+		upstream Pnuc_0485, Uncharacterized protein UPF0065		
m4C	471940	+		upstream Pnuc_0485, Uncharacterized protein UPF0065		
m4C	472091	-		Pnuc_0482, MmgE/PrpD family protein		
m6A	474614	+	CTAYNNNNNNNTRTC	upstream Pnuc_0485, Uncharacterized protein UPF0065		
m6A	474624	-	GAYANNNNNNNRTAG	upstream Pnuc_0484, Rhodanese domain protein		
m4C	477378	+		Pnuc_0488, methyltransferase small		
m6A	477475	-		upstream Pnuc_0484, Rhodanese domain protein		
m4C	479258	-		upstream Pnuc_0484, Rhodanese domain protein		
m4C	480232	-		upstream Pnuc_0484, Rhodanese domain protein		
m4C	480774	-		Pnuc_0490, phosphoglycolate phosphatase		
m4C	486646	+		Pnuc_0494, phosphoserine aminotransferase apoenzyme		
m6A	499723	+		Pnuc_0507, helix-hairpin-helix motif protein		
m4C	501868	+		upstream Pnuc_0510, histone deacetylase superfamily		
m6A	511656	+	GAYANNNNNNNRTAG	Pnuc_0520, LSU ribosomal protein L19P		
m6A	511666	-	CTAYNNNNNNNTRTC	upstream Pnuc_0516, protein of unknown function DUF306, Meta and HslJ		
m4C	511707	-		upstream Pnuc_0516, protein of unknown function DUF306, Meta and HslJ		
m6A	513038	-		upstream Pnuc_0516, protein of unknown function DUF306, Meta and HslJ		
m6A	513793	+	CTAYNNNNNNNTRTC	upstream Pnuc_0525, Exonuclease, RNase T and DNA polymerase III		
m6A	513803	-	GAYANNNNNNNRTAG	Pnuc_0523, ribosome small subunit-dependent GTPase A		
m6A	516835	+	GAYANNNNNNNRTAG	upstream Pnuc_0527, microcin-processing peptidase 1, Unknown type peptidase, MEROPS family U62		
m6A	516845	-	CTAYNNNNNNNTRTC	Pnuc_0526, molybdopterin adenylyltransferase		
m4C	528854	+		upstream Pnuc_0541, Glutathione S-transferase, N-terminal domain protein		
m6A	529410	+	GAYANNNNNNNRTAG	upstream Pnuc_0541, Glutathione S-transferase, N-terminal domain protein		
m6A	529420	-	CTAYNNNNNNNTRTC	upstream Pnuc_0538, hypothetical protein		
m4C	532214	+		upstream Pnuc_0541, Glutathione S-transferase, N-terminal domain protein		
m6A	552556	+		upstream Pnuc_0565, hypothetical protein		
m6A	553023	+	CTAYNNNNNNNTRTC	upstream Pnuc_0565, hypothetical protein		
m6A	553033	-	GAYANNNNNNNRTAG	Pnuc_0563, integral membrane sensor hybrid histidine kinase		
m6A	556728	-		Pnuc_0568, conserved hypothetical protein		
m4C	556883	-		Pnuc_0568, conserved hypothetical protein		
m6A	557691	+		upstream Pnuc_0570, cation diffusion facilitator family transporter		
m6A	560162	+	GAYANNNNNNNRTAG	Pnuc_0570, cation diffusion facilitator family transporter		
m6A	560172	-	CTAYNNNNNNNTRTC	upstream Pnuc_0569, GCN5-related N-acetyltransferase		
m4C	562505	+		Pnuc_0573, multiple antibiotic resistance (MarC)-related protein		
m6A	563811	-		upstream Pnuc_0571, beta-lactamase domain protein		
m6A	564182	-		upstream Pnuc_0571, beta-lactamase domain protein		
m4C	567773	+		upstream Pnuc_0580, hypothetical protein		
m4C	574015	-		upstream Pnuc_0582, hypothetical protein		
m6A	575931	+		upstream Pnuc_0594, 2-hydroxy-3-oxopropionate reductase		
m6A	581452	-		Pnuc_0595, N-acetylmuramoyl-L-alanine amidase		
m4C	585405	-		Pnuc_0600, FAD linked oxidase domain protein		
m6A	587229	+		upstream Pnuc_0602, Serine--glyoxylate transaminase		
m6A	587239	-	CTAYNNNNNNNTRTC	Pnuc_0600, FAD linked oxidase domain protein		
m4C	588024	+		upstream Pnuc_0602, Serine--glyoxylate transaminase		

m6A	590041	-		upstream Pnuc_0601, transcriptional regulator, GntR family
m6A	598932	+	GAYANNNNNNNRTAG	upstream Pnuc_0610, lipid A ABC exporter, fused ATPase and inner membrane subunits MsbA
m4C	600957	-		upstream Pnuc_0609, glycosyl transferase, family 2
m4C	601332	-		Pnuc_0611, RNAse G
m6A	610145	+		upstream Pnuc_0623, aminopeptidase A, Metallo peptidase, MEROPS family M17
m6A	618648	+	GAYANNNNNNNRTAG	Pnuc_0628, TRAP dicarboxylate transporter- DctP subunit
m6A	618658	-	CTAYNNNNNNNTRTC	upstream Pnuc_0622, permease YjgP/YjgQ family protein
m6A	621941	+	GAYANNNNNNNRTAG	Pnuc_0631, AMP-dependent synthetase and ligase
m6A	621951	-	CTAYNNNNNNNTRTC	upstream Pnuc_0622, permease YjgP/YjgQ family protein
m4C	624499	+		upstream Pnuc_0636, cyclic nucleotide-binding protein
m6A	630181	+		Pnuc_0638, L-carnitine dehydratase/bile acid-inducible protein F
m4C	630740	+		Pnuc_0639, putative PAS/PAC sensor protein
m6A	632692	+		Pnuc_0641, Methionyl-tRNA formyltransferase
m4C	637747	-		Pnuc_0647, methionyl-tRNA synthetase
m4C	638606	-		Pnuc_0647, methionyl-tRNA synthetase
m6A	639054	+	GAYANNNNNNNRTAG	Pnuc_0648, formate dehydrogenase, subunit FdhD
m6A	639064	-	CTAYNNNNNNNTRTC	upstream Pnuc_0647, methionyl-tRNA synthetase
m6A	639706	+		Pnuc_0648, formate dehydrogenase, subunit FdhD
m6A	641919	-		upstream Pnuc_0647, methionyl-tRNA synthetase
m6A	642290	+	CTAYNNNNNNNTRTC	Pnuc_0651, Lysine decarboxylase
m6A	642300	-	GAYANNNNNNNRTAG	upstream Pnuc_0647, methionyl-tRNA synthetase
m4C	646068	+		upstream Pnuc_0656, porphobilinogen deaminase
m4C	648337	+		Pnuc_0656, porphobilinogen deaminase
m4C	656646	-		upstream Pnuc_0663, Pyridoxamine 5'-phosphate oxidase
m6A	664401	+	CTAYNNNNNNNTRTC	upstream Pnuc_0672, ABC transporter related protein
m6A	664411	-		Pnuc_0670, cyanophycin synthetase
m4C	665984	-		Pnuc_0671, cyanophycin synthetase
m6A	685642	+	CTAYNNNNNNNTRTC	Pnuc_0686, V-type H(+)-translocating pyrophosphatase
m6A	685652	-	GAYANNNNNNNRTAG	upstream Pnuc_0685, Inorganic diphosphatase
m4C	696773	+		Pnuc_0696, amidohydrolase
m4C	702310	-		upstream Pnuc_R0019, SRP RNA; RNA component of signal recognition particle
m6A	706399	+		Pnuc_0705, DNA-directed DNA polymerase
m6A	709971	+		Pnuc_0711, AsmA family protein
m4C	714147	-		upstream Pnuc_0716, YaeQ family protein
m6A	716815	+	CTAYNNNNNNNTRTC	Pnuc_0718, coproporphyrinogen III oxidase, anaerobic
m6A	716825	-	GAYANNNNNNNRTAG	upstream Pnuc_0716, YaeQ family protein
m6A	717198	+	CTAYNNNNNNNTRTC	upstream Pnuc_0719, OmpW family protein
m6A	717208	-	GAYANNNNNNNRTAG	upstream Pnuc_0716, YaeQ family protein
m4C	718382	+		upstream Pnuc_0721, ABC transporter related protein
m4C	723601	-		upstream Pnuc_0724, Altronate dehydratase
m6A	724976	-		upstream Pnuc_0725, Pirin domain protein
m6A	726362	-		upstream Pnuc_0727, phosphate-starvation-inducible E
m4C	727752	+		upstream Pnuc_0734, 2-oxo-acid dehydrogenase E1 subunit, homodimeric type
m6A	730423	+	CTAYNNNNNNNTRTC	upstream Pnuc_0734, 2-oxo-acid dehydrogenase E1 subunit, homodimeric type

m6A	730433	-	GAYANNNNNNNRTAG	Pnuc_0731, methenyltetrahydrofolate cyclohydrolase / 5,10-methylenetetrahydrofolate dehydrogenase (NADP+)		
m4C	730466	-		Pnuc_0731, methenyltetrahydrofolate cyclohydrolase / 5,10-methylenetetrahydrofolate dehydrogenase (NADP+)		
m4C	737025	-		upstream Pnuc_0733, PAS/PAC sensor signal transduction histidine kinase		
m4C	737919	+		Pnuc_0735, pyruvate dehydrogenase complex dihydrolipoamide acetyltransferase		
m4C	738620	-		upstream Pnuc_0733, PAS/PAC sensor signal transduction histidine kinase		
m6A	739406	+	CTAYNNNNNNNTRTC	Pnuc_0736, dihydrolipoamide dehydrogenase		
m6A	739416	-	GAYANNNNNNNRTAG	upstream Pnuc_0733, PAS/PAC sensor signal transduction histidine kinase		
m6A	741400	+		upstream Pnuc_0738, protein of unknown function DUF6, transmembrane		
m6A	741643	+	CTAYNNNNNNNTRTC	Pnuc_0738, protein of unknown function DUF6, transmembrane		
m6A	741653	-	GAYANNNNNNNRTAG	upstream Pnuc_0737, phasin family protein		
m4C	743185	-		upstream Pnuc_0737, phasin family protein		
m6A	750406	+	GAYANNNNNNNRTAG	upstream Pnuc_0753, protein of unknown function DUF328		
m6A	750416	-	CTAYNNNNNNNTRTC	Pnuc_0747, DSBA oxidoreductase		
m6A	757609	+		upstream Pnuc_0757, transcriptional regulator, GntR family		
m4C	760193	+		Pnuc_0758, succinate dehydrogenase subunit C		
m6A	762421	+		Pnuc_0761, succinate dehydrogenase subunit B		
m6A	762452	+	CTAYNNNNNNNTRTC	Pnuc_0761, succinate dehydrogenase subunit B		
m6A	762462	-	GAYANNNNNNNRTAG	upstream Pnuc_0756, malate dehydrogenase (NAD)		
m6A	767599	+		Pnuc_0767, 3-isopropylmalate dehydrogenase		
m4C	767953	+		Pnuc_0767, 3-isopropylmalate dehydrogenase		
m6A	768513	+	CTAYNNNNNNNTRTC	Pnuc_0768, aspartate semialdehyde dehydrogenase		
m6A	768523	-	GAYANNNNNNNRTAG	upstream Pnuc_0756, malate dehydrogenase (NAD)		
m4C	769803	+		Pnuc_0769, Tfp pilus assembly protein FimV-like protein		
m6A	771608	+		Pnuc_0770, tRNA pseudouridine synthase A		
m6A	774540	+	CTAYNNNNNNNTRTC	Pnuc_0774, acetyl-CoA carboxylase carboxyltransferase subunit alpha		
m6A	774550	-	GAYANNNNNNNRTAG	upstream Pnuc_0756, malate dehydrogenase (NAD)		
m6A	783973	+	GAYANNNNNNNRTAG	Pnuc_0784, OmpA/MotB domain protein		
m6A	783983	-	CTAYNNNNNNNTRTC	upstream Pnuc_0782, molybdate ABC transporter, inner membrane subunit		
m4C	784659	+		Pnuc_0785, hypothetical protein		
m6A	784667	-		upstream Pnuc_0782, molybdate ABC transporter, inner membrane subunit		
m6A	786874	+	GAYANNNNNNNRTAG	upstream Pnuc_0788, protein of unknown function DUF395, YeeE/YedE		
m6A	786884	-	CTAYNNNNNNNTRTC	Pnuc_0787, cytochrome c family protein		
m6A	789389	+	GAYANNNNNNNRTAG	Pnuc_0792, Rhodanese domain protein		
m6A	789399	-	CTAYNNNNNNNTRTC	upstream Pnuc_0790, hypothetical protein		
m6A	793988	+	CTAYNNNNNNNTRTC	upstream Pnuc_0800, beta-lactamase domain protein		
m4C	794908	-		upstream Pnuc_0799, FAD-dependent pyridine nucleotide-disulfide oxidoreductase		
m4C	797305	+		Pnuc_0802, sulfur dehydrogenase subunit SoxD		
m6A	802163	+	GAYANNNNNNNRTAG	Pnuc_0807, sulfate thiol esterase SoxB		
m6A	802173	-	CTAYNNNNNNNTRTC	upstream Pnuc_0799, FAD-dependent pyridine nucleotide-disulfide oxidoreductase		
m4C	812378	-		upstream Pnuc_0818, acyl-CoA dehydrogenase domain protein		
m4C	814873	+		upstream Pnuc_0825, 3-oxoacid CoA-transferase, A subunit		
m6A	815395	+	GAYANNNNNNNRTAG	upstream Pnuc_0825, 3-oxoacid CoA-transferase, A subunit		
m4C	817957	-		upstream Pnuc_0824, Electron-transferring-flavoprotein dehydrogenase		
m4C	818014	+		Pnuc_0825, 3-oxoacid CoA-transferase, A subunit		

m4C	819483	-		upstream Pnuc_0824, Electron-transferring-flavoprotein dehydrogenase	
m6A	820903	+	CTAYNNNNNNNTRTC	Pnuc_0827, cation diffusion facilitator family transporter	
m6A	820913	-	GAYANNNNNNNRTAG	upstream Pnuc_0824, Electron-transferring-flavoprotein dehydrogenase	
m4C	825453	+		Pnuc_0830, bacterial translation initiation factor 3(bIF-3)	
m6A	837877	+	GAYANNNNNNNRTAG	Pnuc_0842, dihydrolipoamide dehydrogenase	
m6A	837887	-	CTAYNNNNNNNTRTC	upstream Pnuc_0839, DNA binding domain, excisionase family	
m4C	843785	+		Pnuc_0848, NlpBDapX family lipoprotein	
m4C	843787	+		Pnuc_0848, NlpBDapX family lipoprotein	
m4C	846473	-		upstream Pnuc_0850, Cupin 4 family protein	
m4C	849590	-		Pnuc_0855, UDP-2,3-diacylglucosamine hydrolase	
m4C	852244	-		upstream Pnuc_0857, Tetratricopeptide TPR_2 repeat protein	
m4C	855676	-		upstream Pnuc_0857, Tetratricopeptide TPR_2 repeat protein	
m6A	856210	+	GAYANNNNNNNRTAG	Pnuc_0862, aspartate kinase	
m6A	856220	-	CTAYNNNNNNNTRTC	upstream Pnuc_0857, Tetratricopeptide TPR_2 repeat protein	
m4C	862259	-		upstream Pnuc_0867, pseudo	
m6A	864766	+	CTAYNNNNNNNTRTC	Pnuc_0870, transporter, hydrophobe/amphiphile efflux-1(HAE1) family	
m6A	864776	-	GAYANNNNNNNRTAG	upstream Pnuc_0865, transcriptional regulator, XRE family	
m6A	865018	+	CTAYNNNNNNNTRTC	Pnuc_0870, transporter, hydrophobe/amphiphile efflux-1(HAE1) family	
m6A	865028	-	GAYANNNNNNNRTAG	upstream Pnuc_0865, transcriptional regulator, XRE family	
m6A	868410	+	GAYANNNNNNNRTAG	Pnuc_0872, Formyl-CoA transferase	
m6A	873168	+	CTAYNNNNNNNTRTC	Pnuc_0879, putative transmembrane protein	
m6A	873178	-	GAYANNNNNNNRTAG	upstream Pnuc_0875, hypothetical protein	
m4C	878961	-		Pnuc_0885, conserved hypothetical protein	
m6A	882214	+	GAYANNNNNNNRTAG	Pnuc_0889, phosphomethylpyrimidine kinase	
m6A	882224	-	CTAYNNNNNNNTRTC	upstream Pnuc_R0023, tRNA-Asn	
m4C	889815	+		upstream Pnuc_0900, DNA topoisomerase IV subunit B	
m6A	894883	+	GAYANNNNNNNRTAG	Pnuc_0901, DNA topoisomerase IV subunit A	
m6A	894893	-	CTAYNNNNNNNTRTC	upstream Pnuc_0899, Formyl-CoA transferase	
m4C	896466	+		Pnuc_0901, DNA topoisomerase IV subunit A	
m6A	898244	-	GAYANNNNNNNRTAG	upstream Pnuc_0902, predicted sulfurylase subunit, molybdopterin cytosine dinucleotide biosynthesis	
m6A	903152	+	GAYANNNNNNNRTAG	upstream Pnuc_0910, methylmalonyl-CoA mutase	
m6A	903162	-	CTAYNNNNNNNTRTC	upstream Pnuc_0909, transcriptional regulator, GntR family	
m6A	905502	+		Pnuc_0911, LAO/AO transport system ATPase	
m4C	906279	-		upstream Pnuc_0909, transcriptional regulator, GntR family	
m4C	907862	+		Pnuc_0912, carboxyl transferase	
m6A	909439	+	CTAYNNNNNNNTRTC	Pnuc_0913, acetyl-CoA carboxylase, biotin carboxylase	
m6A	909449	-	GAYANNNNNNNRTAG	upstream Pnuc_0909, transcriptional regulator, GntR family	
m4C	918085	+		Pnuc_0923, poly(R)-hydroxyalkanoic acid synthase, class I	
m4C	925188	+		upstream Pnuc_0930, 2-C-methyl-D-erythritol 4-phosphate cytidylyltransferase	
m4C	929165	-		upstream Pnuc_0929, transcription-repair coupling factor	
m6A	933218	-		upstream Pnuc_0929, transcription-repair coupling factor	
m4C	933555	+		Pnuc_0935, ATP-dependent Clp protease ATP-binding subunit ClpX	
m4C	934509	-		upstream Pnuc_0929, transcription-repair coupling factor	
m4C	934696	-		upstream Pnuc_0929, transcription-repair coupling factor	

m4C	934777	-		upstream Pnuc_0929, transcription-repair coupling factor
m4C	936875	-		upstream Pnuc_0929, transcription-repair coupling factor
m6A	937001	-	GAYANNNNNNNNRTAG	upstream Pnuc_0929, transcription-repair coupling factor
m4C	939907	+		Pnuc_0940, phosphoribosylformylglycinamidine synthase
m4C	943646	-		upstream Pnuc_0938, Arylesterase
m6A	946091	+	GAYANNNNNNNNRTAG	upstream Pnuc_0945, CTP synthase
m4C	952437	-		Pnuc_0949, Hsp33 protein
m4C	952855	+		upstream Pnuc_0950, glutamyl-tRNA synthetase
m6A	959025	-		Pnuc_0957, hypothetical protein
m6A	959740	+	GAYANNNNNNNNRTAG	Pnuc_0958, 2-hydroxy-3-oxopropionate reductase
m6A	959750	-	CTAYNNNNNNNNTRTC	upstream Pnuc_0957, hypothetical protein
m4C	963789	-		Pnuc_0963, Ankyrin
m6A	966942	+	CTAYNNNNNNNNTRTC	upstream Pnuc_0968, glycine cleavage T protein (aminomethyl transferase)
m6A	967979	+	CTAYNNNNNNNNTRTC	Pnuc_0968, glycine cleavage T protein (aminomethyl transferase)
m6A	967989	-	GAYANNNNNNNNRTAG	upstream Pnuc_0967, aminodeoxychorismate lyase
m4C	969007	-		upstream Pnuc_0967, aminodeoxychorismate lyase
m4C	973870	-		Pnuc_0976, major facilitator superfamily MFS_1
m6A	974583	+		upstream Pnuc_0980, flavodoxin/nitric oxide synthase
m6A	976832	+		upstream Pnuc_0980, flavodoxin/nitric oxide synthase
m6A	979516	+		Pnuc_0982, FAD linked oxidase domain protein
m4C	980292	-		Pnuc_0983, alpha/beta hydrolase fold protein
m6A	980984	+	GAYANNNNNNNNRTAG	upstream Pnuc_0999, ABC-type transporter, periplasmic component, NitT family
m6A	980994	-	CTAYNNNNNNNNTRTC	Pnuc_0983, alpha/beta hydrolase fold protein
m4C	982136	+		upstream Pnuc_0999, ABC-type transporter, periplasmic component, NitT family
m6A	982286	+	CTAYNNNNNNNNTRTC	upstream Pnuc_0999, ABC-type transporter, periplasmic component, NitT family
m6A	982296	-	GAYANNNNNNNNRTAG	Pnuc_0986, hypothetical protein
m4C	986038	-		upstream Pnuc_0990, amino acid ABC transporter substrate-binding protein, PAAT family
m6A	986047	-		upstream Pnuc_0990, amino acid ABC transporter substrate-binding protein, PAAT family
m4C	987808	+		upstream Pnuc_0999, ABC-type transporter, periplasmic component, NitT family
m6A	988136	+	GAYANNNNNNNNRTAG	upstream Pnuc_0999, ABC-type transporter, periplasmic component, NitT family
m6A	988146	-	CTAYNNNNNNNNTRTC	Pnuc_0992, Choloylglycine hydrolase
m4C	991025	-		Pnuc_0995, nitrate ABC transporter, ATPase subunits C and D
m6A	995404	+	CTAYNNNNNNNNTRTC	Pnuc_0999, ABC-type transporter, periplasmic component, NitT family
m6A	995414	-		upstream Pnuc_0998, uroporphyrin-III C-methyltransferase
m6A	996739	+		Pnuc_1000, assimilatory nitrite reductase (NAD(P)H) large subunit precursor
m6A	1008651	+	CTAYNNNNNNNNTRTC	Pnuc_1010, TonB-dependent receptor
m6A	1008661	-	GAYANNNNNNNNRTAG	upstream Pnuc_R0031, tRNA-Pro2
m4C	1009686	+		upstream Pnuc_1015, protein of unknown function UPF0044
m4C	1010247	+		upstream Pnuc_1015, protein of unknown function UPF0044
m4C	1015036	-		Pnuc_1017, transcription elongation factor GreA
m4C	1015612	-		Pnuc_1018, carbamoyl-phosphate synthase large subunit
m4C	1016688	+		upstream Pnuc_1023, Methyltransferase type 11
m6A	1017202	+		upstream Pnuc_1023, Methyltransferase type 11
m6A	1019053	+	CTAYNNNNNNNNTRTC	upstream Pnuc_1023, Methyltransferase type 11

m6A	1019063	-	GAYANNNNNNNRTAG	Pnuc_1019, carbamoyl-phosphate synthase small subunit		
m6A	1020256	+	CTAYNNNNNNNTRTC	upstream Pnuc_1023, Methyltransferase type 11		
m6A	1020266	-	GAYANNNNNNNRTAG	upstream Pnuc_1019, carbamoyl-phosphate synthase small subunit		
m6A	1021303	+	CTAYNNNNNNNTRTC	upstream Pnuc_1023, Methyltransferase type 11		
m6A	1021312	+		upstream Pnuc_1023, Methyltransferase type 11		
m6A	1021313	-	GAYANNNNNNNRTAG	Pnuc_1020, propionyl-CoA synthetase		
m6A	1022212	-		Pnuc_1021, Lytic transglycosylase, catalytic		
m6A	1025742	+		upstream Pnuc_1025, efflux transporter, RND family, MFP subunit		
m6A	1027462	+	GAYANNNNNNNRTAG	Pnuc_1026, acriflavin resistance protein		
m6A	1027472	-	CTAYNNNNNNNTRTC	upstream Pnuc_1022, Hydroxyacylglutathione hydrolase		
m4C	1032904	-		Pnuc_1029, hypothetical protein		
m6A	1033357	+		upstream Pnuc_1032, Amidase		
m6A	1034060	+	CTAYNNNNNNNTRTC	upstream Pnuc_1032, Amidase		
m6A	1034070	-	GAYANNNNNNNRTAG	Pnuc_1031, hypothetical protein		
m4C	1034143	+		upstream Pnuc_1032, Amidase		
m4C	1036580	+		upstream Pnuc_1035, methylmalonate-semialdehyde dehydrogenase (acylating)		
m4C	1037210	+		upstream Pnuc_1035, methylmalonate-semialdehyde dehydrogenase (acylating)		
m4C	1037402	-		Pnuc_1034, NADH:flavin oxidoreductase/NADH oxidase		
m4C	1038133	+		upstream Pnuc_1035, methylmalonate-semialdehyde dehydrogenase (acylating)		
m4C	1039836	+		Pnuc_1035, methylmalonate-semialdehyde dehydrogenase (acylating)		
m6A	1047413	-		Pnuc_1043, NADH dehydrogenase subunit I		
m4C	1053797	+		upstream Pnuc_1057, putative lipoprotein		
m6A	1055538	+	GAYANNNNNNNRTAG	upstream Pnuc_1057, putative lipoprotein		
m4C	1058121	-		Pnuc_1054, Alcohol dehydrogenase, zinc-binding domain protein		
m4C	1059733	+		upstream Pnuc_1057, putative lipoprotein		
m4C	1063511	-		Pnuc_1059, CDP-diacylglycerol-serine O-phosphatidyltransferase		
m4C	1068845	+		Pnuc_1064, RNA polymerase, sigma-24 subunit, RpoE		
m6A	1075780	+	GAYANNNNNNNRTAG	upstream Pnuc_1075, transcription elongation factor GreB		
m6A	1075790	-	CTAYNNNNNNNTRTC	Pnuc_1071, hypothetical protein		
m6A	1076951	+	CTAYNNNNNNNTRTC	upstream Pnuc_1075, transcription elongation factor GreB		
m6A	1076961	-	GAYANNNNNNNRTAG	Pnuc_1073, ABC transporter related protein		
m4C	1082483	+		upstream Pnuc_1080, RNAse PH		
m6A	1092756	-		upstream Pnuc_1085, integral membrane sensor signal transduction histidine kinase		
m6A	1093084	+	GAYANNNNNNNRTAG	Pnuc_1087, hypothetical protein		
m6A	1093094	-	CTAYNNNNNNNTRTC	upstream Pnuc_1085, integral membrane sensor signal transduction histidine kinase		
m6A	1095712	+	CTAYNNNNNNNTRTC	upstream Pnuc_1091, glucose-6-phosphate isomerase		
m6A	1095722	-	GAYANNNNNNNRTAG	upstream Pnuc_1089, hypothetical protein		
m4C	1098640	-		upstream Pnuc_1090, FAD dependent oxidoreductase		
m4C	1099741	+		Pnuc_1092, phosphomannomutase		
m4C	1101992	-		Pnuc_1094, Polypeptide-transport-associated domain protein, ShlB-type		
m6A	1108023	+	GAYANNNNNNNRTAG	upstream Pnuc_1097, hypothetical protein		
m6A	1108033	-	CTAYNNNNNNNTRTC	Pnuc_1095, outer membrane autotransporter barrel domain protein		
m6A	1109515	+	CTAYNNNNNNNTRTC	upstream Pnuc_1097, hypothetical protein		
m6A	1109525	-	GAYANNNNNNNRTAG	Pnuc_1095, outer membrane autotransporter barrel domain protein		

m6A	1111707	+	GAYANNNNNNNRTAG	upstream Pnuc_1097, hypothetical protein		
m6A	1111717	-	CTAYNNNNNNNTRTC	Pnuc_1095, outer membrane autotransporter barrel domain protein		
m6A	1115916	+	GAYANNNNNNNRTAG	upstream Pnuc_1097, hypothetical protein		
m6A	1115926	-	CTAYNNNNNNNTRTC	Pnuc_1095, outer membrane autotransporter barrel domain protein		
m4C	1116271	-		Pnuc_1095, outer membrane autotransporter barrel domain protein		
m4C	1116515	+		upstream Pnuc_1097, hypothetical protein		
m4C	1118009	+		upstream Pnuc_1097, hypothetical protein		
m6A	1120460	+		upstream Pnuc_1097, hypothetical protein		
m6A	1128653	-		Pnuc_1095, outer membrane autotransporter barrel domain protein		
m6A	1134205	-		upstream Pnuc_1095, outer membrane autotransporter barrel domain protein		
m4C	1136504	-		Pnuc_1096, sulfotransferase		
m4C	1137761	+		upstream Pnuc_1097, hypothetical protein		
m4C	1138186	+		upstream Pnuc_1097, hypothetical protein		
m6A	1143445	+		upstream Pnuc_1101, protein of unknown function DUF583		
m6A	1143575	-		upstream Pnuc_1100, peptidase M48, Ste24p		
m6A	1150445	+	GAYANNNNNNNRTAG	upstream Pnuc_1112, protein of unknown function DUF1080		
m6A	1150455	-	CTAYNNNNNNNTRTC	Pnuc_1107, transcriptional modulator of MazE/toxin, MazF		
m6A	1151487	+	GAYANNNNNNNRTAG	upstream Pnuc_1112, protein of unknown function DUF1080		
m6A	1151497	-	CTAYNNNNNNNTRTC	Pnuc_1109, NAD-dependent formate dehydrogenase iron-sulfur protein (catalytic activity)		
m6A	1156356	-	GAYANNNNNNNRTAG	Pnuc_1111, protein of unknown function DUF748		
m6A	1157066	-		Pnuc_1111, protein of unknown function DUF748		
m6A	1159304	+	GAYANNNNNNNRTAG	Pnuc_1112, protein of unknown function DUF1080		
m6A	1159314	-	CTAYNNNNNNNTRTC	upstream Pnuc_1111, protein of unknown function DUF748		
m6A	1167044	+	CTAYNNNNNNNTRTC	Pnuc_1122, Exonuclease, RNase T and DNA polymerase III		
m6A	1167054	-	GAYANNNNNNNRTAG	upstream Pnuc_1121, hypothetical protein		
m4C	1169873	+		upstream Pnuc_1127, hypothetical protein		
m6A	1173977	+	GAYANNNNNNNRTAG	Pnuc_1127, hypothetical protein		
m6A	1173987	-	CTAYNNNNNNNTRTC	upstream Pnuc_1126, hypothetical protein		
m6A	1177319	+	GAYANNNNNNNRTAG	upstream Pnuc_1131, phage transcriptional regulator, AlpA		
m6A	1177329	-	CTAYNNNNNNNTRTC	Pnuc_1130, protein of unknown function DUF1376		
m4C	1190609	+		Pnuc_1139, fumarase		
m6A	1191165	+	CTAYNNNNNNNTRTC	Pnuc_1140, glutamate racemase		
m6A	1191175	-	GAYANNNNNNNRTAG	upstream Pnuc_1138, acetyl-coenzyme A synthetase		
m4C	1192583	+		Pnuc_1142, outer membrane transport energization protein ExbB		
m6A	1199103	+		upstream Pnuc_1150, Penicillin amidase		
m6A	1199870	+	GAYANNNNNNNRTAG	upstream Pnuc_1150, Penicillin amidase		
m6A	1199880	-	CTAYNNNNNNNTRTC	Pnuc_1149, filamentous hemagglutinin family outer membrane protein		
m6A	1206854	+	CTAYNNNNNNNTRTC	Pnuc_1150, Penicillin amidase		
m6A	1206864	-	GAYANNNNNNNRTAG	upstream Pnuc_R0036, tRNA-Leu		
m4C	1210903	-		Pnuc_1158, aldehyde oxidase and xanthine dehydrogenase, molybdopterin binding protein		
m4C	1216436	+		Pnuc_1163, gamma-glutamyltransferase 2, Threonine peptidase, MEROPS family T03		
m6A	1218238	+	CTAYNNNNNNNTRTC	upstream Pnuc_1167, Cellulase		
m6A	1218248	-	GAYANNNNNNNRTAG	Pnuc_1164, conserved hypothetical protein		
m4C	1225329	-		Pnuc_1170, cellulose synthase operon C domain protein		

m6A	1228000	+	GAYANNNNNNNRTAG	upstream Pnuc_1176, hypothetical protein		
m6A	1228010	-	CTAYNNNNNNNTRTC	Pnuc_1170, cellulose synthase operon C domain protein		
m6A	1228683	+	GAYANNNNNNNRTAG	upstream Pnuc_1176, hypothetical protein		
m6A	1228693	-	CTAYNNNNNNNTRTC	Pnuc_1170, cellulose synthase operon C domain protein		
m6A	1228990	+	CTAYNNNNNNNTRTC	upstream Pnuc_1176, hypothetical protein		
m4C	1230189	-		Pnuc_1171, Cellulose synthase (UDP-forming)		
m4C	1235017	+		Pnuc_1176, hypothetical protein		
m4C	1235061	-		upstream Pnuc_1175, metal dependent phosphohydrolase		
m6A	1236722	-	GAYANNNNNNNRTAG	upstream Pnuc_1177, tRNA-U16,U17-dihydrouridine synthase		
m6A	1239968	+	GAYANNNNNNNRTAG	upstream Pnuc_1184, Amidase		
m6A	1239978	-	CTAYNNNNNNNTRTC	Pnuc_1180, putative ABC transporter, periplasmic protein		
m6A	1240127	+	GAYANNNNNNNRTAG	upstream Pnuc_1184, Amidase		
m4C	1246490	+		Pnuc_1187, ABC transporter related protein		
m6A	1247136	+		upstream Pnuc_1210, Glutamate synthase (NADPH)		
m6A	1247968	+	CTAYNNNNNNNTRTC	upstream Pnuc_1210, Glutamate synthase (NADPH)		
m6A	1247978	-	GAYANNNNNNNRTAG	Pnuc_1190, urease accessory protein UreG		
m4C	1251636	+		upstream Pnuc_1210, Glutamate synthase (NADPH)		
m6A	1252457	+	GAYANNNNNNNRTAG	upstream Pnuc_1210, Glutamate synthase (NADPH)		
m6A	1252467	-	CTAYNNNNNNNTRTC	Pnuc_1196, urease, gamma subunit		
m4C	1257725	+		upstream Pnuc_1210, Glutamate synthase (NADPH)		
m4C	1260318	+		upstream Pnuc_1210, Glutamate synthase (NADPH)		
m4C	1260888	+		upstream Pnuc_1210, Glutamate synthase (NADPH)		
m4C	1261107	+		upstream Pnuc_1210, Glutamate synthase (NADPH)		
m6A	1262425	+	CTAYNNNNNNNTRTC	upstream Pnuc_1210, Glutamate synthase (NADPH)		
m6A	1262435	-	GAYANNNNNNNRTAG	Pnuc_1205, protein of unknown function DUF1501		
m6A	1263897	-		Pnuc_1206, conserved hypothetical protein		
m6A	1269593	+		upstream Pnuc_1218, transglutaminase, N-terminal domain protein		
m4C	1271444	+		upstream Pnuc_1218, transglutaminase, N-terminal domain protein		
m4C	1283494	+		Pnuc_1223, major facilitator superfamily MFS_1		
m4C	1284569	+		upstream Pnuc_1232, Patatin		
m4C	1288419	+		upstream Pnuc_1232, Patatin		
m4C	1296340	-		Pnuc_1233, NLP/P60 protein		
m6A	1296429	+	CTAYNNNNNNNTRTC	upstream Pnuc_1238, Enoyl-[acyl-carrier-protein] reductase (NADH)		
m6A	1296439	-	GAYANNNNNNNRTAG	upstream Pnuc_1233, NLP/P60 protein		
m6A	1301267	-		Pnuc_1237, extracellular solute-binding protein, family 5		
m4C	1303180	+		Pnuc_1239, chromate transporter, chromate ion transporter(CHR) family		
m4C	1304745	+		Pnuc_1240, Integrase, catalytic region		
m4C	1305997	-		upstream Pnuc_1237, extracellular solute-binding protein, family 5		
m6A	1307149	+	GAYANNNNNNNRTAG	Pnuc_1242, MmgE/PrpD family protein		
m6A	1307159	-	CTAYNNNNNNNTRTC	upstream Pnuc_1237, extracellular solute-binding protein, family 5		
m6A	1311414	+	CTAYNNNNNNNTRTC	upstream Pnuc_1249, integrase, catalytic region		
m6A	1311424	-	GAYANNNNNNNRTAG	Pnuc_1246, BNR/Asp-box repeat protein		
m6A	1312246	-		Pnuc_1247, glycosyl transferase, family 39		
m4C	1313486	+		upstream Pnuc_1249, integrase, catalytic region		

m4C	1315427	+		upstream Pnuc_1251, pseudo		
m6A	1317420	+	CTAYNNNNNNNNTRTC	upstream Pnuc_1262, N-acetylglutamate synthase		
m6A	1317430	-	GAYANNNNNNNNRTAG	Pnuc_1252, Exodeoxyribonuclease III		
m4C	1318201	+		upstream Pnuc_1262, N-acetylglutamate synthase		
m4C	1318723	+		upstream Pnuc_1262, N-acetylglutamate synthase		
m4C	1318907	-		Pnuc_1253, nitrogen metabolism transcriptional regulator, NtrC, Fis family		
m6A	1325390	+		upstream Pnuc_1262, N-acetylglutamate synthase		
m4C	1336966	-		Pnuc_1267, propionate CoA-transferase		
m6A	1341847	+		upstream Pnuc_R0038, tRNA-Leu		
m4C	1345263	+		upstream Pnuc_1278, hypothetical protein		
m4C	1350920	-		Pnuc_1280, malate synthase G		
m4C	1357004	+		upstream Pnuc_1294, protein of unknown function UPF0005		
m4C	1363387	+		upstream Pnuc_1294, protein of unknown function UPF0005		
m4C	1366237	-		upstream Pnuc_1293, nucleoside diphosphate kinase		
m4C	1369370	+		upstream Pnuc_1307, thioredoxin		
m4C	1378180	-		Pnuc_1304, Tetratricopeptide TPR_2 repeat protein		
m4C	1384960	+		upstream Pnuc_1307, thioredoxin		
m4C	1386002	-		upstream Pnuc_1306, DNA helicase/exodeoxyribonuclease V, subunit B		
m6A	1388390	-		upstream Pnuc_1306, DNA helicase/exodeoxyribonuclease V, subunit B		
m6A	1395251	+		upstream Pnuc_1318, Enoyl-CoA hydratase/isomerase		
m6A	1397943	-		Pnuc_1319, Lytic transglycosylase, catalytic		
m4C	1400832	-		upstream Pnuc_1322, conserved hypothetical protein		
m4C	1405083	+		upstream Pnuc_1331, Carbohydrate-selective porin OprB		
m6A	1410626	+	GAYANNNNNNNNRTAG	Pnuc_1332, negative transcriptional regulator		
m6A	1413234	+		upstream Pnuc_1336, putative sulfate transport system substrate-binding protein		
m6A	1416762	+	CTAYNNNNNNNNTRTC	Pnuc_1340, thioesterase superfamily protein		
m6A	1416772	-	GAYANNNNNNNNRTAG	upstream Pnuc_1338, hypothetical protein		
m6A	1417579	+	GAYANNNNNNNNRTAG	Pnuc_1341, conserved hypothetical protein		
m6A	1417589	-	CTAYNNNNNNNNTRTC	upstream Pnuc_1338, hypothetical protein		
m6A	1419263	+	CTAYNNNNNNNNTRTC	Pnuc_1343, transglutaminase domain protein		
m6A	1419273	-	GAYANNNNNNNNRTAG	upstream Pnuc_1338, hypothetical protein		
m6A	1422263	+	CTAYNNNNNNNNTRTC	upstream Pnuc_1347, protein of unknown function DUF1330		
m6A	1422273	-	GAYANNNNNNNNRTAG	upstream Pnuc_1346, conserved hypothetical protein		
m4C	1423119	+		Pnuc_1349, Uncharacterized protein UPF0065		
m6A	1432389	-		upstream Pnuc_1359, conserved hypothetical protein 730		
m4C	1433506	+		upstream Pnuc_1363, carbohydrate kinase, YjeF related protein		
m6A	1434445	+	CTAYNNNNNNNNTRTC	upstream Pnuc_1363, carbohydrate kinase, YjeF related protein		
m6A	1434455	-	GAYANNNNNNNNRTAG	Pnuc_1362, RNA-metabolising metallo-beta-lactamase		
m6A	1436527	+		Pnuc_1364, protein of unknown function DUF6, transmembrane		
m4C	1442524	-		upstream Pnuc_1370, protein of unknown function DUF6, transmembrane		
m6A	1448329	+		Pnuc_1378, FAD-dependent pyridine nucleotide-disulfide oxidoreductase		
m6A	1449009	+		Pnuc_1378, FAD-dependent pyridine nucleotide-disulfide oxidoreductase		
m4C	1451114	-		Pnuc_1381, type II and III secretion system protein		
m6A	1451211	+		upstream Pnuc_1382, DSBA oxidoreductase		

m6A	1451215	+		upstream Pnuc_1382, DSBA oxidoreductase
m4C	1451602	+		upstream Pnuc_1382, DSBA oxidoreductase
m4C	1453033	-		Pnuc_1381, type II and III secretion system protein
m4C	1454705	+		upstream Pnuc_1388, acyl-CoA dehydrogenase domain protein
m4C	1462285	-		Pnuc_1391, Endonuclease/exonuclease/phosphatase
m4C	1463215	-		upstream Pnuc_1391, Endonuclease/exonuclease/phosphatase
m6A	1464604	-		Pnuc_1394, hypothetical protein
m6A	1464729	+	GAYANNNNNNNNRTAG	upstream Pnuc_1407, heavy metal translocating P-type ATPase
m6A	1464739	-	CTAYNNNNNNNNTRTC	Pnuc_1395, formate dehydrogenase gamma subunit
m4C	1466348	+		upstream Pnuc_1407, heavy metal translocating P-type ATPase
m4C	1470475	+		upstream Pnuc_1407, heavy metal translocating P-type ATPase
m6A	1473645	+	GAYANNNNNNNNRTAG	upstream Pnuc_1407, heavy metal translocating P-type ATPase
m6A	1473655	-	CTAYNNNNNNNNTRTC	Pnuc_1402, conserved hypothetical protein
m4C	1476780	-		upstream Pnuc_1405, protein of unknown function DUF47
m4C	1479800	+		upstream Pnuc_1409, 4-amino-4-deoxy-L-arabinose transferase and related glycosyltransferase of PMT family-like protein
m4C	1479959	+		upstream Pnuc_1409, 4-amino-4-deoxy-L-arabinose transferase and related glycosyltransferase of PMT family-like protein
m6A	1488814	+		upstream Pnuc_1426, SsrA-binding protein
m6A	1488824	-		Pnuc_1418, Radical SAM domain protein
m4C	1496040	+		upstream Pnuc_1426, SsrA-binding protein
m6A	1496168	+	CTAYNNNNNNNNTRTC	upstream Pnuc_1426, SsrA-binding protein
m6A	1496178	-	GAYANNNNNNNNRTAG	Pnuc_1425, cyclase/dehydrase
m4C	1497236	-		upstream Pnuc_1425, cyclase/dehydrase
m4C	1498737	-		upstream Pnuc_1425, cyclase/dehydrase
m6A	1503143	-		Pnuc_1433, phosphoenolpyruvate synthase
m6A	1505598	+	CTAYNNNNNNNNTRTC	Pnuc_1434, protein of unknown function DUF299
m6A	1505608	-	GAYANNNNNNNNRTAG	Pnuc_1433, phosphoenolpyruvate synthase
m4C	1506886	-		Pnuc_1435, secretory lipase
m6A	1509758	+		upstream Pnuc_1452, methionine aminopeptidase, type I
m4C	1515771	+		upstream Pnuc_1452, methionine aminopeptidase, type I
m6A	1519366	-	GAYANNNNNNNNRTAG	Pnuc_1448, ribosome recycling factor
m6A	1532526	+		upstream Pnuc_1458, 2,3,4,5-tetrahydropyridine-2,6-dicarboxylate N-succinyltransferase
m4C	1536151	-		upstream Pnuc_1457, chromosome segregation protein SMC
m6A	1545568	-		Pnuc_1468, lipoprotein releasing system, transmembrane protein, LolC/E family
m4C	1546532	-		upstream Pnuc_1468, lipoprotein releasing system, transmembrane protein, LolC/E family
m4C	1547566	-		upstream Pnuc_1468, lipoprotein releasing system, transmembrane protein, LolC/E family
m6A	1548857	+		Pnuc_1470, exonuclease RecJ
m6A	1552414	+	GAYANNNNNNNNRTAG	upstream Pnuc_1477, peptide deformylase
m6A	1552424	-	CTAYNNNNNNNNTRTC	Pnuc_1473, hypothetical protein
m6A	1553318	+		upstream Pnuc_1477, peptide deformylase
m4C	1554796	-		Pnuc_1476, cysteine synthase A
m4C	1556507	+		Pnuc_1479, Ferredoxin-NADP(+) reductase
m6A	1556599	+		Pnuc_1479, Ferredoxin-NADP(+) reductase
m6A	1556884	-		upstream Pnuc_1478, disulfide bond formation protein DsbB
m6A	1557612	+	CTAYNNNNNNNNTRTC	Pnuc_1480, protein of unknown function DUF81

m6A	1557622	-	GAYANNNNNNNNRTAG	upstream Pnuc_1478, disulfide bond formation protein DsbB		
m4C	1569924	-		Pnuc_1494, transcriptional regulator, BadM/Rrf2 family		
m4C	1570062	-		Pnuc_1494, transcriptional regulator, BadM/Rrf2 family		
m4C	1570576	-		Pnuc_1495, Excinuclease ABC subunit B		
m4C	1570822	-		Pnuc_1495, Excinuclease ABC subunit B		
m6A	1570852	-	GAYANNNNNNNNRTAG	Pnuc_1495, Excinuclease ABC subunit B		
m4C	1572033	-		Pnuc_1495, Excinuclease ABC subunit B		
m4C	1575003	+		Pnuc_1497, polyhydroxyalkanoate depolymerase,intracellular		
m6A	1576519	+		Pnuc_1500, conserved hypothetical protein		
m4C	1579164	+		upstream Pnuc_1528, conserved hypothetical protein		
m4C	1581561	+		upstream Pnuc_1528, conserved hypothetical protein		
m6A	1582960	+	GAYANNNNNNNNRTAG	upstream Pnuc_1528, conserved hypothetical protein		
m6A	1582970	-	CTAYNNNNNNNNTRTC	Pnuc_1507, major facilitator superfamily MFS_1		
m6A	1591897	+	CTAYNNNNNNNNTRTC	upstream Pnuc_1528, conserved hypothetical protein		
m6A	1591907	-	GAYANNNNNNNNRTAG	Pnuc_1518, heme exporter protein CcmA		
m4C	1595553	-		Pnuc_1524, beta-lactamase domain protein		
m6A	1599496	+	CTAYNNNNNNNNTRTC	Pnuc_1529, histone deacetylase superfamily		
m6A	1599506	-	GAYANNNNNNNNRTAG	upstream Pnuc_1527, hypothetical protein		
m4C	1600146	-		Pnuc_1530, putative lipoprotein		
m6A	1601203	+	GAYANNNNNNNNRTAG	upstream Pnuc_1534, alkyl hydroperoxide reductase/ Thiol specific antioxidant/ Mal allergen		
m6A	1601213	-	CTAYNNNNNNNNTRTC	Pnuc_1531, putative lipoprotein		
m6A	1606433	+	GAYANNNNNNNNRTAG	upstream Pnuc_1544, transcriptional regulator, GntR family		
m6A	1606443	-	CTAYNNNNNNNNTRTC	Pnuc_1538, TRAP C4-dicarboxylate transport system permease DctM subunit		
m6A	1612846	+		upstream Pnuc_1544, transcriptional regulator, GntR family		
m6A	1614081	+		Pnuc_1544, transcriptional regulator, GntR family		
m6A	1617388	+	GAYANNNNNNNNRTAG	Pnuc_1547, protein of unknown function DUF6, transmembrane		
m6A	1617398	-	CTAYNNNNNNNNTRTC	upstream Pnuc_1545, class II aldolase/adducin family protein		
m4C	1618715	+		upstream Pnuc_1553, transcriptional regulator, AsnC family		
m4C	1619966	+		upstream Pnuc_1553, transcriptional regulator, AsnC family		
m6A	1623159	+		upstream Pnuc_1553, transcriptional regulator, AsnC family		
m4C	1626430	+		Pnuc_1554, short-chain dehydrogenase/reductase SDR		
m6A	1628517	+	GAYANNNNNNNNRTAG	upstream Pnuc_1560, transcriptional regulator, LysR family		
m6A	1628527	-	CTAYNNNNNNNNTRTC	Pnuc_1556, Enoyl-CoA hydratase/isomerase		
m6A	1635651	-		upstream Pnuc_1561, major facilitator superfamily MFS_1		
m6A	1638119	+	CTAYNNNNNNNNTRTC	Pnuc_1564, FMN-binding domain protein		
m4C	1646478	+		upstream Pnuc_1581, hypothetical protein		
m6A	1647640	-		Pnuc_1575, anti-ECFsigma factor, ChrR		
m6A	1650038	+		upstream Pnuc_1581, hypothetical protein		
m4C	1654385	+		upstream Pnuc_1585, metallophosphoesterase		
m4C	1654654	+		upstream Pnuc_1585, metallophosphoesterase		
m6A	1655064	-		Pnuc_1584, lyso-ornithine lipid acyltransferase		
m6A	1658012	+	GAYANNNNNNNNRTAG	Pnuc_1588, ornithine-acyl[acyl carrier protein]N-acyltransferase		
m6A	1658022	-	CTAYNNNNNNNNTRTC	upstream Pnuc_1584, lyso-ornithine lipid acyltransferase		
m6A	1660810	+	CTAYNNNNNNNNTRTC	upstream Pnuc_1595, MgtC/SapB transporter		

m6A	1660820	-	GAYANNNNNNNRTAG	Pnuc_1591, MgtC/SapB transporter
m4C	1661237	+		upstream Pnuc_1595, MgtC/SapB transporter
m4C	1668542	+		upstream Pnuc_1606, short-chain dehydrogenase/reductase SDR
m4C	1674039	-		Pnuc_1604, conserved hypothetical protein
m6A	1681242	-		upstream Pnuc_1613, conserved hypothetical membrane spanning protein
m4C	1683718	+		upstream Pnuc_1622, hypothetical protein
m4C	1689135	+		upstream Pnuc_1622, hypothetical protein
m4C	1690534	-		upstream Pnuc_1621, Mg2+ transporter protein, CorA family protein
m4C	1698112	-		Pnuc_1631, Hydroxypyruvate isomerase
m6A	1699838	+	CTAYNNNNNNNTRTC	upstream Pnuc_1636, transcriptional regulator, GntR family
m6A	1699848	-		Pnuc_1633, type III effector Hrp-dependent outer domain protein
m4C	1702669	-		Pnuc_1635, major facilitator superfamily MFS_1
m6A	1705954	+	CTAYNNNNNNNTRTC	Pnuc_1638, protein of unknown function DUF6, transmembrane
m6A	1705964	-	GAYANNNNNNNRTAG	upstream Pnuc_1637, NAD-dependent epimerase/dehydratase
m4C	1707923	+		upstream Pnuc_1642, DSBA oxidoreductase
m6A	1712078	+	CTAYNNNNNNNTRTC	upstream Pnuc_1649, hypothetical protein
m6A	1712088	-	GAYANNNNNNNRTAG	Pnuc_1647, DNA polymerase III, epsilon subunit
m6A	1712822	+	CTAYNNNNNNNTRTC	upstream Pnuc_1649, hypothetical protein
m6A	1712832	-	GAYANNNNNNNRTAG	Pnuc_1647, DNA polymerase III, epsilon subunit
m6A	1714221	-		upstream Pnuc_1648, transcriptional regulator, BadM/Rrf2 family
m4C	1718598	+		Pnuc_1655, FAD dependent oxidoreductase
m4C	1722369	-		upstream Pnuc_1659, protein of unknown function DUF427
m4C	1726936	+		upstream Pnuc_1664, spermine/spermidine N-acetyltransferase
m6A	1730416	-		Pnuc_1667, osmosensitive K+ channel signal transduction histidine kinase
m6A	1736959	+	GAYANNNNNNNRTAG	upstream Pnuc_1678, putative transcriptional regulator, MerR family
m6A	1736969	-	CTAYNNNNNNNTRTC	Pnuc_1670, potassium-transporting ATPase, A subunit
m6A	1738403	+	CTAYNNNNNNNTRTC	upstream Pnuc_1678, putative transcriptional regulator, MerR family
m6A	1738413	-	GAYANNNNNNNRTAG	Pnuc_1672, Heavy metal transport/detoxification protein
m4C	1742637	+		upstream Pnuc_1678, putative transcriptional regulator, MerR family
m4C	1743423	+		Pnuc_1678, putative transcriptional regulator, MerR family
m4C	1748045	-		Pnuc_1681, efflux transporter, RND family, MFP subunit
m6A	1748827	+	GAYANNNNNNNRTAG	upstream Pnuc_1688, hypothetical protein
m6A	1748837	-	CTAYNNNNNNNTRTC	Pnuc_1681, efflux transporter, RND family, MFP subunit
m4C	1749332	-		Pnuc_1682, hypothetical protein
m6A	1758014	+	GAYANNNNNNNRTAG	upstream Pnuc_1702, O-sialoglycoprotein endopeptidase
m6A	1758024	-	CTAYNNNNNNNTRTC	Pnuc_1694, phage integrase family protein
m4C	1765686	+		upstream Pnuc_1702, O-sialoglycoprotein endopeptidase
m4C	1769216	-		upstream Pnuc_1701, SSU ribosomal protein S21P
m6A	1769409	+		Pnuc_1702, O-sialoglycoprotein endopeptidase
m6A	1771008	-		Pnuc_1704, 1-deoxy-D-xylulose-5-phosphate synthase
m4C	1773158	+		upstream Pnuc_1707, Rieske (2Fe-2S) domain protein
m4C	1782299	+		Pnuc_1714, putative transmembrane protein
m4C	1784344	+		upstream Pnuc_1716, valyl-tRNA synthetase
m4C	1789753	-		Pnuc_1719, alanyl-tRNA synthetase

m4C	1792905	+		Pnuc_1721, protein of unknown function DUF112,transmembrane
m6A	1797655	+		Pnuc_1725, acetylornithine aminotransferase apoenzyme
m6A	1797750	+	CTAYNNNNNNNTRTC	Pnuc_1725, acetylornithine aminotransferase apoenzyme
m6A	1797760	-	GAYANNNNNNNRTAG	upstream Pnuc_1723, NAD-dependent epimerase/dehydratase
m4C	1799103	+		upstream Pnuc_1733, DNA replication and repair protein RadC
m6A	1805177	+		Pnuc_1733, DNA replication and repair protein RadC
m6A	1809128	-		Pnuc_1738, formyltetrahydrofolate-dependent phosphoribosylglycinamide formyltransferase
m6A	1809406	+		upstream Pnuc_1739, FMN adenylyltransferase / riboflavin kinase
m4C	1810877	+		Pnuc_1740, Isoleucyl-tRNA synthetase
m4C	1811212	-		upstream Pnuc_1738, formyltetrahydrofolate-dependent phosphoribosylglycinamide formyltransferase
m6A	1813329	-		upstream Pnuc_1738, formyltetrahydrofolate-dependent phosphoribosylglycinamide formyltransferase
m4C	1815519	-		upstream Pnuc_1738, formyltetrahydrofolate-dependent phosphoribosylglycinamide formyltransferase
m6A	1816094	-		upstream Pnuc_1738, formyltetrahydrofolate-dependent phosphoribosylglycinamide formyltransferase
m4C	1816646	-		Pnuc_1745, sodium:dicarboxylate symporter
m4C	1822325	-		upstream Pnuc_1749, ATP-dependent Clp protease adaptor protein ClpS
m6A	1823635	+		upstream Pnuc_1755, conserved hypothetical protein
m6A	1832050	-		Pnuc_1761, phosphoribosylformylglycinamidine cyclo-ligase
m6A	1832665	+	CTAYNNNNNNNTRTC	Pnuc_1762, protein of unknown function UPF0118
m6A	1832675	-	GAYANNNNNNNRTAG	upstream Pnuc_1761, phosphoribosylformylglycinamidine cyclo-ligase
m4C	1840381	+		upstream Pnuc_1773, NAD(+) kinase
m4C	1840741	+		upstream Pnuc_1773, NAD(+) kinase
m6A	1843285	+	GAYANNNNNNNRTAG	upstream Pnuc_1773, NAD(+) kinase
m6A	1843295	-	CTAYNNNNNNNTRTC	Pnuc_1772, heat-inducible transcription repressor HrcA
m4C	1845196	-		upstream Pnuc_1772, heat-inducible transcription repressor HrcA
m4C	1846682	-		Pnuc_1775, (Glutamate-ammonia-ligase) adenylyltransferase
m6A	1850932	-		upstream Pnuc_1775, (Glutamate-ammonia-ligase) adenylyltransferase
m6A	1853226	+	CTAYNNNNNNNTRTC	Pnuc_1777, Nitrilase/cyanide hydratase and apolipoprotein N-acyltransferase
m6A	1853236	-	GAYANNNNNNNRTAG	upstream Pnuc_1775, (Glutamate-ammonia-ligase) adenylyltransferase
m4C	1861820	+		Pnuc_1784, alanine racemase domain protein
m6A	1861824	+	GAYANNNNNNNRTAG	Pnuc_1784, alanine racemase domain protein
m6A	1861834	-	CTAYNNNNNNNTRTC	upstream Pnuc_1780, ATP:cob(I)alamin adenosyltransferase
m4C	1863314	-		Pnuc_1786, 4-hydroxybenzoate octaprenyltransferase
m6A	1865372	+	GAYANNNNNNNRTAG	upstream Pnuc_1788, S-adenosylmethionine-tRNA-ribosyltransferase-isomerase
m6A	1865382	-	CTAYNNNNNNNTRTC	Pnuc_1787, ATP-dependent DNA helicase RecG
m6A	1866093	+		Pnuc_1788, S-adenosylmethionine-tRNA-ribosyltransferase-isomerase
m6A	1867542	+	CTAYNNNNNNNTRTC	Pnuc_1789, tRNA-guanine transglycosylase
m6A	1867552	-	GAYANNNNNNNRTAG	upstream Pnuc_1787, ATP-dependent DNA helicase RecG
m4C	1872268	+		upstream Pnuc_1795, Glutathione S-transferase, N-terminal domain protein
m4C	1873321	+		upstream Pnuc_1795, Glutathione S-transferase, N-terminal domain protein
m6A	1874872	+	GAYANNNNNNNRTAG	upstream Pnuc_1797, alanine dehydrogenase/PNT domain protein
m6A	1874879	+		upstream Pnuc_1797, alanine dehydrogenase/PNT domain protein
m6A	1874882	-	CTAYNNNNNNNTRTC	Pnuc_1796, tRNA (5-methylaminomethyl-2-thiouridylate)-methyltransferase
m4C	1879220	+		Pnuc_1800, hypothetical protein
m6A	1880808	+	GAYANNNNNNNRTAG	upstream Pnuc_1802, hypothetical protein

m6A	1880818	-	CTAYNNNNNNNNTRTC	Pnuc_1801, glycosyl transferase, family 11		
m6A	1881762	+	CTAYNNNNNNNNTRTC	Pnuc_1802, hypothetical protein		
m6A	1881772	-	GAYANNNNNNNNRTAG	upstream Pnuc_1801, glycosyl transferase, family 11		
m6A	1885043	-		Pnuc_1805, chaperonin GroEL		
m4C	1886632	+		Pnuc_1808, two component transcriptional regulator, LuxR family		
m6A	1887429	-		upstream Pnuc_1806, chaperonin Cpn10		
m6A	1894804	+	GAYANNNNNNNNRTAG	upstream Pnuc_1818, branched chain amino acid aminotransferase apoenzyme		
m6A	1894814	-	CTAYNNNNNNNNTRTC	Pnuc_1814, phosphoribosylaminoimidazole-succinocarboxamide synthase		
m4C	1895256	-		Pnuc_1815, fructose-bisphosphate aldolase		
m4C	1895901	+		upstream Pnuc_1818, branched chain amino acid aminotransferase apoenzyme		
m4C	1897764	+		upstream Pnuc_1818, branched chain amino acid aminotransferase apoenzyme		
m6A	1899165	+	CTAYNNNNNNNNTRTC	Pnuc_1818, branched chain amino acid aminotransferase apoenzyme		
m6A	1899175	-	GAYANNNNNNNNRTAG	upstream Pnuc_1817, phosphoglycerate kinase		
m4C	1906490	-		upstream Pnuc_1825, TPR repeat-containing protein		
m4C	1910853	-		upstream Pnuc_1829, Integral membrane protein TerC		
m6A	1911071	-		Pnuc_1830, succinyl-CoA synthetase (ADP-forming) alpha subunit		
m6A	1916434	+	GAYANNNNNNNNRTAG	Pnuc_1835, hypothetical protein		
m6A	1916444	-	CTAYNNNNNNNNTRTC	upstream Pnuc_1834, conserved hypothetical protein		
m6A	1918050	+	GAYANNNNNNNNRTAG	Pnuc_1838, hypothetical protein		
m6A	1921973	+	GAYANNNNNNNNRTAG	upstream Pnuc_1847, secreted protein		
m6A	1921983	-	CTAYNNNNNNNNTRTC	upstream Pnuc_1843, hypothetical protein		
m4C	1924413	+		upstream Pnuc_1847, secreted protein		
m6A	1931364	+		Pnuc_1858, protein of unknown function DUF1504		
m6A	1934625	-		upstream Pnuc_1857, Rhodanese domain protein		
m6A	1935097	+	GAYANNNNNNNNRTAG	Pnuc_1861, CBS domain containing protein		
m6A	1935107	-	CTAYNNNNNNNNTRTC	upstream Pnuc_1857, Rhodanese domain protein		
m6A	1940032	+	CTAYNNNNNNNNTRTC	Pnuc_1865, D-alpha,beta-D-heptose 1,7-bisphosphate phosphatase		
m6A	1940042	-	GAYANNNNNNNNRTAG	upstream Pnuc_1857, Rhodanese domain protein		
m4C	1946837	-		Pnuc_1870, Organic solvent tolerance protein		
m4C	1948095	-		upstream Pnuc_1870, Organic solvent tolerance protein		
m4C	1954595	+		Pnuc_1878, crossover junction endodeoxyribonuclease RuvC		
m4C	1961924	-		upstream Pnuc_1886, LSU ribosomal protein L13P		
m4C	1964457	+		upstream Pnuc_1900, L-threonine ammonia-lyase		
m4C	1965110	+		upstream Pnuc_1900, L-threonine ammonia-lyase		
m6A	1968003	+	CTAYNNNNNNNNTRTC	upstream Pnuc_1900, L-threonine ammonia-lyase		
m6A	1968013	-	GAYANNNNNNNNRTAG	Pnuc_1892, aspartyl-tRNA synthetase		
m6A	1968185	+	GAYANNNNNNNNRTAG	upstream Pnuc_1900, L-threonine ammonia-lyase		
m6A	1968195	-	CTAYNNNNNNNNTRTC	Pnuc_1892, aspartyl-tRNA synthetase		
m6A	1970858	+		upstream Pnuc_1900, L-threonine ammonia-lyase		
m6A	1972754	+		upstream Pnuc_1900, L-threonine ammonia-lyase		
m4C	1974527	-		Pnuc_1899, FAD linked oxidase domain protein		
m6A	1974979	+	GAYANNNNNNNNRTAG	upstream Pnuc_1900, L-threonine ammonia-lyase		
m6A	1974989	-	CTAYNNNNNNNNTRTC	Pnuc_1899, FAD linked oxidase domain protein		
m4C	1978655	-		upstream Pnuc_1899, FAD linked oxidase domain protein		

m4C	1978846	-		upstream Pnuc_1899, FAD linked oxidase domain protein	
m4C	1981099	+		Pnuc_1902, GTP cyclohydrolase I	
m4C	1981571	+		Pnuc_1902, GTP cyclohydrolase I	
m4C	1982756	+		upstream Pnuc_1905, Excinuclease ABC subunit A	
m6A	1984673	-		upstream Pnuc_1904, major facilitator superfamily MFS_1	
m4C	1990207	-		upstream Pnuc_1906, Kef-type potassium/proton antiporter, CPA2 family	
m4C	1994211	-		upstream Pnuc_1906, Kef-type potassium/proton antiporter, CPA2 family	
m4C	1996322	-		Pnuc_1917, DNA-(apurinic or apyrimidinic site) lyase	
m4C	1999383	+		Pnuc_1920, ribose-phosphate pyrophosphokinase	
m4C	1999599	+		Pnuc_1920, ribose-phosphate pyrophosphokinase	
m6A	1999753	+	GAYANNNNNNNRTAG	Pnuc_1920, ribose-phosphate pyrophosphokinase	
m6A	1999763	-	CTAYNNNNNNNTRTC	upstream Pnuc_1917, DNA-(apurinic or apyrimidinic site) lyase	
m6A	2000577	-		upstream Pnuc_1917, DNA-(apurinic or apyrimidinic site) lyase	
m4C	2006549	-		upstream Pnuc_1927, peptidase M16 domain protein	
m6A	2008414	+	GAYANNNNNNNRTAG	upstream Pnuc_1935, conserved hypothetical protein	
m6A	2008424	-	CTAYNNNNNNNTRTC	Pnuc_1931, protoheme IX farnesyltransferase	
m4C	2009132	+		upstream Pnuc_1935, conserved hypothetical protein	
m6A	2014371	+	CTAYNNNNNNNTRTC	upstream Pnuc_1942, phosphoribosyltransferase	
m6A	2014381	-	GAYANNNNNNNRTAG	Pnuc_1939, Cytochrome-c oxidase	
m6A	2014641	+	GAYANNNNNNNRTAG	upstream Pnuc_1942, phosphoribosyltransferase	
m6A	2014651	-	CTAYNNNNNNNTRTC	Pnuc_1939, Cytochrome-c oxidase	
m4C	2016549	-		Pnuc_1940, cytochrome c oxidase, subunit II	
m6A	2024520	+		upstream Pnuc_1959, protein of unknown function DUF526	
m4C	2026859	+		upstream Pnuc_1959, protein of unknown function DUF526	
m4C	2033529	+		upstream Pnuc_1967, biotin--acetyl-CoA-carboxylase ligase	
m4C	2041650	-		upstream Pnuc_1969, rfaE bifunctional protein	
m4C	2041656	+		Pnuc_1972, protein of unknown function DUF1365	
m4C	2047910	+		upstream Pnuc_1981, pseudo	
m4C	2050418	+		upstream Pnuc_1981, pseudo	
m6A	2054211	-	GAYANNNNNNNRTAG	upstream Pnuc_1984, Alcohol dehydrogenase, zinc-binding domain protein	
m4C	2055912	+		upstream Pnuc_1996, protein of unknown function DUF185	
m6A	2063777	+	CTAYNNNNNNNTRTC	upstream Pnuc_1996, protein of unknown function DUF185	
m6A	2063787	-	GAYANNNNNNNRTAG	Pnuc_1992, UDP-N-acetylglucosamine pyrophosphorylase / glucosamine-1-phosphate N-acetyltransferase	
m4C	2066268	+		upstream Pnuc_1996, protein of unknown function DUF185	
m6A	2066312	+	GAYANNNNNNNRTAG	upstream Pnuc_1996, protein of unknown function DUF185	
m6A	2066322	-	CTAYNNNNNNNTRTC	Pnuc_1994, dihydroneopterin aldolase	
m4C	2075843	+		upstream Pnuc_2004, lipid A biosynthesis acyltransferase	
m4C	2075980	-		Pnuc_2003, methionine adenosyltransferase	
m4C	2083827	+		upstream Pnuc_2021, rod shape-determining protein MreB	
m6A	2085972	+	GAYANNNNNNNRTAG	upstream Pnuc_2021, rod shape-determining protein MreB	
m6A	2085982	-	CTAYNNNNNNNTRTC	Pnuc_2014, transcriptional regulator, TraR/DksA family	
m6A	2089363	-		Pnuc_2018, aspartyl/glutamyl-tRNA(Asn/Gln) amidotransferase subunit B	
m4C	2092386	+		upstream Pnuc_2021, rod shape-determining protein MreB	
m4C	2093112	-		upstream Pnuc_2020, aspartyl/glutamyl-tRNA(Asn/Gln) amidotransferase subunit C	

m6A	2102026	+	CTAYNNNNNNNTRTC	Pnuc_2031, lipid A biosynthesis acyltransferase	
m6A	2102036	-	GAYANNNNNNNRTAG	upstream Pnuc_2029, protein of unknown function DUF167	
m6A	2104275	-		Pnuc_2034, sodium:dicarboxylate symporter	
m6A	2118474	+	GAYANNNNNNNRTAG	upstream Pnuc_2056, protein of unknown function DUF897	
m6A	2118484	-	CTAYNNNNNNNTRTC	Pnuc_2049, hypothetical protein	
m6A	2133954	-	CTAYNNNNNNNTRTC	upstream Pnuc_2064, hypothetical protein	
m6A	2146256	+	CTAYNNNNNNNTRTC	upstream Pnuc_2080, DNA protecting protein DprA	
m6A	2146266	-	GAYANNNNNNNRTAG	Pnuc_2077, sun protein	
m4C	2148177	+		upstream Pnuc_2080, DNA protecting protein DprA	
m4C	2150602	-		upstream Pnuc_2079, peptide deformylase	
m6A	2151830	-		upstream Pnuc_2079, peptide deformylase	
m6A	2158779	+		upstream Pnuc_0001, chromosomal replication initiator protein DnaA	

Supplementary Table S6. Enlistment of C^5-methyl-cytosine (m5C) and N^6-methyl-adenine (m6A) DNA modification sites in the genome of *P. asymbioticus* str. QLW-P1DMWA-1^T, as revealed by Single Molecule, Real-Time (SMRT) sequencing.

Supplementary Table S7. (a)

	Open Reading Frames		500-bp Upstream	
	UV m6A (%)	UV m4C (%)	UV m6A (%)	UV m4C (%)
Upregulated	12 (17)	8 (15)	0 (0)	2 (25)
Downregulated	59 (83)	44 (85)	16 (100)	6 (75)

Supplementary Table S7. (b)

	Open Reading Frames		500-bp Upstream	
	4ºC m6A (%)	4ºC m4C (%)	4ºC m6A (%)	4ºC m4C (%)
Upregulated	10 (11)	39 (19)	11 (29)	2 (25)
Downregulated	82 (89)	164 (81)	27 (71)	6 (75)

Supplementary Table S7. Table showing counts of adenine and cytosine methylation within differentially expressed genes (and percentage of expressed genes in parenthesis) of: **(a)**. UV plus 26ºC (26ºC*) treated sample and **(b)**. 4ºC incubated sample, within gene body and 500-bp upstream sequence.

Supplementary Table S8.

4ºC incubated sample versus 26ºC sample

Gene id	Microarray (fold expression)	Proteome (fold expression)	Methylome 4°C	Methylome 26°C	upstream methylation (4ºC)
Pnuc_0008	3.69	0.08	m6A		
Pnuc_1750	3.66	0.80	m4C		m4C (222bp)
Pnuc_2021	2.69	1.26	m4C		
Pnuc_1676	2.64	1.40	m6A		
Pnuc_1494	2.44	0.23		m4C	
Pnuc_1362	1.80	0.59	m6A	m6A	
Pnuc_0134	1.40	0.64		m4C	
Pnuc_1804	0.84	0.22	m6A		
Pnuc_1403	0.77	1.06		m4C	m4C (22bp)
Pnuc_0604	0.76	0.78	m6A		
Pnuc_1483	0.60	0.15	m6A		
Pnuc_0714	0.54	0.78		m4C	
Pnuc_0171	0.49	0.46	m6A		
Pnuc_0100	0.42	0.95		m4C	
Pnuc_1624	0.39	0.52		m4C	
Pnuc_1479	-0.28	-0.51			m4C (320bp)
Pnuc_1261	-0.29	-4.99	m6A		m6A (88bp)
Pnuc_1726	-0.30	-1.34	m4C		
Pnuc_0434	-0.31	-0.47	m6A	m6A	
Pnuc_0470	-0.34	-1.03	m6A	m6A	m6A (234bp)
Pnuc_0641	-0.36	-0.74	m6A	m6A	
Pnuc_1774	-0.36	-0.73	m6A		
Pnuc_0689	-0.38	-0.62			m6A (304bp)

Pnuc_0527	-0.39	-1.47			m6A (406bp)
Pnuc_1777	-0.39	-2.60	m6A	m6A	
Pnuc_0699	-0.40	-1.12			m4C (277bp)
Pnuc_1531	-0.41	-2.34	m6A	m6A	
Pnuc_1382	-0.42	-0.75			m6A (201bp)
Pnuc_0353	-0.42	-1.75	m6A	m6A	
Pnuc_0384	-0.44	-0.73			m6A (16bp)
Pnuc_0899	-0.44	-2.41	m4C, m6A		
Pnuc_1306	-0.47	-2.04		m4C	
Pnuc_0991	-0.49	-0.77		m4C	
Pnuc_1256	-0.50	-0.40		m6A	
Pnuc_0447	-0.51	-1.28	m6A, m6A	m4C, m6A, m6A, m4C, m6A	
Pnuc_1246	-0.51	-2.36	m6A	m6A, m6A	
Pnuc_2060	-0.52	-0.59	m6A, m4C	m6A	
Pnuc_1205	-0.53	-3.09	m6A, m4C, m4C	m6A	
Pnuc_0796	-0.54	-0.52		m4C	
Pnuc_0227	-0.55	-0.29		m4C	
Pnuc_0141	-0.55	-0.58		m4C	
Pnuc_1180	-0.56	-0.46	m6A, m6A	m6A, m6A	
Pnuc_0997	-0.56	-0.42			m4C (6bp)
Pnuc_1357	-0.56	-1.53		m6A	
Pnuc_1105	-0.57	-2.52		m6A	
Pnuc_0509	-0.58	-0.54	m4C		
Pnuc_1711	-0.58	-0.12		m4C	
Pnuc_0763	-0.59	-0.30		m6A, m4A	
Pnuc_0220	-0.59	-0.76		m4C, m4C	m4C (395bp)
Pnuc_0489	-0.59	-0.48	m4C		
Pnuc_1206	-0.59	-2.17		m6A	
Pnuc_1799	-0.59	-1.41	m4C		
Pnuc_1103	-0.60	-1.46	m6A		

Pnuc_0258	-0.60	-1.38		m4C, m4C	
Pnuc_0535	-0.61	-1.99		m4C	
Pnuc_0193	-0.64	-1.31		m6A	
Pnuc_0570	-0.64	-1.23	m6A	m6A	
Pnuc_0451	-0.65	-1.04	m6A		
Pnuc_1111	-0.65	-3.08	m6A, m6A	m6A, m6A, m4C	
Pnuc_1787	-0.69	-1.59	m6A, m4C	m6A	
Pnuc_1026	-0.71	-1.65	m6A, m6A	m6A	
Pnuc_1012	-0.75	-1.46		m4C	
Pnuc_1084	-0.75	-0.48	m6A		
Pnuc_1784	-0.81	-1.47	m6A	m6A	
Pnuc_1407	-0.81	-2.22	m4C	m4C	
Pnuc_1148	-0.83	-0.27			
Pnuc_0958	-0.83	-0.37	m6A, m6A	m6A	
Pnuc_1284	-0.84	-1.51		m4C	
Pnuc_2022	-0.85	-3.52	m4C, m4C		
Pnuc_0335	-0.87	-2.99		m4C	
Pnuc_0889	-0.87	-0.19		m6A, m4C	
Pnuc_1163	-0.88	-0.73		m4C	
Pnuc_1300	-0.91	-2.40	m4C, m4C		
Pnuc_0436	-0.92	-1.73		m4C, m4C	
Pnuc_0251	-0.95	-1.68	m4C	m4C	m6A (55bp)
Pnuc_0842	-1.13	-0.89	m6A	m6A	
Pnuc_0431	-1.27	-0.88		m6A	
Pnuc_1873	-1.28	-0.45		m4C	
Pnuc_0672	-1.30	-0.55		m4C	
Pnuc_1150	-1.40	-0.53	m6A	m6A	m4C (384bp)
Pnuc_0775	-1.42	-0.66		m4C	
Pnuc_0690	-1.47	-3.13	m6A	m4C	m4C (70bp)
Pnuc_0426	-1.53	-0.63	m6A		

Pnuc_0747	-1.64	-2.72	m6A	m6A	
Pnuc_1510	-1.68	-0.60			
Pnuc_0668	-1.69	-0.58		m4C	

UV treated sample comparison against 26ºC sample

Gene id	Microarray (fold expression)	Proteome (fold expression)	Methylome UV	Methylome 26°C	upstream methylation (UV)
Pnuc_1805	2.39	2.80	m6A	m4C	
Pnuc_1769	2.12	2.20		m4C	
Pnuc_2021	1.17	0.85			m4C (285bp)
Pnuc_1564	0.49	3.36	m6A	m6A	
Pnuc_1332	0.19	3.85	m6A	m6A	
Pnuc_1504	0.16	0.73		m4C	
Pnuc_1234	-0.20	-0.53		m6A	
Pnuc_0735	-0.23	-0.89	m4C		
Pnuc_0990	-0.33	-2.50			m4C (211bp), m6A (220bp)
Pnuc_0913	-0.34	-1.14	m6A	m6A	
Pnuc_0570	-0.36	-1.32	m6A	m6A	
Pnuc_0775	-0.40	-1.24		m4C	
Pnuc_0488	-0.47	-1.90	m4C		
Pnuc_0842	-0.51	-0.97	m6A	m6A	
Pnuc_0737	-0.52	-0.43			m6A (446bp)
Pnuc_0335	-0.53	-0.98		m4C	
Pnuc_1357	-0.55	-0.47		m6A	
Pnuc_1012	-0.57	-2.59		m4C	
Pnuc_0923	-0.62	-0.50	m4C	m6A	
Pnuc_0092	-0.70	-0.05		m6A	

Pnuc_0400	-0.82	-0.15		m4C
Pnuc_0958	-0.98	-0.30	m6A	m6A
Pnuc_0668	-1.06	-0.23		m4C
Pnuc_0227	-1.19	-0.06		m4C
Pnuc_1150	-1.22	-0.34	m6A	m6A
Pnuc_0526	-1.61	-0.39	m6A	m6A

Supplementary Table S8. Co-occurence of gene methylation, gene expression and corresponding protein detection.

Supplementary Table S9.

Bacteria	Genome (bp)	DNA methylation (all types detected)	Methylated genome (%)	References
Helicobacter pylori UM032	1593537	63299	3.97	Lee, W. C., Anton, B. P., Wang, S., Baybayan, P., Singh, S., Ashby, M., et al. (2015). The complete methylome of *Helicobacter pylori* UM032. *BMC Genomics*. 16,424.
Helicobacter pylori J99-R3	1643831	57345	3.49	Krebes, J., Morgan, R. D., Bunk, B., Spröer, C., Luong, K., Parusel, R., et al. (2013). The complex methylome of the human gastric pathogen *Helicobacter pylori*. *Nucleic Acids Res*. 42,1-18.
Helicobacter pylori 26695	1667867	50533	3.02	Krebes, J., Morgan, R. D., Bunk, B., Spröer, C., Luong, K., Parusel, R., et al. (2013). The complex methylome of the human gastric *pathogen Helicobacter pylori*. *Nucleic Acids Res*. 42,1-18.
Campylobacter jejuni NCTC11168	1641481	32863	2.00	Murray, I. A., Clark, T. A., Morgan, R. D., Boitano, M., Anton, B. P., Luong, K., et al. (2012). The methylomes of six bacteria. *Nucleic Acids Res*. 40,11450-11462.
Campylobacter jejuni 81-176	1699052	32023	1.88	Murray, I. A., Clark, T. A., Morgan, R. D., Boitano, M., Anton, B. P., Luong, K., et al. (2012). The methylomes of six bacteria. *Nucleic Acids Res*. 40,11450-11462.
Escherichia coli O25b:K100:H4-ST131 EC958	5249449	52081	0.99	Forde, B. M., Phan, M. D., Gawthorne, J. A., Ashcroft, M. M., Stanton-Cook, M., Sarkar, S., et al. (2015). Lineage-specific methyltransferases define the methylome of the globally disseminated *Escherichia coli* ST131 clone. *mBio*. 6:e01602-15.

Escherichia coli O104:H4 C227-11	5542971	51972	0.94	Fang, G., Munera, D., Friedman, D. I., Mandlik, A., Chao, M. C., Banerjee, O, et al. (2012). Genome-wide mapping of methylated adenine residues in pathogenic *Escherichia coli* using single-molecule real-time sequencing. *Nat. Biotechnol*. 30,1232-1239.
Mycoplasma pneumoniae M129	816394	6660	0.81	Lluch-Senar, M., Luong, K., Lloréns-Rico, V., Delgado, J., Fang, G., Spittle, K., et al. (2013). Comprehensive methylome *characterization of Mycoplasma genitalium* and *Mycoplasma pneumoniae* at single-base resolution. *PLoS Genet*. 9,e1003191.
Mycoplasma genitalium G-37	580076	4568	0.79	Lluch-Senar, M., Luong, K., Lloréns-Rico, V., Delgado, J., Fang, G., Spittle, K., et al. (2013). Comprehensive methylome *characterization of Mycoplasma genitalium* and *Mycoplasma pneumoniae* at single-base resolution. *PLoS Genet*. 9,e1003191.
Vibrio breoganii 1C-10	4116728	30020	0.72	Murray, I. A., Clark, T. A., Morgan, R. D., Boitano, M., Anton, B. P., Luong, K., et al. (2012). The methylomes of six bacteria. *Nucleic Acids Res*. 40,11450-11462.
Geobacter metallireducens GS-15	4011182	29166	0.72	Murray, I. A., Clark, T. A., Morgan, R. D., Boitano, M., Anton, B. P., Luong, K., et al. (2012). The methylomes of six bacteria. *Nucleic Acids Res*. 40,11450-11462.
Caulobacter crescentus	4016947	13471 - 25927	0.33 - 0.64	Kozdon, J. B., Melfi, M.D., Luong, K., Clark, T. A., Boitano, M., Wang, S, et al. (2013). Global methylation state at base-pair resolution *of the Caulobacter* genome throughout the cell cycle. *Proc. Natl. Acad. Sci. USA*. 110,E4658.
Bacillus cereus ATCC 10987	5432652	10583	0.19	Murray, I. A., Clark, T. A., Morgan, R. D., Boitano, M., Anton, B. P., Luong, K., et al. (2012). The methylomes of six bacteria. *Nucleic*

				Acids Res. 40,11450-11462.
Chromohalobacter salexigens	3696649	5541	0.15	Murray, I. A., Clark, T. A., Morgan, R. D., Boitano, M., Anton, B. P., Luong, K., et al. (2012). The methylomes of six bacteria. *Nucleic Acids Res*. 40,11450-11462.
Polynucleobacter asymbioticus QLW-P1DMWA-1^T	2159490	873 - 1504	0.04 - 0.07	This study.

Supplementary Table S9. Methylation percentage in selected bacterial genome.

Supplementary Table S10.

Selected genes in Beta proteobacterium CB

Sequence	Method	Ka	Ks	Ka/Ks	P-Value(Fisher)	Length	S-Sites	N-Sites	Substitutions	S-Substitutions	N-Substitutions
Alkyl	MS	0.0490	2.3656	0.0207	4.64E-65	420	99.9824	320.018	89	83.4638	5.53621
Alkyl	GMYN	0.0526	2.1569	0.0244	3.92E-41	420	103.032	316.968	89	72.9183	16.0817
Carbo	MS	0.0513	4.1831	0.0123	6.84E-123	615	141.208	473.792	141	135.426	5.57402
Carbo	GMYN	0.0554	3.0948	0.0179	4.18E-65	615	158.128	456.872	141	116.682	24.3185
Cold	MS	0.0000	0.1975	0.0000	0	156	45.9512	110.049	7	6.99998	1.68E-05
Cold	GMYN	0.0000	0.2277	0.0000	0	156	38.2496	117.75	7	7	0
DnaK	MS	0.0142	0.8334	0.0170	3.87E-139	1878	517.35	1360.65	248	237.374	10.626
DnaK	GMYN	0.0133	1.5135	0.0088	5.94E-149	1878	423.783	1454.22	248	228.79	19.2105
DSBA	MS	0.2138	3.7619	0.0568	NA	594	131.622	462.378	197	164.216	32.7843
DSBA	GMYN	0.2140	3.6227	0.0591	3.91E-49	594	125.244	468.756	197	110.641	86.3591
Fatty	MS	0.0441	1.2511	0.0353	7.41E-116	1143	298.721	844.279	212	192.785	19.2146
Fatty	GMYN	0.0439	1.2500	0.0352	4.39E-91	1143	288.675	854.325	212	175.616	36.3844
Fimb	MS	0.0537	3.7249	0.0144	3.32E-86	465	119.856	345.144	112	107.533	4.46737
Fimb	GMYN	0.0545	3.5996	0.0151	4.43E-54	465	121.442	343.558	112	93.9862	18.0138
FtsK	MS	0.0298	1.2058	0.0247	5.23E-226	2256	594.393	1661.61	392	366.665	25.3354
FtsK	GMYN	0.0298	2.2884	0.0130	1.48E-191	2256	569.943	1686.06	392	342.76	49.2405
GroL	MS	NA	NA	NA	NA	NA	NA	NA	NA	NA	NA
GroL	GMYN	0.0239	1.3477	0.0177	5.70E-89	1596	357.271	1238.73	191	161.964	29.0361
Hfq	MS	0.0505	1.1646	0.0434	6.17E-15	195	67.7019	127.298	33	30.511	2.48904
Hfq	GMYN	0.0442	1.1378	0.0388	1.42E-13	195	51.3775	143.623	33	26.9232	6.07677
Hyp	MS	0.0429	1.5402	0.0279	2.74E-22	219	69.9217	149.078	42	39.6458	2.35418
Hyp	GMYN	0.0398	1.0712	0.0371	4.83E-18	219	63.1353	155.865	42	36	6
MscS	MS	0.1362	3.6868	0.0369	NA	768	186.848	581.152	228	204.509	23.4909
MscS	GMYN	0.1339	3.9083	0.0343	1.80E-79	768	183.276	584.724	228	157.031	70.9689
Piri	MS	0.1708	3.6202	0.0472	NA	684	164.432	519.568	208	181.022	26.9781
Piri	GMYN	0.1563	3.7993	0.0411	3.33E-63	684	158.501	525.499	208	134.636	73.3645
RpoE	MS	0.0421	0.8878	0.0475	9.16E-42	522	130.159	391.841	83	72.6232	10.3768
RpoE	GMYN	0.0403	1.5532	0.0260	3.76E-34	522	129.844	392.156	83	67.632	15.368
RpoH	MS	0.0576	1.8488	0.0311	5.55E-108	888	261.831	626.169	196	182.414	13.586

RpoH	GMYN	0.0530	3.3685	0.0157	1.12E-92	888	218.22	669.78	196	161.831	34.1686
Rubre	MS	0.0714	3.2578	0.0219	7.09E-66	438	127.645	310.355	105	99.6893	5.3107
Rubre	GMYN	0.0676	2.1995	0.0307	6.65E-43	438	118.953	319.047	105	84.4634	20.5366
RuvB	MS	0.0216	1.7691	0.0122	7.10E-134	1014	262.888	751.112	195	188.427	6.57318
RuvB	GMYN	0.0207	4.0690	0.0051	3.83E-132	1014	227.079	786.921	195	178.926	16.0736
			**maximum**	0.0591							
			**minimum**	0.0000							
			**average**	0.0263							
			**median**	0.0247							

Housekeeping genes in Beta proteobacterium CB

Sequence	Method	Ka	Ks	Ka/Ks	P-Value(Fisher)	Length	S-Sites	N-Sites	Substitutions	S-Substitutions	N-Substitutions
iso	MS	0.0367	1.0320	0.0355	7.66E-192	2220	555.089	1664.91	358	323.532	34.4682
iso	GMYN	0.0364	2.4546	0.0148	4.93E-164	2220	511.193	1708.81	358	297.458	60.5418
acsA	MS	0.0191	0.7978	0.0240	7.18E-143	1959	537.515	1421.49	267	251.083	15.9166
acsA	GMYN	0.0182	1.1909	0.0153	7.15E-145	1959	455.441	1503.56	267	239.983	27.0171
succ	MS	0.0213	1.1644	0.0183	1.66E-166	1764	411.916	1352.08	259	244.355	14.6446
succ	GMYN	0.0204	1.6253	0.0126	2.26E-165	1764	347.102	1416.9	259	230.516	28.4839
CTP	MS	0.0431	2.5545	0.0169	4.83E-258	1647	388.169	1258.83	348	329.961	18.0385
CTP	GMYN	0.0446	4.4242	0.0101	1.94E-193	1647	364.641	1282.36	348	292.589	55.4106
atpA	MS	0.0058	0.5249	0.0110	3.44E-87	1527	408.415	1118.59	150	145.618	4.3816
atpA	GMYN	0.0054	0.5244	0.0103	9.82E-82	1527	415.773	1111.23	150	143.998	6.00155
gyr	MS	0.0421	2.8067	0.0150	1.76E-219	1422	370.516	1051.48	312	299.273	12.7274
gyr	GMYN	0.0433	2.0001	0.0216	9.49E-147	1422	384.668	1037.33	312	268.459	43.5413
glnA	MS	0.0262	0.6366	0.0412	7.71E-80	1401	327.913	1073.09	159	140.12	18.8796
glnA	GMYN	0.0257	1.3264	0.0193	8.04E-75	1401	284.568	1116.43	159	130.875	28.1248
cit	MS	0.0248	1.0818	0.0229	1.70E-120	1299	314.94	984.06	199	185.712	13.2882
cit	GMYN	0.0235	1.7439	0.0135	6.66E-123	1299	258.548	1040.45	199	174.954	24.0462
adk	MS	0.0394	0.8747	0.0450	1.55E-47	651	167.084	483.916	95	84.0386	10.9614
adk	GMYN	0.0356	1.0140	0.0351	3.30E-39	651	158.644	492.356	95	77.933	17.067
ndk	MS	0.0349	0.6510	0.0537	7.66E-23	411	78.9238	332.076	43	35.0774	7.92264
ndk	GMYN	0.0382	0.4817	0.0794	1.12E-13	411	98.3222	312.678	43	31.3974	11.6026
			**maximum**	0.0794							

**minimum**	0.0101
**average**	0.0258
**median**	0.0188

Selected genes in FNE-F8 bin 6 1 PnecC

Sequence	Method	Ka	Ks	Ka/Ks	P-Value(Fisher)	Length	S-Sites	N-Sites	Substitutions	S-Substitutions	N-Substitutions
FtsK	MS	0.0252	1.5749	0.0160	1.24E-259	2205	596.985	1608.01	416	398.838	17.1616
FtsK	GMYN	0.0248	1.3630	0.0182	7.24E-214	2205	599.942	1605.06	416	376.857	39.1426
DnaK	MS	0.0185	1.0103	0.0183	4.45E-141	1830	550.86	1279.14	267	256.134	10.8657
DnaK	GMYN	0.0168	1.9225	0.0087	2.32E-155	1830	423.986	1406.01	267	243.718	23.2819
GroL	MS	0.0241	0.8124	0.0296	2.24E-102	1551	417.608	1133.39	199	184.183	14.817
GroL	GMYN	0.0239	0.7832	0.0305	2.93E-85	1551	401.001	1150	199	171.99	27.0099
Fatty	MS	0.0511	1.6921	0.0302	2.84E-146	1101	260.493	840.507	227	206.861	20.1391
Fatty	GMYN	0.0510	2.4188	0.0211	1.44E-107	1101	257.707	843.293	227	185.597	41.4029
RuvB	MS	0.0248	2.0379	0.0122	7.82E-130	978	256.851	721.149	191	184.694	6.30582
RuvB	GMYN	0.0213	1.8020	0.0118	1.39E-109	978	257.85	720.15	191	175.891	15.1094
RpoH	MS	0.0443	2.2379	0.0198	1.43E-101	849	248.834	600.166	172	164.159	7.84083
RpoH	GMYN	0.0440	1.7936	0.0245	4.01E-81	849	212.649	636.351	172	144.894	27.1059
Piri	MS	0.1562	3.5478	0.0440	NA	615	153.004	461.996	188	165.936	22.0642
Piri	GMYN	0.1554	3.6912	0.0421	7.69E-62	615	137.219	477.781	188	121.503	66.4971
Carbo	MS	0.0490	2.3829	0.0206	7.60E-86	579	137.615	441.385	120	112.579	7.42133
Carbo	GMYN	0.0530	1.5878	0.0334	2.63E-52	579	148.626	430.374	120	98.0499	21.9501
DSBA	MS	0.2287	3.5739	0.0640	NA	543	125.194	417.806	197	162.327	34.6731
DSBA	GMYN	0.2308	3.5685	0.0647	4.05E-59	543	116.507	426.493	197	113.199	83.8006
RpoE	MS	0.0373	0.9192	0.0406	6.41E-39	489	120.406	368.594	74	65.8281	8.1719
RpoE	GMYN	0.0408	0.6439	0.0634	2.68E-22	489	157.596	331.404	74	60.869	13.131
Rubre	MS	0.0796	3.1231	0.0255	1.44E-52	405	122.483	282.517	92	86.8934	5.1066
Rubre	GMYN	0.0745	1.6575	0.0449	1.76E-33	405	106.147	298.853	92	70.8764	21.1236
Alkyl	MS	0.0477	2.8312	0.0169	1.18E-67	387	80.7862	306.214	79	74.2535	4.74646
Alkyl	GMYN	0.0556	1.5720	0.0353	1.44E-30	387	105.061	281.939	79	63.94	15.06
Hyp	MS	0.1081	1.7632	0.0613	3.83E-30	258	63.4016	194.598	56	47.1288	8.87121
Hyp	GMYN	0.1130	1.3727	0.0823	6.17E-13	258	67.9994	190.001	56	36.1982	19.8018

Hfq	MS	0.0162	4.1595	0.0039	0	165	39.1784	125.822	35	34.568	0.431958
Hfq	GMYN	0.0166	2.8099	0.0059	7.18E-24	165	42.3758	122.624	35	33	2
Cold	MS	0.0587	0.3859	0.1520	0.000282898	126	33.8772	92.1228	14	9.90516	4.09484
Cold	GMYN	0.0555	0.3986	0.1392	0.000979928	126	31.0745	94.9255	14	9	5
			**maximum**	0.1520							
			**minimum**	0.0039							
			**average**	0.0394							
			**median**	0.0299							

Housekeeping genes in FNE-F8 bin 6 1 PnecC

Sequence	Method	Ka	Ks	Ka/Ks	P-Value(Fisher)	Length	S-Sites	N-Sites	Substitutions	S-Substitutions	N-Substitutions
iso	MS	0.0377	1.1919	0.0317	4.93E-205	2229	613.557	1615.44	390	359.982	30.0182
iso	GMYN	0.0367	1.1967	0.0306	2.46E-178	2229	552.028	1676.97	390	330.066	59.9338
acsA	MS	0.0451	0.9224	0.0489	1.22E-147	1968	505.133	1462.87	312	273.294	38.7059
acsA	GMYN	0.0450	1.1273	0.0399	2.22E-115	1968	483.851	1484.15	312	247.275	64.7248
succ	MS	0.0244	1.4442	0.0169	1.68E-197	1773	415.09	1357.91	294	278.629	15.3715
succ	GMYN	0.0231	4.3822	0.0053	2.19E-198	1773	344.8	1428.2	294	261.573	32.4274
CTP	MS	0.0445	2.0753	0.0215	2.13E-220	1656	426.505	1229.5	340	320.192	19.8082
CTP	GMYN	0.0430	4.4527	0.0097	6.11E-180	1656	378.766	1277.23	340	286.693	53.3066
atpA	MS	0.0109	0.5337	0.0205	3.59E-84	1536	431.827	1104.17	163	154.883	8.11725
atpA	GMYN	0.0103	0.8107	0.0128	2.82E-90	1536	366.785	1169.21	163	150.998	12.0025
gyr	MS	0.0394	2.9300	0.0135	8.63E-225	1431	361.43	1069.57	308	296.203	11.797
gyr	GMYN	0.0392	4.3704	0.0090	5.64E-173	1431	339.397	1091.6	308	266.397	41.6028
glnA	MS	0.0212	0.5916	0.0358	1.08E-79	1413	366.88	1046.12	161	146.086	14.9141
glnA	GMYN	0.0212	0.6623	0.0321	1.63E-69	1413	355.65	1057.35	161	138.89	22.1102
cit	MS	0.0226	1.3813	0.0163	2.13E-148	1308	317.063	990.937	222	211.209	10.7907
cit	GMYN	0.0227	1.4395	0.0158	6.41E-126	1308	312.118	995.882	222	199.746	22.2536
adk	MS	0.0582	1.3849	0.0421	6.22E-68	660	172.253	487.747	127	113.485	13.5149
adk	GMYN	0.0551	1.8739	0.0294	5.14E-57	660	144.849	515.151	127	99.7069	27.2931
ndk	MS	0.0321	0.7148	0.0448	1.13E-25	420	101.298	318.702	51	44.6942	6.30577
ndk	GMYN	0.0330	0.6499	0.0508	1.03E-19	420	102.274	317.726	51	40.795	10.205
			**maximum**	0.0508							
			**minimum**	0.0053							

**average**	0.0264
**median**	0.0254

Selected genes in MWH-Adler-W8

Sequence	Method	Ka	Ks	Ka/Ks	P-Value(Fisher)	Length	S-Sites	N-Sites	Substitutions	S-Substitutions	N-Substitutions
FtsK	MS	0.0134	0.9306	0.0144	1.17E-188	2232	655.371	1576.63	339	327.62	11.3798
FtsK	GMYN	0.0133	1.0223	0.0130	1.03E-174	2232	635.419	1596.58	339	317.958	21.042
DnaK	MS	0.0084	0.5584	0.0150	5.98E-105	1857	534.311	1322.69	195	188.012	6.98776
DnaK	GMYN	0.0079	0.7183	0.0110	7.00E-114	1857	443.221	1413.78	195	183.936	11.0639
GroL	MS	0.0038	0.4759	0.0080	2.62E-82	1575	433.088	1141.91	140	137.114	2.88573
GroL	GMYN	0.0033	0.7345	0.0045	3.09E-89	1575	369.8	1205.2	140	136	4.00009
Fatty	MS	0.0600	1.7005	0.0353	1.43E-146	1125	255.687	869.313	230	205.347	24.6531
Fatty	GMYN	0.0594	4.1670	0.0143	1.01E-106	1125	240.517	884.483	230	179.691	50.3093
RuvB	MS	0.0154	1.5575	0.0099	1.30E-124	996	268.221	727.779	185	180.16	4.84025
RuvB	GMYN	0.0154	1.4935	0.0103	1.32E-108	996	268.642	727.358	185	173.976	11.0241
RpoH	MS	0.0165	1.4136	0.0117	2.64E-88	855	237.894	617.106	143	138.795	4.20463
RpoH	GMYN	0.0158	1.3085	0.0121	1.04E-84	855	214.857	640.143	143	132.991	10.0092
Piri	MS	0.1356	3.3205	0.0408	NA	633	135.082	497.918	171	148.624	22.3756
Piri	GMYN	0.1362	3.6944	0.0369	1.26E-51	633	137.803	495.197	171	109.728	61.2724
Carbo	MS	0.0362	2.6543	0.0137	3.55E-88	603	141.123	461.877	116	111.038	4.9616
Carbo	GMYN	0.0387	1.9678	0.0197	2.17E-60	603	141.779	461.221	116	98.6223	17.3777
DSBA	MS	0.1338	3.8000	0.0352	NA	564	133.261	430.739	155	139.167	15.8333
DSBA	GMYN	0.1354	3.6545	0.0370	7.52E-46	564	130.663	433.337	155	101.764	53.2363
RpoE	MS	0.0153	0.8914	0.0172	4.23E-44	507	115.697	391.303	66	62.3697	3.63032
RpoE	GMYN	0.0148	2.0241	0.0073	7.32E-45	507	97.0133	409.987	66	59.9993	6.00071
Superox	MS	0.0731	1.6862	0.0433	9.78E-61	471	104.752	366.248	99	85.9714	13.0286
Superox	GMYN	0.0771	1.6481	0.0468	8.99E-32	471	123.061	347.939	99	73.6828	25.3172
Fimb	MS	0.0323	3.8914	0.0083	5.03E-88	465	116.601	348.399	107	104.409	2.5913
Fimb	GMYN	0.0335	3.5954	0.0093	1.69E-62	465	120.768	344.232	107	95.9016	11.0984
Rubre	MS	0.0245	2.5196	0.0097	5.37E-53	483	146.595	336.405	86	84.1211	1.87894
Rubre	GMYN	0.0244	1.1113	0.0219	1.15E-38	483	148.491	334.509	86	77.9986	8.00139
Alkyl	MS	0.0366	1.6574	0.0221	7.85E-52	405	96.7634	308.237	75	70.0759	4.92413

Alkyl	GMYN	0.0349	1.2734	0.0274	4.78E-36	405	105.767	299.233	75	64.8017	10.1983
Hyp	MS	0.0425	1.8575	0.0229	1.78E-19	204	53.2076	150.792	30	28.1741	1.82585
Hyp	GMYN	0.0384	1.4289	0.0269	1.25E-14	204	42.1986	161.801	30	24	6
Hfq	MS	0.0000	0.9622	0.0000	0	189	54.6278	134.372	24	23.9999	5.90E-05
Hfq	GMYN	0.0000	1.0078	0.0000	0	189	43.2947	145.705	24	24	0
Cold	MS	0.0000	0.0619	0.0000	0	141	32.2973	108.703	2	1.99999	6.73E-06
Cold	GMYN	0.0000	0.0629	0.0000	0	141	35.7646	105.235	2	2	0
			**maximum**	0.0468							
			**minimum**	0.0000							
			**average**	0.0178							
			**median**	0.0140							

Housekeeping genes in MWH-Adler-W8

Sequence	Method	Ka	Ks	Ka/Ks	P-Value(Fisher)	Length	S-Sites	N-Sites	Substitutions	S-Substitutions	N-Substitutions
iso	MS	0.0241	0.9105	0.0265	6.78E-177	2232	583.67	1648.33	324	301.438	22.5624
iso	GMYN	0.0245	1.4186	0.0172	2.73E-144	2232	595.329	1636.67	324	284.646	39.3536
acsA	MS	0.0271	0.7413	0.0365	1.82E-132	1971	537.997	1433	274	249.709	24.2911
acsA	GMYN	0.0253	1.5192	0.0167	9.39E-140	1971	433.419	1537.58	274	235.81	38.1899
succ	MS	0.0121	0.7966	0.0152	2.38E-123	1776	527.008	1248.99	232	223.912	8.08822
succ	GMYN	0.0113	1.4411	0.0078	5.91E-136	1776	427.859	1348.14	232	216.947	15.0531
CTP	MS	0.0441	1.6630	0.0265	5.29E-200	1659	406.643	1252.36	318	294.014	23.9861
CTP	GMYN	0.0409	4.3859	0.0093	3.89E-177	1659	346.475	1312.52	318	265.895	52.1048
atpA	MS	0.0027	0.2263	0.0120	4.25E-44	1539	438.488	1100.51	83	80.5698	2.43025
atpA	GMYN	0.0026	0.2655	0.0098	1.55E-49	1539	381.204	1157.8	83	80	3
pyr	MS	0.0363	3.6677	0.0099	5.11E-210	1434	380.539	1053.46	297	289.072	7.92841
pyr	GMYN	0.0365	2.0799	0.0175	1.84E-152	1434	368.689	1065.31	297	259.245	37.7552
glnA	MS	0.0079	0.3491	0.0225	2.74E-54	1413	404.929	1008.07	109	103.207	5.79339
glnA	GMYN	0.0077	0.4163	0.0184	6.23E-55	1413	360.822	1052.18	109	100.997	8.00264
cit	MS	0.0071	0.6569	0.0108	2.40E-89	1311	330.451	980.549	144	139.534	4.46591
cit	GMYN	0.0072	0.6880	0.0105	2.31E-82	1311	333.787	977.213	144	136.999	7.00117
adk	MS	0.0250	0.7580	0.0330	1.77E-41	663	208.577	454.423	91	84.9041	6.09593
adk	GMYN	0.0230	0.9005	0.0256	2.82E-41	663	175.583	487.417	91	79.9576	11.0424
ndk	MS	0.0030	0.4310	0.0070	0	423	98.4718	324.528	29	28.3486	0.651395

ndk	GMYN	0.0031	0.3955	0.0078	7.47E-19	423	96.2147	326.785	29	28	1
			**maximum**	0.0365							
			**minimum**	0.0070							
			**average**	0.0170							
			**median**	0.0159							

Selected genes in MWH-HuW1

Sequence	Method	Ka	Ks	Ka/Ks	P-Value(Fisher)	Length	S-Sites	N-Sites	Substitutions	S-Substitutions	N-Substitutions
Alkyl	MS	0.0578	2.0329	0.0285	1.81E-60	432	103.051	328.949	90	82.5059	7.49409
Alkyl	GMYN	0.0616	1.7443	0.0353	1.49E-37	432	104.463	327.537	90	70.6916	19.3084
Carbo	MS	0.0658	4.1225	0.0160	NA	627	144.387	482.613	154	146.196	7.80388
Carbo	GMYN	0.0653	3.6205	0.0180	5.11E-98	627	124.875	502.125	154	122.929	31.0711
Cold	MS	0.0000	0.1302	0.0000	0	171	38.4118	132.588	5	4.99998	1.73E-05
Cold	GMYN	0.0000	0.1473	0.0000	0	171	38.8647	132.135	5	5	0
DnaK	MS	0.0176	1.0387	0.0169	4.08E-156	1890	548.263	1341.74	284	272.71	11.2896
DnaK	GMYN	0.0161	1.2408	0.0130	1.65E-172	1890	429.855	1460.14	284	260.749	23.2506
DSBA	MS	0.2284	3.7082	0.0616	NA	600	133.047	466.953	217	178.435	38.5649
DSBA	GMYN	0.2311	3.6198	0.0638	1.92E-67	600	124.76	475.24	217	123.477	93.5227
Fatty	MS	0.0488	1.7769	0.0275	1.86E-158	1155	283.951	871.049	243	224.103	18.8968
Fatty	GMYN	0.0500	1.7359	0.0288	1.15E-106	1155	298.506	856.494	243	201.757	41.2426
Fimb	MS	0.0922	3.6166	0.0255	NA	489	125.954	363.046	143	133.21	9.78959
Fimb	GMYN	0.0955	3.6445	0.0262	2.71E-60	489	128.943	360.057	143	110.897	32.1029
FtsK	MS	0.0308	1.6376	0.0188	7.34E-283	2265	600.328	1664.67	448	425.805	22.1948
FtsK	GMYN	0.0317	1.7850	0.0178	4.45E-232	2265	581.485	1683.51	448	395.749	52.2514
GroL	MS	0.0255	0.8289	0.0308	9.77E-113	1608	433.234	1174.77	219	202.11	16.8905
GroL	GMYN	0.0237	2.8105	0.0084	2.00E-110	1608	363.96	1244.04	219	189.962	29.0379
Hfq	MS	0.0500	1.4481	0.0345	2.03E-22	210	60.6699	149.33	41	37.7899	3.21014
Hfq	GMYN	0.0478	1.2818	0.0373	5.32E-18	210	55.2251	154.775	41	33.9205	7.0795
Hyp	MS	0.0839	3.2142	0.0261	2.06E-35	237	69.3616	167.638	57	53.6181	3.38187
Hyp	GMYN	0.0874	1.9998	0.0437	1.49E-16	237	74.8291	162.171	57	43.7518	13.2482
MscS	MS	0.1593	3.6796	0.0433	NA	783	186.984	596.016	252	221.435	30.5654
MscS	GMYN	0.1594	3.9277	0.0406	3.07E-82	783	188.102	594.898	252	167.729	84.271

Piri	MS	0.2023	3.8314	0.0528	NA	663	145.735	517.265	212	178.54	33.4597
Piri	GMYN	0.2025	3.7300	0.0543	1.38E-49	663	144.516	518.484	212	120.756	91.2439
RpoE	MS	0.0271	1.1903	0.0227	2.71E-52	534	117.046	416.954	78	72.1561	5.84387
RpoE	GMYN	0.0268	1.8250	0.0147	8.89E-43	534	112.498	421.502	78	66.9366	11.0634
RpoH	MS	0.0579	2.0835	0.0278	2.69E-122	897	255.006	641.994	206	192.529	13.4715
RpoH	GMYN	0.0556	2.5579	0.0217	4.37E-92	897	235.377	661.623	206	170.703	35.2973
			**maximum**	0.0638							
			**minimum**	0.0000							
			**average**	0.0285							
			**median**	0.0268							

Housekeeping genes in MWH-HuW1

Sequence	Method	Ka	Ks	Ka/Ks	P-Value(Fisher)	Length	S-Sites	N-Sites	Substitutions	S-Substitutions	N-Substitutions
iso	MS	0.0307	1.1857	0.0259	7.20E-202	2232	621.318	1610.68	373	349.512	23.4885
iso	GMYN	0.0300	1.1779	0.0255	2.24E-183	2232	544.351	1687.65	373	323.368	49.6319
acsA	MS	0.0190	0.9509	0.0199	6.22E-165	1971	534.467	1436.53	291	276.202	14.7979
acsA	GMYN	0.0179	2.7781	0.0065	3.70E-171	1971	443.175	1527.82	291	263.979	27.0206
succ	MS	0.0244	1.1871	0.0206	1.84E-169	1776	457.36	1318.64	283	267.154	15.8464
succ	GMYN	0.0232	2.2408	0.0104	6.18E-182	1776	351.706	1424.29	283	250.484	32.5163
CTP	MS	0.0469	2.3755	0.0197	1.85E-244	1659	395.744	1263.26	345	324.552	20.4483
CTP	GMYN	0.0468	4.4215	0.0106	5.03E-186	1659	363.337	1295.66	345	286.354	58.6459
atpA	MS	0.0049	0.5582	0.0088	3.11E-94	1539	415.525	1123.47	158	154.339	3.66115
atpA	GMYN	0.0044	0.7333	0.0060	2.83E-94	1539	394.052	1144.95	158	152.999	5.00095
gyr	MS	0.0425	3.6752	0.0116	7.32E-232	1434	365.786	1068.21	314	303.75	10.2497
gyr	GMYN	0.0438	2.7416	0.0160	4.14E-155	1434	367.735	1066.27	314	268.672	45.3283
glnA	MS	0.0260	0.7578	0.0343	2.59E-97	1413	365.432	1047.57	189	172.082	16.9183
glnA	GMYN	0.0252	1.1563	0.0218	5.25E-93	1413	316.056	1096.94	189	161.881	27.1187
cit	MS	0.0187	0.8896	0.0210	6.26E-103	1311	344.629	966.371	182	171.865	10.1346
cit	GMYN	0.0172	1.5183	0.0113	1.66E-120	1311	251.927	1059.07	182	163.991	18.0088
adk	MS	NA	NA	NA	NA	NA	NA	NA	NA	NA	NA
adk	GMYN	0.0336	1.3159	0.0255	6.14E-54	663	141.762	521.238	105	87.9287	17.0713
ndk	MS	0.0121	0.5806	0.0209	6.22E-24	423	99.3507	323.649	39	36.5139	2.48615
ndk	GMYN	0.0125	0.5149	0.0243	1.76E-20	423	99.3462	323.654	39	35	4

**maximum**	0.0343
**minimum**	0.0060
**average**	0.0179
**median**	0.0199

Selected genes in MWH-JaK3

Sequence	Method	Ka	Ks	Ka/Ks	P-Value(Fisher)	Length	S-Sites	N-Sites	Substitutions	S-Substitutions	N-Substitutions
Alkyl	MS	0.0498	2.0586	0.0242	1.22E-50	444	125.475	318.525	85	80.0813	4.91868
Alkyl	GMYN	0.0518	1.9869	0.0261	8.43E-33	444	119.039	324.961	85	68.7605	16.2395
Carbo	MS	0.0711	2.6137	0.0272	2.92E-105	639	145.466	493.534	144	131.831	12.1692
Carbo	GMYN	0.0644	3.7198	0.0173	2.17E-69	639	142.548	496.452	144	113.49	30.5099
Cold	MS	0.0000	0.0485	0.0000	0	180	41.2123	138.788	2	1.99999	6.74E-06
Cold	GMYN	NA	0.0506	NA	0	180	43.1023	136.898	2	2	0
DnaK	MS	0.0096	0.5981	0.0160	2.37E-111	1896	497.188	1398.81	197	188.51	8.48977
DnaK	GMYN	0.0091	0.7509	0.0121	2.72E-114	1896	439.163	1456.84	197	183.896	13.1045
DSBA	MS	0.1303	3.6993	0.0352	NA	600	146.215	453.785	163	146.94	16.0595
DSBA	GMYN	0.1316	3.7388	0.0352	2.41E-46	600	145.769	454.231	163	108.579	54.4209
Fatty	MS	0.0671	1.9638	0.0342	9.31E-170	1161	271.284	889.716	257	231.106	25.8945
Fatty	GMYN	0.0672	4.1854	0.0161	7.91E-112	1161	265.218	895.782	257	199.624	57.3765
Fimb	MS	0.0581	3.1576	0.0184	5.75E-82	504	129.606	374.394	114	108.243	5.75668
Fimb	GMYN	0.0580	3.6129	0.0160	3.61E-53	504	123.612	380.388	114	92.8365	21.1635
FtsK	MS	NA	NA	NA	NA	NA	NA	NA	NA	NA	NA
FtsK	GMYN	0.0184	0.8855	0.0208	1.53E-158	2271	671.97	1599.03	347	317.888	29.1116
GroL	MS	0.0043	0.4033	0.0107	1.37E-72	1614	461.508	1152.49	133	129.544	3.45647
GroL	GMYN	0.0041	0.5418	0.0075	1.55E-81	1614	378.782	1235.22	133	128	5.00025
Hfq	MS	0.0067	0.9608	0.0070	0	219	65.6215	153.379	32	31.4868	0.51322
Hfq	GMYN	0.0068	0.8991	0.0075	4.58E-17	219	70.2969	148.703	32	31	1
Hyp	MS	0.0620	1.2128	0.0511	5.61E-22	243	68.3067	174.693	43	38.0284	4.97162
Hyp	GMYN	0.0627	0.9219	0.0680	5.24E-13	243	70.2652	172.735	43	32.7181	10.2819
MscS	MS	0.1391	2.8398	0.0490	5.40E-147	789	183.979	605.021	212	182.586	29.4139
MscS	GMYN	0.1338	3.8727	0.0346	7.12E-64	789	174.787	614.213	212	137.427	74.5728
Piri	MS	0.1090	2.1400	0.0509	4.45E-100	669	150.938	518.062	160	136.19	23.8101

Piri	GMYN	0.1105	3.7016	0.0298	1.67E-52	669	139.138	529.862	160	105.835	54.1649
RpoE	MS	0.0243	0.9293	0.0262	2.06E-46	543	126.226	416.774	77	70.8697	6.13029
RpoE	GMYN	0.0224	1.1076	0.0202	2.31E-39	543	131.462	411.538	77	67.9305	9.06949
RpoH	MS	0.0176	1.1343	0.0155	6.01E-84	891	253.7	637.3	144	138.604	5.39645
RpoH	GMYN	0.0168	1.8747	0.0090	1.14E-81	891	226.384	664.616	144	132.978	11.0217
RuvB	MS	0.0195	1.5113	0.0129	1.17E-122	1032	274.545	757.455	188	181.547	6.45305
RuvB	GMYN	0.0176	1.3142	0.0134	2.34E-104	1032	282.23	749.77	188	174.974	13.0262
			**maximum**	0.0680							
			**minimum**	0.0000							
			**average**	0.0237							
			**median**	0.0193							

Housekeeping genes in MWH-JaK3

Sequence	Method	Ka	Ks	Ka/Ks	P-Value(Fisher)	Length	S-Sites	N-Sites	Substitutions	S-Substitutions	N-Substitutions
iso	MS	0.0161	0.8209	0.0196	1.85E-170	2232	595.338	1636.66	303	287.544	15.4558
iso	GMYN	0.0154	1.8312	0.0084	3.51E-174	2232	513.728	1718.27	303	276.868	26.1316
acsA	MS	0.0259	0.7986	0.0324	1.53E-138	1971	559.195	1411.81	283	261.597	21.4034
acsA	GMYN	0.0240	1.8174	0.0132	1.06E-149	1971	437.394	1533.61	283	246.884	36.1162
succ	MS	0.0176	0.8302	0.0212	4.27E-135	1776	438.421	1337.58	230	216.015	13.9846
succ	GMYN	0.0161	1.7823	0.0090	7.29E-132	1776	393.031	1382.97	230	207.96	22.0404
CTP	MS	0.0383	1.4202	0.0270	1.53E-182	1659	419.317	1239.68	305	282.456	22.5437
CTP	GMYN	0.0379	1.4861	0.0255	3.88E-147	1659	399.829	1259.17	305	258.527	46.4732
atpA	MS	0.0019	0.1442	0.0129	2.04E-30	1539	464.41	1074.59	59	57.2907	1.70933
atpA	GMYN	0.0018	0.1646	0.0107	2.22E-32	1539	400.875	1138.13	59	57	2
gyr	MS	0.0394	3.4594	0.0114	1.43E-214	1434	402.787	1031.21	319	309.964	9.03628
gyr	GMYN	0.0402	2.2461	0.0179	1.53E-156	1434	390.631	1043.37	319	278.312	40.6879
glnA	MS	0.0104	0.4212	0.0248	1.15E-62	1413	361.456	1051.54	118	110.068	7.93202
glnA	GMYN	0.0108	0.3915	0.0275	5.94E-53	1413	382.426	1030.57	118	106.994	11.0057
cit	MS	0.0118	0.6805	0.0173	3.34E-86	1311	376.81	934.19	160	153.414	6.58645
cit	GMYN	0.0117	0.7571	0.0155	1.91E-79	1311	365.424	945.576	160	149	11
adk	MS	0.0231	1.0322	0.0224	1.05E-56	663	176.891	486.109	98	92.3184	5.68161
adk	GMYN	0.0217	2.3886	0.0091	2.01E-57	663	142.78	520.22	98	86.9152	11.0848
ndk	MS	0.0068	0.3892	0.0176	1.40E-18	423	108.122	314.878	31	29.4898	1.51023

ndk	GMYN	0.0063	0.3899	0.0160	9.94E-18	423	101.342	321.658	31	29	2
			**maximum**	0.0324							
			**minimum**	0.0084							
			**average**	0.0180							
			**median**	0.0175							

Selected genes in MWH-MoK4

Sequence	Method	Ka	Ks	Ka/Ks	P-Value(Fisher)	Length	S-Sites	N-Sites	Substitutions	S-Substitutions	N-Substitutions
Alkyl	MS	0.0501	1.4584	0.0343	1.09E-49	435	105.063	329.937	81	73.1168	7.88318
Alkyl	GMYN	0.0501	1.6571	0.0302	1.16E-37	435	96.8239	338.176	81	64.647	16.353
Carbo	MS	0.0640	4.0729	0.0157	4.79E-129	630	147.092	482.908	151	143.591	7.40934
Carbo	GMYN	0.0695	2.4956	0.0279	1.39E-61	630	166.479	463.521	151	120.379	30.621
Cold	MS	0.0000	0.3117	0.0000	0	171	45.7305	125.269	10	9.99997	2.74E-05
Cold	GMYN	NA	0.3436	NA	0	171	39.0396	131.96	10	10	0
DnaK	MS	0.0191	0.8982	0.0213	1.60E-137	1893	554.357	1338.64	266	252.992	13.0085
DnaK	GMYN	0.0182	1.2457	0.0146	3.65E-135	1893	483.934	1409.07	266	240.74	25.2598
DSBA	MS	0.2109	3.8950	0.0541	NA	609	130.442	478.558	197	164.351	32.6492
DSBA	GMYN	0.2078	3.5746	0.0581	2.10E-54	609	117.458	491.542	197	108.721	88.2793
Fatty	MS	0.0546	1.6296	0.0335	4.31E-147	1161	280.18	880.82	238	215.329	22.6708
Fatty	GMYN	0.0510	1.8746	0.0272	9.80E-107	1161	282.956	878.044	238	194.787	43.2133
Fimb	MS	0.0550	3.6437	0.0151	3.75E-96	480	126.395	353.605	122	117.054	4.94559
Fimb	GMYN	0.0563	3.6370	0.0155	2.90E-60	480	127.652	352.348	122	102.96	19.0403
FtsK	MS	NA	NA	NA	NA	NA	NA	NA	NA	NA	NA
FtsK	GMYN	0.0330	1.2264	0.0269	1.94E-196	2271	620.571	1650.43	428	374.709	53.2915
GroL	MS	0.0315	0.6930	0.0454	1.64E-95	1614	436.828	1177.17	210	187.113	22.8874
GroL	GMYN	0.0295	0.8056	0.0366	4.83E-85	1614	398.948	1215.05	210	174.942	35.0579
Hfq	MS	0.0346	1.3306	0.0260	1.16E-21	210	58.9698	151.03	37	34.6928	2.30717
Hfq	GMYN	0.0331	1.2354	0.0268	9.32E-19	210	52.7496	157.25	37	31.943	5.057
Piri	MS	0.1525	3.7022	0.0412	NA	666	159.009	506.991	190	167.943	22.057
Piri	GMYN	0.1515	2.7019	0.0561	4.72E-47	666	167.49	498.51	190	122.194	67.8064
RpoE	MS	0.0346	0.8836	0.0391	3.37E-41	537	122.034	414.966	74	65.3127	8.68725
RpoE	GMYN	0.0343	0.7338	0.0467	6.69E-29	537	138.458	398.542	74	60.6677	13.3323

RpoH	MS	0.0654	1.7953	0.0364	2.41E-113	906	254.257	651.743	204	186.581	17.4195
RpoH	GMYN	0.0616	3.2919	0.0187	1.53E-90	906	220.911	685.089	204	163.627	40.3726
RuvB	MS	0.0226	2.6409	0.0085	3.67E-159	1032	265.99	766.01	216	210.81	5.18987
RuvB	GMYN	0.0218	4.0990	0.0053	8.15E-151	1032	236.349	795.651	216	198.91	17.0905
Superox	MS	0.1478	3.9317	0.0376	NA	501	112.829	388.171	147	130.161	16.8386
Superox	GMYN	0.1545	3.5797	0.0432	2.51E-40	501	118.272	382.728	147	93.8519	53.1481
			**maximum**	0.0581							
			**minimum**	0.0053							
			**average**	0.0332							
			**median**	0.0364							

Housekeeping genes in MWH-MoK4

Sequence	Method	Ka	Ks	Ka/Ks	P-Value(Fisher)	Length	S-Sites	N-Sites	Substitutions	S-Substitutions	N-Substitutions
iso	MS	0.0449	1.3134	0.0342	7.77E-219	2232	594.613	1637.39	406	371.058	34.942
iso	GMYN	0.0436	1.3547	0.0322	1.17E-180	2232	533.201	1698.8	406	334.209	71.7912
acsA	MS	0.0332	0.8538	0.0389	3.33E-145	1971	529.54	1441.46	297	268.591	28.4093
acsA	GMYN	0.0316	1.4453	0.0218	9.23E-142	1971	442.811	1528.19	297	249.879	47.1209
succ	MS	0.0264	1.2285	0.0215	2.73E-172	1776	450.743	1325.26	288	270.908	17.0919
succ	GMYN	0.0252	1.7650	0.0143	1.62E-172	1776	373.114	1402.89	288	253.238	34.7617
CTP	MS	0.0452	2.1968	0.0206	1.82E-239	1659	389.105	1269.89	339	317.671	21.3295
CTP	GMYN	0.0455	4.3991	0.0103	4.97E-188	1659	352.653	1306.35	339	281.475	57.5253
atpA	MS	0.0073	0.5753	0.0127	1.77E-92	1539	437.524	1101.48	168	162.798	5.20194
atpA	GMYN	0.0074	0.5742	0.0129	2.82E-84	1539	449.593	1089.41	168	159.998	8.00241
gyr	MS	0.0440	2.8297	0.0156	1.63E-214	1434	397.871	1036.13	323	310.426	12.5738
gyr	GMYN	0.0432	3.3716	0.0128	2.55E-161	1434	375.774	1058.23	323	278.685	44.3148
glnA	MS	0.0234	0.5632	0.0415	9.55E-74	1413	379.737	1033.26	161	144.653	16.3467
glnA	GMYN	0.0223	0.9748	0.0228	1.26E-75	1413	314.461	1098.54	161	136.929	24.0706
cit	MS	0.0248	1.0765	0.0230	1.78E-104	1311	403.661	907.339	207	196.811	10.1895
cit	GMYN	0.0236	1.3596	0.0173	3.04E-96	1311	359.966	951.034	207	184.961	22.0394
adk	MS	0.0423	0.8510	0.0497	2.46E-46	663	175.563	487.437	100	87.8641	12.1359
adk	GMYN	0.0392	2.1557	0.0182	1.77E-47	663	133.984	529.016	100	79.8747	20.1253
ndk	MS	0.0271	0.6862	0.0394	9.61E-26	423	92.2277	330.772	46	40.3017	5.69831
ndk	GMYN	0.0263	0.9812	0.0268	2.48E-24	423	73.4068	349.593	46	36.9863	9.01365

**maximum**	0.0497
**minimum**	0.0103
**average**	0.0243
**median**	0.0217

Selected genes in MWH-Recht1

Sequence	Method	Ka	Ks	Ka/Ks	P-Value(Fisher)	Length	S-Sites	N-Sites	Substitutions	S-Substitutions	N-Substitutions
Methyl	MS	0.0754	1.1880	0.0635	1.18E-128	1470	350.71	1119.29	276	229.493	46.5069
Methyl	GMYN	0.0697	2.3825	0.0292	7.38E-89	1470	348.153	1121.85	276	201.683	74.3171
RpoE	MS	0.0045	0.0336	0.1354	0.00299834	567	132.04	434.96	6	4.14927	1.85073
RpoE	GMYN	0.0047	0.0289	0.1636	0.0042709	567	142.236	424.764	6	4	2
			**maximum**	0.1636							
			**minimum**	0.0292							
			**average**	0.0979							
			**median**	0.0995							

Housekeeping genes in MWH-Recht1

Sequence	Method	Ka	Ks	Ka/Ks	P-Value(Fisher)	Length	S-Sites	N-Sites	Substitutions	S-Substitutions	N-Substitutions
iso	MS	0.0000	0.0000	0.8609	0	2232	517.539	1714.46	0	0	0
iso	GMYN	NA	0.0000	NA	0	2232	555.654	1676.35	0	0	0
acsA	MS	0.0013	0.0022	0.5994	0.137823	1971	487	1484	3	1.06134	1.93866
acsA	GMYN	0.0014	0.0020	0.6865	0.571049	1971	503.725	1467.28	3	1	2
succ	MS	0.0000	0.0000	0.8609	0	1776	410.966	1365.03	0	0	0
succ	GMYN	0.0000	NA	NA	0	1776	437.879	1338.12	0	0	0
CTP	MS	0.0000	0.0000	0.8609	0	1659	396.751	1262.25	0	0	0
CTP	GMYN	0.0000	0.0000	NA	0	1659	418.508	1240.49	0	0	0
atpA	MS	0.0000	0.0000	0.8609	0	1539	380.541	1158.46	0	0	0
atpA	GMYN	NA	0.0000	NA	0	1539	407.726	1131.27	0	0	0
gyr	MS	0.0000	0.0000	0.8610	0	1434	378.656	1055.34	0	0	0
gyr	GMYN	0.0000	NA	NA	0	1434	387.291	1046.71	0	0	0
glnA	MS	0.0000	0.0030	0.0000	0	1413	332.108	1080.89	1	0.999997	3.25E-06

glnA	GMYN	0.0000	0.0029	0.0000	0	1413	349.801	1063.2	1	1	0
cit	MS	0.0000	0.0034	0.0000	0	1311	291.309	1019.69	1	0.999996	3.50E-06
cit	GMYN	0.0000	0.0032	0.0000	0	1311	312.796	998.204	1	1	0
adk	MS	0.0079	0.1992	0.0397	6.71E-15	663	159.472	503.528	30	26.6596	3.34038
adk	GMYN	0.0078	0.2078	0.0375	1.59E-14	663	146.132	516.868	30	26	4
ndk	MS	0.0000	0.0000	0.8603	0	423	97.5858	325.414	0	0	0
ndk	GMYN	NA	NA	NA	0	423	97.8687	325.131	0	0	0
			**maximum**	0.8610							
			**minimum**	0.0000							
			**average**	0.4663							
			**median**	0.6430							

Selected genes in MWH-RechtKol4

Sequence	Method	Ka	Ks	Ka/Ks	P-Value(Fisher)	Length	S-Sites	N-Sites	Substitutions	S-Substitutions	N-Substitutions
FtsK	MS	0.0024	0.0345	0.0709	4.98E-10	2310	682.337	1627.66	26	22.2388	3.76118
FtsK	GMYN	0.0024	0.0362	0.0657	1.18E-09	2310	625.81	1684.19	26	22	4
Alkyl	MS	0.0031	0.0443	0.0708	0	465	144.282	320.718	7	6.04791	0.952093
Alkyl	GMYN	0.0029	0.0532	0.0549	0.00146005	465	120.669	344.331	7	6	1
DSBA	MS	0.0069	0.0285	0.2402	0.0100676	624	184.867	439.133	8	5.09395	2.90605
DSBA	GMYN	0.0065	0.0321	0.2029	0.0295253	624	160.064	463.936	8	5	3
Fimb	MS	0.0086	0.1329	0.0650	9.77E-09	510	147.434	362.566	20	17.2435	2.75652
Fimb	GMYN	0.0107	0.1371	0.0777	2.66E-07	510	129.6	380.4	20	16	4
GroL	MS	0.0009	0.0020	0.4437	0	1650	523.849	1126.15	2	1.02367	0.976329
GroL	GMYN	0.0008	0.0023	0.3496	0.384551	1650	428.172	1221.83	2	1	1
Hyp	MS	0.0049	0.0001	50.0000	0	264	62.6998	201.3	1	0.00619091	0.993809
Hyp	GMYN	0.0050	0.0000	NA	0	264	64.3212	199.679	1	0	1
MscS	MS	0.0000	0.0437	0.0000	0	819	216.856	602.144	9	8.99998	2.50E-05
MscS	GMYN	NA	0.0455	NA	0	819	205.048	613.952	9	9	0
Piri	MS	0.0055	0.0070	0.7869	0.214849	699	156.124	542.876	4	1.07058	2.92942
Piri	GMYN	0.0056	0.0061	0.9257	0.658823	699	165.625	533.375	4	1	3
RpoE	MS	0.0000	0.0151	0.0000	0	567	132.016	434.984	2	1.99999	6.59E-06
RpoE	GMYN	NA	0.0142	NA	0	567	142.534	424.466	2	2	0

RuvB	MS	0.0013	0.0715	0.0187	0	1068	318.884	749.116	22	21.0732	0.926773
RuvB	GMYN	0.0013	0.0836	0.0150	2.76E-12	1068	270.522	797.478	22	21	1
Superox	MS	0.0000	0.0082	0.0000	0	531	122.67	408.33	1	0.999997	3.33E-06
Superox	GMYN	0.0000	0.0078	0.0000	0	531	130.956	400.044	1	1	0
			**maximum**	50.0000							
			**minimum**	0.0000							
			**average**	2.8099							
			**median**	0.0708							

Housekeeping genes in MWH-RechtKol4

Sequence	Method	Ka	Ks	Ka/Ks	P-Value(Fisher)	Length	S-Sites	N-Sites	Substitutions	S-Substitutions	N-Substitutions
iso	MS	0.0019	0.0942	0.0201	1.10E-28	2232	646.227	1585.77	58	55.2741	2.72588
iso	GMYN	0.0019	0.0891	0.0216	4.46E-26	2232	670.713	1561.29	58	54.9983	3.0017
acsA	MS	0.0028	0.0711	0.0392	1.51E-17	1971	547.583	1423.42	39	35.392	3.60804
acsA	GMYN	0.0028	0.0699	0.0399	1.30E-16	1971	530.919	1440.08	39	35	4
succ	MS	0.0000	0.0366	0.0000	0	1776	430.6	1345.4	15	15	4.69E-05
succ	GMYN	0.0000	0.0369	0.0000	0	1776	421.093	1354.91	15	15	0
CTP	MS	0.0018	0.0190	0.0931	7.22E-05	1659	525.286	1133.71	11	9.15969	1.84031
CTP	GMYN	0.0016	0.0221	0.0730	0.000125077	1659	417.189	1241.81	11	9	2
atpA	MS	0.0000	0.0096	0.0000	0	1539	533.542	1005.46	5	4.99999	9.42E-06
atpA	GMYN	0.0000	0.0124	0.0000	0	1539	407.975	1131.02	5	5	0
gyr	MS	0.0000	0.0079	0.0000	0	1434	378.849	1055.15	3	2.99999	8.36E-06
gyr	GMYN	NA	0.0076	NA	0	1434	395.131	1038.87	3	3	0
glnA	MS	0.0000	0.0247	0.0000	0	1413	393.521	1019.48	9	8.99998	2.33E-05
glnA	GMYN	0.0000	0.0264	0.0000	0	1413	348.816	1064.18	9	9	0
cit	MS	0.0016	0.0234	0.0684	0.000382964	1311	267.118	1043.88	7	5.52364	1.47636
cit	GMYN	0.0021	0.0160	0.1290	0.0101498	1311	312.415	998.585	7	4.9408	2.0592
adk	MS	0.0083	0.1850	0.0449	2.00E-14	663	181.819	481.181	32	28.5993	3.40068
adk	GMYN	0.0090	0.1475	0.0609	5.40E-11	663	215.007	447.993	32	28	4
ndk	MS	0.0000	0.0000	0.8603	0	423	97.5858	325.414	0	0	0
ndk	GMYN	NA	NA	NA	0	423	97.8687	325.131	0	0	0
			**maximum**	0.8603							
			**minimum**	0.0000							

average	0.0806	
median	0.0304	

Selected genes in MWH-RechtKolB

Sequence	Method	Ka	Ks	Ka/Ks	P-Value(Fisher)	Length	S-Sites	N-Sites	Substitutions	S-Substitutions	N-Substitutions
FtsK	MS	0.0024	0.0345	0.0709	4.98E-10	2310	682.337	1627.66	26	22.2388	3.76118
FtsK	GMYN	0.0024	0.0362	0.0657	1.18E-09	2310	625.81	1684.19	26	22	4
Alkyl	MS	0.0031	0.0443	0.0708	0	465	144.282	320.718	7	6.04791	0.952093
Alkyl	GMYN	0.0029	0.0532	0.0549	0.00146005	465	120.669	344.331	7	6	1
DSBA	MS	0.0069	0.0285	0.2402	0.0100676	624	184.867	439.133	8	5.09395	2.90605
DSBA	GMYN	0.0065	0.0321	0.2029	0.0295253	624	160.064	463.936	8	5	3
Fimb	MS	0.0086	0.1329	0.0650	9.77E-09	510	147.434	362.566	20	17.2435	2.75652
Fimb	GMYN	0.0107	0.1371	0.0777	2.66E-07	510	129.6	380.4	20	16	4
GroL	MS	0.0009	0.0020	0.4437	0	1650	523.849	1126.15	2	1.02367	0.976329
GroL	GMYN	0.0008	0.0023	0.3496	0.384551	1650	428.172	1221.83	2	1	1
Hyp	MS	0.0049	0.0001	50.0000	0	264	62.6998	201.3	1	0.00619091	0.993809
Hyp	GMYN	0.0050	0.0000	NA	0	264	64.3212	199.679	1	0	1
MscS	MS	0.0000	0.0437	0.0000	0	819	216.856	602.144	9	8.99998	2.50E-05
MscS	GMYN	NA	0.0455	NA	0	819	205.048	613.952	9	9	0
Piri	MS	0.0055	0.0070	0.7869	0.214849	699	156.124	542.876	4	1.07058	2.92942
Piri	GMYN	0.0056	0.0061	0.9257	0.658823	699	165.625	533.375	4	1	3
RpoE	MS	0.0000	0.0151	0.0000	0	567	132.016	434.984	2	1.99999	6.59E-06
RpoE	GMYN	NA	0.0142	NA	0	567	142.534	424.466	2	2	0
RuvB	MS	0.0013	0.0715	0.0187	0	1068	318.884	749.116	22	21.0732	0.926773
RuvB	GMYN	0.0013	0.0836	0.0150	2.76E-12	1068	270.522	797.478	22	21	1
Superox	MS	0.0000	0.0082	0.0000	0	531	122.67	408.33	1	0.999997	3.33E-06
Superox	GMYN	0.0000	0.0078	0.0000	0	531	130.956	400.044	1	1	0
			maximum	50.0000							
			minimum	0.0000							
			average	2.8099							
			median	0.0708							

Housekeeping genes in MWH-RechtKolB

Sequence	Method	Ka	Ks	Ka/Ks	P-Value(Fisher)	Length	S-Sites	N-Sites	Substitutions	S-Substitutions	N-Substitutions
iso	MS	0.0019	0.0942	0.0201	1.10E-28	2232	646.227	1585.77	58	55.2741	2.72588
iso	GMYN	0.0019	0.0891	0.0216	4.46E-26	2232	670.713	1561.29	58	54.9983	3.0017
acsA	MS	0.0028	0.0711	0.0392	1.51E-17	1971	547.583	1423.42	39	35.392	3.60804
acsA	GMYN	0.0028	0.0699	0.0399	1.30E-16	1971	530.919	1440.08	39	35	4
succ	MS	0.0000	0.0366	0.0000	0	1776	430.6	1345.4	15	15	4.69E-05
succ	GMYN	0.0000	0.0369	0.0000	0	1776	421.093	1354.91	15	15	0
CTP	MS	0.0018	0.0190	0.0931	7.22E-05	1659	525.286	1133.71	11	9.15969	1.84031
CTP	GMYN	0.0016	0.0221	0.0730	0.000125077	1659	417.189	1241.81	11	9	2
atpA	MS	0.0000	0.0096	0.0000	0	1539	533.542	1005.46	5	4.99999	9.42E-06
atpA	GMYN	0.0000	0.0124	0.0000	0	1539	407.975	1131.02	5	5	0
gyr	MS	0.0000	0.0079	0.0000	0	1434	378.849	1055.15	3	2.99999	8.36E-06
gyr	GMYN	NA	0.0076	NA	0	1434	395.131	1038.87	3	3	0
glnA	MS	0.0000	0.0247	0.0000	0	1413	393.521	1019.48	9	8.99998	2.33E-05
glnA	GMYN	0.0000	0.0264	0.0000	0	1413	348.816	1064.18	9	9	0
cit	MS	0.0016	0.0234	0.0684	0.000382964	1311	267.118	1043.88	7	5.52364	1.47636
cit	GMYN	0.0021	0.0160	0.1290	0.0101498	1311	312.415	998.585	7	4.9408	2.0592
adk	MS	0.0083	0.1850	0.0449	2.00E-14	663	181.819	481.181	32	28.5993	3.40068
adk	GMYN	0.0090	0.1475	0.0609	5.40E-11	663	215.007	447.993	32	28	4
ndk	MS	0.0000	0.0000	0.8603	0	423	97.5858	325.414	0	0	0
ndk	GMYN	NA	NA	NA	0	423	97.8687	325.131	0	0	0
			**maximum**	0.8603							
			**minimum**	0.0000							
			**average**	0.0806							
			**median**	0.0304							

Selected genes in MWH-Tro7-1-4

Sequence	Method	Ka	Ks	Ka/Ks	P-Value(Fisher)	Length	S-Sites	N-Sites	Substitutions	S-Substitutions	N-Substitutions
RuvB	MS	0.0000	0.0037	0.0000	0	1068	272.786	795.214	1	0.999997	2.92E-06
RuvB	GMYN	0.0000	0.0036	0.0000	0	1068	282.739	785.261	1	1	0

Fimb	MS	0.0055	0.0540	0.1012	0.00017699	510	139.271	370.729	9	7.09043	1.90957
Fimb	GMYN	0.0054	0.0552	0.0972	0.00180129	510	135.493	374.507	9	7	2
RpoE	MS	0.0045	0.0336	0.1354	0.00299834	567	132.04	434.96	6	4.14927	1.85073
RpoE	GMYN	0.0047	0.0289	0.1636	0.0042709	567	142.236	424.764	6	4	2
Superox	MS	0.0083	0.0606	0.1365	0.000221587	531	163.432	367.568	12	9.18071	2.81929
Superox	GMYN	0.0075	0.0772	0.0974	0.00026245	531	129.114	401.886	12	9	3
			**maximum**	0.1636							
			**minimum**	0.0000							
			**average**	0.0914							
			**median**	0.0993							

Housekeeping genes in MWH-Tro7-1-4

Sequence	Method	Ka	Ks	Ka/Ks	P-Value(Fisher)	Length	S-Sites	N-Sites	Substitutions	S-Substitutions	N-Substitutions
iso	MS	0.0006	0.0236	0.0252	0	2232	561.142	1670.86	14	13.0219	0.978057
iso	GMYN	0.0006	0.0239	0.0250	1.35E-07	2232	555.944	1676.06	14	13	1
acsA	MS	0.0015	0.0000	50.0000	0.434142	1971	627.238	1343.76	2	0.0184984	1.9815
acsA	GMYN	0.0014	0.0000	1.2303E+12	0	1971	503.109	1467.89	2	0	2
succ	MS	0.0000	0.0000	0.8609	0	1776	410.966	1365.03	0	0	0
succ	GMYN	0.0000	NA	NA	0	1776	437.879	1338.12	0	0	0
CTP	MS	0.0000	0.0000	0.8609	0	1659	396.751	1262.25	0	0	0
CTP	GMYN	0.0000	0.0000	NA	0	1659	418.508	1240.49	0	0	0
atpA	MS	0.0000	0.0000	0.8609	0	1539	380.541	1158.46	0	0	0
atpA	GMYN	NA	0.0000	NA	0	1539	407.726	1131.27	0	0	0
gyr	MS	0.0000	0.0144	0.0000	0	1434	430.428	1003.57	6	5.99999	1.40E-05
gyr	GMYN	NA	0.0149	NA	0	1434	408.105	1025.9	6	6	0
glnA	MS	0.0000	0.0060	0.0000	0	1413	332.456	1080.54	2	1.99999	6.50E-06
glnA	GMYN	NA	0.0057	NA	0	1413	350.238	1062.76	2	2	0
cit	MS	0.0000	0.0000	0.8607	0	1311	291.476	1019.52	0	0	0
cit	GMYN	NA	NA	NA	0	1311	313.061	997.939	0	0	0
adk	MS	0.0000	0.0000	0.8610	0	663	160.97	502.03	0	0	0
adk	GMYN	0.0000	NA	NA	0	663	170.565	492.435	0	0	0
ndk	MS	0.0031	0.0001	50.0000	0	423	97.5862	325.414	1	0.00596191	0.994038
ndk	GMYN	0.0031	NA	NA	0	423	97.7649	325.235	1	0	1

**maximum**	1.2303E+12
**minimum**	0.0000
**average**	1.0252E+11
**median**	0.8609

Selected genes in MWH-Tro8-2-5gr

Sequence	Method	Ka	Ks	Ka/Ks	P-Value(Fisher)	Length	S-Sites	N-Sites	Substitutions	S-Substitutions	N-Substitutions
Alkyl	MS	0.0309	2.5430	0.0121	3.46E-65	444	108.251	335.749	88	84.8045	3.19553
Alkyl	GMYN	0.0333	4.3104	0.0077	4.72E-51	444	102.89	341.11	88	76.9209	11.0791
Cold	MS	0.0000	0.0495	0.0000	0	180	40.3807	139.619	2	1.99999	6.92E-06
Cold	GMYN	0.0000	0.0516	0.0000	0	180	42.6501	137.35	2	2	0
DnaK	MS	0.0120	0.7478	0.0161	6.32E-128	1899	549.353	1349.65	239	229.928	9.07215
DnaK	GMYN	0.0115	0.8884	0.0130	2.93E-129	1899	489.13	1409.87	239	222.916	16.0843
DSBA	MS	0.1276	2.9315	0.0435	NA	603	138.561	464.439	163	142.251	20.7487
DSBA	GMYN	0.1248	3.6412	0.0343	1.15E-57	603	128.368	474.632	163	108.823	54.1775
Fatty	MS	0.0642	2.4045	0.0267	2.80E-189	1167	268.666	898.334	262	240.539	21.4612
Fatty	GMYN	0.0623	4.1310	0.0151	1.00E-135	1167	246.651	920.349	262	207.187	54.8133
Fimb	MS	0.0559	3.8195	0.0146	1.50E-86	498	124.921	373.079	113	108.266	4.73378
Fimb	GMYN	0.0562	3.6049	0.0156	4.11E-54	498	122.299	375.701	113	92.7167	20.2833
FtsK	MS	0.0149	1.0308	0.0145	1.69E-209	2277	623.013	1653.99	354	340.878	13.1224
FtsK	GMYN	0.0139	1.3083	0.0106	7.42E-195	2277	606.13	1670.87	354	330.962	23.038
GroL	MS	0.0060	0.5393	0.0111	7.01E-90	1620	460.493	1159.51	162	157.582	4.41769
GroL	GMYN	0.0058	0.6509	0.0088	1.40E-95	1620	399.484	1220.52	162	155	7.00048
Hfq	MS	0.0330	0.5506	0.0598	2.25E-12	228	67.352	160.648	28	24.5025	3.49753
Hfq	GMYN	0.0307	1.0094	0.0304	7.28E-11	228	61.2365	166.763	28	23	5
Piri	MS	0.1350	3.1715	0.0426	NA	675	144.253	530.747	176	152.168	23.8322
Piri	GMYN	0.1387	6.1000	0.0227	6.44E-48	675	146.142	528.858	176	109.574	66.426
RpoE	MS	NA	NA	NA	NA	NA	NA	NA	NA	NA	NA
RpoE	GMYN	0.0305	0.7767	0.0393	3.39E-31	546	136.588	409.412	74	61.7805	12.2195
RpoH	MS	0.0322	1.1725	0.0274	1.47E-82	903	262.057	640.943	155	145.254	9.74615
RpoH	GMYN	0.0298	1.3640	0.0218	1.54E-80	903	214.772	688.228	155	134.944	20.0557
Rubre	MS	0.0367	2.7185	0.0135	6.40E-50	462	147.886	314.114	88	85.5493	2.45071

Rubre	GMYN	0.0345	1.7929	0.0192	2.66E-38	462	134.721	327.279	88	77	11
			**maximum**	0.0598							
			**minimum**	0.0000							
			**average**	0.0208							
			**median**	0.0156							

Housekeeping genes in MWH-Tro8-2-5gr

Sequence	Method	Ka	Ks	Ka/Ks	P-Value(Fisher)	Length	S-Sites	N-Sites	Substitutions	S-Substitutions	N-Substitutions
iso	MS	0.0223	0.9998	0.0223	1.13E-190	2232	596.507	1635.49	339	319.447	19.5526
iso	GMYN	0.0222	1.3559	0.0164	2.72E-166	2232	575.916	1656.08	339	302.855	36.1454
acsA	MS	0.0303	0.8617	0.0351	6.64E-147	1971	493.945	1477.05	281	254.288	26.7124
acsA	GMYN	0.0272	1.5565	0.0175	3.77E-141	1971	432.585	1538.41	281	239.927	41.0729
succ	MS	0.0126	0.8678	0.0145	1.08E-139	1776	459.187	1316.81	233	223.668	9.33209
succ	GMYN	0.0111	1.6003	0.0069	3.14E-141	1776	413.624	1362.38	233	218	15.0005
CTP	MS	0.0291	1.8451	0.0158	1.39E-216	1659	396.265	1262.73	309	294.203	14.7971
CTP	GMYN	0.0292	2.0363	0.0143	9.17E-174	1659	389.391	1269.61	309	272.709	36.2908
atpA	MS	0.0029	0.2471	0.0119	5.50E-48	1539	416.33	1122.67	86	83.3313	2.66866
atpA	GMYN	0.0026	0.2821	0.0092	8.33E-50	1539	378.591	1160.41	86	82.9997	3.00028
gyr	MS	0.0306	1.9569	0.0156	6.70E-173	1434	394.482	1039.52	275	264.113	10.8867
gyr	GMYN	0.0314	1.7556	0.0179	3.20E-125	1434	410.89	1023.11	275	243.578	31.4223
glnA	MS	0.0163	0.4674	0.0349	3.72E-65	1413	369.219	1043.78	136	123.8	12.2004
glnA	GMYN	0.0140	0.8743	0.0161	5.05E-67	1413	332.257	1080.74	136	120.992	15.0082
cit	MS	0.0140	0.8290	0.0169	3.17E-103	1311	318.87	992.13	168	159.61	8.38997
cit	GMYN	0.0128	2.1119	0.0061	3.95E-105	1311	281.381	1029.62	168	154.948	13.0524
adk	MS	0.0281	0.8887	0.0316	2.92E-49	663	190.603	472.397	96	89.0224	6.97763
adk	GMYN	0.0270	1.0241	0.0263	2.16E-43	663	169.874	493.126	96	82.9971	13.0029
ndk	MS	0.0029	0.4709	0.0063	0	423	87.6668	335.333	29	28.3217	0.678306
ndk	GMYN	0.0030	0.4702	0.0065	3.64E-19	423	94.1205	328.879	29	28	1
			**maximum**	0.0351							
			**minimum**	0.0061							
			**average**	0.0171							
			**median**	0.0159							

Selected genes in MWH-Tro8-2-9

Sequence	Method	Ka	Ks	Ka/Ks	P-Value(Fisher)	Length	S-Sites	N-Sites	Substitutions	S-Substitutions	N-Substitutions
RpoE	MS	0.0000	0.0076	0.0000	0	567	131.79	435.21	1	0.999997	3.30E-06
RpoE	GMYN	0.0000	0.0071	0.0000	0	567	142.392	424.608	1	1	0
			**maximum**	0.0000							
			**minimum**	0.0000							
			**average**	0.0000							
			**median**	0.0000							

Housekeeping genes in MWH-Tro8-2-9

Sequence	Method	Ka	Ks	Ka/Ks	P-Value(Fisher)	Length	S-Sites	N-Sites	Substitutions	S-Substitutions	N-Substitutions
iso	MS	0.0000	0.0000	0.8609	0	2232	517.539	1714.46	0	0	0
iso	GMYN	NA	0.0000	NA	0	2232	555.654	1676.35	0	0	0
acsA	MS	0.0007	0.0247	0.0269	0	1971	486.858	1484.14	12	11.0915	0.908467
acsA	GMYN	0.0007	0.0222	0.0307	2.52E-06	1971	503.811	1467.19	12	11	1
succ	MS	0.0000	0.0000	0.8609	0	1776	410.966	1365.03	0	0	0
succ	GMYN	0.0000	NA	NA	0	1776	437.879	1338.12	0	0	0
CTP	MS	0.0000	0.0000	0.8609	0	1659	396.751	1262.25	0	0	0
CTP	GMYN	0.0000	0.0000	NA	0	1659	418.508	1240.49	0	0	0
atpA	MS	0.0000	0.0000	0.8609	0	1539	380.541	1158.46	0	0	0
atpA	GMYN	NA	0.0000	NA	0	1539	407.726	1131.27	0	0	0
gyr	MS	0.0000	0.0000	0.8610	0	1434	378.656	1055.34	0	0	0
gyr	GMYN	0.0000	NA	NA	0	1434	387.291	1046.71	0	0	0
glnA	MS	0.0000	0.0000	0.8607	0	1413	332.453	1080.55	0	0	0
glnA	GMYN	0.0000	0.0000	NA	0	1413	350.104	1062.9	0	0	0
cit	MS	0.0000	0.0034	0.0000	0	1311	291.309	1019.69	1	0.999996	3.50E-06
cit	GMYN	0.0000	0.0032	0.0000	0	1311	312.796	998.204	1	1	0
adk	MS	0.0000	0.0000	0.8610	0	663	160.97	502.03	0	0	0
adk	GMYN	0.0000	NA	NA	0	663	170.565	492.435	0	0	0
ndk	MS	0.0000	0.0102	0.0000	0	423	98.1022	324.898	1	0.999997	3.31E-06
ndk	GMYN	0.0000	0.0103	0.0000	0	423	98.4044	324.596	1	1	0

**maximum**	0.8610
**minimum**	0.0000
**average**	0.4680
**median**	0.8607

Selected genes in MWH-UH21B

Sequence	Method	Ka	Ks	Ka/Ks	P-Value(Fisher)	Length	S-Sites	N-Sites	Substitutions	S-Substitutions	N-Substitutions
FtsK	MS	0.0222	1.2567	0.0177	1.08E-233	2292	651.722	1640.28	405	387.739	17.2606
FtsK	GMYN	0.0229	1.2204	0.0188	1.41E-185	2292	685.669	1606.33	405	368.807	36.1931
Alkyl	MS	0.0605	2.5784	0.0235	5.54E-73	459	112.785	346.215	102	95.1455	6.85449
Alkyl	GMYN	0.0621	3.4839	0.0178	1.74E-48	459	104.089	354.911	102	80.9172	21.0828
Carbo	MS	0.0326	2.0699	0.0158	4.94E-94	654	149.461	504.539	126	119.631	6.36862
Carbo	GMYN	0.0327	2.6131	0.0125	2.31E-71	654	151.246	502.754	126	109.954	16.0458
Cold	MS	0.0000	0.1104	0.0000	0	195	45.2978	149.702	5	4.99998	1.65E-05
Cold	GMYN	NA	0.1144	NA	0	195	48.3411	146.659	5	5	0
DnaK	MS	0.0163	0.9043	0.0180	3.02E-146	1917	539.196	1377.8	265	253.358	11.6424
DnaK	GMYN	0.0156	1.1806	0.0132	2.47E-141	1917	483.017	1433.98	265	242.828	22.1722
DSBA	MS	0.1640	3.8153	0.0430	NA	618	141.504	476.496	187	163.361	23.6387
DSBA	GMYN	0.1667	3.6609	0.0455	1.10E-55	618	131.796	486.204	187	114.925	72.0753
Fatty	MS	0.0708	1.9511	0.0363	3.10E-164	1182	312.083	869.917	276	250.66	25.3403
Fatty	GMYN	0.0672	4.1833	0.0161	2.82E-133	1182	264.481	917.519	276	217.206	58.7944
Fimb	MS	0.0778	3.6881	0.0211	1.30E-91	510	130.276	379.724	125	117.764	7.23638
Fimb	GMYN	0.0788	3.6154	0.0218	5.38E-53	510	124.028	385.972	125	96.2343	28.7657
GroL	MS	0.0098	0.7316	0.0134	6.69E-107	1635	445.156	1189.84	187	180.532	6.46815
GroL	GMYN	0.0096	0.8940	0.0108	1.68E-111	1635	377.041	1257.96	187	174.998	12.0019
Hfq	MS	0.0422	0.9785	0.0431	6.20E-20	234	60.883	173.117	38	33.8495	4.15045
Hfq	GMYN	0.0414	1.2440	0.0333	8.08E-16	234	59.4434	174.557	38	30.9876	7.01241
Piri	MS	0.1323	4.4088	0.0300	NA	690	139.996	550.004	191	170.853	20.1475
Piri	GMYN	0.1361	3.7244	0.0365	2.62E-65	690	143.423	546.577	191	123.533	67.4672
RpoH	MS	0.0392	1.2508	0.0313	1.71E-91	912	255.122	656.878	169	156.386	12.6136
RpoH	GMYN	0.0378	2.9140	0.0130	1.69E-78	912	230.945	681.055	169	143.987	25.0134
RuvB	MS	0.0229	3.3218	0.0069	1.49E-179	1053	255.307	797.693	222	217.314	4.68602

RuvB	GMYN	0.0227	4.1202	0.0055	7.49E-153	1053	243.125	809.875	222	203.947	18.0532
			**maximum**	0.0455							
			**minimum**	0.0000							
			**average**	0.0218							
			**median**	0.0180							

Housekeeping genes in MWH-UH21B

Sequence	Method	Ka	Ks	Ka/Ks	P-Value(Fisher)	Length	S-Sites	N-Sites	Substitutions	S-Substitutions	N-Substitutions
iso	MS	0.0246	1.0173	0.0242	7.82E-196	2232	578.399	1653.6	345	322.713	22.287
iso	GMYN	0.0239	2.2572	0.0106	1.91E-182	2232	522.921	1709.08	345	304.774	40.2261
acsA	MS	0.0335	0.9679	0.0346	1.03E-150	1971	592.697	1378.3	318	294.294	23.7064
acsA	GMYN	0.0307	1.1675	0.0263	7.83E-157	1971	460.746	1510.25	318	272.702	45.2985
succ	MS	0.0249	0.8416	0.0296	3.14E-131	1776	464.767	1311.23	249	229.811	19.1886
succ	GMYN	0.0235	2.8881	0.0081	2.81E-134	1776	383.146	1392.85	249	216.877	32.1227
CTP	MS	0.0311	2.2809	0.0136	7.07E-232	1659	402.797	1256.2	324	310.796	13.2037
CTP	GMYN	0.0283	2.8327	0.0100	1.49E-188	1659	394.251	1264.75	324	288.919	35.0806
atpA	MS	NA	NA	NA	NA	NA	NA	NA	NA	NA	NA
atpA	GMYN	0.0099	0.3724	0.0265	1.84E-55	1539	417.596	1121.4	123	112	11
gyr	MS	0.0400	2.7876	0.0144	2.61E-202	1434	380.038	1053.96	299	287.548	11.4519
gyr	GMYN	0.0413	1.5850	0.0260	5.04E-137	1434	390.054	1043.95	299	257.19	41.8102
glnA	MS	0.0245	0.4959	0.0494	3.22E-65	1413	347.415	1065.59	144	125.049	18.9507
glnA	GMYN	0.0231	0.7397	0.0312	5.55E-64	1413	301.413	1111.59	144	118.749	25.2505
cit	MS	0.0220	0.9288	0.0237	2.36E-108	1311	325.072	985.928	186	173.521	12.4791
cit	GMYN	0.0194	1.5580	0.0124	2.85E-98	1311	316.295	994.705	186	166.995	19.0047
adk	MS	0.0273	1.6085	0.0170	2.16E-71	663	178.29	484.71	116	110.878	5.12185
adk	GMYN	0.0278	1.8006	0.0154	1.89E-66	663	149.552	513.448	116	101.997	14.0029
ndk	MS	0.0300	0.5242	0.0573	2.44E-19	423	112.061	310.939	45	38.829	6.17102
ndk	GMYN	0.0281	0.6091	0.0461	1.96E-18	423	94.9409	328.059	45	35.9672	9.03279
			**maximum**	0.0573							
			**minimum**	0.0081							
			**average**	0.0251							
			**median**	0.0242							

Selected genes in MWH-Weng1-1

Sequence	Method	Ka	Ks	Ka/Ks	P-Value(Fisher)	Length	S-Sites	N-Sites	Substitutions	S-Substitutions	N-Substitutions
FtsK	MS	0.0368	1.3299	0.0276	1.73E-229	2244	653.403	1590.6	430	402.885	27.1152
FtsK	GMYN	0.0365	1.2947	0.0282	9.82E-195	2244	602.918	1641.08	430	371.722	58.2781
DnaK	MS	0.0160	1.0334	0.0155	1.01E-143	1866	583.693	1282.31	277	267.866	9.13392
DnaK	GMYN	0.0160	0.7616	0.0210	6.21E-123	1866	585.487	1280.51	277	256.799	20.2014
GroL	MS	0.0173	0.6752	0.0256	5.00E-95	1590	479.487	1110.51	198	186.937	11.0633
GroL	GMYN	0.0163	0.7967	0.0204	2.08E-97	1590	408.032	1181.97	198	178.996	19.0042
Fatty	MS	0.0614	2.1489	0.0286	6.27E-186	1137	254.923	882.077	256	232.957	23.0428
Fatty	GMYN	0.0598	4.0524	0.0148	7.34E-151	1137	222.11	914.89	256	203.582	52.4181
RuvB	MS	0.0289	3.2835	0.0088	5.08E-168	1008	224.501	783.499	202	195.973	6.02699
RuvB	GMYN	0.0289	4.0509	0.0071	1.21E-130	1008	221.661	786.339	202	179.927	22.0727
RpoH	MS	0.0967	2.8548	0.0339	1.83E-142	885	232.111	652.889	222	202.684	19.3164
RpoH	GMYN	0.0880	4.0555	0.0217	8.64E-83	885	223.028	661.972	222	167.291	54.7091
MscS	MS	0.1795	3.6195	0.0496	NA	774	184.613	589.387	249	214.968	34.0321
MscS	GMYN	0.1792	3.9383	0.0455	4.08E-65	774	190.775	583.225	249	157.243	91.7568
Piri	MS	0.1342	4.1084	0.0327	NA	654	142.481	511.519	185	165.585	19.4154
Piri	GMYN	0.1383	3.7079	0.0373	1.97E-62	654	140.313	513.687	185	120.648	64.3521
Carbo	MS	0.0692	3.1345	0.0221	1.38E-109	624	144.509	479.491	142	132.305	9.6952
Carbo	GMYN	0.0699	3.6402	0.0192	1.67E-72	624	128.208	495.792	142	109.07	32.9296
DSBA	MS	0.2520	3.4307	0.0735	NA	591	139.069	451.931	224	180.83	43.1698
DSBA	GMYN	0.2600	3.6288	0.0717	2.50E-59	591	126.273	464.727	224	122.845	101.155
RpoE	MS	0.0425	1.6226	0.0262	1.53E-66	534	123.714	410.286	101	92.9237	8.07626
RpoE	GMYN	0.0453	1.4523	0.0312	2.25E-42	534	140.738	393.262	101	83.734	17.266
Fimb	MS	0.0546	3.8451	0.0142	4.05E-84	489	121.966	367.034	110	105.496	4.50405
Fimb	GMYN	0.0541	3.5853	0.0151	7.74E-54	489	119.156	369.844	110	90.7937	19.2063
Rubre	MS	0.0488	3.3624	0.0145	5.52E-58	450	129.181	320.819	92	88.8017	3.19828
Rubre	GMYN	0.0465	1.5976	0.0291	1.76E-41	450	116.512	333.488	92	77	15
Alkyl	MS	0.0440	1.6646	0.0264	1.07E-55	432	103.259	328.741	84	77.4847	6.51525
Alkyl	GMYN	0.0451	1.5451	0.0292	5.34E-39	432	106.656	325.344	84	69.7976	14.2024
Hfq	MS	0.0128	1.4557	0.0088	0	207	55.9527	151.047	32	31.2594	0.740612
Hfq	GMYN	0.0131	2.0777	0.0063	8.37E-20	207	52.0075	154.992	32	29.9994	2.00059

Cold	MS	0.0000	0.1806	0.0000	0	168	48.6253	119.375	7	6.99998	1.72E-05
Cold	GMYN	0.0000	0.2167	0.0000	0	168	40.1796	127.82	7	7	0
			**maximum**	0.0735							
			**minimum**	0.0000							
			**average**	0.0252							
			**median**	0.0238							

Housekeeping genes in MWH-Weng1-1

Sequence	Method	Ka	Ks	Ka/Ks	P-Value(Fisher)	Length	S-Sites	N-Sites	Substitutions	S-Substitutions	N-Substitutions
iso	MS	0.0126	1.4558	0.0087	1.58E-164	1527	417.299	1109.7	255	249.242	5.75769
iso	GMYN	0.0121	1.7223	0.0070	8.02E-170	1527	357.192	1169.81	255	240.939	14.0612
acsA	MS	0.0123	1.2940	0.0095	1.95E-137	1323	346.255	976.745	210	204.51	5.48991
acsA	GMYN	0.0120	1.3882	0.0086	3.91E-135	1323	313.179	1009.82	210	197.983	12.0165
succ	MS	0.0052	1.2822	0.0041	6.65E-120	1137	286.368	850.632	171	168.951	2.04913
succ	GMYN	0.0047	1.2350	0.0038	6.38E-119	1137	275.818	861.182	171	167	4.00027
CTP	MS	0.0157	1.5813	0.0099	1.40E-127	1044	271.359	772.641	188	182.824	5.1758
CTP	GMYN	0.0163	2.9439	0.0055	1.49E-126	1044	238.02	805.98	188	174.992	13.0082
atpA	MS	0.0000	0.7320	0.0000	0	963	252.127	710.873	110	110	0.000310145
atpA	GMYN	0.0000	0.9391	0.0000	0	963	238.516	724.484	110	110	9.42E-10
gyr	MS	0.0198	2.5768	0.0077	6.94E-108	894	236.303	657.697	158	154.7	3.30038
gyr	GMYN	0.0201	1.4627	0.0137	4.18E-91	894	225.047	668.953	158	144.779	13.2208
glnA	MS	0.0174	1.2335	0.0141	6.92E-87	873	232.469	640.531	140	134.776	5.22418
glnA	GMYN	0.0166	1.4398	0.0116	8.00E-87	873	201.482	671.518	140	128.951	11.0489
cit	MS	0.0055	1.8270	0.0030	0	816	267.637	548.363	146	145.107	0.892631
cit	GMYN	0.0053	1.1520	0.0046	4.48E-88	816	242.962	573.038	146	142.999	3.00097
adk	MS	0.0077	1.7370	0.0044	0	375	112.06	262.94	68	67.3013	0.698709
adk	GMYN	0.0073	1.6208	0.0045	1.83E-45	375	99.4572	275.543	68	65.9998	2.00022
ndk	MS	0.0000	0.8468	0.0000	0	225	71.2132	153.787	25	24.9999	5.40E-05
ndk	GMYN	NA	0.8193	NA	0	225	58.0371	166.963	25	25	0
			**maximum**	0.0141							
			**minimum**	0.0000							
			**average**	0.0064							
			**median**	0.0055							

Selected genes in P1-Kol8

Sequence	Method	Ka	Ks	Ka/Ks	P-Value(Fisher)	Length	S-Sites	N-Sites	Substitutions	S-Substitutions	N-Substitutions
Fimb	MS	0.0084	0.1283	0.0655	1.37E-08	510	140.079	369.921	19	16.199	2.80101
Fimb	GMYN	0.0107	0.1227	0.0876	1.54E-06	510	134.308	375.692	19	15	4
			**maximum**	0.0876							
			**minimum**	0.0655							
			**average**	0.0765							
			**median**	0.0765							

Housekeeping genes in P1-Kol8

Sequence	Method	Ka	Ks	Ka/Ks	P-Value(Fisher)	Length	S-Sites	N-Sites	Substitutions	S-Substitutions	N-Substitutions
iso	MS	NA	NA	NA	NA	NA	NA	NA	NA	NA	NA
iso	GMYN	0.0006	0.0392	0.0152	0	2232	553.663	1678.34	22	21	1
acsA	MS	0.0000	0.0000	0.8609	0	1971	487.169	1483.83	0	0	0
acsA	GMYN	0.0000	0.0000	0.0000	0	1971	503.592	1467.41	0	0	0
succ	MS	0.0000	0.0000	0.8609	0	1776	410.966	1365.03	0	0	0
succ	GMYN	0.0000	NA	NA	0	1776	437.879	1338.12	0	0	0
CTP	MS	0.0000	0.0000	0.8609	0	1659	396.751	1262.25	0	0	0
CTP	GMYN	0.0000	0.0000	NA	0	1659	418.508	1240.49	0	0	0
atpA	MS	0.0000	0.0000	0.8609	0	1539	380.541	1158.46	0	0	0
atpA	GMYN	NA	0.0000	NA	0	1539	407.726	1131.27	0	0	0
gyr	MS	0.0000	0.0000	0.8610	0	1434	378.656	1055.34	0	0	0
gyr	GMYN	0.0000	NA	NA	0	1434	387.291	1046.71	0	0	0
glnA	MS	0.0000	0.0000	0.8607	0	1413	332.453	1080.55	0	0	0
glnA	GMYN	0.0000	0.0000	NA	0	1413	350.104	1062.9	0	0	0
cit	MS	0.0000	0.0034	0.0000	0	1311	291.309	1019.69	1	0.999996	3.50E-06
cit	GMYN	0.0000	0.0032	0.0000	0	1311	312.796	998.204	1	1	0
adk	MS	0.0059	0.1633	0.0364	3.45E-13	663	160.211	502.789	25	22.4396	2.56039
adk	GMYN	0.0061	0.1459	0.0419	3.77E-11	663	170.116	492.884	25	22	3
ndk	MS	0.0000	0.0000	0.8603	0	423	97.5858	325.414	0	0	0

ndk	GMYN	NA	NA	NA	0	423	97.8687	325.131	0	0	0
			**maximum**	0.8610							
			**minimum**	0.0000							
			**average**	0.4707							
			**median**	0.8603							

Selected genes in QLW-P1DATA-2

Sequence	Method	Ka	Ks	Ka/Ks	P-Value(Fisher)	Length	S-Sites	N-Sites	Substitutions	S-Substitutions	N-Substitutions
Alkyl	MS	0.0309	2.5430	0.0121	3.46E-65	444	108.251	335.749	88	84.8045	3.19553
Alkyl	GMYN	0.0333	4.3104	0.0077	4.72E-51	444	102.89	341.11	88	76.9209	11.0791
Carbo	MS	0.0217	1.6203	0.0134	1.31E-73	639	169.63	469.37	114	109.927	4.07271
Carbo	GMYN	0.0215	1.4251	0.0151	3.55E-64	639	162.959	476.041	114	103.941	10.0589
Cold	MS	0.0000	0.0495	0.0000	0	180	40.3807	139.619	2	1.99999	6.92E-06
Cold	GMYN	0.0000	0.0516	0.0000	0	180	42.6501	137.35	2	2	0
DnaK	MS	0.0121	0.7511	0.0161	3.00E-127	1890	547.109	1342.89	238	228.969	9.03078
DnaK	GMYN	0.0116	0.9016	0.0128	2.25E-129	1890	483.897	1406.1	238	221.917	16.0828
DSBA	MS	0.1276	2.9315	0.0435	NA	603	138.561	464.439	163	142.251	20.7487
DSBA	GMYN	0.1248	3.6412	0.0343	1.15E-57	603	128.368	474.632	163	108.823	54.1775
Fatty	MS	0.0647	2.4607	0.0263	1.27E-190	1158	264.889	893.111	261	239.749	21.2512
Fatty	GMYN	0.0629	4.1281	0.0152	1.96E-134	1158	245.709	912.291	261	206.176	54.8241
Fimb	MS	0.0559	3.8195	0.0146	1.50E-86	498	124.921	373.079	113	108.266	4.73378
Fimb	GMYN	0.0562	3.6049	0.0156	4.11E-54	498	122.299	375.701	113	92.7167	20.2833
FtsK	MS	0.0150	1.0321	0.0146	5.19E-209	2268	620.198	1647.8	353	339.852	13.1481
FtsK	GMYN	0.0140	1.3271	0.0105	1.31E-194	2268	602.647	1665.35	353	329.962	23.0377
GroL	MS	0.0060	0.5348	0.0113	1.07E-88	1611	457.404	1153.6	160	155.571	4.4293
GroL	GMYN	0.0058	0.6384	0.0091	7.74E-94	1611	398.76	1212.24	160	152.999	7.00051
Piri	MS	0.1350	3.1715	0.0426	NA	675	144.253	530.747	176	152.168	23.8322
Piri	GMYN	0.1387	6.1000	0.0227	6.44E-48	675	146.142	528.858	176	109.574	66.426
RpoE	MS	NA	NA	NA	NA	NA	NA	NA	NA	NA	NA
RpoE	GMYN	0.0305	0.7767	0.0393	3.39E-31	546	136.588	409.412	74	61.7805	12.2195
RpoH	MS	0.0438	1.2207	0.0359	1.02E-82	894	254.367	639.633	162	148.596	13.4035
RpoH	GMYN	0.0413	1.2961	0.0318	6.98E-73	894	218.78	675.22	162	134.941	27.0586

Rubre	MS	0.0367	2.7185	0.0135	6.40E-50	462	147.886	314.114	88	85.5493	2.45071
Rubre	GMYN	0.0345	1.7929	0.0192	2.66E-38	462	134.721	327.279	88	77	11
RuvB	MS	0.0095	1.2635	0.0075	9.59E-112	1029	286.963	742.037	174	170.69	3.31038
RuvB	GMYN	0.0094	1.8698	0.0050	1.27E-105	1029	278.984	750.016	174	166.992	7.00753
			**maximum**	0.0435							
			**minimum**	0.0000							
			**average**	0.0181							
			**median**	0.0146							

Housekeeping genes in QLW-P1DATA-2

Sequence	Method	Ka	Ks	Ka/Ks	P-Value(Fisher)	Length	S-Sites	N-Sites	Substitutions	S-Substitutions	N-Substitutions
iso	MS	0.0223	0.9998	0.0223	1.13E-190	2232	596.507	1635.49	339	319.447	19.5526
iso	GMYN	0.0222	1.3559	0.0164	2.72E-166	2232	575.916	1656.08	339	302.855	36.1454
acsA	MS	0.0303	0.8617	0.0351	6.64E-147	1971	493.945	1477.05	281	254.288	26.7124
acsA	GMYN	0.0272	1.5565	0.0175	3.77E-141	1971	432.585	1538.41	281	239.927	41.0729
succ	MS	0.0126	0.8678	0.0145	1.08E-139	1776	459.187	1316.81	233	223.668	9.33209
succ	GMYN	0.0111	1.6003	0.0069	3.14E-141	1776	413.624	1362.38	233	218	15.0005
CTP	MS	0.0291	1.8451	0.0158	1.39E-216	1659	396.265	1262.73	309	294.203	14.7971
CTP	GMYN	0.0292	2.0363	0.0143	9.17E-174	1659	389.391	1269.61	309	272.709	36.2908
atpA	MS	0.0029	0.2471	0.0119	5.50E-48	1539	416.33	1122.67	86	83.3313	2.66866
atpA	GMYN	0.0026	0.2821	0.0092	8.33E-50	1539	378.591	1160.41	86	82.9997	3.00028
gyr	MS	0.0292	1.8620	0.0157	1.38E-164	1434	412.584	1021.42	275	264.715	10.2845
gyr	GMYN	0.0295	1.9973	0.0148	5.21E-126	1434	417.706	1016.29	275	245.663	29.3372
glnA	MS	0.0163	0.4674	0.0349	3.72E-65	1413	369.219	1043.78	136	123.8	12.2004
glnA	GMYN	0.0140	0.8743	0.0161	5.05E-67	1413	332.257	1080.74	136	120.992	15.0082
cit	MS	0.0140	0.8290	0.0169	3.17E-103	1311	318.87	992.13	168	159.61	8.38997
cit	GMYN	0.0128	2.1119	0.0061	3.95E-105	1311	281.381	1029.62	168	154.948	13.0524
adk	MS	0.0281	0.8887	0.0316	2.92E-49	663	190.603	472.397	96	89.0224	6.97763
adk	GMYN	0.0270	1.0241	0.0263	2.16E-43	663	169.874	493.126	96	82.9971	13.0029
ndk	MS	0.0029	0.4709	0.0063	0	423	87.6668	335.333	29	28.3217	0.678306
ndk	GMYN	0.0030	0.4702	0.0065	3.64E-19	423	94.1205	328.879	29	28	1
			**maximum**	0.0351							
			**minimum**	0.0061							

**average**	0.0170
**median**	0.0157

Selected genes in QLW-P1FAT50C-4

Sequence	Method	Ka	Ks	Ka/Ks	P-Value(Fisher)	Length	S-Sites	N-Sites	Substitutions	S-Substitutions	N-Substitutions
FtsK	MS	0.0125	1.0848	0.0115	8.69E-202	2202	661.527	1540.47	356	346.733	9.26654
FtsK	GMYN	0.0123	1.4236	0.0086	3.02E-189	2202	635.725	1566.27	356	336.975	19.0248
Alkyl	MS	0.0224	1.7097	0.0131	9.32E-46	363	80.6686	282.331	60	57.3708	2.62924
Alkyl	GMYN	0.0258	2.3727	0.0109	8.14E-33	363	86.3952	276.605	60	52.9968	7.00321
Carbo	MS	0.0266	2.0222	0.0131	4.16E-79	558	127.269	430.731	104	99.5746	4.42543
Carbo	GMYN	0.0295	1.6056	0.0183	9.12E-56	558	138.878	419.122	104	91.9133	12.0867
Cold	MS	0.0000	0.0816	0.0000	0	99	24.5139	74.4861	2	1.99999	6.08E-06
Cold	GMYN	0.0000	0.0818	0.0000	0	99	28.0974	70.9026	2	2	0
DnaK	MS	0.0109	0.7159	0.0152	2.12E-122	1824	524.785	1299.22	226	217.802	8.19835
DnaK	GMYN	0.0102	1.6663	0.0061	1.63E-134	1824	431.263	1392.74	226	211.953	14.0469
DSBA	MS	0.1384	3.8109	0.0363	NA	522	122.466	399.534	153	136.792	16.208
DSBA	GMYN	0.1401	3.6040	0.0389	3.66E-50	522	122.167	399.833	153	102.162	50.8375
Fatty	MS	0.0597	2.1779	0.0274	6.46E-171	1092	249.207	842.793	240	219.643	20.3566
Fatty	GMYN	0.0590	4.0728	0.0145	2.00E-127	1092	228.241	863.759	240	191.199	48.8012
Fimb	MS	0.0307	3.5496	0.0087	3.34E-64	417	114.861	302.139	91	88.974	2.02604
Fimb	GMYN	0.0311	3.0580	0.0102	4.80E-51	417	110.971	306.029	91	81.8172	9.18276
GroL	MS	0.0099	0.6139	0.0162	9.83E-92	1545	435.919	1109.08	169	162.326	6.67375
GroL	GMYN	0.0095	0.7438	0.0128	2.28E-94	1545	378.873	1166.13	169	157.998	11.0017
Hfq	MS	0.0309	0.5873	0.0527	2.46E-08	144	42.0409	101.959	18	15.9613	2.0387
Hfq	GMYN	0.0292	0.7239	0.0403	7.47E-08	144	37.722	106.278	18	15	3
Hyp	MS	0.0329	4.2779	0.0077	0	162	36.9056	125.094	33	32.1607	0.839256
Hyp	GMYN	0.0335	3.0362	0.0110	3.22E-19	162	39.3534	122.647	33	29	4
Piri	MS	0.1626	3.5973	0.0452	NA	594	144.815	449.185	176	154.353	21.647
Piri	GMYN	0.1614	3.6715	0.0440	4.43E-49	594	133.658	460.342	176	109.705	66.2949
RpoE	MS	0.0224	0.8582	0.0261	3.76E-38	465	103.751	361.249	62	56.8306	5.16941
RpoE	GMYN	0.0228	1.1131	0.0205	3.71E-32	465	106.014	358.986	62	53.9583	8.04168
RpoH	MS	0.0333	1.2678	0.0263	3.24E-79	822	231.935	590.065	145	135.908	9.09161

RpoH	GMYN	0.0309	1.6432	0.0188	1.47E-78	822	188.964	633.036	145	125.878	19.1225
Rubre	MS	0.0362	3.0609	0.0118	1.38E-42	381	121.404	259.596	72	70.2252	1.7748
Rubre	GMYN	0.0357	1.0941	0.0326	2.11E-29	381	122.203	258.797	72	63	9
RuvB	MS	0.0161	1.5556	0.0104	3.38E-115	963	272.316	690.684	180	175.384	4.61607
RuvB	GMYN	0.0161	1.3747	0.0117	1.25E-101	963	268.211	694.789	180	168.983	11.0174
Superox	MS	0.0476	2.2384	0.0213	3.12E-56	429	118.796	310.204	89	84.3182	4.68179
Superox	GMYN	0.0486	2.4670	0.0197	4.26E-34	429	128.64	300.36	89	74.9082	14.0918
			**maximum**	0.0527							
			**minimum**	0.0000							
			**average**	0.0195							
			**median**	0.0148							

Housekeeping genes in QLW-P1FAT50C-4

Sequence	Method	Ka	Ks	Ka/Ks	P-Value(Fisher)	Length	S-Sites	N-Sites	Substitutions	S-Substitutions	N-Substitutions
iso	MS	0.0194	0.9274	0.0209	3.43E-180	2232	612.155	1619.84	327	309.838	17.1624
iso	GMYN	0.0190	1.2797	0.0149	1.91E-167	2232	569.193	1662.81	327	295.802	31.1981
acsA	MS	0.0254	0.9576	0.0265	4.92E-162	1971	532.638	1438.36	299	279	19.9996
acsA	GMYN	0.0241	2.8319	0.0085	2.40E-161	1971	444.845	1526.16	299	262.838	36.1616
succ	MS	0.0228	0.7962	0.0287	6.53E-126	1776	506.432	1269.57	250	233.227	16.7728
succ	GMYN	0.0216	1.7305	0.0125	1.09E-131	1776	408.645	1367.35	250	220.971	29.029
CTP	MS	0.0287	1.4080	0.0204	5.77E-183	1659	410.376	1248.62	288	271.188	16.812
CTP	GMYN	0.0287	1.3198	0.0218	1.33E-147	1659	407.071	1251.93	288	252.764	35.2364
atpA	MS	0.0069	0.3281	0.0210	2.25E-58	1539	414.089	1124.91	111	105.009	5.99135
atpA	GMYN	0.0063	0.3240	0.0194	4.47E-54	1539	420.3	1118.7	111	104	7
gyr	MS	0.0294	2.9137	0.0101	9.18E-210	1434	389.71	1044.29	302	294.044	7.95621
gyr	GMYN	0.0295	2.2954	0.0128	1.71E-164	1434	379.947	1054.05	302	271.606	30.3935
glnA	MS	0.0009	0.3288	0.0029	0	1413	376.781	1036.22	93	92.2678	0.732182
glnA	GMYN	0.0010	0.3391	0.0028	1.14E-57	1413	360.354	1052.65	93	91.9999	1.00008
cit	MS	0.0228	0.6920	0.0329	1.20E-84	1311	342.568	968.432	167	152.781	14.2187
cit	GMYN	0.0225	0.9200	0.0244	3.22E-78	1311	316.177	994.823	167	144.987	22.0131
adk	MS	0.0266	1.0241	0.0260	1.51E-54	663	196.497	466.503	103	97.0207	5.97934
adk	GMYN	0.0245	1.9126	0.0128	6.89E-54	663	159.511	503.489	103	90.8916	12.1084
ndk	MS	0.0097	0.4362	0.0222	9.09E-21	423	116.198	306.802	36	34.0073	1.99266

ndk	GMYN	0.0093	0.5018	0.0186	4.30E-20	423	98.377	324.623	36	33	3
			**maximum**	0.0329							
			**minimum**	0.0028							
			**average**	0.0180							
			**median**	0.0199							

Selected genes in STIR1

Sequence	Method	Ka	Ks	Ka/Ks	P-Value(Fisher)	Length	S-Sites	N-Sites	Substitutions	S-Substitutions	N-Substitutions
Alkyl	MS	0.0374733	4.36149	0.00859187	4.13E-71	387	86.1463	300.854	81	78.6403	2.35967
Alkyl	GMYN	0.0421388	3.35097	0.0125751	2.70E-46	387	87.1785	299.822	81	68.7847	12.2153
Carbo	MS	NA	NA	NA	NA	NA	NA	NA	NA	NA	NA
Carbo	GMYN	0.0806866	3.5869	0.0224948	5.76E-63	522	119.406	402.594	133	102.329	30.6713
Cold	MS	5.04E-08	0.0504104	1.00E-06	0	195	65.3257	129.674	3	2.99999	5.96E-06
Cold	GMYN	1.11E-15	0.0675801	1.64E-14	0	195	47.762	147.238	3	3	0
DnaK	MS	0.0176324	0.752496	0.0234318	2.69E-118	1764	495.011	1268.99	226	213.194	12.8063
DnaK	GMYN	0.0162898	1.39952	0.0116396	1.41E-128	1764	390.355	1373.64	226	203.891	22.1088
Fatty	MS	0.0686111	1.57088	0.0436769	1.40E-131	1050	263.549	786.451	231	204.364	26.6359
Fatty	GMYN	0.0674697	2.72085	0.0247973	1.48E-97	1050	245.528	804.472	231	179.253	51.7474
FtsK	MS	0.0186167	1.07178	0.0173699	9.31E-196	2142	623.939	1518.06	352	337.727	14.2728
FtsK	GMYN	0.0191907	1.01163	0.01897	1.43E-159	2142	655.893	1486.11	352	323.879	28.1214
GroL	MS	0.0132982	0.594931	0.0223525	7.80E-85	1494	418.369	1075.63	163	154.142	8.85828
GroL	GMYN	0.0126462	0.531829	0.0237787	2.22E-69	1494	456.241	1037.76	163	149.998	13.0021
Hfq	MS	0.0118487	0.954838	0.0124092	2.40E-20	234	61.682	172.318	32	30.9278	1.07217
Hfq	GMYN	0.0112376	2.02923	0.00553787	4.09E-21	234	53.0886	180.911	32	29.9916	2.00839
RpoE	MS	0.142481	3.63299	0.0392187	NA	426	95.0634	330.937	111	97.6658	13.3342
RpoE	GMYN	0.150827	1.68973	0.0892605	1.74E-24	426	99.2193	326.781	111	66.5945	44.4055
RpoH	MS	0.0368315	1.0868	0.03389	3.08E-69	780	218.821	561.179	133	122.365	10.6351
RpoH	GMYN	0.0360859	1.24944	0.0288816	5.77E-56	780	209.234	570.766	133	112.938	20.062
RuvB	MS	0.0222919	2.61062	0.00853893	3.84E-144	921	191.99	729.01	169	163.693	5.30748
RuvB	GMYN	0.0233258	1.98957	0.0117241	1.43E-100	921	219.415	701.585	169	152.967	16.0331
			**maximum**	0.0893							
			**minimum**	0.0000							

**average**	0.0219
**median**	0.0190

Housekeeping genes in STIR1

Sequence	Method	Ka	Ks	Ka/Ks	P-Value(Fisher)	Length	S-Sites	N-Sites	Substitutions	S-Substitutions	N-Substitutions
iso	MS	0.0246	0.8577	0.0286	5.64E-158	2148	607.868	1540.13	313	291.834	21.166
iso	GMYN	0.0244	0.8963	0.0272	3.15E-134	2148	596.948	1551.05	313	275.79	37.2096
acsA	MS	0.0365	0.9353	0.0390	4.32E-149	1887	507.18	1379.82	300	271.218	28.782
acsA	GMYN	0.0356	1.4655	0.0243	3.08E-127	1887	469.867	1417.13	300	250.775	49.2249
atpA	MS	NA	NA	NA	NA	NA	NA	NA	NA	NA	NA
atpA	GMYN	0.0118	0.3687	0.0320	5.77E-48	1470	357.029	1112.97	107	94	13
gyr	MS	0.0484	3.6212	0.0134	1.86E-215	1365	363.563	1001.44	305	294.174	10.8262
gyr	GMYN	0.0496	2.0975	0.0237	2.78E-138	1365	365.201	999.799	305	257.189	47.8108
glnA	MS	0.0110	0.4696	0.0234	9.82E-66	1344	344.318	999.682	122	114.235	7.76513
glnA	GMYN	0.0107	0.5838	0.0184	1.10E-64	1344	311.139	1032.86	122	110.994	11.0057
cit	MS	0.0190	0.6864	0.0277	1.37E-78	1242	345.667	896.333	155	144.602	10.3981
cit	GMYN	0.0175	1.6527	0.0106	2.62E-89	1242	259.875	982.125	155	137.996	17.0036
adk	MS	0.0336	1.2359	0.0272	4.20E-55	594	152.53	441.47	94	87.1361	6.86386
adk	GMYN	0.0320	0.8913	0.0359	2.95E-37	594	172.334	421.666	94	80.8497	13.1503
ndk	MS	0.0108	0.3335	0.0325	8.68E-12	354	84.6678	269.332	22	19.9386	2.06145
ndk	GMYN	0.0112	0.2822	0.0398	1.89E-10	354	83.7145	270.285	22	19	3
			**maximum**	0.0398							
			**minimum**	0.0106							
			**average**	0.0269							
			**median**	0.0272							

Selected genes in Tro8F10W22

Sequence	Method	Ka	Ks	Ka/Ks	P-Value(Fisher)	Length	S-Sites	N-Sites	Substitutions	S-Substitutions	N-Substitutions
GroL	MS	0.0009	0.0000	50.0000	0	1650	524.594	1125.41	1	0.00923665	0.990763
GroL	GMYN	0.0008	0.0000	2.5E+11	0	1650	428.744	1221.26	1	0	1
Alkyl	MS	0.0032	0.0730	0.0439	0	465	149.252	315.748	11	10.0655	0.93448

Alkyl	GMYN	0.0030	0.0841	0.0356	1.76E-05	465	129.538	335.462	11	10	1
Fimb	MS	0.0359	0.6772	0.0530	3.91E-30	510	134.073	375.927	66	57.4616	8.53837
Fimb	GMYN	0.0355	0.7096	0.0500	8.03E-24	510	131.263	378.737	66	52.9318	13.0682
RpoE	MS	0.0000	0.0076	0.0000	0	567	131.79	435.21	1	0.999997	3.30E-06
RpoE	GMYN	0.0000	0.0071	0.0000	0	567	142.392	424.608	1	1	0
Superox	MS	0.0034	0.0001	50.0000	0	531	235.19	295.81	1	0.0156525	0.984347
Superox	GMYN	0.0025	NA	NA	0	531	130.855	400.145	1	0	1
			**maximum**	2.5E+11							
			**minimum**	0.0000							
			**average**	2.7E+10							
			**median**	0.0500							

Housekeeping genes in Tro8F10W22

Sequence	Method	Ka	Ks	Ka/Ks	P-Value(Fisher)	Length	S-Sites	N-Sites	Substitutions	S-Substitutions	N-Substitutions
iso	MS	0.0000	0.0000	0.8609	0	2232	517.539	1714.46	0	0	0
iso	GMYN	NA	0.0000	NA	0	2232	555.654	1676.35	0	0	0
acsA	MS	0.0007	0.0017	0.4511	0	1971	626.959	1344.04	2	1.0167	0.983301
acsA	GMYN	0.0007	0.0020	0.3417	0.38025	1971	502.821	1468.18	2	1	1
succ	MS	0.0000	0.0000	0.8609	0	1776	410.966	1365.03	0	0	0
succ	GMYN	0.0000	NA	NA	0	1776	437.879	1338.12	0	0	0
CTP	MS	0.0000	0.0000	0.8609	0	1659	396.751	1262.25	0	0	0
CTP	GMYN	0.0000	0.0000	NA	0	1659	418.508	1240.49	0	0	0
atpA	MS	0.0010	0.0058	0.1735	0	1539	533.218	1005.78	4	3.01356	0.986439
atpA	GMYN	0.0009	0.0074	0.1194	0.0544848	1539	407.728	1131.27	4	3	1
gyr	MS	0.0000	0.0000	0.8610	0	1434	378.656	1055.34	0	0	0
gyr	GMYN	0.0000	NA	NA	0	1434	387.291	1046.71	0	0	0
glnA	MS	0.0000	0.0000	0.8607	0	1413	332.453	1080.55	0	0	0
glnA	GMYN	0.0000	0.0000	NA	0	1413	350.104	1062.9	0	0	0
cit	MS	0.0000	0.0034	0.0000	0	1311	291.309	1019.69	1	0.999996	3.50E-06
cit	GMYN	0.0000	0.0032	0.0000	0	1311	312.796	998.204	1	1	0
adk	MS	0.0079	0.1992	0.0397	6.71E-15	663	159.472	503.528	30	26.6596	3.34038
adk	GMYN	0.0078	0.2078	0.0375	1.59E-14	663	146.132	516.868	30	26	4
ndk	MS	0.0000	0.0102	0.0000	0	423	98.1022	324.898	1	0.999997	3.31E-06

ndk	GMYN	0.0000	0.0103	0.0000	0	423	98.4044	324.596	1	1	0
			**maximum**	0.8610							
			**minimum**	0.0000							
			**average**	0.3645							
			**median**	0.1735							

GMYN = Approximate method (γ-MYN)
MS = Maximum-likelihood method

Supplementary Table S10. Ka/Ks analysis of highly expressed genes and housekeeping genes in selected strains of genus *Polynucleobacter*. Ka/Ks values were calculated twice; once by approximate method (γ-MYN) and then using a maximum-likelihood method (MS, i.e. the substitution model with the best score was selected).

Supplementary Table S11.

Genomes	Ka	Ks	Ka/Ks	Length	S-Sites	N-Sites	Substitutions	S-Substitutions	N-Substitutions
QLW-P1DMWA-1 vs. MWH-Tro7-1-4	0.00195056	0.023324	0.083629	1706745	454441	1.25E+06	12836	10396.8	2439.17
P1-4-10KL vs. MWH-Tro7-1-4	0.00196293	0.0232851	0.0842999	1706853	455223	1.25E+06	12851	10397.7	2453.29
P1-Kol8 vs. MWH-Tro7-1-4	0.00198399	0.0234529	0.0845945	1706637	457937	1.25E+06	13007	10533.2	2473.75
MWH-Recht1 vs. MWH-Tro7-1-4	0.00221659	0.0258409	0.0857784	1706475	453292	1.25E+06	14240	11466.7	2773.26
MWH-Tro7-1-4 vs. Tro8F10W22	0.00244588	0.0284838	0.0858691	1705725	455481	1.25E+06	15724	12671.6	3052.41
MWH-Tro7-1-4 vs. MWH-Tro8-2-9	0.00202447	0.023507	0.0861219	1703907	455736	1.25E+06	13029	10505.9	2523.1
P1-Kol8 vs. MWH-Recht1	0.00104664	0.0121318	0.086273	1707705	453078	1.25E+06	6754	5441.87	1312.13
QLW-P1DMWA-1 vs. P1-Kol8	0.000725487	0.00834921	0.0868929	1707858	457364	1.25E+06	4699	3792.27	906.726
QLW-P1DMWA-1 vs. MWH-Recht1	0.0012077	0.0138429	0.0872431	1707726	449003	1.26E+06	7664	6145.19	1518.81
MWH-Recht1 vs. Tro8F10W22	0.00249435	0.0284729	0.0876046	1705869	456741	1.25E+06	15813	12702.9	3110.06
P1-4-10KL vs. MWH-Recht1	0.0012184	0.01381	0.0882259	1707789	450122	1.26E+06	7677	6146.03	1530.97
P1-Kol8 vs. P1-4-10KL	0.000736141	0.00831726	0.0885076	1707975	459092	1.25E+06	4711	3792.15	918.849
MWH-Recht1 vs. MWH-Tro8-2-9	0.00144906	0.0162686	0.0890708	1704606	459228	1.25E+06	9174	7371.3	1802.7
P1-Kol8 vs. Tro8F10W22	0.00238612	0.0264698	0.0901451	1705965	457107	1.25E+06	14813	11838.3	2974.69
QLW-P1DMWA-1 vs. Tro8F10W22	0.00257755	0.0283585	0.0908917	1706103	455977	1.25E+06	15849	12632.8	3216.15
P1-4-10KL vs. Tro8F10W22	0.00258822	0.0283283	0.0913654	1706166	456483	1.25E+06	15862	12633.7	3228.3
MWH-Tro8-2-9 vs. Tro8F10W22	0.0022336	0.0243685	0.0916592	1704483	454515	1.25E+06	13644	10856.6	2787.36
MWH-RechtKol4 vs. MWH-RechtKolB	8.93E-05	0.000974467	0.0916854	1704036	487083	1.22E+06	583	474.28	108.72
MWH-RechtKol4 vs. Tro8F10W22	0.00658013	0.0696538	0.094469	1701204	449438	1.25E+06	37776	29578.9	8197.09
MWH-RechtKolB vs. Tro8F10W22	0.00654865	0.0691745	0.0946685	1701531	449685	1.25E+06	37560	29401.4	8158.59
QLW-P1DMWA-1 vs. MWH-RechtKol4	0.00588615	0.0617279	0.0953563	1702494	450910	1.25E+06	33797	26461.8	7335.17
P1-4-10KL vs. MWH-RechtKol4	0.00589486	0.0617132	0.0955202	1702494	451027	1.25E+06	33808	26462.7	7345.28
QLW-P1DMWA-1 vs. MWH-RechtKolB	0.00585996	0.0612638	0.0956513	1702890	451304	1.25E+06	33597	26294.3	7302.7
P1-4-10KL vs. MWH-RechtKolB	0.00586868	0.0612441	0.0958245	1702890	451423	1.25E+06	33606	26293.2	7312.81
MWH-Recht1 vs. MWH-RechtKol4	0.00601424	0.0626273	0.0960321	1702197	450690	1.25E+06	34310	26816.3	7493.71
MWH-Recht1 vs. MWH-RechtKolB	0.00598426	0.0621824	0.0962372	1702593	450975	1.25E+06	34108	26650.8	7457.18
P1-Kol8 vs. MWH-RechtKol4	0.00587588	0.0608286	0.0965973	1702464	450401	1.25E+06	33393	26067.7	7325.28
P1-Kol8 vs. MWH-RechtKolB	0.00584633	0.0603935	0.0968039	1702755	450666	1.25E+06	33193	25904.3	7288.75
MWH-RechtKol4 vs. MWH-Tro8-2-9	0.00604144	0.061833	0.0977057	1699644	451725	1.25E+06	34061	26555.2	7505.84
MWH-RechtKolB vs. MWH-Tro8-2-9	0.00601881	0.0612945	0.098195	1699956	452263	1.25E+06	33842	26365.5	7476.5
MWH-RechtKol4 vs. MWH-Tro7-1-4	0.00594435	0.0598772	0.0992758	1701645	448774	1.25E+06	33005	25589.9	7415.11

MWH-RechtKolB vs. MWH-Tro7-1-4	0.00591963	0.0594336	0.0996007	1702062	449149	1.25E+06	32814	25429.4	7384.65
QLW-P1DMWA-1 vs. MWH-Tro8-2-9	0.00125452	0.0125479	0.0999781	1705017	462707	1.24E+06	7303	5745.94	1557.06
P1-Kol8 vs. MWH-Tro8-2-9	0.0011471	0.0114233	0.100418	1705023	466364	1.24E+06	6697	5277.34	1419.66
P1-4-10KL vs. MWH-Tro8-2-9	0.00126523	0.0125232	0.101031	1705080	463678	1.24E+06	7316	5746.8	1569.2
		**Median**	**0.0916854**						

Ka = Numbers of substitutions per nonsynonymous site

Ks = Numbers of substitutions per synonymous site

Supplementary Table S11. Ka/Ks analysis among the nine strains of *Polynucleobacter asymbioticus* using their whole core genomes.

Supplementary Table S12.

Selected genes in *Polynucleobacter asymbioticus* strain QLW-P1DMWA-1[T]

Sequence	Method	Ka	Ks	Ka/Ks	P-Value(Fisher)	Length	S-Sites	N-Sites	Substitutions	S-Substitutions	N-Substitutions
DSBA	MS	0.0069	0.0285	0.2402	0.0100676	624	184.867	439.133	8	5.09395	2.90605
DSBA	MS	0.0069	0.0285	0.2402	0.0100676	624	184.867	439.133	8	5.09395	2.90605
DSBA	GMYN	0.0065	0.0321	0.2029	0.0295253	624	160.064	463.936	8	5	3
DSBA	GMYN	0.0065	0.0321	0.2029	0.0295253	624	160.064	463.936	8	5	3
RpoE	GMYN	0.0047	0.0289	0.1636	0.0042709	567	142.236	424.764	6	4	2
RpoE	GMYN	0.0047	0.0289	0.1636	0.0042709	567	142.236	424.764	6	4	2
Superox	MS	0.0083	0.0606	0.1365	0.000221587	531	163.432	367.568	12	9.18071	2.81929
RpoE	MS	0.0045	0.0336	0.1354	0.00299834	567	132.04	434.96	6	4.14927	1.85073
RpoE	MS	0.0045	0.0336	0.1354	0.00299834	567	132.04	434.96	6	4.14927	1.85073
Fimb	MS	0.0055	0.0540	0.1012	0.00017699	510	139.271	370.729	9	7.09043	1.90957
Superox	GMYN	0.0075	0.0772	0.0974	0.00026245	531	129.114	401.886	12	9	3
Fimb	GMYN	0.0054	0.0552	0.0972	0.00180129	510	135.493	374.507	9	7	2
Fimb	GMYN	0.0107	0.1227	0.0876	1.54E-06	510	134.308	375.692	19	15	4
Fimb	GMYN	0.0107	0.1371	0.0777	2.66E-07	510	129.6	380.4	20	16	4
Fimb	GMYN	0.0107	0.1371	0.0777	2.66E-07	510	129.6	380.4	20	16	4
FtsK	MS	0.0024	0.0345	0.0709	4.98E-10	2310	682.337	1627.66	26	22.2388	3.76118
FtsK	MS	0.0024	0.0345	0.0709	4.98E-10	2310	682.337	1627.66	26	22.2388	3.76118
Alkyl	MS	0.0031	0.0443	0.0708	0	465	144.282	320.718	7	6.04791	0.952093
Alkyl	MS	0.0031	0.0443	0.0708	0	465	144.282	320.718	7	6.04791	0.952093
FtsK	GMYN	0.0024	0.0362	0.0657	1.18E-09	2310	625.81	1684.19	26	22	4
FtsK	GMYN	0.0024	0.0362	0.0657	1.18E-09	2310	625.81	1684.19	26	22	4
Fimb	MS	0.0084	0.1283	0.0655	1.37E-08	510	140.079	369.921	19	16.199	2.80101
Fimb	MS	0.0086	0.1329	0.0650	9.77E-09	510	147.434	362.566	20	17.2435	2.75652
Fimb	MS	0.0086	0.1329	0.0650	9.77E-09	510	147.434	362.566	20	17.2435	2.75652
Methyl	MS	0.0754	1.1880	0.0635	1.18E-128	1470	350.71	1119.29	276	229.493	46.5069
Alkyl	GMYN	0.0029	0.0532	0.0549	0.00146005	465	120.669	344.331	7	6	1
Alkyl	GMYN	0.0029	0.0532	0.0549	0.00146005	465	120.669	344.331	7	6	1
Fimb	MS	0.0359	0.6772	0.0530	3.91E-30	510	134.073	375.927	66	57.4616	8.53837

Fimb	GMYN	0.0355	0.7096	0.0500	8.03E-24	510	131.263	378.737	66	52.9318	13.0682
Alkyl	MS	0.0032	0.0730	0.0439	0	465	149.252	315.748	11	10.0655	0.93448
Alkyl	GMYN	0.0030	0.0841	0.0356	1.76E-05	465	129.538	335.462	11	10	1
Methyl	GMYN	0.0697	2.3825	0.0292	7.38E-89	1470	348.153	1121.85	276	201.683	74.3171
RuvB	MS	0.0013	0.0715	0.0187	0	1068	318.884	749.116	22	21.0732	0.926773
RuvB	MS	0.0013	0.0715	0.0187	0	1068	318.884	749.116	22	21.0732	0.926773
RuvB	GMYN	0.0013	0.0836	0.0150	2.76E-12	1068	270.522	797.478	22	21	1
RuvB	GMYN	0.0013	0.0836	0.0150	2.76E-12	1068	270.522	797.478	22	21	1
MscS	MS	0.0000	0.0437	0.0000	0	819	216.856	602.144	9	8.99998	2.50E-05
MscS	MS	0.0000	0.0437	0.0000	0	819	216.856	602.144	9	8.99998	2.50E-05

Housekeeping genes in *Polynucleobacter asymbioticus* strain QLW-P1DMWA-1T

Sequence	Method	Ka	Ks	Ka/Ks	P-Value(Fisher)	Length	S-Sites	N-Sites	Substitutions	S-Substitutions	N-Substitutions
atpA	MS	0.0010	0.0058	0.1735	0	1539	533.218	1005.78	4	3.01356	0.986439
cit	GMYN	0.0021	0.0160	0.1290	0.0101498	1311	312.415	998.585	7	4.9408	2.0592
cit	GMYN	0.0021	0.0160	0.1290	0.0101498	1311	312.415	998.585	7	4.9408	2.0592
CTP	MS	0.0018	0.0190	0.0931	7.22E-05	1659	525.286	1133.71	11	9.15969	1.84031
CTP	MS	0.0018	0.0190	0.0931	7.22E-05	1659	525.286	1133.71	11	9.15969	1.84031
CTP	GMYN	0.0016	0.0221	0.0730	0.000125077	1659	417.189	1241.81	11	9	2
CTP	GMYN	0.0016	0.0221	0.0730	0.000125077	1659	417.189	1241.81	11	9	2
cit	MS	0.0016	0.0234	0.0684	0.000382964	1311	267.118	1043.88	7	5.52364	1.47636
cit	MS	0.0016	0.0234	0.0684	0.000382964	1311	267.118	1043.88	7	5.52364	1.47636
adk	GMYN	0.0090	0.1475	0.0609	5.40E-11	663	215.007	447.993	32	28	4
adk	GMYN	0.0090	0.1475	0.0609	5.40E-11	663	215.007	447.993	32	28	4
adk	MS	0.0083	0.1850	0.0449	2.00E-14	663	181.819	481.181	32	28.5993	3.40068
adk	MS	0.0083	0.1850	0.0449	2.00E-14	663	181.819	481.181	32	28.5993	3.40068
adk	GMYN	0.0061	0.1459	0.0419	3.77E-11	663	170.116	492.884	25	22	3
acsA	GMYN	0.0028	0.0699	0.0399	1.30E-16	1971	530.919	1440.08	39	35	4
acsA	GMYN	0.0028	0.0699	0.0399	1.30E-16	1971	530.919	1440.08	39	35	4
adk	MS	0.0079	0.1992	0.0397	6.71E-15	663	159.472	503.528	30	26.6596	3.34038
adk	MS	0.0079	0.1992	0.0397	6.71E-15	663	159.472	503.528	30	26.6596	3.34038
acsA	MS	0.0028	0.0711	0.0392	1.51E-17	1971	547.583	1423.42	39	35.392	3.60804

acsA	MS	0.0028	0.0711	0.0392	1.51E-17	1971	547.583	1423.42	39	35.392	3.60804
adk	GMYN	0.0078	0.2078	0.0375	1.59E-14	663	146.132	516.868	30	26	4
adk	GMYN	0.0078	0.2078	0.0375	1.59E-14	663	146.132	516.868	30	26	4
adk	MS	0.0059	0.1633	0.0364	3.45E-13	663	160.211	502.789	25	22.4396	2.56039
acsA	GMYN	0.0007	0.0222	0.0307	2.52E-06	1971	503.811	1467.19	12	11	1
acsA	MS	0.0007	0.0247	0.0269	0	1971	486.858	1484.14	12	11.0915	0.908467
iso	MS	0.0006	0.0236	0.0252	0	2232	561.142	1670.86	14	13.0219	0.978057
iso	GMYN	0.0006	0.0239	0.0250	1.35E-07	2232	555.944	1676.06	14	13	1
iso	GMYN	0.0019	0.0891	0.0216	4.46E-26	2232	670.713	1561.29	58	54.9983	3.0017
iso	GMYN	0.0019	0.0891	0.0216	4.46E-26	2232	670.713	1561.29	58	54.9983	3.0017
iso	MS	0.0019	0.0942	0.0201	1.10E-28	2232	646.227	1585.77	58	55.2741	2.72588
iso	MS	0.0019	0.0942	0.0201	1.10E-28	2232	646.227	1585.77	58	55.2741	2.72588
iso	GMYN	0.0006	0.0392	0.0152	0	2232	553.663	1678.34	22	21	1
gyr	MS	0.0000	0.0079	0.0000	0	1434	378.849	1055.15	3	2.99999	8.36E-06
gyr	MS	0.0000	0.0079	0.0000	0	1434	378.849	1055.15	3	2.99999	8.36E-06
atpA	MS	0.0000	0.0096	0.0000	0	1539	533.542	1005.46	5	4.99999	9.42E-06
atpA	MS	0.0000	0.0096	0.0000	0	1539	533.542	1005.46	5	4.99999	9.42E-06
gyr	MS	0.0000	0.0144	0.0000	0	1434	430.428	1003.57	6	5.99999	1.40E-05
glnA	MS	0.0000	0.0247	0.0000	0	1413	393.521	1019.48	9	8.99998	2.33E-05
glnA	MS	0.0000	0.0247	0.0000	0	1413	393.521	1019.48	9	8.99998	2.33E-05
succ	MS	0.0000	0.0366	0.0000	0	1776	430.6	1345.4	15	15	4.69E-05
succ	MS	0.0000	0.0366	0.0000	0	1776	430.6	1345.4	15	15	4.69E-05
atpA	GMYN	0.0000	0.0124	0.0000	0	1539	407.975	1131.02	5	5	0
atpA	GMYN	0.0000	0.0124	0.0000	0	1539	407.975	1131.02	5	5	0
glnA	GMYN	0.0000	0.0264	0.0000	0	1413	348.816	1064.18	9	9	0
glnA	GMYN	0.0000	0.0264	0.0000	0	1413	348.816	1064.18	9	9	0
succ	GMYN	0.0000	0.0369	0.0000	0	1776	421.093	1354.91	15	15	0
succ	GMYN	0.0000	0.0369	0.0000	0	1776	421.093	1354.91	15	15	0

Selected genes in PnecC subcluster

Sequence	Method	Ka	Ks	Ka/Ks	P-Value(Fisher)	Length	S-Sites	N-Sites	Substitutions	S-Substitutions	N-Substitutions
Cold	MS	0.0587	0.3859	0.1520	0.000282898	126	33.8772	92.1228	14	9.90516	4.09484

Cold	GMYN	0.0555	0.3986	0.1392	0.000979928	126	31.0745	94.9255	14	9	5
RpoE	GMYN	0.150827	1.68973	0.0892605	1.74E-24	426	99.2193	326.781	111	66.5945	44.4055
Hyp	GMYN	0.1130	1.3727	0.0823	6.17E-13	258	67.9994	190.001	56	36.1982	19.8018
DSBA	MS	0.2520	3.4307	0.0735	NA	591	139.069	451.931	224	180.83	43.1698
DSBA	GMYN	0.2600	3.6288	0.0717	2.50E-59	591	126.273	464.727	224	122.845	101.155
Hyp	GMYN	0.0627	0.9219	0.0680	5.24E-13	243	70.2652	172.735	43	32.7181	10.2819
DSBA	GMYN	0.2308	3.5685	0.0647	4.05E-59	543	116.507	426.493	197	113.199	83.8006
DSBA	MS	0.2287	3.5739	0.0640	NA	543	125.194	417.806	197	162.327	34.6731
DSBA	GMYN	0.2311	3.6198	0.0638	1.92E-67	600	124.76	475.24	217	123.477	93.5227
RpoE	GMYN	0.0408	0.6439	0.0634	2.68E-22	489	157.596	331.404	74	60.869	13.131
DSBA	MS	0.2284	3.7082	0.0616	NA	600	133.047	466.953	217	178.435	38.5649
Hyp	MS	0.1081	1.7632	0.0613	3.83E-30	258	63.4016	194.598	56	47.1288	8.87121
Hfq	MS	0.0330	0.5506	0.0598	2.25E-12	228	67.352	160.648	28	24.5025	3.49753
DSBA	GMYN	0.2140	3.6227	0.0591	3.91E-49	594	125.244	468.756	197	110.641	86.3591
DSBA	GMYN	0.2078	3.5746	0.0581	2.10E-54	609	117.458	491.542	197	108.721	88.2793
DSBA	MS	0.2138	3.7619	0.0568	NA	594	131.622	462.378	197	164.216	32.7843
Piri	GMYN	0.1515	2.7019	0.0561	4.72E-47	666	167.49	498.51	190	122.194	67.8064
Piri	GMYN	0.2025	3.7300	0.0543	1.38E-49	663	144.516	518.484	212	120.756	91.2439
DSBA	MS	0.2109	3.8950	0.0541	NA	609	130.442	478.558	197	164.351	32.6492
Piri	MS	0.2023	3.8314	0.0528	NA	663	145.735	517.265	212	178.54	33.4597
Hfq	MS	0.0309	0.5873	0.0527	2.46E-08	144	42.0409	101.959	18	15.9613	2.0387
Hyp	MS	0.0620	1.2128	0.0511	5.61E-22	243	68.3067	174.693	43	38.0284	4.97162
Piri	MS	0.1090	2.1400	0.0509	4.45E-100	669	150.938	518.062	160	136.19	23.8101
MscS	MS	0.1795	3.6195	0.0496	NA	774	184.613	589.387	249	214.968	34.0321
MscS	MS	0.1391	2.8398	0.0490	5.40E-147	789	183.979	605.021	212	182.586	29.4139
RpoE	MS	0.0421	0.8878	0.0475	9.16E-42	522	130.159	391.841	83	72.6232	10.3768
Piri	MS	0.1708	3.6202	0.0472	NA	684	164.432	519.568	208	181.022	26.9781
Superox	GMYN	0.0771	1.6481	0.0468	8.99E-32	471	123.061	347.939	99	73.6828	25.3172
RpoE	GMYN	0.0343	0.7338	0.0467	6.69E-29	537	138.458	398.542	74	60.6677	13.3323
DSBA	GMYN	0.1667	3.6609	0.0455	1.10E-55	618	131.796	486.204	187	114.925	72.0753
MscS	GMYN	0.1792	3.9383	0.0455	4.08E-65	774	190.775	583.225	249	157.243	91.7568
GroL	MS	0.0315	0.6930	0.0454	1.64E-95	1614	436.828	1177.17	210	187.113	22.8874
Piri	MS	0.1626	3.5973	0.0452	NA	594	144.815	449.185	176	154.353	21.647
Rubre	GMYN	0.0745	1.6575	0.0449	1.76E-33	405	106.147	298.853	92	70.8764	21.1236

Piri	MS	0.1562	3.5478	0.0440	NA	615	153.004	461.996	188	165.936	22.0642
Piri	GMYN	0.1614	3.6715	0.0440	4.43E-49	594	133.658	460.342	176	109.705	66.2949
Hyp	GMYN	0.0874	1.9998	0.0437	1.49E-16	237	74.8291	162.171	57	43.7518	13.2482
Fatty	MS	0.0686111	1.57088	0.0436769	1.40E-131	1050	263.549	786.451	231	204.364	26.6359
DSBA	MS	0.1276	2.9315	0.0435	NA	603	138.561	464.439	163	142.251	20.7487
DSBA	MS	0.1276	2.9315	0.0435	NA	603	138.561	464.439	163	142.251	20.7487
Hfq	MS	0.0505	1.1646	0.0434	6.17E-15	195	67.7019	127.298	33	30.511	2.48904
Superox	MS	0.0731	1.6862	0.0433	9.78E-61	471	104.752	366.248	99	85.9714	13.0286
MscS	MS	0.1593	3.6796	0.0433	NA	783	186.984	596.016	252	221.435	30.5654
Superox	GMYN	0.1545	3.5797	0.0432	2.51E-40	501	118.272	382.728	147	93.8519	53.1481
Hfq	MS	0.0422	0.9785	0.0431	6.20E-20	234	60.883	173.117	38	33.8495	4.15045
DSBA	MS	0.1640	3.8153	0.0430	NA	618	141.504	476.496	187	163.361	23.6387
Piri	MS	0.1350	3.1715	0.0426	NA	675	144.253	530.747	176	152.168	23.8322
Piri	MS	0.1350	3.1715	0.0426	NA	675	144.253	530.747	176	152.168	23.8322
Piri	GMYN	0.1554	3.6912	0.0421	7.69E-62	615	137.219	477.781	188	121.503	66.4971
Piri	MS	0.1525	3.7022	0.0412	NA	666	159.009	506.991	190	167.943	22.057
Piri	GMYN	0.1563	3.7993	0.0411	3.33E-63	684	158.501	525.499	208	134.636	73.3645
Piri	MS	0.1356	3.3205	0.0408	NA	633	135.082	497.918	171	148.624	22.3756
MscS	GMYN	0.1594	3.9277	0.0406	3.07E-82	783	188.102	594.898	252	167.729	84.271
RpoE	MS	0.0373	0.9192	0.0406	6.41E-39	489	120.406	368.594	74	65.8281	8.1719
Hfq	GMYN	0.0292	0.7239	0.0403	7.47E-08	144	37.722	106.278	18	15	3
RpoE	GMYN	0.0305	0.7767	0.0393	3.39E-31	546	136.588	409.412	74	61.7805	12.2195
RpoE	GMYN	0.0305	0.7767	0.0393	3.39E-31	546	136.588	409.412	74	61.7805	12.2195
RpoE	MS	0.142481	3.63299	0.0392187	NA	426	95.0634	330.937	111	97.6658	13.3342
RpoE	MS	0.0346	0.8836	0.0391	3.37E-41	537	122.034	414.966	74	65.3127	8.68725
DSBA	GMYN	0.1401	3.6040	0.0389	3.66E-50	522	122.167	399.833	153	102.162	50.8375
Hfq	GMYN	0.0442	1.1378	0.0388	1.42E-13	195	51.3775	143.623	33	26.9232	6.07677
Superox	MS	0.1478	3.9317	0.0376	NA	501	112.829	388.171	147	130.161	16.8386
Piri	GMYN	0.1383	3.7079	0.0373	1.97E-62	654	140.313	513.687	185	120.648	64.3521
Hfq	GMYN	0.0478	1.2818	0.0373	5.32E-18	210	55.2251	154.775	41	33.9205	7.0795
Hyp	GMYN	0.0398	1.0712	0.0371	4.83E-18	219	63.1353	155.865	42	36	6
DSBA	GMYN	0.1354	3.6545	0.0370	7.52E-46	564	130.663	433.337	155	101.764	53.2363
MscS	MS	0.1362	3.6868	0.0369	NA	768	186.848	581.152	228	204.509	23.4909
Piri	GMYN	0.1362	3.6944	0.0369	1.26E-51	633	137.803	495.197	171	109.728	61.2724

GroL	GMYN	0.0295	0.8056	0.0366	4.83E-85	1614	398.948	1215.05	210	174.942	35.0579
Piri	GMYN	0.1361	3.7244	0.0365	2.62E-65	690	143.423	546.577	191	123.533	67.4672
RpoH	MS	0.0654	1.7953	0.0364	2.41E-113	906	254.257	651.743	204	186.581	17.4195
DSBA	MS	0.1384	3.8109	0.0363	NA	522	122.466	399.534	153	136.792	16.208
Fatty	MS	0.0708	1.9511	0.0363	3.10E-164	1182	312.083	869.917	276	250.66	25.3403
RpoH	MS	0.0438	1.2207	0.0359	1.02E-82	894	254.367	639.633	162	148.596	13.4035
Alkyl	GMYN	0.0556	1.5720	0.0353	1.44E-30	387	105.061	281.939	79	63.94	15.06
Fatty	MS	0.0600	1.7005	0.0353	1.43E-146	1125	255.687	869.313	230	205.347	24.6531
Alkyl	GMYN	0.0616	1.7443	0.0353	1.49E-37	432	104.463	327.537	90	70.6916	19.3084
Fatty	MS	0.0441	1.2511	0.0353	7.41E-116	1143	298.721	844.279	212	192.785	19.2146
DSBA	MS	0.1303	3.6993	0.0352	NA	600	146.215	453.785	163	146.94	16.0595
DSBA	MS	0.1338	3.8000	0.0352	NA	564	133.261	430.739	155	139.167	15.8333
DSBA	GMYN	0.1316	3.7388	0.0352	2.41E-46	600	145.769	454.231	163	108.579	54.4209
Fatty	GMYN	0.0439	1.2500	0.0352	4.39E-91	1143	288.675	854.325	212	175.616	36.3844
MscS	GMYN	0.1338	3.8727	0.0346	7.12E-64	789	174.787	614.213	212	137.427	74.5728
Hfq	MS	0.0500	1.4481	0.0345	2.03E-22	210	60.6699	149.33	41	37.7899	3.21014
Alkyl	MS	0.0501	1.4584	0.0343	1.09E-49	435	105.063	329.937	81	73.1168	7.88318
DSBA	GMYN	0.1248	3.6412	0.0343	1.15E-57	603	128.368	474.632	163	108.823	54.1775
DSBA	GMYN	0.1248	3.6412	0.0343	1.15E-57	603	128.368	474.632	163	108.823	54.1775
MscS	GMYN	0.1339	3.9083	0.0343	1.80E-79	768	183.276	584.724	228	157.031	70.9689
Fatty	MS	0.0671	1.9638	0.0342	9.31E-170	1161	271.284	889.716	257	231.106	25.8945
RpoH	MS	0.0368315	1.0868	0.03389	3.08E-69	780	218.821	561.179	133	122.365	10.6351
RpoH	MS	0.0967	2.8548	0.0339	1.83E-142	885	232.111	652.889	222	202.684	19.3164
Fatty	MS	0.0546	1.6296	0.0335	4.31E-147	1161	280.18	880.82	238	215.329	22.6708
Carbo	GMYN	0.0530	1.5878	0.0334	2.63E-52	579	148.626	430.374	120	98.0499	21.9501
Hfq	GMYN	0.0414	1.2440	0.0333	8.08E-16	234	59.4434	174.557	38	30.9876	7.01241
Piri	MS	0.1342	4.1084	0.0327	NA	654	142.481	511.519	185	165.585	19.4154
Rubre	GMYN	0.0357	1.0941	0.0326	2.11E-29	381	122.203	258.797	72	63	9
RpoH	GMYN	0.0413	1.2961	0.0318	6.98E-73	894	218.78	675.22	162	134.941	27.0586
RpoH	MS	0.0392	1.2508	0.0313	1.71E-91	912	255.122	656.878	169	156.386	12.6136
RpoE	GMYN	0.0453	1.4523	0.0312	2.25E-42	534	140.738	393.262	101	83.734	17.266
RpoH	MS	0.0576	1.8488	0.0311	5.55E-108	888	261.831	626.169	196	182.414	13.586
GroL	MS	0.0255	0.8289	0.0308	9.77E-113	1608	433.234	1174.77	219	202.11	16.8905
Rubre	GMYN	0.0676	2.1995	0.0307	6.65E-43	438	118.953	319.047	105	84.4634	20.5366

GroL	GMYN	0.0239	0.7832	0.0305	2.93E-85	1551	401.001	1150	199	171.99	27.0099
Hfq	GMYN	0.0307	1.0094	0.0304	7.28E-11	228	61.2365	166.763	28	23	5
Alkyl	GMYN	0.0501	1.6571	0.0302	1.16E-37	435	96.8239	338.176	81	64.647	16.353
Fatty	MS	0.0511	1.6921	0.0302	2.84E-146	1101	260.493	840.507	227	206.861	20.1391
Piri	MS	0.1323	4.4088	0.0300	NA	690	139.996	550.004	191	170.853	20.1475
Piri	GMYN	0.1105	3.7016	0.0298	1.67E-52	669	139.138	529.862	160	105.835	54.1649
GroL	MS	0.0241	0.8124	0.0296	2.24E-102	1551	417.608	1133.39	199	184.183	14.817
Alkyl	GMYN	0.0451	1.5451	0.0292	5.34E-39	432	106.656	325.344	84	69.7976	14.2024
Rubre	GMYN	0.0465	1.5976	0.0291	1.76E-41	450	116.512	333.488	92	77	15
RpoH	GMYN	0.0360859	1.24944	0.0288816	5.77E-56	780	209.234	570.766	133	112.938	20.062
Fatty	GMYN	0.0500	1.7359	0.0288	1.15E-106	1155	298.506	856.494	243	201.757	41.2426
Fatty	MS	0.0614	2.1489	0.0286	6.27E-186	1137	254.923	882.077	256	232.957	23.0428
Alkyl	MS	0.0578	2.0329	0.0285	1.81E-60	432	103.051	328.949	90	82.5059	7.49409
FtsK	GMYN	0.0365	1.2947	0.0282	9.82E-195	2244	602.918	1641.08	430	371.722	58.2781
Carbo	GMYN	0.0695	2.4956	0.0279	1.39E-61	630	166.479	463.521	151	120.379	30.621
Hyp	MS	0.0429	1.5402	0.0279	2.74E-22	219	69.9217	149.078	42	39.6458	2.35418
RpoH	MS	0.0579	2.0835	0.0278	2.69E-122	897	255.006	641.994	206	192.529	13.4715
FtsK	MS	0.0368	1.3299	0.0276	1.73E-229	2244	653.403	1590.6	430	402.885	27.1152
Fatty	MS	0.0488	1.7769	0.0275	1.86E-158	1155	283.951	871.049	243	224.103	18.8968
Alkyl	GMYN	0.0349	1.2734	0.0274	4.78E-36	405	105.767	299.233	75	64.8017	10.1983
RpoH	MS	0.0322	1.1725	0.0274	1.47E-82	903	262.057	640.943	155	145.254	9.74615
Fatty	MS	0.0597	2.1779	0.0274	6.46E-171	1092	249.207	842.793	240	219.643	20.3566
Fatty	GMYN	0.0510	1.8746	0.0272	9.80E-107	1161	282.956	878.044	238	194.787	43.2133
Carbo	MS	0.0711	2.6137	0.0272	2.92E-105	639	145.466	493.534	144	131.831	12.1692
FtsK	GMYN	0.0330	1.2264	0.0269	1.94E-196	2271	620.571	1650.43	428	374.709	53.2915
Hyp	GMYN	0.0384	1.4289	0.0269	1.25E-14	204	42.1986	161.801	30	24	6
Hfq	GMYN	0.0331	1.2354	0.0268	9.32E-19	210	52.7496	157.25	37	31.943	5.057
Fatty	MS	0.0642	2.4045	0.0267	2.80E-189	1167	268.666	898.334	262	240.539	21.4612
Alkyl	MS	0.0440	1.6646	0.0264	1.07E-55	432	103.259	328.741	84	77.4847	6.51525
RpoH	MS	0.0333	1.2678	0.0263	3.24E-79	822	231.935	590.065	145	135.908	9.09161
Fatty	MS	0.0647	2.4607	0.0263	1.27E-190	1158	264.889	893.111	261	239.749	21.2512
RpoE	MS	0.0425	1.6226	0.0262	1.53E-66	534	123.714	410.286	101	92.9237	8.07626
Fimb	GMYN	0.0955	3.6445	0.0262	2.71E-60	489	128.943	360.057	143	110.897	32.1029
RpoE	MS	0.0243	0.9293	0.0262	2.06E-46	543	126.226	416.774	77	70.8697	6.13029

RpoE	MS	0.0224	0.8582	0.0261	3.76E-38	465	103.751	361.249	62	56.8306	5.16941
Hyp	MS	0.0839	3.2142	0.0261	2.06E-35	237	69.3616	167.638	57	53.6181	3.38187
Alkyl	GMYN	0.0518	1.9869	0.0261	8.43E-33	444	119.039	324.961	85	68.7605	16.2395
Hfq	MS	0.0346	1.3306	0.0260	1.16E-21	210	58.9698	151.03	37	34.6928	2.30717
RpoE	GMYN	0.0403	1.5532	0.0260	3.76E-34	522	129.844	392.156	83	67.632	15.368
GroL	MS	0.0173	0.6752	0.0256	5.00E-95	1590	479.487	1110.51	198	186.937	11.0633
Fimb	MS	0.0922	3.6166	0.0255	NA	489	125.954	363.046	143	133.21	9.78959
Rubre	MS	0.0796	3.1231	0.0255	1.44E-52	405	122.483	282.517	92	86.8934	5.1066
Fatty	GMYN	0.0674697	2.72085	0.0247973	1.48E-97	1050	245.528	804.472	231	179.253	51.7474
FtsK	MS	0.0298	1.2058	0.0247	5.23E-226	2256	594.393	1661.61	392	366.665	25.3354
RpoH	GMYN	0.0440	1.7936	0.0245	4.01E-81	849	212.649	636.351	172	144.894	27.1059
Alkyl	GMYN	0.0526	2.1569	0.0244	3.92E-41	420	103.032	316.968	89	72.9183	16.0817
Alkyl	MS	0.0498	2.0586	0.0242	1.22E-50	444	125.475	318.525	85	80.0813	4.91868
GroL	GMYN	0.0126462	0.531829	0.0237787	2.22E-69	1494	456.241	1037.76	163	149.998	13.0021
Alkyl	MS	0.0605	2.5784	0.0235	5.54E-73	459	112.785	346.215	102	95.1455	6.85449
DnaK	MS	0.0176324	0.752496	0.0234318	2.69E-118	1764	495.011	1268.99	226	213.194	12.8063
Hyp	MS	0.0425	1.8575	0.0229	1.78E-19	204	53.2076	150.792	30	28.1741	1.82585
Piri	GMYN	0.1387	6.1000	0.0227	6.44E-48	675	146.142	528.858	176	109.574	66.426
Piri	GMYN	0.1387	6.1000	0.0227	6.44E-48	675	146.142	528.858	176	109.574	66.426
RpoE	MS	0.0271	1.1903	0.0227	2.71E-52	534	117.046	416.954	78	72.1561	5.84387
Carbo	GMYN	0.0806866	3.5869	0.0224948	5.76E-63	522	119.406	402.594	133	102.329	30.6713
GroL	MS	0.0132982	0.594931	0.0223525	7.80E-85	1494	418.369	1075.63	163	154.142	8.85828
Carbo	MS	0.0692	3.1345	0.0221	1.38E-109	624	144.509	479.491	142	132.305	9.6952
Alkyl	MS	0.0366	1.6574	0.0221	7.85E-52	405	96.7634	308.237	75	70.0759	4.92413
Rubre	GMYN	0.0244	1.1113	0.0219	1.15E-38	483	148.491	334.509	86	77.9986	8.00139
Rubre	MS	0.0714	3.2578	0.0219	7.09E-66	438	127.645	310.355	105	99.6893	5.3107
RpoH	GMYN	0.0298	1.3640	0.0218	1.54E-80	903	214.772	688.228	155	134.944	20.0557
Fimb	GMYN	0.0788	3.6154	0.0218	5.38E-53	510	124.028	385.972	125	96.2343	28.7657
RpoH	GMYN	0.0556	2.5579	0.0217	4.37E-92	897	235.377	661.623	206	170.703	35.2973
RpoH	GMYN	0.0880	4.0555	0.0217	8.64E-83	885	223.028	661.972	222	167.291	54.7091
DnaK	MS	0.0191	0.8982	0.0213	1.60E-137	1893	554.357	1338.64	266	252.992	13.0085
Superox	MS	0.0476	2.2384	0.0213	3.12E-56	429	118.796	310.204	89	84.3182	4.68179
Fimb	MS	0.0778	3.6881	0.0211	1.30E-91	510	130.276	379.724	125	117.764	7.23638
Fatty	GMYN	0.0510	2.4188	0.0211	1.44E-107	1101	257.707	843.293	227	185.597	41.4029

DnaK	GMYN	0.0160	0.7616	0.0210	6.21E-123	1866	585.487	1280.51	277	256.799	20.2014
FtsK	GMYN	0.0184	0.8855	0.0208	1.53E-158	2271	671.97	1599.03	347	317.888	29.1116
Alkyl	MS	0.0490	2.3656	0.0207	4.64E-65	420	99.9824	320.018	89	83.4638	5.53621
Carbo	MS	0.0490	2.3829	0.0206	7.60E-86	579	137.615	441.385	120	112.579	7.42133
RpoE	GMYN	0.0228	1.1131	0.0205	3.71E-32	465	106.014	358.986	62	53.9583	8.04168
GroL	GMYN	0.0163	0.7967	0.0204	2.08E-97	1590	408.032	1181.97	198	178.996	19.0042
RpoE	GMYN	0.0224	1.1076	0.0202	2.31E-39	543	131.462	411.538	77	67.9305	9.06949
RpoH	MS	0.0443	2.2379	0.0198	1.43E-101	849	248.834	600.166	172	164.159	7.84083
Superox	GMYN	0.0486	2.4670	0.0197	4.26E-34	429	128.64	300.36	89	74.9082	14.0918
Carbo	GMYN	0.0387	1.9678	0.0197	2.17E-60	603	141.779	461.221	116	98.6223	17.3777
Rubre	GMYN	0.0345	1.7929	0.0192	2.66E-38	462	134.721	327.279	88	77	11
Rubre	GMYN	0.0345	1.7929	0.0192	2.66E-38	462	134.721	327.279	88	77	11
Carbo	GMYN	0.0699	3.6402	0.0192	1.67E-72	624	128.208	495.792	142	109.07	32.9296
FtsK	GMYN	0.0191907	1.01163	0.01897	1.43E-159	2142	655.893	1486.11	352	323.879	28.1214
RpoH	GMYN	0.0309	1.6432	0.0188	1.47E-78	822	188.964	633.036	145	125.878	19.1225
FtsK	MS	0.0308	1.6376	0.0188	7.34E-283	2265	600.328	1664.67	448	425.805	22.1948
FtsK	GMYN	0.0229	1.2204	0.0188	1.41E-185	2292	685.669	1606.33	405	368.807	36.1931
RpoH	GMYN	0.0616	3.2919	0.0187	1.53E-90	906	220.911	685.089	204	163.627	40.3726
Fimb	MS	0.0581	3.1576	0.0184	5.75E-82	504	129.606	374.394	114	108.243	5.75668
Carbo	GMYN	0.0295	1.6056	0.0183	9.12E-56	558	138.878	419.122	104	91.9133	12.0867
DnaK	MS	0.0185	1.0103	0.0183	4.45E-141	1830	550.86	1279.14	267	256.134	10.8657
FtsK	GMYN	0.0248	1.3630	0.0182	7.24E-214	2205	599.942	1605.06	416	376.857	39.1426
Carbo	GMYN	0.0653	3.6205	0.0180	5.11E-98	627	124.875	502.125	154	122.929	31.0711
DnaK	MS	0.0163	0.9043	0.0180	3.02E-146	1917	539.196	1377.8	265	253.358	11.6424
Carbo	GMYN	0.0554	3.0948	0.0179	4.18E-65	615	158.128	456.872	141	116.682	24.3185
Alkyl	GMYN	0.0621	3.4839	0.0178	1.74E-48	459	104.089	354.911	102	80.9172	21.0828
FtsK	GMYN	0.0317	1.7850	0.0178	4.45E-232	2265	581.485	1683.51	448	395.749	52.2514
GroL	GMYN	0.0239	1.3477	0.0177	5.70E-89	1596	357.271	1238.73	191	161.964	29.0361
FtsK	MS	0.0222	1.2567	0.0177	1.08E-233	2292	651.722	1640.28	405	387.739	17.2606
FtsK	MS	0.0186167	1.07178	0.0173699	9.31E-196	2142	623.939	1518.06	352	337.727	14.2728
Carbo	GMYN	0.0644	3.7198	0.0173	2.17E-69	639	142.548	496.452	144	113.49	30.5099
RpoE	MS	0.0153	0.8914	0.0172	4.23E-44	507	115.697	391.303	66	62.3697	3.63032
DnaK	MS	0.0142	0.8334	0.0170	3.87E-139	1878	517.35	1360.65	248	237.374	10.626
DnaK	MS	0.0176	1.0387	0.0169	4.08E-156	1890	548.263	1341.74	284	272.71	11.2896

Alkyl	MS	0.0477	2.8312	0.0169	1.18E-67	387	80.7862	306.214	79	74.2535	4.74646
GroL	MS	0.0099	0.6139	0.0162	9.83E-92	1545	435.919	1109.08	169	162.326	6.67375
Fatty	GMYN	0.0672	4.1833	0.0161	2.82E-133	1182	264.481	917.519	276	217.206	58.7944
DnaK	MS	0.0121	0.7511	0.0161	3.00E-127	1890	547.109	1342.89	238	228.969	9.03078
Fatty	GMYN	0.0672	4.1854	0.0161	7.91E-112	1161	265.218	895.782	257	199.624	57.3765
DnaK	MS	0.0120	0.7478	0.0161	6.32E-128	1899	549.353	1349.65	239	229.928	9.07215
Fimb	GMYN	0.0580	3.6129	0.0160	3.61E-53	504	123.612	380.388	114	92.8365	21.1635
DnaK	MS	0.0096	0.5981	0.0160	2.37E-111	1896	497.188	1398.81	197	188.51	8.48977
FtsK	MS	0.0252	1.5749	0.0160	1.24E-259	2205	596.985	1608.01	416	398.838	17.1616
Carbo	MS	0.0658	4.1225	0.0160	NA	627	144.387	482.613	154	146.196	7.80388
Carbo	MS	0.0326	2.0699	0.0158	4.94E-94	654	149.461	504.539	126	119.631	6.36862
RpoH	GMYN	0.0530	3.3685	0.0157	1.12E-92	888	218.22	669.78	196	161.831	34.1686
Carbo	MS	0.0640	4.0729	0.0157	4.79E-129	630	147.092	482.908	151	143.591	7.40934
Fimb	GMYN	0.0562	3.6049	0.0156	4.11E-54	498	122.299	375.701	113	92.7167	20.2833
Fimb	GMYN	0.0562	3.6049	0.0156	4.11E-54	498	122.299	375.701	113	92.7167	20.2833
DnaK	MS	0.0160	1.0334	0.0155	1.01E-143	1866	583.693	1282.31	277	267.866	9.13392
RpoH	MS	0.0176	1.1343	0.0155	6.01E-84	891	253.7	637.3	144	138.604	5.39645
Fimb	GMYN	0.0563	3.6370	0.0155	2.90E-60	480	127.652	352.348	122	102.96	19.0403
Fatty	GMYN	0.0629	4.1281	0.0152	1.96E-134	1158	245.709	912.291	261	206.176	54.8241
DnaK	MS	0.0109	0.7159	0.0152	2.12E-122	1824	524.785	1299.22	226	217.802	8.19835
Fimb	GMYN	0.0545	3.5996	0.0151	4.43E-54	465	121.442	343.558	112	93.9862	18.0138
Carbo	GMYN	0.0215	1.4251	0.0151	3.55E-64	639	162.959	476.041	114	103.941	10.0589
Fimb	MS	0.0550	3.6437	0.0151	3.75E-96	480	126.395	353.605	122	117.054	4.94559
Fimb	GMYN	0.0541	3.5853	0.0151	7.74E-54	489	119.156	369.844	110	90.7937	19.2063
Fatty	GMYN	0.0623	4.1310	0.0151	1.00E-135	1167	246.651	920.349	262	207.187	54.8133
DnaK	MS	0.0084	0.5584	0.0150	5.98E-105	1857	534.311	1322.69	195	188.012	6.98776
Fatty	GMYN	0.0598	4.0524	0.0148	7.34E-151	1137	222.11	914.89	256	203.582	52.4181
RpoE	GMYN	0.0268	1.8250	0.0147	8.89E-43	534	112.498	421.502	78	66.9366	11.0634
Fimb	MS	0.0559	3.8195	0.0146	1.50E-86	498	124.921	373.079	113	108.266	4.73378
Fimb	MS	0.0559	3.8195	0.0146	1.50E-86	498	124.921	373.079	113	108.266	4.73378
DnaK	GMYN	0.0182	1.2457	0.0146	3.65E-135	1893	483.934	1409.07	266	240.74	25.2598
FtsK	MS	0.0150	1.0321	0.0146	5.19E-209	2268	620.198	1647.8	353	339.852	13.1481
Rubre	MS	0.0488	3.3624	0.0145	5.52E-58	450	129.181	320.819	92	88.8017	3.19828
FtsK	MS	0.0149	1.0308	0.0145	1.69E-209	2277	623.013	1653.99	354	340.878	13.1224

Fatty	GMYN	0.0590	4.0728	0.0145	2.00E-127	1092	228.241	863.759	240	191.199	48.8012
FtsK	MS	0.0134	0.9306	0.0144	1.17E-188	2232	655.371	1576.63	339	327.62	11.3798
Fimb	MS	0.0537	3.7249	0.0144	3.32E-86	465	119.856	345.144	112	107.533	4.46737
Fatty	GMYN	0.0594	4.1670	0.0143	1.01E-106	1125	240.517	884.483	230	179.691	50.3093
Fimb	MS	0.0546	3.8451	0.0142	4.05E-84	489	121.966	367.034	110	105.496	4.50405
Carbo	MS	0.0362	2.6543	0.0137	3.55E-88	603	141.123	461.877	116	111.038	4.9616
Rubre	MS	0.0367	2.7185	0.0135	6.40E-50	462	147.886	314.114	88	85.5493	2.45071
Rubre	MS	0.0367	2.7185	0.0135	6.40E-50	462	147.886	314.114	88	85.5493	2.45071
GroL	MS	0.0098	0.7316	0.0134	6.69E-107	1635	445.156	1189.84	187	180.532	6.46815
RuvB	GMYN	0.0176	1.3142	0.0134	2.34E-104	1032	282.23	749.77	188	174.974	13.0262
Carbo	MS	0.0217	1.6203	0.0134	1.31E-73	639	169.63	469.37	114	109.927	4.07271
DnaK	GMYN	0.0156	1.1806	0.0132	2.47E-141	1917	483.017	1433.98	265	242.828	22.1722
Carbo	MS	0.0266	2.0222	0.0131	4.16E-79	558	127.269	430.731	104	99.5746	4.42543
Alkyl	MS	0.0224	1.7097	0.0131	9.32E-46	363	80.6686	282.331	60	57.3708	2.62924
FtsK	GMYN	0.0298	2.2884	0.0130	1.48E-191	2256	569.943	1686.06	392	342.76	49.2405
FtsK	GMYN	0.0133	1.0223	0.0130	1.03E-174	2232	635.419	1596.58	339	317.958	21.042
DnaK	GMYN	0.0115	0.8884	0.0130	2.93E-129	1899	489.13	1409.87	239	222.916	16.0843
DnaK	GMYN	0.0161	1.2408	0.0130	1.65E-172	1890	429.855	1460.14	284	260.749	23.2506
RpoH	GMYN	0.0378	2.9140	0.0130	1.69E-78	912	230.945	681.055	169	143.987	25.0134
RuvB	MS	0.0195	1.5113	0.0129	1.17E-122	1032	274.545	757.455	188	181.547	6.45305
DnaK	GMYN	0.0116	0.9016	0.0128	2.25E-129	1890	483.897	1406.1	238	221.917	16.0828
GroL	GMYN	0.0095	0.7438	0.0128	2.28E-94	1545	378.873	1166.13	169	157.998	11.0017
Alkyl	GMYN	0.0421388	3.35097	0.0125751	2.70E-46	387	87.1785	299.822	81	68.7847	12.2153
Carbo	GMYN	0.0327	2.6131	0.0125	2.31E-71	654	151.246	502.754	126	109.954	16.0458
Hfq	MS	0.0118487	0.954838	0.0124092	2.40E-20	234	61.682	172.318	32	30.9278	1.07217
Carbo	MS	0.0513	4.1831	0.0123	6.84E-123	615	141.208	473.792	141	135.426	5.57402
RuvB	MS	0.0216	1.7691	0.0122	7.10E-134	1014	262.888	751.112	195	188.427	6.57318
RuvB	MS	0.0248	2.0379	0.0122	7.82E-130	978	256.851	721.149	191	184.694	6.30582
Alkyl	MS	0.0309	2.5430	0.0121	3.46E-65	444	108.251	335.749	88	84.8045	3.19553
Alkyl	MS	0.0309	2.5430	0.0121	3.46E-65	444	108.251	335.749	88	84.8045	3.19553
RpoH	GMYN	0.0158	1.3085	0.0121	1.04E-84	855	214.857	640.143	143	132.991	10.0092
DnaK	GMYN	0.0091	0.7509	0.0121	2.72E-114	1896	439.163	1456.84	197	183.896	13.1045
RuvB	GMYN	0.0213	1.8020	0.0118	1.39E-109	978	257.85	720.15	191	175.891	15.1094
Rubre	MS	0.0362	3.0609	0.0118	1.38E-42	381	121.404	259.596	72	70.2252	1.7748

RuvB	GMYN	0.0233258	1.98957	0.0117241	1.43E-100	921	219.415	701.585	169	152.967	16.0331
RuvB	GMYN	0.0161	1.3747	0.0117	1.25E-101	963	268.211	694.789	180	168.983	11.0174
RpoH	MS	0.0165	1.4136	0.0117	2.64E-88	855	237.894	617.106	143	138.795	4.20463
DnaK	GMYN	0.0162898	1.39952	0.0116396	1.41E-128	1764	390.355	1373.64	226	203.891	22.1088
FtsK	MS	0.0125	1.0848	0.0115	8.69E-202	2202	661.527	1540.47	356	346.733	9.26654
GroL	MS	0.0060	0.5348	0.0113	1.07E-88	1611	457.404	1153.6	160	155.571	4.4293
GroL	MS	0.0060	0.5393	0.0111	7.01E-90	1620	460.493	1159.51	162	157.582	4.41769
Hyp	GMYN	0.0335	3.0362	0.0110	3.22E-19	162	39.3534	122.647	33	29	4
DnaK	GMYN	0.0079	0.7183	0.0110	7.00E-114	1857	443.221	1413.78	195	183.936	11.0639
Alkyl	GMYN	0.0258	2.3727	0.0109	8.14E-33	363	86.3952	276.605	60	52.9968	7.00321
GroL	GMYN	0.0096	0.8940	0.0108	1.68E-111	1635	377.041	1257.96	187	174.998	12.0019
GroL	MS	0.0043	0.4033	0.0107	1.37E-72	1614	461.508	1152.49	133	129.544	3.45647
FtsK	GMYN	0.0139	1.3083	0.0106	7.42E-195	2277	606.13	1670.87	354	330.962	23.038
FtsK	GMYN	0.0140	1.3271	0.0105	1.31E-194	2268	602.647	1665.35	353	329.962	23.0377
RuvB	MS	0.0161	1.5556	0.0104	3.38E-115	963	272.316	690.684	180	175.384	4.61607
RuvB	GMYN	0.0154	1.4935	0.0103	1.32E-108	996	268.642	727.358	185	173.976	11.0241
Fimb	GMYN	0.0311	3.0580	0.0102	4.80E-51	417	110.971	306.029	91	81.8172	9.18276
RuvB	MS	0.0154	1.5575	0.0099	1.30E-124	996	268.221	727.779	185	180.16	4.84025
Rubre	MS	0.0245	2.5196	0.0097	5.37E-53	483	146.595	336.405	86	84.1211	1.87894
Fimb	GMYN	0.0335	3.5954	0.0093	1.69E-62	465	120.768	344.232	107	95.9016	11.0984
GroL	GMYN	0.0058	0.6384	0.0091	7.74E-94	1611	398.76	1212.24	160	152.999	7.00051
RpoH	GMYN	0.0168	1.8747	0.0090	1.14E-81	891	226.384	664.616	144	132.978	11.0217
GroL	GMYN	0.0058	0.6509	0.0088	1.40E-95	1620	399.484	1220.52	162	155	7.00048
DnaK	GMYN	0.0133	1.5135	0.0088	5.94E-149	1878	423.783	1454.22	248	228.79	19.2105
RuvB	MS	0.0289	3.2835	0.0088	5.08E-168	1008	224.501	783.499	202	195.973	6.02699
Hfq	MS	0.0128	1.4557	0.0088	0	207	55.9527	151.047	32	31.2594	0.740612
DnaK	GMYN	0.0168	1.9225	0.0087	2.32E-155	1830	423.986	1406.01	267	243.718	23.2819
Fimb	MS	0.0307	3.5496	0.0087	3.34E-64	417	114.861	302.139	91	88.974	2.02604
FtsK	GMYN	0.0123	1.4236	0.0086	3.02E-189	2202	635.725	1566.27	356	336.975	19.0248
Alkyl	MS	0.0374733	4.36149	0.00859187	4.13E-71	387	86.1463	300.854	81	78.6403	2.35967
RuvB	MS	0.0226	2.6409	0.0085	3.67E-159	1032	265.99	766.01	216	210.81	5.18987
RuvB	MS	0.0222919	2.61062	0.00853893	3.84E-144	921	191.99	729.01	169	163.693	5.30748
GroL	GMYN	0.0237	2.8105	0.0084	2.00E-110	1608	363.96	1244.04	219	189.962	29.0379
Fimb	MS	0.0323	3.8914	0.0083	5.03E-88	465	116.601	348.399	107	104.409	2.5913

GroL	MS	0.0038	0.4759	0.0080	2.62E-82	1575	433.088	1141.91	140	137.114	2.88573
Alkyl	GMYN	0.0333	4.3104	0.0077	4.72E-51	444	102.89	341.11	88	76.9209	11.0791
Alkyl	GMYN	0.0333	4.3104	0.0077	4.72E-51	444	102.89	341.11	88	76.9209	11.0791
Hyp	MS	0.0329	4.2779	0.0077	0	162	36.9056	125.094	33	32.1607	0.839256
Hfq	GMYN	0.0068	0.8991	0.0075	4.58E-17	219	70.2969	148.703	32	31	1
RuvB	MS	0.0095	1.2635	0.0075	9.59E-112	1029	286.963	742.037	174	170.69	3.31038
GroL	GMYN	0.0041	0.5418	0.0075	1.55E-81	1614	378.782	1235.22	133	128	5.00025
RpoE	GMYN	0.0148	2.0241	0.0073	7.32E-45	507	97.0133	409.987	66	59.9993	6.00071
RuvB	GMYN	0.0289	4.0509	0.0071	1.21E-130	1008	221.661	786.339	202	179.927	22.0727
Hfq	MS	0.0067	0.9608	0.0070	0	219	65.6215	153.379	32	31.4868	0.51322
RuvB	MS	0.0229	3.3218	0.0069	1.49E-179	1053	255.307	797.693	222	217.314	4.68602
Hfq	GMYN	0.0131	2.0777	0.0063	8.37E-20	207	52.0075	154.992	32	29.9994	2.00059
DnaK	GMYN	0.0102	1.6663	0.0061	1.63E-134	1824	431.263	1392.74	226	211.953	14.0469
Hfq	GMYN	0.0166	2.8099	0.0059	7.18E-24	165	42.3758	122.624	35	33	2
Hfq	GMYN	0.0112376	2.02923	0.00553787	4.09E-21	234	53.0886	180.911	32	29.9916	2.00839
RuvB	GMYN	0.0227	4.1202	0.0055	7.49E-153	1053	243.125	809.875	222	203.947	18.0532
RuvB	GMYN	0.0218	4.0990	0.0053	8.15E-151	1032	236.349	795.651	216	198.91	17.0905
RuvB	GMYN	0.0207	4.0690	0.0051	3.83E-132	1014	227.079	786.921	195	178.926	16.0736
RuvB	GMYN	0.0094	1.8698	0.0050	1.27E-105	1029	278.984	750.016	174	166.992	7.00753
GroL	GMYN	0.0033	0.7345	0.0045	3.09E-89	1575	369.8	1205.2	140	136	4.00009
Hfq	MS	0.0162	4.1595	0.0039	0	165	39.1784	125.822	35	34.568	0.431958
Hfq	MS	0.0000	0.9622	0.0000	0	189	54.6278	134.372	24	23.9999	5.90E-05
Cold	MS	0.0000	0.3117	0.0000	0	171	45.7305	125.269	10	9.99997	2.74E-05
Cold	MS	0.0000	0.1806	0.0000	0	168	48.6253	119.375	7	6.99998	1.72E-05
Cold	MS	0.0000	0.1975	0.0000	0	156	45.9512	110.049	7	6.99998	1.68E-05
Cold	MS	0.0000	0.1104	0.0000	0	195	45.2978	149.702	5	4.99998	1.65E-05
Cold	MS	0.0000	0.1302	0.0000	0	171	38.4118	132.588	5	4.99998	1.73E-05
Cold	MS	5.04E-08	0.0504104	1.00E-06	0	195	65.3257	129.674	3	2.99999	5.96E-06
Cold	GMYN	1.11E-15	0.0675801	1.64E-14	0	195	47.762	147.238	3	3	0
Cold	GMYN	0.0000	0.2167	0.0000	0	168	40.1796	127.82	7	7	0
Hfq	GMYN	0.0000	1.0078	0.0000	0	189	43.2947	145.705	24	24	0
Cold	GMYN	0.0000	0.2277	0.0000	0	156	38.2496	117.75	7	7	0
Cold	GMYN	0.0000	0.1473	0.0000	0	171	38.8647	132.135	5	5	0

Housekeeping genes in PnecC subcluster

Sequence	Method	Ka	Ks	Ka/Ks	P-Value(Fisher)	Length	S-Sites	N-Sites	Substitutions	S-Substitutions	N-Substitutions
ndk	GMYN	0.0382	0.4817	0.0794	1.12E-13	411	98.3222	312.678	43	31.3974	11.6026
ndk	MS	0.0300	0.5242	0.0573	2.44E-19	423	112.061	310.939	45	38.829	6.17102
ndk	MS	0.0349	0.6510	0.0537	7.66E-23	411	78.9238	332.076	43	35.0774	7.92264
ndk	GMYN	0.0330	0.6499	0.0508	1.03E-19	420	102.274	317.726	51	40.795	10.205
adk	MS	0.0423	0.8510	0.0497	2.46E-46	663	175.563	487.437	100	87.8641	12.1359
glnA	MS	0.0245	0.4959	0.0494	3.22E-65	1413	347.415	1065.59	144	125.049	18.9507
acsA	MS	0.0451	0.9224	0.0489	1.22E-147	1968	505.133	1462.87	312	273.294	38.7059
ndk	GMYN	0.0281	0.6091	0.0461	1.96E-18	423	94.9409	328.059	45	35.9672	9.03279
adk	MS	0.0394	0.8747	0.0450	1.55E-47	651	167.084	483.916	95	84.0386	10.9614
ndk	MS	0.0321	0.7148	0.0448	1.13E-25	420	101.298	318.702	51	44.6942	6.30577
adk	MS	0.0582	1.3849	0.0421	6.22E-68	660	172.253	487.747	127	113.485	13.5149
glnA	MS	0.0234	0.5632	0.0415	9.55E-74	1413	379.737	1033.26	161	144.653	16.3467
glnA	MS	0.0262	0.6366	0.0412	7.71E-80	1401	327.913	1073.09	159	140.12	18.8796
acsA	GMYN	0.0450	1.1273	0.0399	2.22E-115	1968	483.851	1484.15	312	247.275	64.7248
ndk	GMYN	0.0112	0.2822	0.0398	1.89E-10	354	83.7145	270.285	22	19	3
ndk	MS	0.0271	0.6862	0.0394	9.61E-26	423	92.2277	330.772	46	40.3017	5.69831
acsA	MS	0.0365	0.9353	0.0390	4.32E-149	1887	507.18	1379.82	300	271.218	28.782
acsA	MS	0.0332	0.8538	0.0389	3.33E-145	1971	529.54	1441.46	297	268.591	28.4093
acsA	MS	0.0271	0.7413	0.0365	1.82E-132	1971	537.997	1433	274	249.709	24.2911
adk	GMYN	0.0320	0.8913	0.0359	2.95E-37	594	172.334	421.666	94	80.8497	13.1503
glnA	MS	0.0212	0.5916	0.0358	1.08E-79	1413	366.88	1046.12	161	146.086	14.9141
iso	MS	0.0367	1.0320	0.0355	7.66E-192	2220	555.089	1664.91	358	323.532	34.4682
acsA	MS	0.0303	0.8617	0.0351	6.64E-147	1971	493.945	1477.05	281	254.288	26.7124
acsA	MS	0.0303	0.8617	0.0351	6.64E-147	1971	493.945	1477.05	281	254.288	26.7124
adk	GMYN	0.0356	1.0140	0.0351	3.30E-39	651	158.644	492.356	95	77.933	17.067
glnA	MS	0.0163	0.4674	0.0349	3.72E-65	1413	369.219	1043.78	136	123.8	12.2004
glnA	MS	0.0163	0.4674	0.0349	3.72E-65	1413	369.219	1043.78	136	123.8	12.2004
acsA	MS	0.0335	0.9679	0.0346	1.03E-150	1971	592.697	1378.3	318	294.294	23.7064
glnA	MS	0.0260	0.7578	0.0343	2.59E-97	1413	365.432	1047.57	189	172.082	16.9183
iso	MS	0.0449	1.3134	0.0342	7.77E-219	2232	594.613	1637.39	406	371.058	34.942
adk	MS	0.0250	0.7580	0.0330	1.77E-41	663	208.577	454.423	91	84.9041	6.09593

cit	MS	0.0228	0.6920	0.0329	1.20E-84	1311	342.568	968.432	167	152.781	14.2187
ndk	MS	0.0108	0.3335	0.0325	8.68E-12	354	84.6678	269.332	22	19.9386	2.06145
acsA	MS	0.0259	0.7986	0.0324	1.53E-138	1971	559.195	1411.81	283	261.597	21.4034
iso	GMYN	0.0436	1.3547	0.0322	1.17E-180	2232	533.201	1698.8	406	334.209	71.7912
glnA	GMYN	0.0212	0.6623	0.0321	1.63E-69	1413	355.65	1057.35	161	138.89	22.1102
atpA	GMYN	0.0118	0.3687	0.0320	5.77E-48	1470	357.029	1112.97	107	94	13
iso	MS	0.0377	1.1919	0.0317	4.93E-205	2229	613.557	1615.44	390	359.982	30.0182
adk	MS	0.0281	0.8887	0.0316	2.92E-49	663	190.603	472.397	96	89.0224	6.97763
adk	MS	0.0281	0.8887	0.0316	2.92E-49	663	190.603	472.397	96	89.0224	6.97763
glnA	GMYN	0.0231	0.7397	0.0312	5.55E-64	1413	301.413	1111.59	144	118.749	25.2505
iso	GMYN	0.0367	1.1967	0.0306	2.46E-178	2229	552.028	1676.97	390	330.066	59.9338
succ	MS	0.0249	0.8416	0.0296	3.14E-131	1776	464.767	1311.23	249	229.811	19.1886
adk	GMYN	0.0551	1.8739	0.0294	5.14E-57	660	144.849	515.151	127	99.7069	27.2931
succ	MS	0.0228	0.7962	0.0287	6.53E-126	1776	506.432	1269.57	250	233.227	16.7728
iso	MS	0.0246	0.8577	0.0286	5.64E-158	2148	607.868	1540.13	313	291.834	21.166
cit	MS	0.0190	0.6864	0.0277	1.37E-78	1242	345.667	896.333	155	144.602	10.3981
glnA	GMYN	0.0108	0.3915	0.0275	5.94E-53	1413	382.426	1030.57	118	106.994	11.0057
iso	GMYN	0.0244	0.8963	0.0272	3.15E-134	2148	596.948	1551.05	313	275.79	37.2096
adk	MS	0.0336	1.2359	0.0272	4.20E-55	594	152.53	441.47	94	87.1361	6.86386
CTP	MS	0.0383	1.4202	0.0270	1.53E-182	1659	419.317	1239.68	305	282.456	22.5437
ndk	GMYN	0.0263	0.9812	0.0268	2.48E-24	423	73.4068	349.593	46	36.9863	9.01365
acsA	MS	0.0254	0.9576	0.0265	4.92E-162	1971	532.638	1438.36	299	279	19.9996
atpA	GMYN	0.0099	0.3724	0.0265	1.84E-55	1539	417.596	1121.4	123	112	11
iso	MS	0.0241	0.9105	0.0265	6.78E-177	2232	583.67	1648.33	324	301.438	22.5624
CTP	MS	0.0441	1.6630	0.0265	5.29E-200	1659	406.643	1252.36	318	294.014	23.9861
adk	GMYN	0.0270	1.0241	0.0263	2.16E-43	663	169.874	493.126	96	82.9971	13.0029
adk	GMYN	0.0270	1.0241	0.0263	2.16E-43	663	169.874	493.126	96	82.9971	13.0029
acsA	GMYN	0.0307	1.1675	0.0263	7.83E-157	1971	460.746	1510.25	318	272.702	45.2985
gyr	GMYN	0.0413	1.5850	0.0260	5.04E-137	1434	390.054	1043.95	299	257.19	41.8102
adk	MS	0.0266	1.0241	0.0260	1.51E-54	663	196.497	466.503	103	97.0207	5.97934
iso	MS	0.0307	1.1857	0.0259	7.20E-202	2232	621.318	1610.68	373	349.512	23.4885
adk	GMYN	0.0230	0.9005	0.0256	2.82E-41	663	175.583	487.417	91	79.9576	11.0424
adk	GMYN	0.0336	1.3159	0.0255	6.14E-54	663	141.762	521.238	105	87.9287	17.0713
CTP	GMYN	0.0379	1.4861	0.0255	3.88E-147	1659	399.829	1259.17	305	258.527	46.4732

iso	GMYN	0.0300	1.1779	0.0255	2.24E-183	2232	544.351	1687.65	373	323.368	49.6319
glnA	MS	0.0104	0.4212	0.0248	1.15E-62	1413	361.456	1051.54	118	110.068	7.93202
cit	GMYN	0.0225	0.9200	0.0244	3.22E-78	1311	316.177	994.823	167	144.987	22.0131
acsA	GMYN	0.0356	1.4655	0.0243	3.08E-127	1887	469.867	1417.13	300	250.775	49.2249
ndk	GMYN	0.0125	0.5149	0.0243	1.76E-20	423	99.3462	323.654	39	35	4
iso	MS	0.0246	1.0173	0.0242	7.82E-196	2232	578.399	1653.6	345	322.713	22.287
acsA	MS	0.0191	0.7978	0.0240	7.18E-143	1959	537.515	1421.49	267	251.083	15.9166
cit	MS	0.0220	0.9288	0.0237	2.36E-108	1311	325.072	985.928	186	173.521	12.4791
gyr	GMYN	0.0496	2.0975	0.0237	2.78E-138	1365	365.201	999.799	305	257.189	47.8108
glnA	MS	0.0110	0.4696	0.0234	9.82E-66	1344	344.318	999.682	122	114.235	7.76513
cit	MS	0.0248	1.0765	0.0230	1.78E-104	1311	403.661	907.339	207	196.811	10.1895
cit	MS	0.0248	1.0818	0.0229	1.70E-120	1299	314.94	984.06	199	185.712	13.2882
glnA	GMYN	0.0223	0.9748	0.0228	1.26E-75	1413	314.461	1098.54	161	136.929	24.0706
glnA	MS	0.0079	0.3491	0.0225	2.74E-54	1413	404.929	1008.07	109	103.207	5.79339
adk	MS	0.0231	1.0322	0.0224	1.05E-56	663	176.891	486.109	98	92.3184	5.68161
iso	MS	0.0223	0.9998	0.0223	1.13E-190	2232	596.507	1635.49	339	319.447	19.5526
iso	MS	0.0223	0.9998	0.0223	1.13E-190	2232	596.507	1635.49	339	319.447	19.5526
ndk	MS	0.0097	0.4362	0.0222	9.09E-21	423	116.198	306.802	36	34.0073	1.99266
acsA	GMYN	0.0316	1.4453	0.0218	9.23E-142	1971	442.811	1528.19	297	249.879	47.1209
glnA	GMYN	0.0252	1.1563	0.0218	5.25E-93	1413	316.056	1096.94	189	161.881	27.1187
CTP	GMYN	0.0287	1.3198	0.0218	1.33E-147	1659	407.071	1251.93	288	252.764	35.2364
gyr	GMYN	0.0433	2.0001	0.0216	9.49E-147	1422	384.668	1037.33	312	268.459	43.5413
CTP	MS	0.0445	2.0753	0.0215	2.13E-220	1656	426.505	1229.5	340	320.192	19.8082
succ	MS	0.0264	1.2285	0.0215	2.73E-172	1776	450.743	1325.26	288	270.908	17.0919
succ	MS	0.0176	0.8302	0.0212	4.27E-135	1776	438.421	1337.58	230	216.015	13.9846
cit	MS	0.0187	0.8896	0.0210	6.26E-103	1311	344.629	966.371	182	171.865	10.1346
atpA	MS	0.0069	0.3281	0.0210	2.25E-58	1539	414.089	1124.91	111	105.009	5.99135
iso	MS	0.0194	0.9274	0.0209	3.43E-180	2232	612.155	1619.84	327	309.838	17.1624
ndk	MS	0.0121	0.5806	0.0209	6.22E-24	423	99.3507	323.649	39	36.5139	2.48615
CTP	MS	0.0452	2.1968	0.0206	1.82E-239	1659	389.105	1269.89	339	317.671	21.3295
succ	MS	0.0244	1.1871	0.0206	1.84E-169	1776	457.36	1318.64	283	267.154	15.8464
atpA	MS	0.0109	0.5337	0.0205	3.59E-84	1536	431.827	1104.17	163	154.883	8.11725
CTP	MS	0.0287	1.4080	0.0204	5.77E-183	1659	410.376	1248.62	288	271.188	16.812
acsA	MS	0.0190	0.9509	0.0199	6.22E-165	1971	534.467	1436.53	291	276.202	14.7979

CTP	MS	0.0469	2.3755	0.0197	1.85E-244	1659	395.744	1263.26	345	324.552	20.4483
iso	MS	0.0161	0.8209	0.0196	1.85E-170	2232	595.338	1636.66	303	287.544	15.4558
atpA	GMYN	0.0063	0.3240	0.0194	4.47E-54	1539	420.3	1118.7	111	104	7
glnA	GMYN	0.0257	1.3264	0.0193	8.04E-75	1401	284.568	1116.43	159	130.875	28.1248
ndk	GMYN	0.0093	0.5018	0.0186	4.30E-20	423	98.377	324.623	36	33	3
glnA	GMYN	0.0107	0.5838	0.0184	1.10E-64	1344	311.139	1032.86	122	110.994	11.0057
glnA	GMYN	0.0077	0.4163	0.0184	6.23E-55	1413	360.822	1052.18	109	100.997	8.00264
succ	MS	0.0213	1.1644	0.0183	1.66E-166	1764	411.916	1352.08	259	244.355	14.6446
adk	GMYN	0.0392	2.1557	0.0182	1.77E-47	663	133.984	529.016	100	79.8747	20.1253
gyr	GMYN	0.0314	1.7556	0.0179	3.20E-125	1434	410.89	1023.11	275	243.578	31.4223
gyr	GMYN	0.0402	2.2461	0.0179	1.53E-156	1434	390.631	1043.37	319	278.312	40.6879
ndk	MS	0.0068	0.3892	0.0176	1.40E-18	423	108.122	314.878	31	29.4898	1.51023
pyr	GMYN	0.0365	2.0799	0.0175	1.84E-152	1434	368.689	1065.31	297	259.245	37.7552
acsA	GMYN	0.0272	1.5565	0.0175	3.77E-141	1971	432.585	1538.41	281	239.927	41.0729
acsA	GMYN	0.0272	1.5565	0.0175	3.77E-141	1971	432.585	1538.41	281	239.927	41.0729
cit	GMYN	0.0236	1.3596	0.0173	3.04E-96	1311	359.966	951.034	207	184.961	22.0394
cit	MS	0.0118	0.6805	0.0173	3.34E-86	1311	376.81	934.19	160	153.414	6.58645
iso	GMYN	0.0245	1.4186	0.0172	2.73E-144	2232	595.329	1636.67	324	284.646	39.3536
adk	MS	0.0273	1.6085	0.0170	2.16E-71	663	178.29	484.71	116	110.878	5.12185
cit	MS	0.0140	0.8290	0.0169	3.17E-103	1311	318.87	992.13	168	159.61	8.38997
cit	MS	0.0140	0.8290	0.0169	3.17E-103	1311	318.87	992.13	168	159.61	8.38997
succ	MS	0.0244	1.4442	0.0169	1.68E-197	1773	415.09	1357.91	294	278.629	15.3715
CTP	MS	0.0431	2.5545	0.0169	4.83E-258	1647	388.169	1258.83	348	329.961	18.0385
acsA	GMYN	0.0253	1.5192	0.0167	9.39E-140	1971	433.419	1537.58	274	235.81	38.1899
iso	GMYN	0.0222	1.3559	0.0164	2.72E-166	2232	575.916	1656.08	339	302.855	36.1454
iso	GMYN	0.0222	1.3559	0.0164	2.72E-166	2232	575.916	1656.08	339	302.855	36.1454
cit	MS	0.0226	1.3813	0.0163	2.13E-148	1308	317.063	990.937	222	211.209	10.7907
glnA	GMYN	0.0140	0.8743	0.0161	5.05E-67	1413	332.257	1080.74	136	120.992	15.0082
glnA	GMYN	0.0140	0.8743	0.0161	5.05E-67	1413	332.257	1080.74	136	120.992	15.0082
ndk	GMYN	0.0063	0.3899	0.0160	9.94E-18	423	101.342	321.658	31	29	2
gyr	GMYN	0.0438	2.7416	0.0160	4.14E-155	1434	367.735	1066.27	314	268.672	45.3283
cit	GMYN	0.0227	1.4395	0.0158	6.41E-126	1308	312.118	995.882	222	199.746	22.2536
CTP	MS	0.0291	1.8451	0.0158	1.39E-216	1659	396.265	1262.73	309	294.203	14.7971
CTP	MS	0.0291	1.8451	0.0158	1.39E-216	1659	396.265	1262.73	309	294.203	14.7971

gyr	MS	0.0292	1.8620	0.0157	1.38E-164	1434	412.584	1021.42	275	264.715	10.2845
gyr	MS	0.0306	1.9569	0.0156	6.70E-173	1434	394.482	1039.52	275	264.113	10.8867
gyr	MS	0.0440	2.8297	0.0156	1.63E-214	1434	397.871	1036.13	323	310.426	12.5738
cit	GMYN	0.0117	0.7571	0.0155	1.91E-79	1311	365.424	945.576	160	149	11
adk	GMYN	0.0278	1.8006	0.0154	1.89E-66	663	149.552	513.448	116	101.997	14.0029
acsA	GMYN	0.0182	1.1909	0.0153	7.15E-145	1959	455.441	1503.56	267	239.983	27.0171
succ	MS	0.0121	0.7966	0.0152	2.38E-123	1776	527.008	1248.99	232	223.912	8.08822
gyr	MS	0.0421	2.8067	0.0150	1.76E-219	1422	370.516	1051.48	312	299.273	12.7274
iso	GMYN	0.0190	1.2797	0.0149	1.91E-167	2232	569.193	1662.81	327	295.802	31.1981
iso	GMYN	0.0364	2.4546	0.0148	4.93E-164	2220	511.193	1708.81	358	297.458	60.5418
gyr	GMYN	0.0295	1.9973	0.0148	5.21E-126	1434	417.706	1016.29	275	245.663	29.3372
succ	MS	0.0126	0.8678	0.0145	1.08E-139	1776	459.187	1316.81	233	223.668	9.33209
succ	MS	0.0126	0.8678	0.0145	1.08E-139	1776	459.187	1316.81	233	223.668	9.33209
gyr	MS	0.0400	2.7876	0.0144	2.61E-202	1434	380.038	1053.96	299	287.548	11.4519
CTP	GMYN	0.0292	2.0363	0.0143	9.17E-174	1659	389.391	1269.61	309	272.709	36.2908
CTP	GMYN	0.0292	2.0363	0.0143	9.17E-174	1659	389.391	1269.61	309	272.709	36.2908
succ	GMYN	0.0252	1.7650	0.0143	1.62E-172	1776	373.114	1402.89	288	253.238	34.7617
glnA	MS	0.0174	1.2335	0.0141	6.92E-87	873	232.469	640.531	140	134.776	5.22418
gyr	GMYN	0.0201	1.4627	0.0137	4.18E-91	894	225.047	668.953	158	144.779	13.2208
CTP	MS	0.0311	2.2809	0.0136	7.07E-232	1659	402.797	1256.2	324	310.796	13.2037
cit	GMYN	0.0235	1.7439	0.0135	6.66E-123	1299	258.548	1040.45	199	174.954	24.0462
gyr	MS	0.0394	2.9300	0.0135	8.63E-225	1431	361.43	1069.57	308	296.203	11.797
gyr	MS	0.0484	3.6212	0.0134	1.86E-215	1365	363.563	1001.44	305	294.174	10.8262
acsA	GMYN	0.0240	1.8174	0.0132	1.06E-149	1971	437.394	1533.61	283	246.884	36.1162
atpA	MS	0.0019	0.1442	0.0129	2.04E-30	1539	464.41	1074.59	59	57.2907	1.70933
atpA	GMYN	0.0074	0.5742	0.0129	2.82E-84	1539	449.593	1089.41	168	159.998	8.00241
gyr	GMYN	0.0295	2.2954	0.0128	1.71E-164	1434	379.947	1054.05	302	271.606	30.3935
gyr	GMYN	0.0432	3.3716	0.0128	2.55E-161	1434	375.774	1058.23	323	278.685	44.3148
adk	GMYN	0.0245	1.9126	0.0128	6.89E-54	663	159.511	503.489	103	90.8916	12.1084
atpA	GMYN	0.0103	0.8107	0.0128	2.82E-90	1536	366.785	1169.21	163	150.998	12.0025
atpA	MS	0.0073	0.5753	0.0127	1.77E-92	1539	437.524	1101.48	168	162.798	5.20194
succ	GMYN	0.0204	1.6253	0.0126	2.26E-165	1764	347.102	1416.9	259	230.516	28.4839
succ	GMYN	0.0216	1.7305	0.0125	1.09E-131	1776	408.645	1367.35	250	220.971	29.029
cit	GMYN	0.0194	1.5580	0.0124	2.85E-98	1311	316.295	994.705	186	166.995	19.0047

atpA	MS	0.0027	0.2263	0.0120	4.25E-44	1539	438.488	1100.51	83	80.5698	2.43025
atpA	MS	0.0029	0.2471	0.0119	5.50E-48	1539	416.33	1122.67	86	83.3313	2.66866
atpA	MS	0.0029	0.2471	0.0119	5.50E-48	1539	416.33	1122.67	86	83.3313	2.66866
glnA	GMYN	0.0166	1.4398	0.0116	8.00E-87	873	201.482	671.518	140	128.951	11.0489
gyr	MS	0.0425	3.6752	0.0116	7.32E-232	1434	365.786	1068.21	314	303.75	10.2497
gyr	MS	0.0394	3.4594	0.0114	1.43E-214	1434	402.787	1031.21	319	309.964	9.03628
cit	GMYN	0.0172	1.5183	0.0113	1.66E-120	1311	251.927	1059.07	182	163.991	18.0088
atpA	MS	0.0058	0.5249	0.0110	3.44E-87	1527	408.415	1118.59	150	145.618	4.3816
cit	MS	0.0071	0.6569	0.0108	2.40E-89	1311	330.451	980.549	144	139.534	4.46591
atpA	GMYN	0.0018	0.1646	0.0107	2.22E-32	1539	400.875	1138.13	59	57	2
cit	GMYN	0.0175	1.6527	0.0106	2.62E-89	1242	259.875	982.125	155	137.996	17.0036
iso	GMYN	0.0239	2.2572	0.0106	1.91E-182	2232	522.921	1709.08	345	304.774	40.2261
CTP	GMYN	0.0468	4.4215	0.0106	5.03E-186	1659	363.337	1295.66	345	286.354	58.6459
cit	GMYN	0.0072	0.6880	0.0105	2.31E-82	1311	333.787	977.213	144	136.999	7.00117
succ	GMYN	0.0232	2.2408	0.0104	6.18E-182	1776	351.706	1424.29	283	250.484	32.5163
atpA	GMYN	0.0054	0.5244	0.0103	9.82E-82	1527	415.773	1111.23	150	143.998	6.00155
CTP	GMYN	0.0455	4.3991	0.0103	4.97E-188	1659	352.653	1306.35	339	281.475	57.5253
gyr	MS	0.0294	2.9137	0.0101	9.18E-210	1434	389.71	1044.29	302	294.044	7.95621
CTP	GMYN	0.0446	4.4242	0.0101	1.94E-193	1647	364.641	1282.36	348	292.589	55.4106
CTP	GMYN	0.0283	2.8327	0.0100	1.49E-188	1659	394.251	1264.75	324	288.919	35.0806
CTP	MS	0.0157	1.5813	0.0099	1.40E-127	1044	271.359	772.641	188	182.824	5.1758
pyr	MS	0.0363	3.6677	0.0099	5.11E-210	1434	380.539	1053.46	297	289.072	7.92841
atpA	GMYN	0.0026	0.2655	0.0098	1.55E-49	1539	381.204	1157.8	83	80	3
CTP	GMYN	0.0430	4.4527	0.0097	6.11E-180	1656	378.766	1277.23	340	286.693	53.3066
acsA	MS	0.0123	1.2940	0.0095	1.95E-137	1323	346.255	976.745	210	204.51	5.48991
CTP	GMYN	0.0409	4.3859	0.0093	3.89E-177	1659	346.475	1312.52	318	265.895	52.1048
atpA	GMYN	0.0026	0.2821	0.0092	8.33E-50	1539	378.591	1160.41	86	82.9997	3.00028
atpA	GMYN	0.0026	0.2821	0.0092	8.33E-50	1539	378.591	1160.41	86	82.9997	3.00028
adk	GMYN	0.0217	2.3886	0.0091	2.01E-57	663	142.78	520.22	98	86.9152	11.0848
succ	GMYN	0.0161	1.7823	0.0090	7.29E-132	1776	393.031	1382.97	230	207.96	22.0404
gyr	GMYN	0.0392	4.3704	0.0090	5.64E-173	1431	339.397	1091.6	308	266.397	41.6028
atpA	MS	0.0049	0.5582	0.0088	3.11E-94	1539	415.525	1123.47	158	154.339	3.66115
iso	MS	0.0126	1.4558	0.0087	1.58E-164	1527	417.299	1109.7	255	249.242	5.75769
acsA	GMYN	0.0120	1.3882	0.0086	3.91E-135	1323	313.179	1009.82	210	197.983	12.0165

acsA	GMYN	0.0241	2.8319	0.0085	2.40E-161	1971	444.845	1526.16	299	262.838	36.1616
iso	GMYN	0.0154	1.8312	0.0084	3.51E-174	2232	513.728	1718.27	303	276.868	26.1316
succ	GMYN	0.0235	2.8881	0.0081	2.81E-134	1776	383.146	1392.85	249	216.877	32.1227
succ	GMYN	0.0113	1.4411	0.0078	5.91E-136	1776	427.859	1348.14	232	216.947	15.0531
ndk	GMYN	0.0031	0.3955	0.0078	7.47E-19	423	96.2147	326.785	29	28	1
gyr	MS	0.0198	2.5768	0.0077	6.94E-108	894	236.303	657.697	158	154.7	3.30038
iso	GMYN	0.0121	1.7223	0.0070	8.02E-170	1527	357.192	1169.81	255	240.939	14.0612
ndk	MS	0.0030	0.4310	0.0070	0	423	98.4718	324.528	29	28.3486	0.651395
succ	GMYN	0.0111	1.6003	0.0069	3.14E-141	1776	413.624	1362.38	233	218	15.0005
succ	GMYN	0.0111	1.6003	0.0069	3.14E-141	1776	413.624	1362.38	233	218	15.0005
ndk	GMYN	0.0030	0.4702	0.0065	3.64E-19	423	94.1205	328.879	29	28	1
ndk	GMYN	0.0030	0.4702	0.0065	3.64E-19	423	94.1205	328.879	29	28	1
acsA	GMYN	0.0179	2.7781	0.0065	3.70E-171	1971	443.175	1527.82	291	263.979	27.0206
ndk	MS	0.0029	0.4709	0.0063	0	423	87.6668	335.333	29	28.3217	0.678306
ndk	MS	0.0029	0.4709	0.0063	0	423	87.6668	335.333	29	28.3217	0.678306
cit	GMYN	0.0128	2.1119	0.0061	3.95E-105	1311	281.381	1029.62	168	154.948	13.0524
cit	GMYN	0.0128	2.1119	0.0061	3.95E-105	1311	281.381	1029.62	168	154.948	13.0524
atpA	GMYN	0.0044	0.7333	0.0060	2.83E-94	1539	394.052	1144.95	158	152.999	5.00095
CTP	GMYN	0.0163	2.9439	0.0055	1.49E-126	1044	238.02	805.98	188	174.992	13.0082
succ	GMYN	0.0231	4.3822	0.0053	2.19E-198	1773	344.8	1428.2	294	261.573	32.4274
cit	GMYN	0.0053	1.1520	0.0046	4.48E-88	816	242.962	573.038	146	142.999	3.00097
adk	GMYN	0.0073	1.6208	0.0045	1.83E-45	375	99.4572	275.543	68	65.9998	2.00022
adk	MS	0.0077	1.7370	0.0044	0	375	112.06	262.94	68	67.3013	0.698709
succ	MS	0.0052	1.2822	0.0041	6.65E-120	1137	286.368	850.632	171	168.951	2.04913
succ	GMYN	0.0047	1.2350	0.0038	6.38E-119	1137	275.818	861.182	171	167	4.00027
cit	MS	0.0055	1.8270	0.0030	0	816	267.637	548.363	146	145.107	0.892631
glnA	MS	0.0009	0.3288	0.0029	0	1413	376.781	1036.22	93	92.2678	0.732182
glnA	GMYN	0.0010	0.3391	0.0028	1.14E-57	1413	360.354	1052.65	93	91.9999	1.00008
ndk	MS	0.0000	0.8468	0.0000	0	225	71.2132	153.787	25	24.9999	5.40E-05
atpA	MS	0.0000	0.7320	0.0000	0	963	252.127	710.873	110	110	0.000310145
atpA	GMYN	0.0000	0.9391	0.0000	0	963	238.516	724.484	110	110	9.42E-10

GMYN = Approximate method (γ-MYN). Wang, D. P., Wan, H. L., Zhang, S., Yu, J. (2009). γ-MYN: a new algorithm for estimating Ka and Ks with consideration of variable substitution rates. *Biol. Direct*. 4, 20.

MS = Maximum-likelihood method. Posada, D. (2003). "Using Modeltest and PAUP* to select a model of nucleotide substitution", in Current Protocols in Bioinformatics, ed. A. D. Baxevanis (New York, USA: JohnWiley & Sons), 6.5.1-6.5.14.

Supplementary Table S12. Ka/Ks calculation using highly expressed selected genes and housekeeping genes from *Polynucleobacter asymbioticus* st QLW-P1DMWA-1^{T} and strains belonging to the PnecC subcluster.

Details of the analysed genes related to *Polynucleobacter asymbioticus* strain QLW-P1DMWA-1^{T}.

Locus Tag	Gene Product Name	Abbreviation	DNA Sequence Length (bp)
**Gene set**			
Pnuc_0690	DNA translocase FtsK	FtsK	2313
Pnuc_1769	Chaperone protein DnaK	Dank	1935
Pnuc_1805	Chaperonin GroEL	GroL	1653
Pnuc_1133	N-6 DNA methylase	methyl	1473
Pnuc_1736	Fatty acid desaturase	fatty	1200
Pnuc_1880	Holliday junction DNA helicase subunit RuvB	RuvB	1071
Pnuc_1929	RNA polymerase, sigma 32 subunit, RpoH	RpoH	930
Pnuc_0426	MscS Mechanosensitive ion channel	MscS	822
Pnuc_0725	Pirin domain protein	Piri	702
Pnuc_2030	Carbonate dehydratase	Carbo	666
Pnuc_0747	DSBA oxidoreductase	DSBA	627
Pnuc_0334	Adenylyl-sulfate kinase	CysC	588
Pnuc_1064	RNA polymerase, sigma-24 subunit, RpoE	RpoE	570
Pnuc_1626	Superoxide dismutase, copper/zinc binding	Superox	534
Pnuc_1828	Fimbrial protein pilin	Fimb	513
Pnuc_1376	Rubrerythrin	Rubre	486
Pnuc_0429	Alkyl hydroperoxide reductase	Alkyl	468
Pnuc_0425	Hypothetical protein	Hyp	267
Pnuc_1286	RNA-binding protein Hfq	Hfq	243
Pnuc_1750	Cold-shock DNA-binding protein family	Cold	204
**Housekeeping genes**			
Pnuc_0366	Isocitrate dehydrogenase, NADP-dependent	iso	2235
Pnuc_1138	Acetyl-coenzyme A synthetase (EC 6.2.1.1)	acsA	1974
Pnuc_0760	Succinate dehydrogenase subunit A (EC 1.3.5.1)	succ	1779
Pnuc_0945	CTP synthase (EC 6.3.4.2)	CTP	1662
Pnuc_0024	ATP synthase F1 subcomplex alpha subunit	atpA	1542
Pnuc_1816	Pyruvate kinase (EC 2.7.1.40)	gyr	1437
Pnuc_1255	L-glutamine synthetase (EC 6.3.1.2)	glnA	1416
Pnuc_0763	Citrate synthase (EC 2.3.3.1)	cit	1314
Pnuc_0281	Adenylate kinase (EC 2.7.4.3)	adk	666
Pnuc_1293	Nucleoside diphosphate kinase (EC 2.7.4.6)	ndk	426
