# Supplementary material for: Combined Methylome, Transcriptome and Proteome Analyses Document Rapid Acclimatization of a Bacterium to Environmental Changes
Source: Front Microbiol. 2020 Sep 15;11:544785. doi: 10.3389/fmicb.2020.544785 (PMC7522526; doi:10.3389/fmicb.2020.544785)
Supplement: Supplementary file 5 [file Data_Sheet_1.pdf]

Supplementary Files

Combined methylome, transcriptome and proteome analyses document rapid acclimatization of a bacterium to environmental changes

Abhishek Srivastava[1,2], Jayaseelan Murugaiyan[3,4], Juan AL Garcia[2], Daniele De Corte[5], Matthias Hoetzinger[6], Murat Eravci[7], Christoph Weise[7], Yadhu Kumar[8], Uwe Roesler[3], Martin W Hahn[9], Hans-Peter Grossart[1,10]

[1] Leibniz-Institute of Freshwater Ecology and Inland Fisheries, Alte Fischerhuette 2, 16775 Stechlin, Germany

[2] Department of Functional and Evolutionary Ecology, University of Vienna, Althanstrasse 14, 1090 Vienna, Austria

[3] Freie Universität Berlin, Institute for Animal Health and Environmental Hygiene, Centre for Infectious Medicine, Robert-von-Ostertag Strasse 7-13, 14163 Berlin, Germany

[4] SRM University-AP, Department of Biotechnology, Mangalagiri Mandal, Guntur District, Andhra Pradesh 522 502, India

[5] Research and Development Center for Marine Biosciences, Japan Agency for Marine-Earth Science and Technology (JAMSTEC), Natushima 2-15, Yokosuka, Kanagawa 237-0061, Japan

[6] Department of Biology and Environmental Science, Linnaeus University, Kalmar, Sweden

[7] Freie Universität Berlin, Institute of Chemistry and Biochemistry, Berlin, Germany

[8] Eurofins Genomics Europe Sequencing GmbH, Konstanz, Germany

[9] Research Department for Limnology, University of Innsbruck, Mondseestrasse 9, Mondsee 5310, Austria

[10] Potsdam University, Institute for Biochemistry and Biology, Am Neuen Palais 10, 14469 Potsdam, Germany

Supplementary Figure S1.

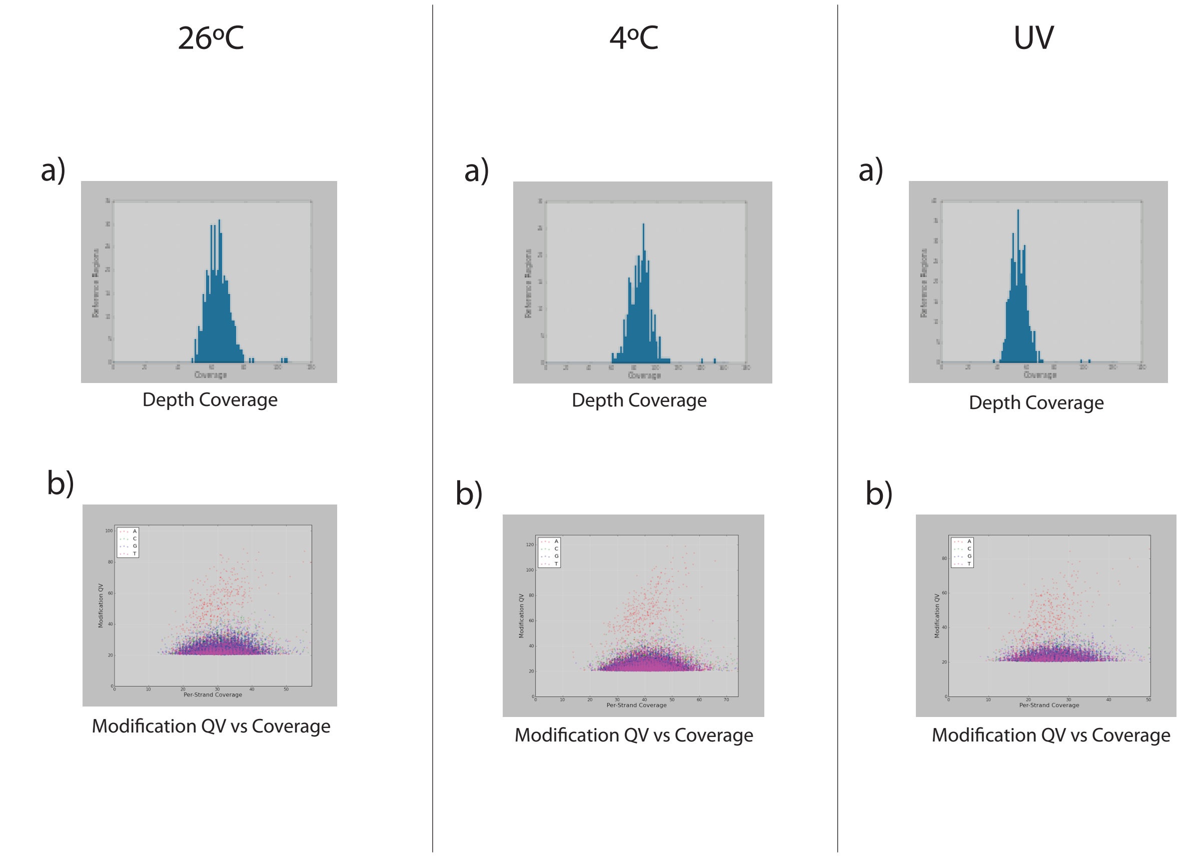

Supplementary Figure S1.

a). SMRT® sequencing of bacterial genome and its depth coverage (26ºC: 69.48, 4ºC: 65.33, UV: 54.23), and **b).** Relationships between base modification quality score and per strand coverage. A score of 30 was used as threshold for calling a modified position.

Supplementary Figure S2.

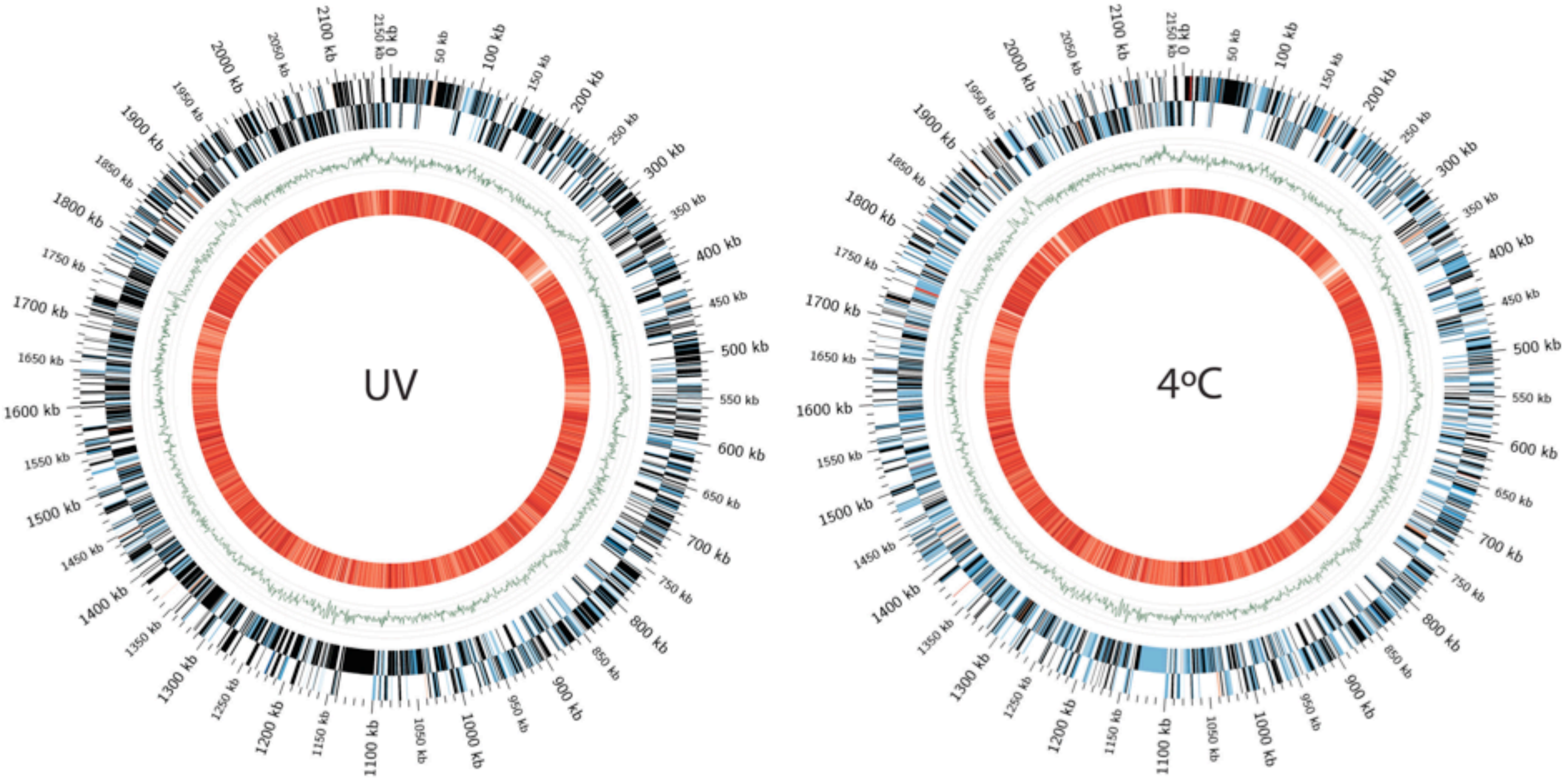

Supplementary Figure S2.

Circos plot representation of differentially expressed genes in bacteria grown at 26ºC* (UV irradiation) and cells grown at 4ºC (in relative comparison to 26ºC treated cells) and averaged melting energy distribution in bacterial genome. Outer two concentric tracks represent sense and antisense strands, respectively with given color-coding: red (upregulated genes) with transient color (expressed transcripts gradients) towards blue (downregulated genes), black genes are undetected in microarray analysis. Green GC skew curve is then followed with innermost track representing average negative DNA melting energy with 100-kb scanning window size (bright orange to pale yellow represents high to low values).

Supplementary Figure S3.

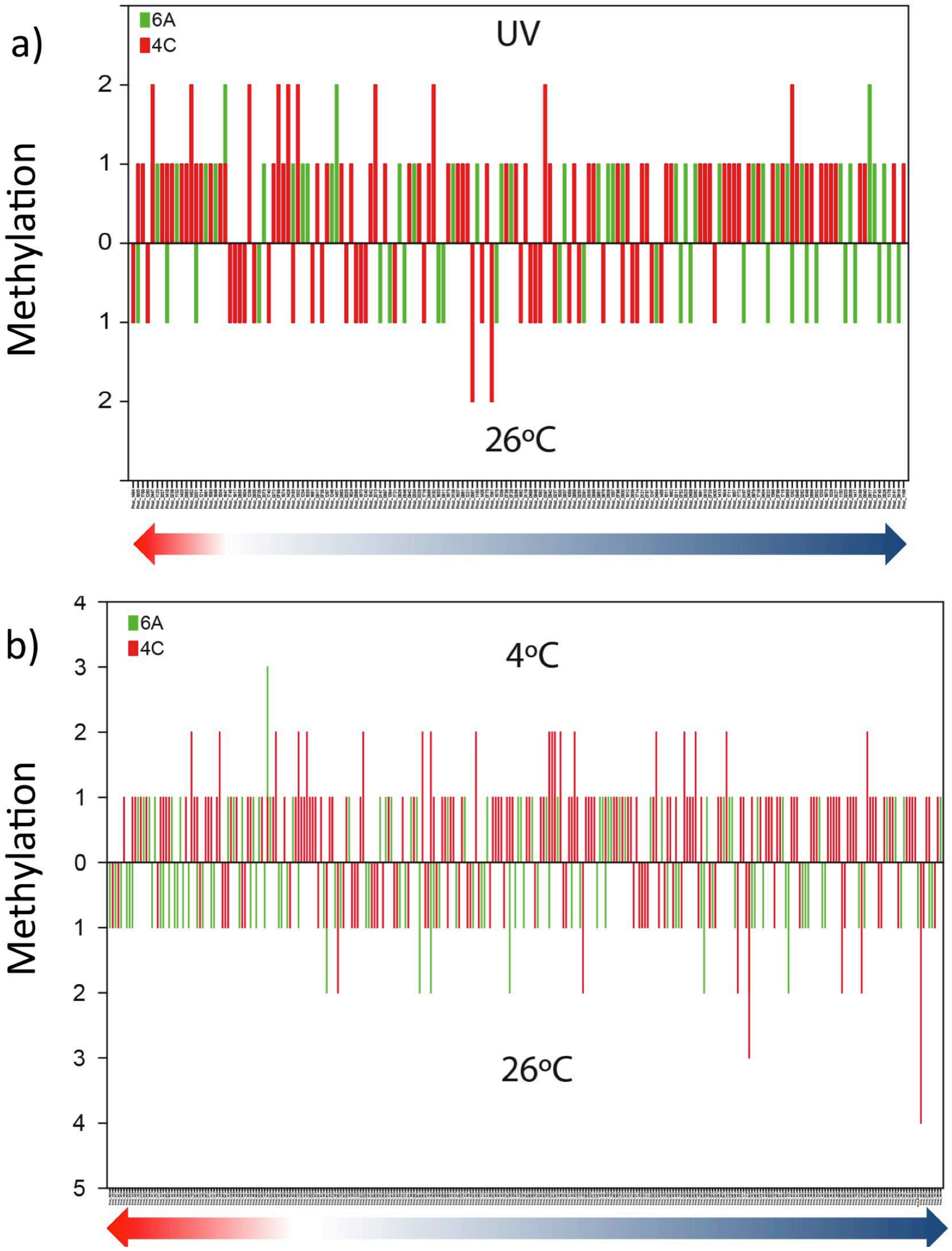

Supplementary Figure S3.

Adenine and cytosine methylation averaged distribution inside the differentially expressed genes. Red arrow: upregulation; blue represents downregulation of genes and methylation status in **a).** 26ºC* treated cells (top-half) and **b).** 4ºC treated cells (top-half), in compared with only 26ºC incubated cells (bottom-half in both sub-figures).

Supplementary Figure S4.

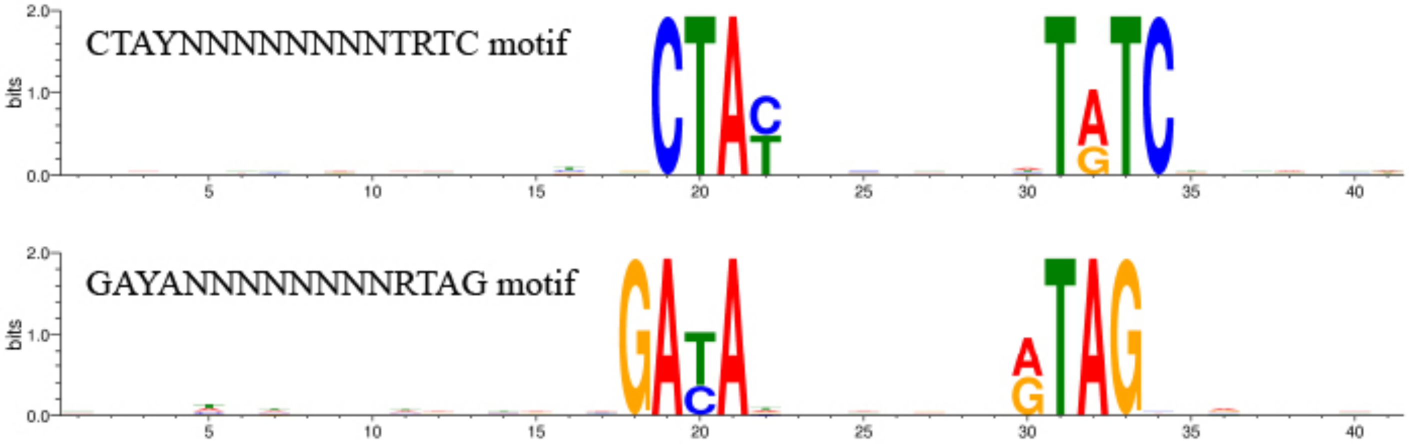

Supplementary Figure S4.

Weblogo depiction showing the potential binding motif of the methyltransferase responsible for modification on the adenine nucleotides (third adenine in CT**A**YNNNNNNNNTRTC motif and on fourth adenine in GAY**A**NNNNNNNNRTAG motif).

Supplementary Figure S5.

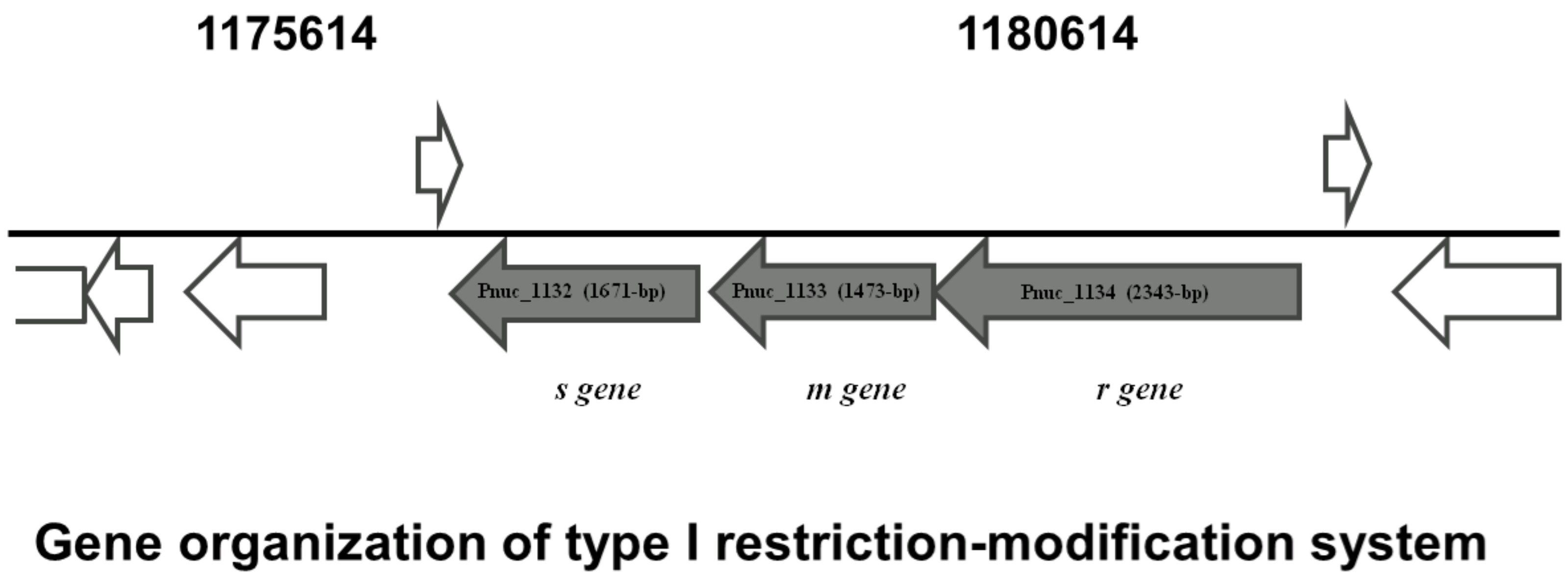

Supplementary Figure S5. Type I restriction modification system in *P. asymbioticus* str. QLW-P1DMWA-1^{T}.

We detected only one copy of type I restriction modification system in the genome and two candidate genes (Pnuc_0029, Pnuc_0929) possibly involved in the type III restriction modification process.
